# Burden of psoriasis in young adults worldwide from the global burden of disease study 2019

**DOI:** 10.3389/fendo.2024.1308822

**Published:** 2024-02-13

**Authors:** Yuanchen Zhang, Shuai Dong, Yuan Ma, Yan Mou

**Affiliations:** Second Affiliated Hospital of Jilin University, Changchun, China

**Keywords:** global disease burden, young adults, psoriasis, age-standardized DALY rate, ASIR, estimated annual percentage change

## Abstract

**Background:**

To determine the global burden of psoriasis in young adults, i.e., those aged 15–49, from 1990 to 2019 and predict trends in this burden for 2020 to 2030

**Methods:**

Age-standardized disease burden indicators and their estimated annual percentage changes were assessed and used to compare the estimated burden between regions. In addition, generalized additive models were used to predict the burden in this population from 2020 to 2030.

**Results:**

From 1990 to 2019, the overall burden of psoriasis in young adults worldwide trended downward, as the age-standardized incidence rate and the age-standardized disability-adjusted life year rate decreased. From 1990 to 2019, there were gender differences in the burden of psoriasis between regions with different Socio-demographic index. Specifically, there was a smaller increase in the burden in young men than in young women in middle- and low–middle-Socio-demographic index areas. In 2019, Western Europe, Australasia, and Southern Latin America had the highest age-standardized incidence rate of psoriasis in young adults, whereas age-standardized disability-adjusted life year rates of psoriasis in young adults were highest in high-income North America. In 2019, the psoriasis burden in young adults was the highest in high-Socio-demographic index areas and the lowest in low-Socio-demographic index regions. We predict that from 2020 to 2030, the incidence rate and disability-adjusted life year rate of psoriasis in all age groups of young adults will continue to decline, but the burden in those aged 30–39 will increase.

**Conclusion:**

From 1990 to 2019, the overall burden of psoriasis in each age group trended downward in this period. We predict that from 2020 to 2030, the burden of psoriasis in those aged 30–39 will increase.

## 1 Introduction

Psoriasis is a common chronic skin disease that has substantial adverse effects on the health and well-being of many people worldwide (1, 2). Currently, over 125 million people worldwide are affected by psoriasis, and this number is increasing year by year. As psoriasis is a long-term health problem, it not only causes chronic physical discomfort to patients but also decreases their psychological and social well-being, thereby decreasing their overall quality of life (3, 4). Therefore, comprehensive research on psoriasis to identify its risk factors, develop effective treatments and preventive methods, and understand its pathogenesis is a key focus in the fields of dermatology and global public health. Such research can improve patients’ quality of life and lead to enormous social and economic benefits.

Young adults are the main labor force of countries, and thus, their health directly affects the economic and cultural development of societies. Moreover, as healthy behaviors often persist as people age, those related to diet, exercise, drug use, and mental health can have long-term effects. Thus, maintaining the health of young adults can support their future health and well-being and thereby benefit society. The risk of psoriasis in younger adults has gradually increased over the past few decades, which has aroused widespread concern in the medical and public health field, as the physical and psychological stress associated with psoriasis may decrease young adults’ quality of life, work efficiency, and social activity. This highlights the need for effective methods for the prevention and treatment of psoriasis in young adults. However, few studies have evaluated the burden of psoriasis in young adults at the global and national levels (5)and the temporal changes in this burden, and such studies are needed to devise global preventive strategies.

Accordingly, we analyzed the Global Burden of Disease Study (GBD) data in 2019 to determine the incidence number of psoriasis, the age-standardized incidence rate (ASIR) of psoriasis, the number of disability-adjusted life years (DALYs) due to psoriasis, and the age-standardized DALY rate due to psoriasis in young adults worldwide from 1990 to 2019, and the estimated annual percentage changes (EAPCs) in the aforementioned parameters. The results reveal regional and age-group distinctions in the psoriasis burden in young adults over this 30-year period. We also considered various potential risk and protective factors and analyzed the data using generalized additive models (GAMs) to predict possible trends in the global burden of psoriasis in young adults from 2020 to 2030. This enabled us to make recommendations and suggest strategies that public health decision-makers could employ to alleviate this burden.

## 2 Method

### 2.1 Data

This study used data from the GBD 2019, which is the most recent study issued by a global epidemiological project that involves more than 3600 experts in a total of 145 countries and aims to assess the cost of illnesses, injuries, and risk causes worldwide. The GBD 2019 analyzed a large number of published studies, surveys, and sets of epidemiological data on more than 350 health outcomes and risk factors and thus afforded the incidence rates, morbidity, mortality, DALYs, and other data on multiple diseases in 204 countries and regions worldwide (6, 7). The GBD updates its databases annually to ensure that they reflect the dynamic changes in the global burden of diseases, which helps to predict future demand for health services. The Second Hospital of Jilin University reviewed and approved this study.

The Socio-development Index (SDI) is a comprehensive index composed of three indicators: per capita income, schooling level, and total fertility rate. It is widely used to evaluate and compare the socio-economic development levels of different nations and areas. In the GBD, the SDI is used to reveal the relationship between disease burdens and socio-economic development.

The clinical diagnosis of psoriasis was based on the Ninth Revision of the International Statistical Classification of Diseases and Related Health Problems (ICD-9), in which the code for psoriasis is 696.1. In the ICD-10, the code for psoriasis is L40.

### 2.2 Statistical analysis

In our study, we employed the GBD methodology to assess the impact of psoriasis among young adults on a global scale. This involved utilizing standardized population-age structures to estimate the global burden of psoriasis. Our approach combined age-standardized incidence rates (ASIR) data with disability weights (DW) to compute the DALYs attributed to psoriasis in young adults. Furthermore, we calculated the Estimated Annual Percentage Changes (EAPCs) in the ASIRs and the age-standardized DALY rates for psoriasis within this demographic (8, 9).

We evaluated the health impact of psoriasis in young adults by calculating the age normalization rate and its associated uncertainty intervals (UIs) based on global standards (World Health Organization, 2000–2025) using the equation below:


$$\frac{\sum_{\text{i}=1}^{\text{A}}{\text{a}}_{i\text{wi}}}{\sum_{1=1}^{\text{A}}{\text{w}}^{i}}$$

where ai is the age-specific rate of the ith age group, and wi is the weight of this age group in the selected reference standard population.

We conducted 1000 Monte Carlo simulations to estimate the 95% Uncertain Intervals (UIs) for each incidence rate, mortality rate, and DALYs associated with psoriasis. For these intervals, we designated the 2.5th percentile as the lower bound and the 97.5th percentile as the upper bound, ensuring a 95% probability that the parameter of interest falls within this range. The interpretation of the EAPC in the ASIR of psoriasis was as follows: if the EAPC’s lower bound and the entire 95% CI were above zero, it suggested an increasing trend in the global ASIR of psoriasis. Conversely, if the EAPC’s upper bound and the entire 95% CI were below zero, this indicated a decreasing trend in the global ASIR. In cases where the 95% CI included zero, it implied that the ASIR of psoriasis globally remained stable over time, indicating no significant upward or downward trend.

## 3 Results

### 3.1 Trends in the burden of psoriasis in young adults worldwide from 1990 to 2019

**Figure 1:**
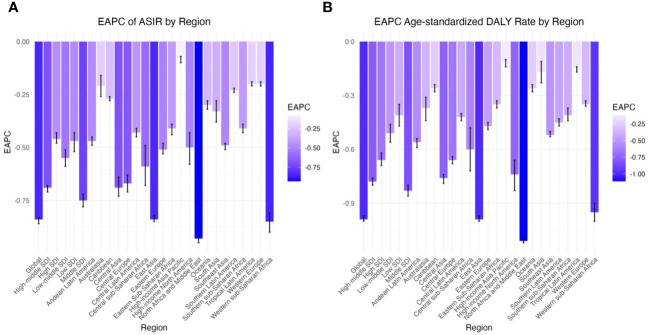
EAPC of global psoriasis burden of young adults, by Regions. **(A)** EAPC of age standardized incidence rate **(B)** EAPC of age standardized DALY rate.

From 1990 to 2019, the overall burden of psoriasis in young adults worldwide trended downward, and in various regions, the ASIR and age-standardized DALY rate of psoriasis were both less than zero. In particular, in young adults worldwide, the ASIR of psoriasis decreased from 76.57 (95% CI: 76.47, 76.68) in 1990 to 59.87 (95% CI: 59.79, 59.95) in 2019, and the age-standardized DALY rate decreased from 55.67 (95% CI: 55.58, 55.77) in 1990 to 41.84 (95% CI: 41.77, 41.90) in 2019. Moreover, from 1990 to 2019, the EAPC in the ASIR was -0.84 (95% CI: -0.86, -0.83), and the EAPC in the age-standardized DALY rate was -0.99 (95% CI: -1.00, -0.97) (**Tables 1**, **2**; **Figure 1**).

**Table 1:** The age-standardized incidence rate of global psoriasis burden of young adults from 1990 to 2019 in different regions.

Location	1990	Male/Female	2019	Male/Female	1990-2019
	Age-standardized incidence rate (per 100000)		Age-standardized incidence rate (per 100000)		EAPC
	No. (95%UI)		No. (95%UI)		No. (95%CI)
Global	76.57 (76.47,76.68)	1.05	59.87 (59.79,59.95)	1.04	-0.84 (-0.86,-0.83)
Sociodemographic index	–	–	–	–	–
High-middle SDI	90.15 (89.91,90.40)	1.05	73.30 (73.10,73.50)	1.05	-0.69 (-0.71,-0.68)
High SDI	133.14 (132.80,133.48)	1.06	118.31 (118.00,118.63)	1.03	-0.46 (-0.48,-0.43)
Low-middle SDI	56.15 (55.94,56.36)	0.99	47.78 (47.63,47.92)	0.99	-0.55 (-0.59,-0.51)
Low SDI	47.68 (47.39,47.98)	0.96	41.85 (41.67,42.03)	0.97	-0.47 (-0.52,-0.43)
Middle SDI	57.41 (57.24,57.57)	1.07	45.73 (45.61,45.85)	1.03	-0.75 (-0.78,-0.72)
Region	–	–	–	–	–
Andean Latin America	96.52 (95.05,98.02)	0.95	83.83 (82.84,84.83)	0.95	-0.47 (-0.49,-0.45)
Australasia	167.94 (165.50,170.40)	0.96	155.56 (153.46,157.67)	0.96	-0.21 (-0.26,-0.16)
Caribbean	63.48 (62.29,64.68)	0.95	59.03 (58.06,60.01)	0.95	-0.27 (-0.28,-0.26)
Central Asia	80.19 (79.17,81.21)	1.06	66.41 (65.68,67.14)	1.09	-0.69 (-0.73,-0.64)
Central Europe	77.19 (76.49,77.89)	1.08	64.20 (63.51,64.90)	1.11	-0.67 (-0.71,-0.63)
Central Latin America	24.57 (24.22,24.94)	0.95	21.59 (21.34,21.84)	0.96	-0.43 (-0.45,-0.41)
Central sub-Saharan Africa	64.18 (63.11,65.26)	0.95	53.37 (52.77,53.98)	0.96	-0.59 (-0.68,-0.49)
East Asia	74.98 (74.76,75.19)	1.16	58.55 (58.38,58.73)	1.16	-0.84 (-0.85,-0.82)
Eastern Europe	71.69 (71.19,72.20)	1.03	62.34 (61.84,62.85)	1.05	-0.51 (-0.53,-0.48)
Eastern Sub-Saharan Africa	29.11 (28.72,29.51)	0.94	25.85 (25.61,26.09)	0.95	-0.41 (-0.44,-0.39)
High-income Asia Pacific	44.71 (44.28,45.14)	0.99	43.10 (42.65,43.56)	1.03	-0.08 (-0.10,-0.07)
High-income North America	105.23 (104.71,105.75)	1.05	93.97 (93.51,94.43)	1.03	-0.50 (-0.58,-0.43)
North Africa and Middle East	79.81 (79.35,80.27)	0.91	61.24 (60.98,61.51)	0.91	-0.93 (-0.95,-0.92)
Oceania	46.02 (43.55,48.61)	1.06	41.72 (40.17,43.33)	1.06	-0.30 (-0.32,-0.28)
South Asia	51.32 (51.12,51.52)	0.96	46.75 (46.61,46.89)	0.96	-0.33 (-0.38,-0.28)
Southeast Asia	23.82 (23.61,24.03)	0.97	20.50 (20.35,20.65)	0.96	-0.49 (-0.51,-0.48)
Southern Latin America	117.40 (116.04,118.77)	0.99	109.43 (108.32,110.54)	1.00	-0.23 (-0.24,-0.22)
Southern sub-Saharan Africa	38.94 (38.14,39.75)	0.94	34.71 (34.15,35.28)	0.95	-0.41 (-0.43,-0.39)
Tropical Latin America	94.49 (93.79,95.20)	0.95	88.77 (88.23,89.30)	0.95	-0.20 (-0.21,-0.19)
Western Europe	239.55 (238.87,240.24)	1.06	226.20 (225.53,226.88)	1.07	-0.20 (-0.21,-0.19)
Western sub-Saharan Africa	42.83 (42.37,43.29)	0.95	33.98 (33.72,34.24)	0.96	-0.85 (-0.90,-0.81)

**Table 2:** The age-standardized DALY rate of global psoriasis burden of young adults from 1990 to 2019 in different regions.

Location	1990	Male/Female	2019	Male/Female	1990-2019
	Age-standardized DALY rate (per 100000)		Age-standardized DALY rate (per 100000)		EAPC
	No. (95%UI)		No. (95%UI)		No. (95%CI)
Global	55.67 (55.58,55.77)	1.00	41.84 (41.77,41.90)	1.01	-0.99 (-1.00,-0.97)
Sociodemographic index	–	–	–	–	–
High-middle SDI	64.77 (64.56,64.97)	0.99	50.94 (50.78,51.11)	0.99	-0.78 (-0.80,-0.76)
High SDI	110.75 (110.44,111.06)	1.00	93.84 (93.56,94.11)	0.97	-0.66 (-0.69,-0.62)
Low-middle SDI	36.84 (36.67,37.01)	0.99	31.65 (31.53,31.76)	1.00	-0.51 (-0.56,-0.46)
Low SDI	30.59 (30.36,30.83)	0.98	27.28 (27.14,27.43)	0.99	-0.41 (-0.47,-0.35)
Middle SDI	37.80 (37.67,37.93)	0.99	29.51 (29.41,29.60)	0.97	-0.83 (-0.86,-0.80)
Region	–	–	–	–	–
Andean Latin America	75.09 (73.79,76.41)	0.98	63.27 (62.42,64.14)	0.98	-0.56 (-0.59,-0.54)
Australasia	158.09 (155.74,160.47)	0.85	138.18 (136.22,140.16)	0.84	-0.37 (-0.44,-0.31)
Caribbean	40.10 (39.16,41.06)	0.97	37.53 (36.76,38.32)	0.97	-0.26 (-0.28,-0.24)
Central Asia	52.81 (51.99,53.65)	1.04	42.39 (41.82,42.98)	1.05	-0.76 (-0.79,-0.74)
Central Europe	49.86 (49.30,50.43)	1.06	41.02 (40.46,41.58)	1.06	-0.66 (-0.68,-0.64)
Central Latin America	13.81 (13.54,14.08)	0.97	12.20 (12.01,12.39)	0.97	-0.42 (-0.44,-0.40)
Central sub-Saharan Africa	45.95 (45.04,46.87)	0.98	37.69 (37.19,38.20)	0.98	-0.60 (-0.72,-0.48)
East Asia	51.22 (51.04,51.40)	1.02	38.48 (38.34,38.62)	1.01	-0.99 (-1.00,-0.97)
Eastern Europe	44.80 (44.41,45.20)	0.99	38.95 (38.56,39.36)	0.99	-0.47 (-0.49,-0.45)
Eastern Sub-Saharan Africa	16.78 (16.48,17.08)	0.95	15.18 (15.00,15.36)	0.97	-0.35 (-0.37,-0.33)
High-income Asia Pacific	25.60 (25.28,25.93)	0.96	24.35 (24.01,24.70)	1.00	-0.12 (-0.14,-0.10)
High-income North America	109.27 (108.74,109.80)	0.94	93.10 (92.64,93.56)	0.93	-0.74 (-0.83,-0.66)
North Africa and Middle East	51.82 (51.44,52.19)	0.90	37.82 (37.61,38.03)	0.89	-1.11 (-1.12,-1.09)
Oceania	26.79 (24.91,28.79)	0.96	24.54 (23.35,25.78)	0.95	-0.26 (-0.28,-0.24)
South Asia	32.31 (32.15,32.47)	0.99	30.77 (30.66,30.88)	0.99	-0.17 (-0.23,-0.11)
Southeast Asia	13.32 (13.17,13.48)	0.87	11.42 (11.31,11.53)	0.86	-0.52 (-0.53,-0.50)
Southern Latin America	92.54 (91.33,93.76)	0.94	80.56 (79.62,81.52)	0.95	-0.45 (-0.47,-0.43)
Southern sub-Saharan Africa	23.06 (22.45,23.68)	0.96	20.56 (20.13,21.00)	0.98	-0.41 (-0.44,-0.37)
Tropical Latin America	70.56 (69.96,71.17)	0.98	67.17 (66.71,67.63)	0.98	-0.15 (-0.17,-0.14)
Western Europe	191.46 (190.85,192.08)	1.04	172.50 (171.91,173.08)	1.05	-0.35 (-0.36,-0.33)
Western sub-Saharan Africa	26.93 (26.57,27.30)	0.97	20.81 (20.60,21.01)	0.98	-0.95 (-1.00,-0.90)

### 3.2 Age distribution of the burden of psoriasis in young adults worldwide in 2019

**Figure 2:**
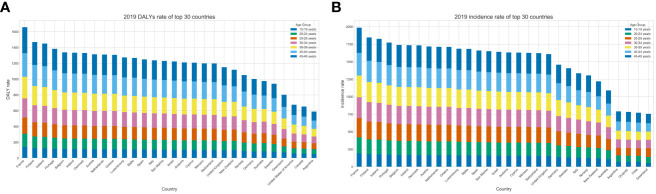
The rate for global psoriasis burden of young adults in 2019, by top 30 country and age group. **(A)** DALY rate **(B)** Incidence rate.

**Figure 3:**
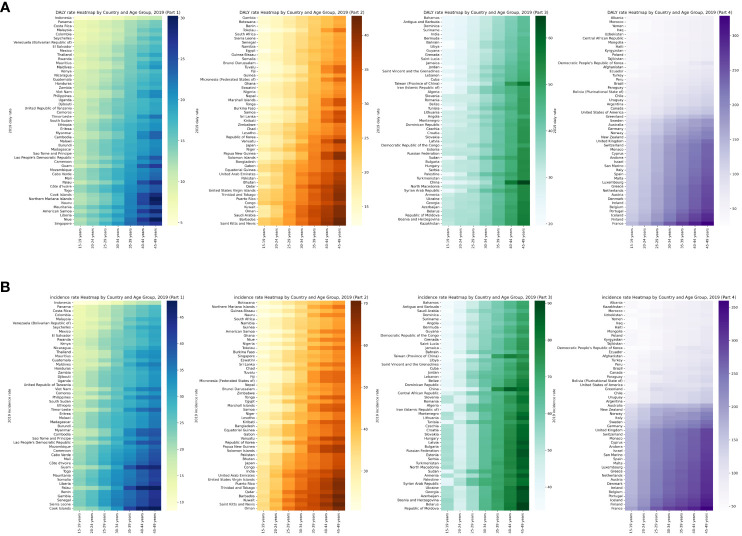
Heatmap of rate for global psoriasis burden of young adults in 2019, by country and age group. **(A)** DALY rate **(B)** Incidence rate.

In 2019, there was no particularly tremendous distinction in the overall burden of psoriasis in distinct age groups of young adults worldwide, but the burden nevertheless trended upward with age, especially in countries whose young adults had a high burden of psoriasis (**Figure 2**). We classified 204 countries into four groups based on their age-standardized rates (ASRs) of psoriasis and then constructed a heatmap of age groups. We found that the increase in the burden of psoriasis was not significant in those aged 15–29 but was significant in those aged 30–49 (**Supplementary Tables 3**, **4**; **Figures 2**, **3**).

### 3.3 Gender distribution of the burden of psoriasis in young adults worldwide from 1990 to 2019

**Figure 4:**
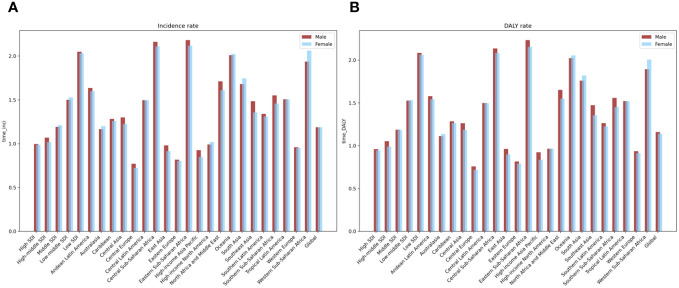
The EAPC of global psoriasis burden of young adults from 1990 to 2019, by sex and regions. **(A)** Age standardized incidence rate **(B)** age-standardized DALY rate.

From 1990 to 2019, the overall burden of psoriasis in young adults worldwide trended downward. However, the growth trends of the ASIR and age-standardized DALY rate of psoriasis in young people presented that the burden of psoriasis differed between gender and between regions with different levels of economic development. Specifically, from 1990 to 2019 and based on the EAPCs in the ASIR and age-standardized DALY rate of psoriasis in young people, the burden increased more in young men than in young women in high-SDI, high–middle-SDI, and low-SDI regions, whereas it increased more in young women than in young men in middle-SDI and low–middle-SDI areas (**Tables 1**, **2**; **Supplementary Tables 1**, **2**; **Figure 4**).

### 3.4 Geographical distribution of the burden of psoriasis in young adults worldwide from 1990 to 2019

In 2019, among the 21 geographical regions worldwide, the ASIR of psoriasis in young adults was highest in Western Europe (226.20), followed by Australasia (155.56) and Southern Latin America (109.43), whereas it was lowest in Southeast Asia (20.50), followed by Central Latin America (21.59) and Eastern sub-Saharan Africa (25.85). From 1990 to 2019, the ASIR of psoriasis in young adults worldwide trended downward; the smallest decreases occurred in Tropical Latin America (EAPC = -0.20) and Western Europe (EAPC = -0.20), followed by High-income Asia Pacific (EAPC = -0.08), whereas the largest decreases occurred in North Africa and Middle East (EAPC = -0.93), Western sub-Saharan Africa (EAPC = -0.85), and East Asia (EAPC = -0.84) (**Tables 1**, **2**; **Supplementary Tables 5**, **6**).

In 2019, among the 21 geographical regions worldwide, the age-standardized DALY rate of psoriasis in young adults was highest in Western Europe (172.50), followed by Australasia (138.18) and High-income North America (93.10), whereas it was lowest in Southeastern Asia (11.42), followed by Central Latin America (12.20) and Eastern sub-Saharan Africa (15.18). From 1990 to 2019, the age-standardized DALY rate decreased the least in South Asia (-0.17), Tropical Latin America (-0.15), and High-income Asia Pacific (-0.12), whereas it decreased the most in North Africa and Middle East (-1.11), East Asia (-0.99), and Western sub-Saharan Africa (-0.95) (**Tables 1**, **2**; **Supplementary Tables 5**, **6**).

ASIRs and age-standardized DALY rates of psoriasis were lowest in young adults in South Asia, Central Latin America, and Eastern sub-Saharan Africa in 2019 and in young adults in North Africa and Middle East, Western sub-Sahara African, and East Asia from 1990 to 2019 (**Supplementary Tables 5**, **6**).

In 2019, among 204 countries worldwide, the ASIRs and age-standardized DALY rates of psoriasis were highest in young adults in France, Finland, and Iceland. From 1990 to 2019, among 204 countries worldwide, the EAPC in the ASIR was highest in Japan (EAPC = 0.04), Sweden (EAPC = -0.08), and Somalia (EAPC = -0.09), and the EAPC in the age-standardized DALY rate was highest in Somalia (EAPC = 0.14), Japan (EAPC = 0.04), and Kiribati (EAPC = -0.01) (**Supplementary Tables 5**, **6**, **7**, **8**; **Supplementary Figure 1**).

### 3.5 Relationship between the burden of psoriasis in young adults and the SDI of regions from 1990 to 2019

In 2019, the burden of psoriasis in young adults varied with regions’ SDIs. The ASIR of psoriasis in young adults in 2019 was highest in high-SDI regions (118.31, 95% CI: 118.00, 118.63) and lowest in low-SDI regions (41.85, 95% CI: 41.67, 42.03). Similarly, age-standardized DALY rate of psoriasis in young adults in 2019 was highest in high-SDI regions (93.84, 95% CI: 93.56, 94.11) and lowest in low-SDI regions (27.28, 95% CI: 27.14, 27.43 (**Tables 1**, **2**).

From 1990 to 2019, the trend in the burden of psoriasis in young adults worldwide varied with regions’ SDIs, with the ASIRs and age-standardized DALY rates showing the same trend. Regression-fitting the trend in the burden in various countries revealed that as SDIs changed from 0 to 1, the burden almost always showed a cyclical change, i.e., first increased and then decreased. Specifically, the burden increased as the SDI increased from 0 to 0.6; decreased as the SDI increased from 0.6 to 0.7; and reached a maximum and then decreased to a trough as the SDI increased to approximately 0.8. This trend indicates that young adults in countries with SDIs of approximately 0.8 had a high burden of psoriasis, whereas young adults in countries with SDIs that were extremely low, extremely high, and approximately 0.7 had low burden of psoriasis. From 1990 to 2019 in 204 countries worldwide, the relationship between SDIs and the burden followed a similar pattern. As SDIs increases, the burden of psoriasis in young adults fluctuated only slightly around 0.6, showing little change overall (**Tables 1**, **2**; **Supplementary Tables 1**, **2**; **Supplementary Figures 2**, **3**).

### 3.6 Prediction of the burden of psoriasis in young adults worldwide from 2020 to 2030

We fitted data from 1990 to 2019 to GAMs to predict the trend changes in the burden of psoriasis in young adults worldwide from 2020 to 2030.

Fitting the trends in incidence number, incidence rate, number of DALYs, and DALY rate for distinct age groups from 1990 to 2019 to GAMs revealed that the overall incidence rate and DALY rate for each age group trended downward in this period. However, there were some between-age-group differences in the incidence number and number of DALYs. From 1990 to 2019, the incidence number and number of DALYs peaked first in those aged 15–19, then in those aged 20–24, and finally in those aged 25–29. Similarly, the incidence number and number of DALYs peaked first in those aged 30–34, then in those aged 35–39, those aged 40–44, and finally in those aged 45–49. In addition, overall, the peak values in those aged 15–29 were higher than in those aged 30–49 (**Tables 1**, **2**; **Supplementary Tables 1**, **2**; **Supplementary Figure 4**).

According to our predictions, from 2020 to 2030, the overall incidence rate and DALY rate of psoriasis in each age group will trend downward. However, we predict that the incidence and number of DALYs will increase in those aged 30–34 and 35–39, respectively, but decrease in other age groups. Thus, up until 2030, efforts should be focused on alleviating the burden of psoriasis in those aged 30–39 (**Supplementary Figure 4**)

## 4 Discussion

This study represents the most recent investigation into the global burden psoriasis among young adults, utilizing data from the Global Burden of Disease Study 2019. Our findings reveal a steady decrease in the overall burden of psoriasis from 1990 to 2019. Specifically, both the age-standardized incidence rate (ASIR) and the age-standardized disability-adjusted life years (DALY) rate demonstrated negative estimated annual percentage changes (EAPCs), indicating a downward trend in disease burden over time.

There are several possible explanations for the decline in the incidence of psoriasis among young adults across the globe. Firstly, significant progress has been made in the treatment of psoriasis psoriasis in the country. Conventional treatment options for psoriasis, such as corticosteroids, Vitamin D analogues, phototherapy and systemic treatments, have shown positive results. In cases where these traditional treatments have been ineffective, contraindicated or resulted in severe adverse effects, biological therapies have been recommended. These advancements in treatment have been made possible through collaborative efforts among clinical physicians, patients, the academic community and the pharmaceutical industry (10). Secondly, advancements in medical technology may have contributed to the improved effectiveness of early diagnosis and treatment of psoriasis, thereby reducing the mortality rate and burden of the disease among young adults worldwide (11). However, it is important to consider that these advancements in medical technology and diagnosis might also have led to an increase in the number of cases of identified and reported psoriasis cases, subsequently, increasing the overall burden of the disease. Thirdly, health education initiatives focusing on raising public awareness about psoriasis risk factors may have prompted young adults to make positive lifestyle changes, such as by reducing prolonged long-term exposure to sunlight, thereby potentially loweringg their risk of developing psoriasis.

However, there was a notable rise in the incidence among those aged 30-49. Psoriasis can manifest at any stage of life. However, recent estimates indicate that the average age of onset for psoriasis vulgaris is 33 years, with 75% of cases occurring before the age of 46 (12). Research suggests that the onset of psoriasis follows a bimodal distribution, with peaks between 16 and 22 years, and later between 57 and 60 years. Additionally, it appears that women tend to experience the onset of psoriasis slightly earlier than men (12).

From 1990 to 2019, the analysis of the gender-specific burden of psoriasis among young adults revealed distinct patterns across regions with varying socio-demographic indices (SDIs). Notably, a more pronounced increase in the psoriasis burden was observed among young men compared to young women in regions classified as high-SDI, high–middle-SDI, and low-SDI. This difference could be linked to several factors that disproportionately affect young men in these regions. For example, occupational exposures to psoriasis risk factors, such as chemicals, might be more common among young men due to the nature of their employment. Additionally, lifestyle factors, including higher levels of stress and potentially lower rates of seeking medical advice or acknowledging early psoriasis symptoms, could contribute to this observed discrepancy, leading to delayed diagnosis and management (13).

Conversely, in middle-SDI and low–middle-SDI regions, the data suggested a greater increase in psoriasis burden among young women than in young men. This trend could be influenced by a complex interplay of lifestyle, genetic, and environmental factors uniquely impacting young women in these areas. Hormonal changes, particularly around puberty and perimenarchal periods, have been implicated in modulating the risk and severity of psoriasis, potentially explaining the increased incidence among young women. Furthermore, exposure to certain environmental triggers, such as specific medications and cosmetic products, might also play a role in the differential gender-based incidence observed in these regions (14).

From 1990 to 2019, the gender distribution of the burden of psoriasis in young adults worldwide varied according to the regions’ socio-demographic indices (SDIs). Firstly, the burden exhibited a higher growth rate in young men compared to in young women in high-SDI, high–middle-SDI, and low-SDI regions. This discrepancy may be attributable to the higher exposure of young men in these areas to risk factors for psoriasis, such as chemicals in the work environment, and life stresses (13). Moreover, young men in these regions may demonstrate a lesser inclination to seek medical assistance or overlook early symptoms of psoriasis, resulting in disease progression. Conversely, in middle-SDI and low–middle-SDI regions, the burden’s growth rate was higher in young women than in young men. This pattern may arise due to changes in young women’s lifestyles, genetics, and environmental factors in these regions. For instance, compared to young men, young women in these areas may experience greater hormonal fluctuations, as supported by reports of a perimenarchal increase in the prevalence of psoriasis, or be more exposed to triggers for psoriasis, such as certain drugs and cosmetics.

From 1990 to 2019, there have been significant differences in the geographical distribution of psoriasis burden among young adults worldwide. In 2019, Western Europe, Australasia, and Southern Latin America had the highest burden, while Southeast Asia, Central Latin America, and Eastern sub-Saharan Africa had the lowest burden. Multiple factors, including differences in sun exposure and climate, likely contribute to the higher incidence of psoriasis in countries farther from the equator (15). Over the same time period, the burden of psoriasis in young adults has generally decreased in most regions, albeit at different rates. The fastest decreases were observed in North Africa, the Middle East, Western sub-Saharan Africa, and East Asia. One study suggests that dietary factors, such as a high lineolic acid intake seen in parts of Africa, may play a protective role (16). This improvement in burden may be attributed to advancements in prevention methods and treatments for psoriasis (17). In contrast, Tropical Latin America, Western Europe, and High-income Asia Pacific had the slowest decreases, which could be partly attributed to the increasing prevalence of obesity, a known risk factor for psoriasis (18). France, Finland, and Iceland had the highest national burdens of psoriasis in young people in 2019, possibly due to genetic, environmental, and lifestyle factors, as psoriasis has been found to be more common in cold, dry climates (19). Japan, Sweden, and Somalia experienced the largest average annual percent changes in the age-standardized incidence rate of psoriasis among young people from 1990 to 2019, This could be related to economic development, advancements in medical technology, and improvements in prevention methods and treatments for psoriasis in these countries during the specified period (20).

Furthermore, from 1990 to 2019, a clear pattern emerged in the relationship between the burden of psoriasis in young adults and regions’ socio-demographic indexes (SDIs). Firstly, in 2019, the burden in high-SDI regions was significantly exceeded that in low-SDI regions. This can potentially be attributed to young adults in economically developed areas having lifestyles or exposures to environmental and genetic factors that heighten their susceptibility to developing psoriasis. Additionally, a compelling pattern was observed when exploring the relationship between SDIs and the burden of psoriasis in young adults. As the SDI increased, the burden did not follow a linear progression, but manifested in a complex, periodic manner. Specifically, as the SDI surged from 0 to 0.6, the burden showed an upward trend. With further increase to 0.7, the burden decreased, only to increase again as the SDI reached 0.8. It then peaked and subsequently declined. This pattern may signify the combined effect of various factors. For instance, in low-SDI countries, economic development and enhanced living standards may augment young individuals’ exposure to risk factors like an unhealthy diet and sedentary lifestyle, subsequently increasing their burden of psoriasis (17, 20). However, subsequent increases in the SDI could result in improved availability of medical resources and health education, thereby managing the incidence rate and burden of psoriasis in young adults. Consequently, at a certain SDI, the burden of psoriasis in young adults may experience another upsurge as a result of lifestyle changes and environmental pollution (21).

The above-mentioned findings indicate that enhancing economic development alone is insufficient to adequately manage the burden of psoriasis in young adults. Instead, a comprehensive approach must be taken, considering multiple factors including economic, societal, cultural, and healthcare variables. It is imperative to develop targeted strategies and interventions to effectively prevent and treat psoriasis in this specific demographic group.

Generalized additive models (GAMs) were utilized to analyze data on the burden of psoriasis in young adults from 1990 to 2019. The analysis revealed important trends and allowed us to predict the burden for the period of 2020 to 2030. It was observed that although the incidence rate and disability-adjusted life years (DALY) rate of psoriasis declined across all age groups from 1990 to 2019. However, significant variations in the incidence and DALY numbers were found between different age groups. Specifically, the highest incidence and DALY numbers occurred later in the 1990 to 2019 period for individuals aged 15-29, compared with those aged 30-49. Although the overall burden of psoriasis in young adults decreased, it remained high in certain age groups. The predictive analysis indicates that the incidence rate and DALY rate of psoriasis will continue to decline in young adults of all ages from 2020 to 2030, implying an overall improvement in global health. However, it is predicted that the incidence and DALY numbers of psoriasis will increase among individuals aged 30-39. This observed trend may be linked to factors such as lifestyle, genetic, and environmental. For instance, individuals in this age group may adopt unhealthy habits due to work, family, and other social pressures (22), thereby increasing susceptibility to psoriasis. Furthermore, this particular age group may have had prior exposure to risk factors for psoriasis, such as certain medications or infections (23).

This comprehensive analysis of the global burden of psoriasis in young adults has revealed a general downward trend from 1990 to 2019. However, despite this positive trend, there are still key risk factors and challenges that need to be addressed. In order to effectively tackle these challenges, we propose the following points for the global community to consider. Firstly, there is a need to improve public education and awareness about psoriasis, including its genetic, immune responses, hormonal and infection associations (24). Additional attention should be given to informing the public about how to avoid triggers for psoriasis, such as skin damage, certain medications, and excessive alcohol consumption. Secondly, individuals should take precautions to prevent skin injuries, such as cuts, sunburn, or friction, as these can trigger or worsen psoriasis (25, 26). Similarly, maintaining a healthy diet is important, as the excessive consumption of certain foods, such as red meat, dairy products, and processed foods, may exacerbate psoriasis symptoms. It is recommended that individuals adopt a balanced diet by increasing their intake of foods high in omega-3 fatty acids (27, 28), such as fish and nuts, while reducing excessive alcohol consumption and smoking, as these can also worsen psoriasis symptoms. Thirdly, individuals should avoid, if possible, the use of medications known to induce or worsen psoriasis, such as lithium, antimalarial drugs, and certain hypertensive medications (29). Fourthly, governments should prioritize the provision of social and mental health care, including psychological support for patients, as stress and emotional problems can trigger or worsen psoriasis. Lastly, from a policy and regulatory standpoint, governments worldwide should encourage the development of new treatment methods and drugs, broaden treatment options for patients, provide economic support, and increase access to effective treatment for a larger number of patients. Countries should also strengthen collaboration and engage in joint studies focused on the causes of psoriasis, risk factors, and optimal treatment methods.

In this study, we comprehensively evaluated the burden of psoriasis in young adults worldwide based on long-term data. We also analyzed how this burden varied with SDI, geography, and gender. In addition, we utilized GAMs to predict the burden for 2020-2030, thereby providing valuable reference information for public health decision-makers. We also provided targeted strategic recommendations on how countries and regions with different SDIs can reduced their burdens.

This study presents a comprehensive evaluation of the global burden of psoriasis in young adults, using long-term data. We examine the variations in this burden across different Socio-demographic Index (SDI) levels, geographical regions and gender. Additionally, we employ Generalized Additive Models (GAMs) to predict the burden for the period 2020-2030. These predictions serve as valuable reference information for public health decision-makers. Furthermore, we offer targeted strategic recommendations for reducing the burden of psoriasis in countries and regions with varying SDIs.

However, this study also had some limitations. First, although we used a wide range of data sources, there may be some biases and inaccuracies in these data, which might have affected our results. Second, although our prediction model considered multiple factors, it might have failed to account for future events or new risk factors that may arise. In addition, although we conducted an in-depth analysis of multiple factors, there may be other important factors related to psoriasis that we failed to consider. Finally, although we provided a series of strategic recommendations, they may require further on-site verification and implementation to ensure their effectiveness and applicability.

However, this study has several limitations. Firstly, the data used in this study were obtained from a variety of sources, which may introduce biases and inaccuracies that could potentially influence our findings. Secondly, although our prediction model incorporated multiple factors, it may not have accounted for future events or new risk factors that may emerge. Moreover, despite conducting a thorough analysis of multiple factors, there may be other significant variables related to psoriasis that were not taken into consideration. Lastly, while we have offered a set of strategic recommendations, their effectiveness and applicability may need to be further validated and implemented on-site.

## 5 Conclusion

This study revealed a downward trend in the global burden of psoriasis in young adults from 1990 to 2019. However, in 2019, the burden showed an upward trend with increasing age, particularly in the age group of 30-49, where there was a significant increase in the burden. Furthermore, the burden was generally higher men compared to young women, but this pattern was reversed in regions with middle-SDI and low-middle-SDI. Geographically, the incidence rate of psoriasis in young adults was highest in Western Europe, Australia, and High-income North America. Moreover, as the SDI increased, the burden initially decreased and then increased, particularly in countries with very low or very high SDIs. Based on our predictions, we anticipate an increase in the burden of psoriasis will in the age group of 30-39 from 2020 to 2030, emphasizing the need for focused attention on the prevention and treatment of psoriasis in this specific age group.

## Data availability statement

The original contributions presented in the study are included in the article/**Supplementary Material**. Further inquiries can be directed to the corresponding author.

## Ethics statement

The studies involving humans were approved by Global Burden of Disease Study. The studies were conducted in accordance with the local legislation and institutional requirements. The participants provided their written informed consent to participate in this study.

## Author contributions

YZ: Writing – original draft, Writing – review & editing. SD: Data curation, Formal Analysis, Writing – original draft, Writing – review & editing. YuM: Writing – review & editing. YaM: Conceptualization, Methodology, Writing – review & editing.
