# Supplementary material for: Burden of psoriasis in young adults worldwide from the global burden of disease study 2019
Source: Front Endocrinol (Lausanne). 2024 Feb 13;15:1308822. doi: 10.3389/fendo.2024.1308822 (PMC10897041; doi:10.3389/fendo.2024.1308822)
Supplement: Supplementary file 1 [file DataSheet_1.docx]

Supplementary Material

# Supplementary Figures and Tables

For more information on Supplementary Material and for details on the different file types accepted, please see [here](https://www.frontiersin.org/guidelines/author-guidelines#supplementary-material).

## Supplementary Figures


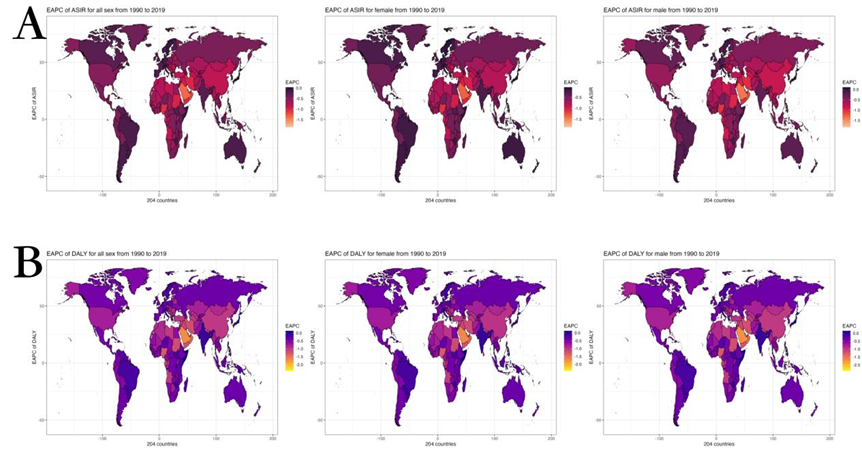


**Supplementary Figure 1.** The EAPC heat map of global psoriasis burden of young adults for both genders in 204 countries from 1990 to 2019. (A) The EAPC of age-standardized incidence rate (B) The EAPC of age-standardized DALY rate.

**
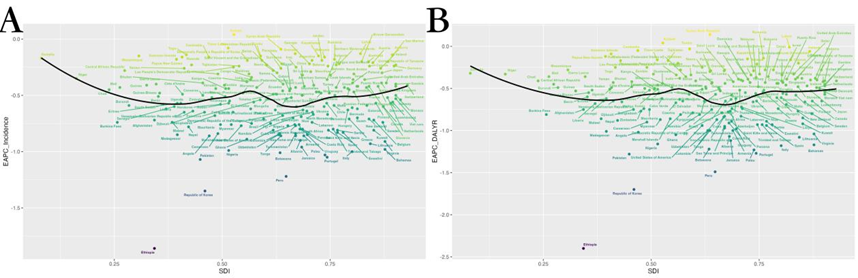
**

**Supplementary Figure 2.** Changes in EAPC of global psoriasis burden of young adults from 1990 to 2019 in different countries with 2019 SDIs. (A) EAPC of age-standardized incidence rate (B) EAPC of age-standardized DALY rate.

**
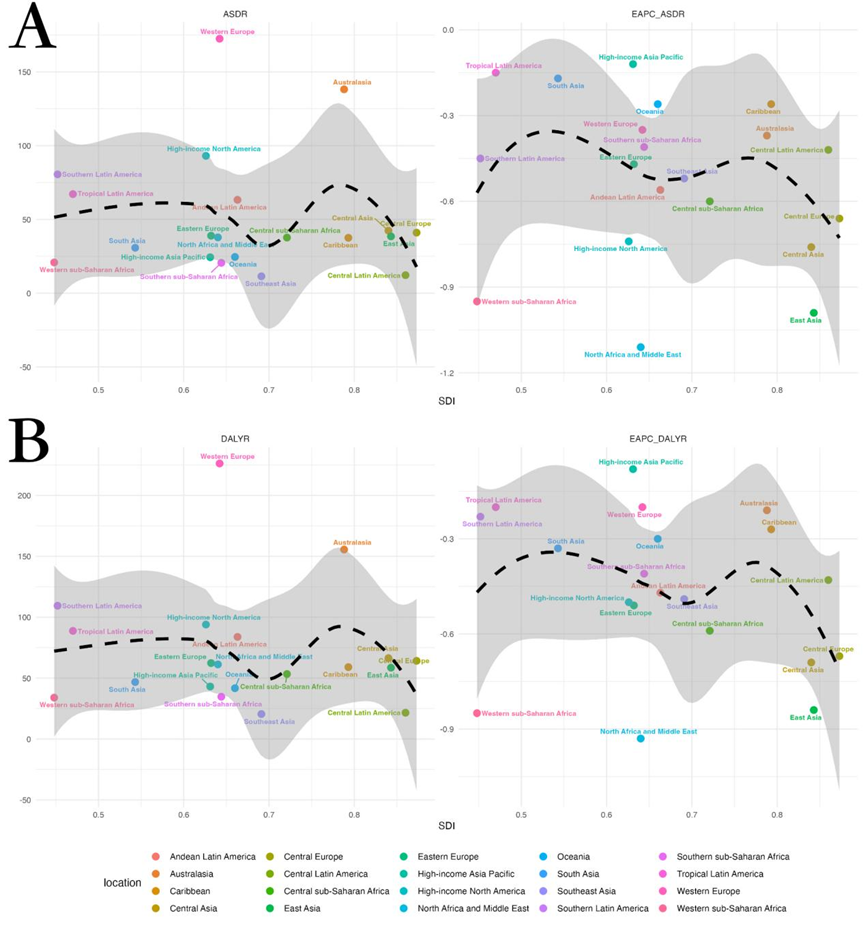
**

**Supplementary Figure 3.** Changes in EAPC of global psoriasis burden of young adults from 1990 to 2019 in different regions with 2019 SDIs. (A) age-standardized incidence rate and its EAPC (B) age-standardized DALY rate and its EAPC.

**
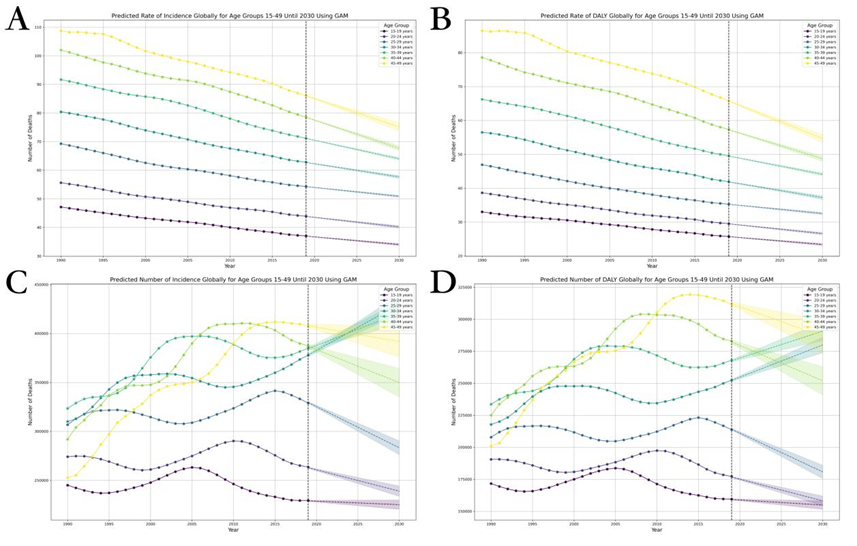
**

**Supplementary Figure 4.** Trends in global psoriasis burden of young adults from 1990 to 2019 and projected changes in global burden from 2019 to 2030 using GAMs modeling, by age group. (A)Rate of incidence (B)Rate of DALY (C)Number of incidence (D)Number of D

## Supplementary tables

**Supplementary Table 1. The incidence of global psoriasis burden of young adults in 1990 and 2019, and its temporal trends from 1990 to 2019.**

|  |  | Incidence case No.(95%UI) |  |  | Age-standardized Incidence rate (per 100000) No.(95% UI) |  |  |
| --- | --- | --- | --- | --- | --- | --- | --- |
| nation | sex | 1990 | 2019 | 1990-2019 EAPC  No.(95%CI) | 1990 | 2019 | 1990-2019 EAPC No.(95%CI) |
| Afghanistan | both | 4216.33(3958.00,4488.31) | 13509.85(12737.74,14369.11) | 3.54 | 95.53(92.48,98.66) | 78.69(77.31,80.09) | -0.72(-0.79,-0.65) |
| Albania | both | 1340.69(1267.67,1416.50) | 839.70(795.36,887.22) | -1.86 | 81.07(76.71,85.65) | 64.75(60.43,69.32) | -0.90(-0.96,-0.83) |
| Algeria | both | 8303.54(7786.79,8845.85) | 13717.96(12953.96,14561.01) | 1.78 | 75.19(73.50,76.92) | 60.10(59.10,61.12) | -0.77(-0.80,-0.74) |
| American Samoa | both | 8.73(8.18,9.30) | 8.96(8.41,9.52) | -0.15 | 38.33(16.90,77.14) | 33.78(15.20,66.52) | -0.41(-0.44,-0.38) |
| Andorra | both | 78.05(73.81,82.11) | 99.65(93.94,104.98) | 0.54 | 236.50(186.36,297.43) | 227.81(182.46,282.76) | -0.14(-0.14,-0.13) |
| Angola | both | 3059.57(2847.66,3289.55) | 6889.92(6410.06,7371.34) | 2.87 | 68.36(65.88,70.91) | 53.13(51.85,54.44) | -0.86(-0.92,-0.81) |
| Antigua and Barbuda | both | 17.57(16.50,18.68) | 25.39(23.86,26.95) | 1.30 | 57.67(33.63,94.03) | 51.93(33.64,77.16) | -0.36(-0.37,-0.35) |
| Argentina | both | 18541.89(17316.91,19887.26) | 25794.01(24152.70,27604.28) | 1.18 | 117.37(115.68,119.07) | 110.23(108.89,111.59) | -0.19(-0.21,-0.18) |
| Armenia | both | 1307.21(1221.39,1390.93) | 949.32(888.75,1009.04) | -1.33 | 78.83(74.49,83.40) | 62.89(58.88,67.15) | -0.86(-0.92,-0.80) |
| Australia | both | 14942.22(13988.96,15956.71) | 18120.68(16950.94,19384.76) | 0.67 | 165.02(162.38,167.69) | 152.90(150.65,155.17) | -0.20(-0.26,-0.15) |
| Austria | both | 10389.03(9867.59,10937.18) | 10069.95(9524.96,10587.99) | -0.07 | 254.45(249.55,259.43) | 239.72(234.95,244.57) | -0.21(-0.22,-0.20) |
| Azerbaijan | both | 2642.61(2473.29,2818.18) | 3580.34(3348.21,3818.50) | 1.07 | 75.74(72.73,78.87) | 63.68(61.58,65.83) | -0.69(-0.78,-0.60) |
| Bahamas | both | 76.48(71.79,81.65) | 107.76(100.87,114.38) | 1.15 | 55.82(43.80,70.44) | 51.67(42.34,62.52) | -0.32(-0.34,-0.29) |
| Bahrain | both | 202.52(189.31,216.12) | 548.29(512.38,583.88) | 4.45 | 67.02(57.58,77.90) | 55.28(50.30,60.71) | -0.68(-0.71,-0.66) |
| Bangladesh | both | 23728.60(22442.36,25186.38) | 35357.84(33375.72,37337.37) | 1.41 | 49.88(49.22,50.54) | 41.59(41.15,42.02) | -0.57(-0.60,-0.54) |
| Barbados | both | 72.23(67.96,77.30) | 73.58(69.20,78.16) | 0.07 | 54.50(42.56,69.01) | 50.69(39.64,64.04) | -0.24(-0.25,-0.23) |
| Belarus | both | 3934.57(3710.29,4140.27) | 2936.66(2768.86,3104.14) | -1.18 | 77.75(75.32,80.23) | 63.92(61.53,66.39) | -0.72(-0.77,-0.67) |
| Belgium | both | 13061.51(12367.40,13766.14) | 12593.96(11893.31,13310.05) | -0.14 | 259.25(254.80,263.76) | 242.71(238.42,247.07) | -0.23(-0.23,-0.22) |
| Belize | both | 54.24(50.77,57.92) | 127.41(119.17,135.60) | 3.10 | 68.42(50.59,91.48) | 58.37(48.61,69.58) | -0.51(-0.54,-0.47) |
| Benin | both | 739.07(696.99,783.98) | 1802.48(1702.12,1911.48) | 3.09 | 38.84(35.99,41.89) | 32.62(31.08,34.22) | -0.62(-0.67,-0.58) |
| Bermuda | both | 20.34(19.02,21.57) | 15.72(14.71,16.68) | -0.94 | 58.67(35.76,92.72) | 52.88(29.12,91.18) | -0.39(-0.41,-0.38) |
| Bhutan | both | 146.16(138.18,155.04) | 186.72(176.80,197.49) | 1.30 | 51.76(43.30,61.60) | 43.46(37.40,50.29) | -0.62(-0.63,-0.61) |
| Bolivia (Plurinational State of) | both | 3098.77(2896.58,3318.98) | 5621.23(5250.99,6036.06) | 2.18 | 108.88(105.00,112.87) | 93.60(91.16,96.09) | -0.47(-0.51,-0.42) |
| Bosnia and Herzegovina | both | 1978.71(1865.96,2087.89) | 1009.79(954.89,1068.21) | -2.15 | 82.44(78.83,86.17) | 63.95(59.98,68.14) | -1.01(-1.07,-0.95) |
| Botswana | both | 229.25(215.06,244.45) | 441.19(413.71,470.62) | 2.13 | 40.37(35.08,46.34) | 33.52(30.45,36.84) | -0.68(-0.73,-0.63) |
| Brazil | both | 69771.56(66149.52,73559.10) | 104318.71(98996.54,109453.70) | 1.42 | 94.43(93.72,95.15) | 88.68(88.14,89.22) | -0.20(-0.21,-0.19) |
| Brunei Darussalam | both | 57.63(54.40,61.26) | 102.31(96.76,107.88) | 1.94 | 40.43(30.36,53.37) | 37.69(30.71,45.90) | -0.23(-0.25,-0.21) |
| Bulgaria | both | 3246.97(3068.31,3428.54) | 2004.10(1888.08,2119.39) | -1.71 | 76.27(73.65,78.97) | 61.97(59.13,64.92) | -0.74(-0.80,-0.68) |
| Burkina Faso | both | 1519.01(1436.16,1606.47) | 3483.18(3290.02,3691.03) | 2.94 | 41.64(39.51,43.86) | 35.68(34.47,36.92) | -0.59(-0.63,-0.55) |
| Burundi | both | 657.70(618.50,702.77) | 1425.36(1342.66,1514.09) | 2.99 | 28.52(26.29,30.91) | 27.24(25.80,28.75) | -0.16(-0.19,-0.12) |
| Cabo Verde | both | 51.15(48.21,54.43) | 91.42(86.52,96.75) | 2.03 | 37.69(27.19,51.66) | 30.05(24.16,37.04) | -0.84(-0.90,-0.79) |
| Cambodia | both | 1387.57(1305.17,1479.89) | 2341.90(2197.90,2487.01) | 1.85 | 32.51(30.76,34.34) | 27.39(26.28,28.54) | -0.60(-0.62,-0.59) |
| Cameroon | both | 1514.98(1432.60,1605.30) | 4022.09(3795.62,4280.67) | 3.46 | 35.26(33.45,37.15) | 29.35(28.42,30.31) | -0.65(-0.69,-0.60) |
| Canada | both | 14796.52(13987.12,15615.21) | 15166.18(14332.39,16009.93) | -0.03 | 97.47(95.90,99.07) | 90.38(88.93,91.85) | -0.28(-0.30,-0.27) |
| Central African Republic | both | 812.78(755.44,869.81) | 1466.47(1369.90,1570.40) | 2.02 | 68.04(63.29,73.08) | 59.71(56.63,62.93) | -0.45(-0.47,-0.44) |
| Chad | both | 1012.54(955.17,1075.53) | 2348.16(2215.32,2486.40) | 2.87 | 42.87(40.18,45.71) | 36.59(35.06,38.17) | -0.59(-0.63,-0.54) |
| Chile | both | 8205.60(7652.60,8775.05) | 10094.90(9446.85,10765.87) | 0.72 | 117.86(115.30,120.46) | 107.46(105.36,109.59) | -0.32(-0.34,-0.31) |
| China | both | 476366.43(450415.86,501938.78) | 451290.33(427463.75,474173.14) | -0.06 | 74.88(74.66,75.10) | 58.37(58.20,58.55) | -0.84(-0.86,-0.83) |
| Colombia | both | 3723.28(3508.77,3947.61) | 4974.01(4687.43,5277.32) | 0.92 | 23.03(22.28,23.80) | 19.98(19.43,20.55) | -0.50(-0.52,-0.48) |
| Comoros | both | 53.91(50.58,57.27) | 91.95(86.64,97.22) | 1.88 | 27.95(20.72,37.13) | 25.10(20.21,30.86) | -0.34(-0.36,-0.33) |
| Congo | both | 590.85(551.77,634.22) | 1186.60(1109.43,1269.50) | 2.44 | 56.68(51.98,61.74) | 44.96(42.43,47.60) | -0.80(-0.84,-0.76) |
| Cook Islands | both | 3.42(3.22,3.64) | 2.74(2.57,2.91) | -0.66 | 38.14(8.70,114.45) | 32.90(6.00,105.09) | -0.49(-0.50,-0.47) |
| Costa Rica | both | 334.39(314.56,354.44) | 498.26(469.74,528.45) | 1.33 | 22.65(20.23,25.31) | 19.82(18.12,21.65) | -0.45(-0.47,-0.42) |
| Croatia | both | 1857.42(1754.92,1959.98) | 1192.52(1123.52,1256.18) | -1.67 | 74.81(71.42,78.32) | 61.32(57.79,65.03) | -0.76(-0.82,-0.71) |
| Cuba | both | 3617.61(3395.12,3850.61) | 3134.79(2934.27,3327.87) | -0.39 | 60.57(58.60,62.60) | 56.46(54.45,58.53) | -0.24(-0.26,-0.22) |
| Cyprus | both | 1030.34(976.52,1083.61) | 1637.88(1549.01,1730.44) | 1.73 | 253.86(238.58,269.90) | 227.46(216.20,239.27) | -0.38(-0.42,-0.35) |
| Czechia | both | 4059.05(3832.63,4274.84) | 3143.70(2951.37,3321.56) | -0.94 | 75.78(73.44,78.18) | 61.05(58.79,63.38) | -0.75(-0.80,-0.70) |
| Côte d'Ivoire | both | 1955.55(1846.03,2069.63) | 3944.14(3728.80,4183.60) | 2.35 | 37.02(35.33,38.78) | 31.20(30.22,32.21) | -0.57(-0.63,-0.52) |
| Democratic People's Republic of Korea | both | 8940.17(8403.97,9486.22) | 10256.60(9629.94,10847.61) | 0.66 | 85.75(83.97,87.56) | 71.10(69.71,72.50) | -0.65(-0.67,-0.63) |
| Democratic Republic of the Congo | both | 10067.07(9363.60,10817.42) | 21554.43(20121.82,23115.86) | 2.74 | 63.38(62.09,64.69) | 54.09(53.35,54.85) | -0.48(-0.59,-0.36) |
| Denmark | both | 6967.86(6594.58,7334.91) | 6362.32(6035.39,6693.29) | -0.37 | 255.16(249.15,261.28) | 241.95(235.96,248.06) | -0.19(-0.20,-0.18) |
| Djibouti | both | 61.60(57.70,65.63) | 158.48(149.66,167.85) | 3.67 | 28.05(21.15,36.85) | 24.76(21.04,28.97) | -0.46(-0.48,-0.44) |
| Dominica | both | 19.77(18.56,21.02) | 17.79(16.72,18.93) | -0.33 | 58.93(35.43,93.35) | 52.22(30.81,83.17) | -0.44(-0.47,-0.40) |
| Dominican Republic | both | 2319.71(2165.58,2476.45) | 3369.04(3157.16,3594.67) | 1.19 | 67.66(64.83,70.58) | 58.90(56.93,60.93) | -0.56(-0.60,-0.51) |
| Ecuador | both | 4264.39(3970.79,4569.70) | 7079.97(6608.63,7580.62) | 1.73 | 90.77(88.00,93.62) | 78.25(76.43,80.10) | -0.50(-0.51,-0.49) |
| Egypt | both | 12320.65(11592.03,13068.71) | 19422.48(18422.69,20503.66) | 1.81 | 47.77(46.91,48.63) | 38.35(37.81,38.89) | -0.57(-0.62,-0.51) |
| El Salvador | both | 612.00(576.50,650.86) | 712.68(670.72,757.57) | 0.46 | 26.07(23.99,28.31) | 22.20(20.59,23.91) | -0.59(-0.62,-0.56) |
| Equatorial Guinea | both | 117.14(109.05,125.60) | 295.08(274.41,318.26) | 3.17 | 68.27(56.13,82.55) | 42.08(37.11,47.64) | -1.86(-1.93,-1.79) |
| Eritrea | both | 395.13(370.06,421.85) | 883.43(831.83,941.78) | 2.95 | 30.62(27.56,33.96) | 26.68(24.91,28.56) | -0.43(-0.48,-0.39) |
| Estonia | both | 580.01(547.51,612.96) | 379.62(357.68,401.33) | -1.45 | 75.19(69.17,81.62) | 61.92(55.62,68.85) | -0.70(-0.74,-0.66) |
| Eswatini | both | 142.52(133.52,152.28) | 211.23(197.59,225.01) | 1.16 | 42.54(35.55,50.65) | 36.12(31.31,41.55) | -0.57(-0.60,-0.54) |
| Ethiopia | both | 6284.94(5934.38,6641.17) | 13082.87(12383.87,13881.82) | 2.45 | 29.93(29.17,30.71) | 26.30(25.83,26.77) | -0.43(-0.47,-0.40) |
| Fiji | both | 157.68(147.96,168.49) | 169.31(158.87,179.88) | 0.30 | 41.85(35.50,49.09) | 36.26(31.00,42.16) | -0.46(-0.49,-0.44) |
| Finland | both | 7321.55(6909.50,7695.54) | 6199.18(5875.65,6521.94) | -0.67 | 274.04(267.67,280.53) | 257.78(251.32,264.37) | -0.22(-0.22,-0.21) |
| France | both | 87228.77(82304.53,92574.92) | 81102.62(76299.22,85798.26) | -0.33 | 296.45(294.48,298.43) | 277.24(275.31,279.18) | -0.24(-0.26,-0.22) |
| Gabon | both | 233.17(215.83,250.25) | 379.63(354.74,406.23) | 1.70 | 55.12(47.97,63.20) | 42.09(37.93,46.61) | -0.89(-0.91,-0.88) |
| Gambia | both | 166.66(157.21,177.07) | 345.94(326.79,367.48) | 2.35 | 39.70(33.65,46.69) | 32.76(29.27,36.60) | -0.71(-0.76,-0.66) |
| Georgia | both | 1943.91(1817.96,2068.43) | 1088.20(1018.14,1156.33) | -2.13 | 73.13(69.90,76.48) | 63.59(59.80,67.58) | -0.50(-0.57,-0.43) |
| Germany | both | 85659.35(81256.16,90117.76) | 75796.99(71740.49,79896.68) | -0.53 | 209.72(208.30,211.15) | 203.36(201.89,204.85) | -0.13(-0.17,-0.10) |
| Ghana | both | 2710.77(2518.08,2921.25) | 5559.05(5174.88,5975.97) | 2.47 | 41.55(39.95,43.20) | 34.56(33.64,35.50) | -0.70(-0.73,-0.66) |
| Greece | both | 13083.19(12364.71,13785.09) | 11271.73(10623.58,11867.76) | -0.54 | 256.74(252.35,261.18) | 239.02(234.42,243.70) | -0.25(-0.27,-0.22) |
| Greenland | both | 40.22(38.07,42.47) | 28.73(27.18,30.36) | -1.06 | 116.53(82.78,161.66) | 105.54(70.37,153.50) | -0.41(-0.43,-0.38) |
| Grenada | both | 23.41(22.01,25.05) | 29.19(27.36,31.08) | 0.83 | 63.37(39.58,98.17) | 53.98(36.10,78.13) | -0.48(-0.54,-0.41) |
| Guam | both | 26.03(24.46,27.78) | 25.01(23.50,26.52) | -0.30 | 35.10(22.73,52.68) | 30.79(19.88,45.74) | -0.49(-0.53,-0.46) |
| Guatemala | both | 931.66(877.48,995.68) | 2153.06(2026.25,2294.66) | 3.05 | 28.42(26.57,30.37) | 23.52(22.52,24.56) | -0.64(-0.67,-0.61) |
| Guinea | both | 1013.64(957.10,1073.99) | 1857.54(1754.84,1970.58) | 1.94 | 40.12(37.64,42.73) | 34.17(32.58,35.82) | -0.58(-0.63,-0.53) |
| Guinea-Bissau | both | 168.03(158.53,178.44) | 304.11(286.62,323.38) | 2.06 | 40.20(34.13,47.18) | 33.76(29.93,38.00) | -0.63(-0.68,-0.59) |
| Guyana | both | 229.99(214.94,245.23) | 218.29(205.17,231.95) | -0.34 | 60.40(52.60,69.18) | 53.77(46.83,61.50) | -0.39(-0.40,-0.37) |
| Haiti | both | 2095.60(1958.30,2239.22) | 4310.28(4030.76,4591.49) | 2.55 | 74.70(71.48,78.05) | 67.41(65.39,69.47) | -0.36(-0.37,-0.35) |
| Honduras | both | 537.20(505.40,571.66) | 1196.77(1124.56,1273.65) | 2.81 | 27.96(25.57,30.54) | 24.13(22.76,25.57) | -0.52(-0.56,-0.48) |
| Hungary | both | 4007.11(3771.81,4220.51) | 2913.38(2741.90,3067.97) | -1.23 | 76.57(74.19,79.02) | 61.85(59.52,64.27) | -0.77(-0.82,-0.72) |
| Iceland | both | 360.68(340.94,380.03) | 424.97(402.54,448.64) | 0.52 | 272.92(245.40,302.83) | 254.83(231.01,280.62) | -0.25(-0.26,-0.25) |
| India | both | 209681.04(199086.57,221600.00) | 360448.47(342640.97,380250.68) | 1.92 | 51.52(51.30,51.74) | 48.00(47.85,48.16) | -0.26(-0.31,-0.20) |
| Indonesia | both | 13328.17(12606.20,14102.23) | 19436.59(18303.52,20565.01) | 1.33 | 14.98(14.72,15.24) | 13.36(13.17,13.55) | -0.36(-0.39,-0.33) |
| Iran (Islamic Republic of) | both | 19728.57(18741.98,20796.75) | 29565.33(28034.30,31148.67) | 1.59 | 79.30(78.15,80.47) | 60.35(59.65,61.06) | -1.00(-1.06,-0.95) |
| Iraq | both | 6496.43(6093.94,6923.18) | 15170.65(14313.76,16097.53) | 2.71 | 88.02(85.82,90.27) | 67.53(66.44,68.63) | -0.96(-0.99,-0.92) |
| Ireland | both | 4677.86(4442.82,4922.16) | 5857.50(5538.09,6184.23) | 1.00 | 265.09(257.53,272.82) | 242.33(236.00,248.79) | -0.30(-0.32,-0.29) |
| Israel | both | 5791.09(5490.11,6111.02) | 10097.15(9565.74,10630.48) | 1.81 | 242.23(235.98,248.61) | 228.98(224.52,233.50) | -0.19(-0.20,-0.17) |
| Italy | both | 57909.44(55021.36,60829.57) | 49866.52(46940.19,52650.90) | -0.25 | 199.97(198.34,201.61) | 186.52(184.80,188.24) | -0.15(-0.22,-0.08) |
| Jamaica | both | 681.18(638.18,726.83) | 830.31(774.73,885.82) | 0.67 | 62.02(57.29,67.07) | 54.66(51.00,58.53) | -0.44(-0.46,-0.42) |
| Japan | both | 29143.30(27619.72,30613.40) | 24061.44(22768.50,25304.66) | -0.60 | 43.66(43.15,44.17) | 43.55(42.97,44.13) | 0.04(0.03,0.05) |
| Jordan | both | 1180.52(1106.72,1258.63) | 3567.98(3363.35,3773.55) | 3.90 | 71.73(67.42,76.27) | 57.34(55.47,59.26) | -0.81(-0.83,-0.79) |
| Kazakhstan | both | 6343.24(5928.40,6756.53) | 6119.91(5750.77,6490.89) | -0.03 | 79.04(77.07,81.05) | 64.81(63.18,66.48) | -0.71(-0.75,-0.67) |
| Kenya | both | 2398.24(2269.14,2536.26) | 5706.86(5404.13,6038.52) | 3.02 | 25.32(24.26,26.43) | 22.83(22.23,23.45) | -0.31(-0.34,-0.28) |
| Kiribati | both | 14.38(13.54,15.29) | 22.85(21.50,24.19) | 1.88 | 41.85(22.72,72.21) | 39.17(24.68,59.72) | -0.13(-0.16,-0.09) |
| Kuwait | both | 650.55(608.65,693.39) | 1599.06(1507.03,1693.92) | 3.63 | 61.79(56.97,66.96) | 51.67(49.01,54.48) | -0.74(-0.78,-0.70) |
| Kyrgyzstan | both | 1623.68(1514.68,1738.50) | 2312.91(2170.56,2458.82) | 1.26 | 81.19(77.10,85.47) | 69.85(67.02,72.78) | -0.51(-0.55,-0.47) |
| Lao People's Democratic Republic | both | 584.11(548.47,621.49) | 1056.52(992.55,1121.25) | 2.10 | 33.51(30.79,36.44) | 28.06(26.38,29.83) | -0.62(-0.65,-0.60) |
| Latvia | both | 971.17(919.44,1021.85) | 534.71(503.85,562.33) | -2.04 | 74.32(69.70,79.19) | 61.77(56.44,67.54) | -0.65(-0.70,-0.61) |
| Lebanon | both | 1096.30(1032.38,1170.71) | 1597.90(1505.37,1692.65) | 1.43 | 75.26(70.82,79.91) | 58.44(55.58,61.43) | -0.93(-0.95,-0.90) |
| Lesotho | both | 363.33(340.18,388.91) | 437.86(409.56,468.80) | 0.46 | 46.21(41.44,51.43) | 39.28(35.62,43.26) | -0.56(-0.59,-0.52) |
| Liberia | both | 297.43(280.84,315.54) | 772.06(731.22,816.60) | 3.38 | 37.63(33.41,42.28) | 32.22(29.96,34.61) | -0.67(-0.72,-0.63) |
| Libya | both | 1300.21(1219.83,1384.59) | 2337.67(2196.83,2479.15) | 2.36 | 71.52(67.54,75.70) | 55.75(53.50,58.07) | -0.85(-0.93,-0.77) |
| Lithuania | both | 1362.47(1288.69,1433.24) | 764.07(720.90,805.45) | -2.07 | 74.12(70.22,78.17) | 60.58(56.26,65.19) | -0.74(-0.78,-0.70) |
| Luxembourg | both | 501.69(473.44,527.16) | 753.97(712.45,794.55) | 1.36 | 245.48(224.18,268.53) | 235.28(218.35,253.36) | -0.16(-0.17,-0.16) |
| Madagascar | both | 1486.97(1398.03,1580.77) | 3436.13(3240.98,3647.06) | 2.93 | 29.69(28.13,31.32) | 27.13(26.21,28.08) | -0.31(-0.33,-0.28) |
| Malawi | both | 1229.41(1154.52,1309.19) | 2299.21(2166.87,2455.26) | 2.14 | 30.41(28.67,32.25) | 27.03(25.89,28.22) | -0.43(-0.46,-0.39) |
| Malaysia | both | 2086.40(1969.11,2207.71) | 3508.07(3299.13,3712.07) | 1.71 | 24.29(23.24,25.38) | 20.32(19.65,21.01) | -0.61(-0.64,-0.58) |
| Maldives | both | 25.54(24.06,27.23) | 77.05(72.22,82.00) | 3.94 | 29.58(18.74,45.13) | 23.58(18.41,30.04) | -0.77(-0.82,-0.73) |
| Mali | both | 1279.27(1188.91,1376.20) | 2748.97(2543.93,2972.69) | 2.68 | 36.95(34.91,39.08) | 30.35(29.18,31.56) | -0.76(-0.79,-0.73) |
| Malta | both | 502.25(473.91,529.60) | 482.97(456.93,509.39) | -0.22 | 256.13(233.96,279.98) | 234.83(213.80,257.71) | -0.29(-0.31,-0.28) |
| Marshall Islands | both | 8.24(7.72,8.79) | 11.35(10.64,12.05) | 0.98 | 44.70(18.65,97.59) | 37.97(19.16,68.20) | -0.53(-0.55,-0.51) |
| Mauritania | both | 328.81(310.15,348.12) | 584.21(550.13,619.03) | 1.91 | 37.92(33.80,42.45) | 31.63(29.05,34.39) | -0.64(-0.69,-0.59) |
| Mauritius | both | 159.54(150.07,169.22) | 156.30(146.56,166.48) | -0.19 | 27.13(23.02,31.84) | 23.26(19.73,27.27) | -0.53(-0.54,-0.52) |
| Mexico | both | 10023.73(9511.31,10601.55) | 14579.59(13816.31,15395.64) | 1.28 | 25.07(24.57,25.59) | 21.94(21.58,22.30) | -0.44(-0.47,-0.41) |
| Micronesia (Federated States of) | both | 18.49(17.35,19.68) | 18.84(17.63,20.02) | -0.10 | 42.26(24.72,69.33) | 36.57(21.87,57.75) | -0.48(-0.49,-0.46) |
| Monaco | both | 34.22(32.38,36.04) | 33.88(32.00,35.66) | 0.10 | 236.27(161.21,342.12) | 226.89(154.60,324.11) | -0.15(-0.15,-0.14) |
| Mongolia | both | 829.63(774.03,888.00) | 1271.22(1191.22,1348.32) | 1.64 | 85.53(79.50,91.97) | 69.04(65.25,73.02) | -0.76(-0.80,-0.72) |
| Montenegro | both | 230.77(218.67,243.53) | 184.80(174.60,194.88) | -0.98 | 73.01(63.87,83.14) | 60.60(52.08,70.21) | -0.75(-0.83,-0.68) |
| Morocco | both | 9926.42(9324.32,10590.34) | 12850.82(12109.61,13592.69) | 0.85 | 83.41(81.73,85.11) | 66.81(65.65,67.97) | -0.78(-0.80,-0.77) |
| Mozambique | both | 1784.42(1678.49,1902.98) | 3613.44(3424.96,3843.01) | 2.32 | 33.14(31.59,34.74) | 28.64(27.68,29.64) | -0.52(-0.55,-0.49) |
| Myanmar | both | 6237.16(5871.15,6638.32) | 7870.31(7395.43,8350.57) | 0.70 | 32.95(32.11,33.80) | 27.27(26.67,27.88) | -0.71(-0.73,-0.68) |
| Namibia | both | 247.84(231.69,265.02) | 417.31(390.84,445.16) | 1.71 | 39.91(34.89,45.53) | 34.15(30.91,37.65) | -0.55(-0.57,-0.52) |
| Nauru | both | 1.78(1.67,1.89) | 1.79(1.68,1.90) | -0.08 | 37.46(3.59,161.00) | 33.28(3.23,141.15) | -0.35(-0.43,-0.27) |
| Nepal | both | 4120.73(3904.05,4356.37) | 5822.19(5506.12,6155.52) | 1.11 | 48.32(46.83,49.85) | 37.26(36.29,38.24) | -0.99(-1.03,-0.95) |
| Netherlands | both | 20771.80(19662.64,21909.65) | 18475.52(17479.08,19475.39) | -0.48 | 252.67(249.23,256.14) | 239.15(235.66,242.68) | -0.19(-0.20,-0.18) |
| New Zealand | both | 3295.91(3127.72,3472.37) | 3456.73(3274.84,3642.69) | 0.20 | 182.52(176.33,188.87) | 170.99(165.27,176.85) | -0.22(-0.23,-0.21) |
| Nicaragua | both | 435.28(410.93,464.49) | 792.36(744.61,841.79) | 2.05 | 27.17(24.56,30.02) | 23.11(21.52,24.79) | -0.54(-0.57,-0.52) |
| Niger | both | 1399.17(1318.29,1481.48) | 3435.89(3241.97,3658.14) | 3.03 | 43.91(41.58,46.36) | 38.94(37.58,40.35) | -0.43(-0.48,-0.39) |
| Nigeria | both | 18235.66(17298.98,19252.09) | 33457.95(31681.83,35374.66) | 2.03 | 46.54(45.85,47.24) | 34.85(34.47,35.24) | -1.07(-1.15,-0.99) |
| Niue | both | 0.40(0.37,0.42) | 0.27(0.25,0.28) | -1.69 | 39.76(0.01,485.84) | 34.20(0.00,622.62) | -0.54(-0.55,-0.53) |
| North Macedonia | both | 801.76(758.89,845.59) | 700.55(661.97,738.31) | -0.52 | 76.89(71.66,82.42) | 62.44(57.81,67.40) | -0.80(-0.86,-0.75) |
| Northern Mariana Islands | both | 10.22(9.59,10.87) | 6.86(6.42,7.32) | -1.41 | 35.71(17.08,67.67) | 33.13(12.09,79.62) | -0.18(-0.22,-0.14) |
| Norway | both | 4224.57(4010.87,4436.14) | 4680.49(4441.93,4910.93) | 0.33 | 192.48(186.70,198.41) | 182.05(176.80,187.43) | -0.22(-0.23,-0.21) |
| Oman | both | 698.15(652.81,742.27) | 1672.78(1569.44,1780.95) | 3.33 | 73.88(68.36,79.79) | 51.83(49.20,54.59) | -1.22(-1.30,-1.14) |
| Pakistan | both | 24243.80(22991.14,25627.45) | 46496.83(43978.08,49099.16) | 2.24 | 51.62(50.95,52.29) | 43.45(43.05,43.85) | -0.63(-0.64,-0.61) |
| Palau | both | 2.98(2.80,3.17) | 3.24(3.03,3.45) | -0.18 | 36.16(7.23,114.74) | 32.06(6.65,102.01) | -0.39(-0.41,-0.38) |
| Palestine | both | 697.99(653.27,743.98) | 1539.98(1451.62,1636.64) | 2.86 | 85.22(78.61,92.30) | 63.20(60.02,66.52) | -1.05(-1.07,-1.03) |
| Panama | both | 261.22(245.90,277.19) | 411.04(387.32,436.78) | 1.55 | 22.18(19.53,25.12) | 19.57(17.72,21.56) | -0.42(-0.43,-0.40) |
| Papua New Guinea | both | 885.65(831.82,943.26) | 2089.48(1963.26,2223.78) | 3.14 | 47.89(44.71,51.25) | 42.71(40.89,44.60) | -0.36(-0.38,-0.34) |
| Paraguay | both | 1752.21(1632.58,1884.38) | 3346.55(3117.89,3584.16) | 2.28 | 96.91(92.34,101.67) | 91.58(88.49,94.76) | -0.18(-0.19,-0.16) |
| Peru | both | 9702.38(9056.46,10402.89) | 14882.61(13869.16,15872.46) | 1.47 | 95.70(93.76,97.67) | 83.37(82.04,84.72) | -0.47(-0.50,-0.44) |
| Philippines | both | 8036.83(7611.35,8475.12) | 14241.60(13485.70,15023.34) | 2.09 | 27.54(26.92,28.16) | 24.97(24.56,25.38) | -0.23(-0.29,-0.17) |
| Poland | both | 15242.02(14405.77,16062.57) | 13326.13(12727.79,13871.84) | -0.65 | 79.25(77.98,80.54) | 69.69(68.46,70.94) | -0.43(-0.46,-0.40) |
| Portugal | both | 13515.72(12791.53,14224.97) | 12174.19(11479.38,12875.95) | -0.39 | 270.41(265.87,275.01) | 247.46(242.91,252.09) | -0.30(-0.31,-0.29) |
| Puerto Rico | both | 1003.67(943.16,1066.76) | 801.84(755.46,852.66) | -0.82 | 54.80(51.46,58.31) | 49.14(45.76,52.71) | -0.40(-0.41,-0.39) |
| Qatar | both | 194.12(181.30,206.63) | 1145.34(1076.88,1223.60) | 7.78 | 64.53(55.28,75.15) | 50.40(47.17,53.91) | -0.86(-0.88,-0.84) |
| Republic of Korea | both | 12117.48(11422.86,12868.55) | 11949.40(11259.05,12665.93) | -0.02 | 47.80(46.94,48.67) | 43.16(42.36,43.98) | -0.31(-0.36,-0.26) |
| Republic of Moldova | both | 1667.72(1575.45,1758.67) | 1233.98(1166.84,1300.31) | -1.22 | 75.75(72.13,79.52) | 64.26(60.61,68.11) | -0.60(-0.67,-0.54) |
| Romania | both | 8455.45(7995.69,8892.76) | 5490.33(5190.34,5806.28) | -1.62 | 74.35(72.77,75.97) | 59.68(58.04,61.36) | -0.81(-0.88,-0.75) |
| Russian Federation | both | 52650.82(49746.03,55571.88) | 44739.28(42502.73,46924.11) | -0.79 | 70.50(69.89,71.11) | 61.91(61.31,62.51) | -0.47(-0.49,-0.45) |
| Rwanda | both | 755.81(693.22,825.71) | 1409.33(1300.79,1524.45) | 2.80 | 25.52(23.63,27.55) | 22.70(21.50,23.96) | -0.40(-0.43,-0.38) |
| Saint Kitts and Nevis | both | 10.95(10.20,11.76) | 16.67(15.65,17.66) | 1.57 | 58.42(27.66,116.17) | 51.52(29.68,84.18) | -0.42(-0.44,-0.40) |
| Saint Lucia | both | 38.56(36.11,41.20) | 51.97(48.61,55.15) | 1.14 | 61.69(43.15,86.50) | 54.18(40.37,71.44) | -0.42(-0.46,-0.38) |
| Saint Vincent and the Grenadines | both | 31.54(29.44,33.69) | 32.27(30.27,34.36) | 0.06 | 63.39(42.27,93.03) | 55.71(38.11,78.86) | -0.42(-0.45,-0.40) |
| Samoa | both | 28.58(26.81,30.47) | 37.87(35.62,40.40) | 0.93 | 40.75(26.59,60.53) | 38.34(26.98,53.07) | -0.16(-0.19,-0.13) |
| San Marino | both | 29.01(27.58,30.50) | 36.05(34.15,38.04) | 0.80 | 241.34(161.50,347.85) | 230.26(159.98,322.99) | -0.17(-0.19,-0.15) |
| Sao Tome and Principe | both | 16.17(15.27,17.16) | 29.41(27.82,31.06) | 2.06 | 34.73(19.18,59.38) | 28.38(18.97,41.17) | -0.76(-0.80,-0.73) |
| Saudi Arabia | both | 6114.69(5729.99,6510.97) | 13197.46(12411.01,13978.22) | 2.85 | 78.62(76.61,80.66) | 52.32(51.41,53.24) | -1.35(-1.43,-1.27) |
| Senegal | both | 1205.47(1135.95,1277.47) | 2322.99(2184.65,2465.04) | 2.20 | 39.13(36.87,41.52) | 33.22(31.84,34.64) | -0.58(-0.63,-0.54) |
| Serbia | both | 3602.90(3410.20,3801.58) | 2575.91(2431.86,2707.27) | -1.54 | 77.25(74.73,79.82) | 62.21(59.79,64.70) | -0.83(-0.89,-0.77) |
| Seychelles | both | 8.40(7.92,8.92) | 12.08(11.33,12.79) | 1.41 | 24.27(10.40,49.95) | 21.26(10.90,38.37) | -0.42(-0.46,-0.38) |
| Sierra Leone | both | 615.08(580.20,652.67) | 1316.76(1242.31,1395.68) | 3.08 | 38.93(35.85,42.23) | 33.31(31.48,35.24) | -0.57(-0.61,-0.54) |
| Singapore | both | 738.52(696.98,781.44) | 1186.32(1119.09,1253.96) | 1.81 | 38.87(36.09,41.80) | 35.66(33.51,37.94) | -0.26(-0.29,-0.24) |
| Slovakia | both | 2082.63(1974.70,2191.03) | 1700.74(1605.73,1793.46) | -0.86 | 77.32(74.02,80.73) | 61.34(58.34,64.49) | -0.83(-0.88,-0.78) |
| Slovenia | both | 749.00(707.35,789.10) | 568.49(537.08,599.38) | -1.04 | 72.97(67.81,78.44) | 59.66(54.61,65.12) | -0.74(-0.79,-0.69) |
| Solomon Islands | both | 67.28(63.15,71.67) | 135.31(126.93,143.86) | 2.47 | 49.00(37.54,63.19) | 42.82(35.86,50.79) | -0.41(-0.44,-0.39) |
| Somalia | both | 985.70(927.17,1049.77) | 2873.48(2710.53,3056.63) | 3.65 | 32.91(30.85,35.09) | 31.91(30.71,33.14) | -0.09(-0.11,-0.07) |
| South Africa | both | 6963.19(6604.88,7358.74) | 10316.28(9786.36,10885.85) | 1.28 | 38.06(37.15,38.99) | 33.83(33.18,34.49) | -0.44(-0.47,-0.42) |
| South Sudan | both | 718.96(676.18,768.24) | 1045.92(988.99,1106.20) | 1.48 | 27.98(25.87,30.24) | 26.04(24.44,27.72) | -0.24(-0.26,-0.22) |
| Spain | both | 48154.02(45390.06,50982.48) | 50856.40(47925.12,54042.53) | 0.49 | 248.79(246.57,251.03) | 231.70(229.56,233.85) | -0.23(-0.24,-0.21) |
| Sri Lanka | both | 3895.57(3643.56,4181.44) | 4007.91(3725.45,4297.09) | 0.05 | 43.71(42.33,45.11) | 35.93(34.82,37.06) | -0.63(-0.65,-0.60) |
| Sudan | both | 7169.57(6731.65,7651.70) | 12550.76(11821.53,13329.46) | 1.82 | 83.13(81.16,85.15) | 62.81(61.70,63.95) | -0.98(-0.99,-0.97) |
| Suriname | both | 109.70(102.81,117.22) | 152.79(143.27,162.05) | 1.41 | 58.52(47.85,71.00) | 52.51(44.50,61.58) | -0.40(-0.42,-0.39) |
| Sweden | both | 8656.96(8185.68,9098.46) | 9082.35(8593.77,9574.42) | 0.20 | 200.15(195.90,204.48) | 196.21(192.14,200.36) | -0.08(-0.09,-0.06) |
| Switzerland | both | 8769.19(8291.36,9262.16) | 9453.60(8948.83,9962.43) | 0.33 | 235.12(230.16,240.17) | 226.37(221.70,231.13) | -0.14(-0.14,-0.13) |
| Syrian Arab Republic | both | 4238.41(3969.86,4518.93) | 4578.23(4305.86,4847.62) | 0.39 | 80.92(78.38,83.54) | 63.59(61.66,65.58) | -0.86(-0.91,-0.81) |
| Taiwan (Province of China) | both | 7711.37(7269.01,8174.93) | 6933.40(6509.47,7355.25) | -0.45 | 70.18(68.60,71.79) | 54.91(53.58,56.27) | -0.80(-0.82,-0.78) |
| Tajikistan | both | 1909.53(1781.90,2042.29) | 3439.75(3228.13,3679.14) | 2.20 | 84.25(80.24,88.44) | 71.09(68.70,73.54) | -0.60(-0.67,-0.53) |
| Thailand | both | 8052.36(7578.17,8560.88) | 8732.60(8225.38,9324.97) | 0.32 | 26.62(26.03,27.22) | 22.96(22.47,23.46) | -0.48(-0.50,-0.46) |
| Timor-Leste | both | 115.28(108.55,122.42) | 158.52(148.59,168.70) | 0.80 | 31.92(26.24,38.58) | 26.28(22.22,30.91) | -0.84(-0.94,-0.75) |
| Togo | both | 559.31(526.89,592.70) | 1195.00(1130.89,1264.55) | 2.63 | 36.95(33.82,40.34) | 31.35(29.58,33.21) | -0.57(-0.62,-0.52) |
| Tokelau | both | 0.27(0.25,0.28) | 0.22(0.21,0.24) | -1.07 | 40.80(0.00,852.13) | 34.51(0.00,742.30) | -0.60(-0.61,-0.59) |
| Tonga | both | 17.34(16.27,18.40) | 17.78(16.66,18.83) | 0.27 | 43.02(24.62,70.65) | 37.78(22.20,60.39) | -0.39(-0.42,-0.36) |
| Trinidad and Tobago | both | 332.18(311.59,353.33) | 353.90(329.78,376.07) | 0.14 | 55.00(49.19,61.36) | 49.18(44.12,54.71) | -0.42(-0.45,-0.39) |
| Tunisia | both | 3001.76(2806.56,3200.25) | 3774.81(3553.43,4004.62) | 0.74 | 76.76(73.96,79.65) | 60.81(58.86,62.80) | -0.81(-0.85,-0.78) |
| Turkey | both | 30621.27(28648.17,32645.17) | 38475.40(36219.03,40714.99) | 0.70 | 106.24(105.03,107.46) | 82.89(82.06,83.72) | -0.85(-0.87,-0.84) |
| Turkmenistan | both | 1306.68(1222.83,1393.26) | 1643.51(1538.48,1748.47) | 0.73 | 78.53(74.06,83.24) | 62.41(59.42,65.51) | -0.85(-0.88,-0.82) |
| Tuvalu | both | 1.84(1.73,1.95) | 2.11(1.98,2.24) | 0.53 | 41.77(4.34,167.94) | 36.14(4.67,131.51) | -0.44(-0.47,-0.42) |
| Uganda | both | 2026.43(1904.29,2164.89) | 4391.69(4143.25,4677.86) | 2.63 | 29.15(27.82,30.54) | 24.79(24.03,25.58) | -0.65(-0.68,-0.62) |
| Ukraine | both | 18476.97(17492.09,19407.80) | 13934.74(13150.72,14659.66) | -1.09 | 73.18(72.13,74.25) | 63.41(62.32,64.53) | -0.53(-0.58,-0.48) |
| United Arab Emirates | both | 759.71(712.11,811.24) | 3751.40(3506.10,4010.07) | 6.83 | 62.75(58.14,67.69) | 48.56(46.49,50.71) | -0.91(-0.98,-0.84) |
| United Kingdom | both | 70117.04(66665.95,73250.99) | 70748.08(67470.73,73918.59) | 0.11 | 242.35(240.56,244.17) | 224.40(222.72,226.08) | -0.26(-0.28,-0.25) |
| United Republic of Tanzania | both | 3011.52(2756.01,3304.45) | 6383.35(5895.26,6926.23) | 2.58 | 28.37(27.31,29.46) | 25.02(24.39,25.66) | -0.41(-0.44,-0.38) |
| United States of America | both | 145089.88(137926.35,152089.13) | 145305.33(138318.35,152593.17) | -0.18 | 106.09(105.54,106.64) | 94.36(93.87,94.84) | -0.52(-0.61,-0.44) |
| United States Virgin Islands | both | 30.94(29.07,32.82) | 21.82(20.47,23.10) | -1.26 | 55.55(37.60,79.51) | 48.41(30.02,74.59) | -0.50(-0.53,-0.48) |
| Uruguay | both | 1714.50(1606.94,1838.00) | 1824.07(1709.92,1943.35) | 0.17 | 115.62(110.21,121.23) | 109.15(104.18,114.30) | -0.20(-0.21,-0.19) |
| Uzbekistan | both | 7758.59(7233.76,8297.76) | 11999.81(11223.55,12764.41) | 1.51 | 83.86(81.87,85.89) | 67.06(65.86,68.28) | -0.81(-0.85,-0.77) |
| Vanuatu | both | 31.01(29.14,32.92) | 57.78(54.13,61.60) | 2.27 | 47.38(31.85,68.53) | 41.67(31.52,54.25) | -0.43(-0.44,-0.42) |
| Venezuela (Bolivarian Republic of) | both | 2092.94(1973.69,2211.60) | 3044.29(2867.92,3222.78) | 1.58 | 22.87(21.88,23.90) | 20.76(20.03,21.52) | -0.28(-0.33,-0.24) |
| Viet Nam | both | 9075.45(8524.51,9674.36) | 13492.90(12632.21,14319.22) | 1.41 | 30.05(29.40,30.72) | 24.70(24.28,25.12) | -0.67(-0.68,-0.65) |
| Yemen | both | 4494.33(4224.11,4796.40) | 10117.12(9513.14,10755.73) | 2.75 | 86.93(84.33,89.61) | 67.19(65.86,68.55) | -1.03(-1.08,-0.98) |
| Zambia | both | 921.48(868.49,981.11) | 2102.11(1980.65,2236.85) | 2.91 | 28.03(26.13,30.03) | 24.44(23.37,25.55) | -0.49(-0.54,-0.44) |
| Zimbabwe | both | 1758.58(1645.23,1884.86) | 2746.87(2571.51,2923.88) | 1.38 | 40.81(38.82,42.89) | 37.93(36.49,39.41) | -0.15(-0.21,-0.09) |
| Afghanistan | female | 2366.06(2208.87,2525.36) | 6736.95(6321.85,7148.82) | 3.23 | 98.88(94.75,103.17) | 80.54(78.54,82.59) | -0.77(-0.84,-0.71) |
| Albania | female | 623.33(585.66,666.84) | 392.79(369.97,416.60) | -1.75 | 77.65(71.50,84.23) | 60.87(54.96,67.28) | -0.90(-0.94,-0.87) |
| Algeria | female | 4299.00(4007.27,4579.15) | 7080.27(6670.31,7521.90) | 1.77 | 78.33(75.89,80.84) | 62.58(61.12,64.06) | -0.78(-0.82,-0.75) |
| American Samoa | female | 4.26(3.97,4.57) | 4.40(4.12,4.69) | -0.12 | 37.16(10.15,103.59) | 32.86(9.48,85.53) | -0.38(-0.41,-0.34) |
| Andorra | female | 34.79(32.73,36.78) | 47.09(44.38,49.83) | 0.80 | 231.53(160.35,326.43) | 222.83(159.82,306.30) | -0.13(-0.13,-0.13) |
| Angola | female | 1544.26(1435.48,1670.29) | 3690.66(3424.73,3958.16) | 3.09 | 70.04(66.48,73.76) | 54.27(52.49,56.10) | -0.88(-0.94,-0.82) |
| Antigua and Barbuda | female | 9.34(8.76,9.99) | 13.42(12.58,14.32) | 1.25 | 59.08(27.15,115.70) | 53.23(28.49,92.27) | -0.36(-0.38,-0.35) |
| Argentina | female | 9428.36(8747.49,10166.54) | 13037.95(12155.85,13954.23) | 1.16 | 117.86(115.49,120.26) | 110.29(108.40,112.20) | -0.20(-0.21,-0.19) |
| Armenia | female | 644.79(600.54,692.42) | 458.63(427.97,488.27) | -1.34 | 76.89(70.90,83.30) | 60.34(54.78,66.39) | -0.88(-0.92,-0.83) |
| Australia | female | 7527.24(7023.79,8052.17) | 9245.10(8621.81,9924.49) | 0.71 | 168.05(164.26,171.90) | 156.49(153.26,159.77) | -0.18(-0.24,-0.12) |
| Austria | female | 4976.51(4699.94,5265.50) | 4807.77(4536.58,5074.96) | -0.03 | 248.86(241.94,255.94) | 234.17(227.41,241.11) | -0.21(-0.21,-0.20) |
| Azerbaijan | female | 1317.34(1230.82,1408.79) | 1689.26(1572.08,1807.54) | 0.94 | 73.82(69.69,78.18) | 61.03(58.10,64.08) | -0.71(-0.79,-0.63) |
| Bahamas | female | 40.04(37.46,42.98) | 56.73(52.97,60.42) | 1.17 | 57.19(40.60,78.93) | 52.92(39.99,68.88) | -0.33(-0.35,-0.30) |
| Bahrain | female | 78.81(73.74,84.34) | 199.06(186.74,211.38) | 3.85 | 70.48(54.90,90.11) | 58.27(50.24,67.36) | -0.69(-0.71,-0.66) |
| Bangladesh | female | 11802.75(11059.95,12572.58) | 18783.13(17637.42,19918.05) | 1.67 | 50.89(49.93,51.87) | 42.42(41.81,43.03) | -0.58(-0.61,-0.55) |
| Barbados | female | 37.77(35.49,40.55) | 38.69(36.25,41.24) | 0.10 | 55.79(39.32,77.31) | 51.98(36.69,71.96) | -0.24(-0.26,-0.23) |
| Belarus | female | 1941.05(1824.47,2054.29) | 1426.79(1342.09,1510.49) | -1.20 | 76.67(73.27,80.18) | 62.57(59.19,66.11) | -0.73(-0.77,-0.69) |
| Belgium | female | 6236.09(5888.17,6598.79) | 6077.07(5730.37,6449.11) | -0.10 | 253.54(247.25,259.96) | 237.15(231.11,243.32) | -0.22(-0.22,-0.22) |
| Belize | female | 27.41(25.42,29.33) | 66.52(62.18,71.00) | 3.22 | 70.15(45.14,106.45) | 59.77(46.20,76.23) | -0.52(-0.55,-0.48) |
| Benin | female | 415.12(389.72,441.81) | 954.65(898.84,1013.30) | 2.86 | 39.84(35.95,44.09) | 33.20(31.06,35.46) | -0.67(-0.73,-0.62) |
| Bermuda | female | 10.56(9.86,11.28) | 8.09(7.56,8.62) | -0.99 | 60.08(29.12,114.48) | 54.18(22.46,116.57) | -0.39(-0.41,-0.38) |
| Bhutan | female | 69.02(65.08,73.48) | 89.47(83.88,94.88) | 1.26 | 52.82(40.59,68.05) | 44.38(35.60,54.82) | -0.62(-0.63,-0.61) |
| Bolivia (Plurinational State of) | female | 1626.57(1510.22,1752.39) | 2883.91(2682.37,3090.62) | 2.08 | 111.64(106.18,117.34) | 96.15(92.67,99.75) | -0.47(-0.51,-0.42) |
| Bosnia and Herzegovina | female | 919.79(866.10,972.51) | 468.31(441.35,496.82) | -2.17 | 78.91(73.88,84.21) | 60.15(54.69,66.07) | -1.02(-1.07,-0.97) |
| Botswana | female | 125.60(117.37,134.20) | 227.14(212.62,243.85) | 1.89 | 41.41(34.16,49.95) | 34.33(29.98,39.17) | -0.70(-0.75,-0.65) |
| Brazil | female | 36366.18(34502.88,38322.21) | 54339.36(51543.17,57040.60) | 1.41 | 96.81(95.81,97.83) | 90.89(90.12,91.66) | -0.20(-0.21,-0.18) |
| Brunei Darussalam | female | 26.52(24.90,28.17) | 46.58(43.88,49.28) | 1.90 | 40.54(26.12,61.64) | 37.01(27.11,49.59) | -0.30(-0.33,-0.28) |
| Bulgaria | female | 1551.19(1455.64,1643.79) | 903.58(847.89,956.92) | -1.90 | 73.19(69.56,76.97) | 58.20(54.24,62.41) | -0.77(-0.81,-0.73) |
| Burkina Faso | female | 861.76(811.78,913.94) | 1885.37(1776.85,2000.15) | 2.75 | 42.71(39.83,45.75) | 36.31(34.65,38.03) | -0.64(-0.70,-0.59) |
| Burundi | female | 352.01(329.19,376.22) | 739.71(696.15,787.88) | 2.82 | 29.37(26.26,32.79) | 28.03(25.96,30.25) | -0.18(-0.22,-0.13) |
| Cabo Verde | female | 28.65(26.87,30.50) | 44.57(42.00,47.59) | 1.49 | 38.65(25.04,58.21) | 30.63(22.24,41.35) | -0.89(-0.95,-0.83) |
| Cambodia | female | 787.18(737.28,844.72) | 1208.97(1126.71,1293.31) | 1.54 | 32.93(30.61,35.40) | 27.81(26.26,29.44) | -0.58(-0.59,-0.57) |
| Cameroon | female | 815.64(768.39,868.92) | 2081.46(1965.30,2218.01) | 3.29 | 36.21(33.68,38.90) | 29.88(28.57,31.25) | -0.70(-0.76,-0.64) |
| Canada | female | 7179.60(6773.72,7614.75) | 7456.63(7012.51,7912.64) | 0.03 | 95.71(93.49,97.98) | 88.99(86.95,91.07) | -0.27(-0.29,-0.26) |
| Central African Republic | female | 429.17(398.53,460.49) | 773.38(718.88,832.64) | 2.02 | 69.62(62.99,76.82) | 61.08(56.74,65.70) | -0.46(-0.49,-0.44) |
| Chad | female | 556.44(524.22,590.13) | 1250.19(1173.26,1328.78) | 2.73 | 43.99(40.30,47.95) | 37.23(35.10,39.46) | -0.64(-0.70,-0.59) |
| Chile | female | 4203.81(3891.62,4516.38) | 5040.70(4703.48,5401.05) | 0.67 | 118.38(114.81,122.05) | 107.57(104.60,110.62) | -0.33(-0.34,-0.32) |
| China | female | 217018.43(205064.33,229664.04) | 198204.80(187731.18,208436.43) | -0.15 | 69.20(68.91,69.50) | 54.19(53.94,54.44) | -0.83(-0.85,-0.81) |
| Colombia | female | 1955.43(1834.58,2078.96) | 2590.45(2438.47,2751.25) | 0.88 | 23.58(22.52,24.69) | 20.42(19.64,21.23) | -0.51(-0.53,-0.49) |
| Comoros | female | 28.67(26.80,30.39) | 46.81(44.07,49.70) | 1.69 | 28.82(18.93,42.59) | 25.83(18.93,34.52) | -0.36(-0.38,-0.34) |
| Congo | female | 308.94(287.40,332.06) | 614.20(573.71,658.38) | 2.40 | 58.03(51.45,65.31) | 45.93(42.35,49.75) | -0.81(-0.85,-0.76) |
| Cook Islands | female | 1.63(1.53,1.75) | 1.40(1.31,1.49) | -0.40 | 37.01(2.95,172.57) | 32.05(1.95,150.82) | -0.45(-0.47,-0.44) |
| Costa Rica | female | 172.78(161.98,183.53) | 263.88(248.06,280.89) | 1.43 | 23.19(19.78,27.09) | 20.25(17.88,22.88) | -0.45(-0.48,-0.43) |
| Croatia | female | 871.58(819.61,920.62) | 546.28(514.53,579.11) | -1.69 | 71.73(67.02,76.71) | 57.65(52.77,62.91) | -0.78(-0.82,-0.75) |
| Cuba | female | 1849.77(1726.74,1977.56) | 1583.63(1475.56,1688.70) | -0.43 | 62.10(59.27,65.02) | 57.93(55.03,60.96) | -0.24(-0.26,-0.22) |
| Cyprus | female | 492.96(466.22,522.23) | 806.83(754.85,855.14) | 1.87 | 248.37(226.89,271.40) | 222.80(206.99,239.74) | -0.37(-0.40,-0.34) |
| Czechia | female | 1925.15(1808.46,2039.23) | 1418.97(1331.40,1505.76) | -1.11 | 72.64(69.39,76.02) | 57.32(54.17,60.63) | -0.77(-0.82,-0.72) |
| Côte d'Ivoire | female | 982.55(922.06,1042.23) | 1945.46(1831.10,2072.50) | 2.24 | 38.14(35.67,40.76) | 31.80(30.36,33.29) | -0.64(-0.70,-0.58) |
| Democratic People's Republic of Korea | female | 4222.26(3947.16,4512.81) | 4282.27(4005.25,4575.54) | 0.17 | 77.31(74.98,79.71) | 63.73(61.82,65.70) | -0.66(-0.69,-0.63) |
| Democratic Republic of the Congo | female | 5274.57(4891.03,5697.49) | 10909.05(10195.13,11756.93) | 2.61 | 64.87(63.07,66.71) | 55.36(54.29,56.44) | -0.48(-0.60,-0.36) |
| Denmark | female | 3322.32(3133.22,3505.54) | 3048.48(2882.80,3221.23) | -0.31 | 249.74(241.24,258.48) | 236.11(227.68,244.79) | -0.19(-0.20,-0.18) |
| Djibouti | female | 28.82(27.03,30.79) | 78.64(73.80,83.32) | 3.87 | 29.03(19.02,43.29) | 25.51(20.14,31.95) | -0.49(-0.51,-0.48) |
| Dominica | female | 9.77(9.14,10.47) | 8.90(8.32,9.50) | -0.24 | 60.43(28.13,115.99) | 53.57(24.33,103.06) | -0.44(-0.47,-0.41) |
| Dominican Republic | female | 1232.68(1151.66,1321.54) | 1704.92(1593.85,1825.04) | 0.97 | 69.29(65.33,73.46) | 60.36(57.52,63.30) | -0.56(-0.61,-0.51) |
| Ecuador | female | 2223.12(2066.09,2391.92) | 3656.57(3403.60,3917.49) | 1.70 | 93.07(89.14,97.14) | 80.13(77.55,82.78) | -0.51(-0.52,-0.49) |
| Egypt | female | 6107.51(5740.23,6487.65) | 9848.40(9297.34,10409.54) | 1.90 | 48.20(46.98,49.44) | 39.98(39.19,40.79) | -0.45(-0.51,-0.40) |
| El Salvador | female | 328.54(308.14,349.89) | 394.96(371.33,420.69) | 0.58 | 26.67(23.79,29.85) | 22.65(20.47,25.01) | -0.60(-0.63,-0.57) |
| Equatorial Guinea | female | 65.97(61.16,71.05) | 138.48(128.63,149.55) | 2.45 | 69.72(53.58,89.72) | 43.07(35.97,51.34) | -1.86(-1.92,-1.79) |
| Eritrea | female | 205.10(191.69,219.54) | 445.39(417.73,476.14) | 2.82 | 31.55(27.24,36.41) | 27.47(24.91,30.24) | -0.45(-0.50,-0.41) |
| Estonia | female | 287.47(270.35,305.25) | 179.42(168.87,190.71) | -1.65 | 74.19(65.80,83.42) | 60.61(51.71,70.82) | -0.71(-0.74,-0.68) |
| Eswatini | female | 80.06(74.62,85.48) | 111.09(103.47,118.95) | 0.92 | 43.61(34.18,55.18) | 36.92(30.21,44.86) | -0.59(-0.63,-0.55) |
| Ethiopia | female | 3339.67(3146.83,3538.50) | 6764.90(6380.42,7183.97) | 2.35 | 30.79(29.71,31.90) | 27.00(26.34,27.69) | -0.46(-0.50,-0.41) |
| Fiji | female | 77.19(72.02,82.73) | 80.08(74.94,85.73) | 0.21 | 40.67(32.01,51.14) | 35.20(27.91,43.83) | -0.45(-0.48,-0.42) |
| Finland | female | 3472.37(3272.00,3672.49) | 2922.55(2759.30,3083.72) | -0.69 | 267.55(258.54,276.82) | 251.19(242.04,260.62) | -0.21(-0.22,-0.21) |
| France | female | 42865.01(40093.37,45771.90) | 40235.76(37713.04,42641.15) | -0.27 | 294.68(291.89,297.50) | 273.97(271.26,276.71) | -0.24(-0.27,-0.22) |
| Gabon | female | 116.68(108.27,125.57) | 203.01(188.54,217.87) | 1.95 | 56.46(46.19,68.72) | 42.97(37.21,49.42) | -0.91(-0.94,-0.89) |
| Gambia | female | 87.13(82.02,92.82) | 182.62(171.99,194.42) | 2.38 | 40.86(32.22,51.55) | 33.38(28.53,38.94) | -0.77(-0.83,-0.72) |
| Georgia | female | 969.33(905.69,1035.92) | 513.68(477.64,548.35) | -2.27 | 71.30(66.86,75.97) | 60.88(55.59,66.60) | -0.52(-0.58,-0.47) |
| Germany | female | 40397.95(38184.53,42768.40) | 35336.58(33304.90,37458.06) | -0.51 | 204.46(202.44,206.50) | 197.63(195.52,199.75) | -0.14(-0.17,-0.11) |
| Ghana | female | 1442.46(1337.53,1554.28) | 2966.06(2758.37,3183.87) | 2.47 | 42.84(40.58,45.20) | 35.22(33.95,36.53) | -0.77(-0.83,-0.72) |
| Greece | female | 6393.34(6026.64,6764.09) | 5489.94(5164.58,5815.77) | -0.53 | 251.37(245.23,257.62) | 233.60(227.14,240.21) | -0.25(-0.27,-0.23) |
| Greenland | female | 16.81(15.84,17.87) | 13.30(12.52,14.12) | -0.65 | 113.00(64.42,188.08) | 102.95(55.05,177.95) | -0.38(-0.41,-0.36) |
| Grenada | female | 11.98(11.20,12.91) | 14.46(13.54,15.41) | 0.73 | 64.99(32.69,119.70) | 55.36(30.47,93.58) | -0.48(-0.54,-0.42) |
| Guam | female | 11.63(10.88,12.44) | 11.60(10.85,12.35) | -0.18 | 34.08(17.16,63.16) | 29.94(15.21,53.47) | -0.47(-0.50,-0.44) |
| Guatemala | female | 491.92(461.29,524.64) | 1154.60(1086.69,1232.02) | 3.10 | 29.08(26.49,31.87) | 24.01(22.63,25.46) | -0.65(-0.68,-0.62) |
| Guinea | female | 561.44(527.98,598.44) | 1028.54(966.06,1093.59) | 1.99 | 41.20(37.80,44.85) | 34.75(32.59,37.03) | -0.63(-0.69,-0.58) |
| Guinea-Bissau | female | 93.01(87.62,98.80) | 163.22(152.97,173.49) | 1.95 | 41.25(33.03,51.16) | 34.35(29.11,40.40) | -0.69(-0.75,-0.63) |
| Guyana | female | 119.37(111.22,127.82) | 113.24(106.06,120.84) | -0.35 | 61.90(50.93,74.82) | 55.08(45.35,66.35) | -0.39(-0.41,-0.38) |
| Haiti | female | 1122.76(1048.21,1202.30) | 2319.45(2169.71,2483.07) | 2.57 | 76.55(72.03,81.30) | 68.87(66.07,71.76) | -0.37(-0.39,-0.36) |
| Honduras | female | 282.47(264.91,301.66) | 643.86(603.63,688.29) | 2.92 | 28.59(25.24,32.31) | 24.64(22.75,26.66) | -0.52(-0.56,-0.49) |
| Hungary | female | 1917.28(1799.97,2020.42) | 1339.49(1256.08,1418.99) | -1.35 | 73.49(70.19,76.92) | 58.13(54.90,61.53) | -0.79(-0.84,-0.75) |
| Iceland | female | 171.88(161.52,181.68) | 199.68(188.79,211.45) | 0.49 | 266.32(227.90,309.63) | 248.27(214.80,285.84) | -0.25(-0.25,-0.24) |
| India | female | 102616.43(97355.36,108380.50) | 178464.08(169335.55,188461.46) | 1.97 | 52.57(52.24,52.89) | 48.94(48.71,49.16) | -0.26(-0.32,-0.20) |
| Indonesia | female | 7062.57(6652.56,7476.50) | 9825.46(9257.98,10399.61) | 1.20 | 15.36(14.99,15.73) | 13.77(13.50,14.04) | -0.29(-0.33,-0.26) |
| Iran (Islamic Republic of) | female | 10190.15(9672.92,10738.92) | 15021.18(14239.22,15819.09) | 1.51 | 82.62(80.95,84.32) | 62.87(61.85,63.91) | -1.01(-1.06,-0.95) |
| Iraq | female | 3278.79(3058.41,3509.66) | 7530.68(7092.14,8000.80) | 2.69 | 90.60(87.42,93.89) | 68.92(67.35,70.52) | -1.00(-1.03,-0.96) |
| Ireland | female | 2275.31(2138.46,2407.86) | 2892.12(2728.93,3054.93) | 1.03 | 259.09(248.52,269.99) | 236.99(228.19,246.06) | -0.30(-0.31,-0.29) |
| Israel | female | 2874.07(2710.69,3037.62) | 4906.95(4635.89,5174.28) | 1.73 | 237.77(229.09,246.72) | 224.08(217.84,230.46) | -0.19(-0.20,-0.18) |
| Italy | female | 27863.56(26413.19,29387.08) | 23682.47(22232.90,25017.53) | -0.31 | 193.36(191.09,195.66) | 179.44(177.03,181.88) | -0.19(-0.28,-0.10) |
| Jamaica | female | 356.80(333.76,381.31) | 431.11(401.07,459.49) | 0.62 | 63.49(56.84,70.79) | 55.99(50.82,61.55) | -0.44(-0.46,-0.42) |
| Japan | female | 14456.91(13702.43,15209.35) | 11511.37(10885.61,12135.60) | -0.73 | 43.86(43.13,44.59) | 42.79(41.97,43.63) | -0.04(-0.05,-0.02) |
| Jordan | female | 582.58(543.89,623.51) | 1690.75(1594.09,1788.56) | 3.75 | 74.93(68.60,81.76) | 60.04(57.20,62.99) | -0.80(-0.82,-0.78) |
| Kazakhstan | female | 3096.33(2894.94,3314.83) | 2950.32(2760.07,3142.89) | -0.03 | 76.95(74.22,79.75) | 62.11(59.86,64.43) | -0.72(-0.75,-0.69) |
| Kenya | female | 1263.55(1195.41,1336.46) | 2962.59(2801.47,3137.33) | 2.95 | 26.29(24.76,27.89) | 23.47(22.61,24.36) | -0.36(-0.39,-0.33) |
| Kiribati | female | 7.31(6.83,7.82) | 11.66(10.89,12.41) | 1.89 | 40.72(16.35,88.13) | 38.18(19.41,68.82) | -0.11(-0.14,-0.07) |
| Kuwait | female | 267.25(249.53,286.12) | 779.96(732.54,826.59) | 4.30 | 64.94(57.04,73.86) | 54.41(50.43,58.70) | -0.74(-0.79,-0.70) |
| Kyrgyzstan | female | 793.77(738.77,851.30) | 1108.77(1030.18,1188.50) | 1.25 | 79.05(73.39,85.11) | 66.90(63.01,70.99) | -0.53(-0.56,-0.50) |
| Lao People's Democratic Republic | female | 313.25(292.46,335.70) | 537.87(502.96,576.18) | 1.96 | 33.79(30.05,37.89) | 28.40(26.03,30.93) | -0.58(-0.60,-0.55) |
| Latvia | female | 484.40(455.92,511.38) | 258.56(243.02,273.08) | -2.14 | 73.33(66.89,80.26) | 60.52(53.03,68.93) | -0.66(-0.70,-0.62) |
| Lebanon | female | 587.06(550.04,627.48) | 845.32(794.87,900.32) | 1.40 | 78.42(72.17,85.09) | 60.97(56.86,65.33) | -0.92(-0.95,-0.90) |
| Lesotho | female | 191.31(178.69,205.31) | 219.91(205.53,235.85) | 0.27 | 47.52(40.80,55.14) | 40.25(35.02,46.14) | -0.58(-0.62,-0.54) |
| Liberia | female | 161.58(152.35,171.47) | 394.34(372.02,416.64) | 3.12 | 38.68(32.81,45.41) | 32.81(29.60,36.29) | -0.73(-0.79,-0.67) |
| Libya | female | 615.09(573.81,657.73) | 1169.57(1100.07,1239.50) | 2.43 | 74.86(68.74,81.45) | 58.32(55.02,61.79) | -0.85(-0.93,-0.77) |
| Lithuania | female | 678.88(639.04,717.90) | 372.82(349.21,395.26) | -2.10 | 73.10(67.68,78.85) | 59.40(53.33,66.06) | -0.74(-0.77,-0.71) |
| Luxembourg | female | 237.87(224.36,251.64) | 359.28(338.13,380.29) | 1.39 | 240.52(210.48,274.25) | 229.92(206.10,256.14) | -0.16(-0.16,-0.16) |
| Madagascar | female | 777.88(727.22,831.92) | 1782.78(1668.83,1891.08) | 2.90 | 30.62(28.41,32.97) | 27.90(26.59,29.26) | -0.33(-0.35,-0.31) |
| Malawi | female | 651.97(609.39,697.81) | 1219.90(1149.60,1308.23) | 2.13 | 31.34(28.88,33.98) | 27.78(26.17,29.48) | -0.45(-0.49,-0.41) |
| Malaysia | female | 1067.05(1004.51,1133.71) | 1702.58(1599.62,1804.66) | 1.56 | 24.55(23.08,26.11) | 20.67(19.70,21.68) | -0.57(-0.60,-0.53) |
| Maldives | female | 13.29(12.38,14.17) | 27.48(25.73,29.39) | 2.75 | 29.84(15.28,54.46) | 23.95(15.73,35.38) | -0.71(-0.76,-0.66) |
| Mali | female | 693.46(644.77,747.45) | 1456.76(1342.57,1584.98) | 2.57 | 37.78(34.96,40.79) | 30.98(29.34,32.71) | -0.81(-0.86,-0.76) |
| Malta | female | 243.17(228.79,257.28) | 226.82(213.81,240.06) | -0.32 | 250.92(219.96,285.35) | 229.25(199.57,262.78) | -0.29(-0.30,-0.29) |
| Marshall Islands | female | 4.00(3.73,4.30) | 5.40(5.05,5.75) | 0.92 | 43.25(10.67,138.19) | 36.91(12.61,85.45) | -0.49(-0.52,-0.46) |
| Mauritania | female | 174.18(163.68,185.05) | 310.47(291.26,330.49) | 1.91 | 38.98(33.23,45.56) | 32.19(28.63,36.11) | -0.70(-0.76,-0.64) |
| Mauritius | female | 81.11(76.00,86.56) | 77.67(72.49,82.94) | -0.24 | 27.49(21.77,34.43) | 23.62(18.62,29.61) | -0.50(-0.52,-0.48) |
| Mexico | female | 5285.36(5012.83,5591.62) | 7658.40(7245.37,8085.70) | 1.27 | 25.66(24.96,26.39) | 22.41(21.91,22.92) | -0.44(-0.47,-0.42) |
| Micronesia (Federated States of) | female | 8.96(8.38,9.58) | 9.03(8.43,9.65) | -0.14 | 40.94(17.94,84.93) | 35.55(16.16,68.66) | -0.45(-0.46,-0.43) |
| Monaco | female | 16.89(15.94,17.85) | 16.89(15.88,17.94) | 0.19 | 231.99(130.86,399.52) | 222.30(125.50,371.55) | -0.14(-0.15,-0.14) |
| Mongolia | female | 404.94(377.33,434.67) | 606.03(567.84,644.98) | 1.59 | 83.19(74.83,92.37) | 66.15(60.90,71.77) | -0.77(-0.80,-0.74) |
| Montenegro | female | 108.56(102.21,115.28) | 85.89(80.82,90.93) | -0.95 | 70.01(57.45,84.56) | 57.06(45.46,70.94) | -0.76(-0.81,-0.71) |
| Morocco | female | 5237.78(4917.44,5595.71) | 6653.05(6271.45,7035.70) | 0.79 | 86.59(84.21,89.03) | 69.07(67.42,70.75) | -0.81(-0.82,-0.80) |
| Mozambique | female | 1002.85(936.95,1072.89) | 1975.27(1865.31,2101.85) | 2.19 | 34.06(31.94,36.28) | 29.40(28.06,30.78) | -0.54(-0.58,-0.51) |
| Myanmar | female | 3265.09(3054.66,3503.70) | 4155.98(3885.26,4417.54) | 0.77 | 33.28(32.12,34.48) | 27.68(26.84,28.53) | -0.67(-0.69,-0.64) |
| Namibia | female | 130.88(121.92,140.45) | 218.97(204.21,234.68) | 1.65 | 40.97(33.96,49.17) | 34.93(30.41,39.98) | -0.57(-0.61,-0.54) |
| Nauru | female | 0.86(0.80,0.91) | 0.88(0.83,0.94) | -0.03 | 36.32(0.48,267.90) | 32.41(0.50,226.14) | -0.33(-0.42,-0.24) |
| Nepal | female | 2150.00(2029.34,2286.40) | 3249.71(3068.02,3453.30) | 1.39 | 49.27(47.16,51.45) | 37.93(36.63,39.28) | -0.99(-1.03,-0.96) |
| Netherlands | female | 9889.83(9357.03,10486.84) | 8951.40(8442.86,9472.50) | -0.39 | 247.40(242.52,252.35) | 233.82(228.92,238.80) | -0.18(-0.19,-0.18) |
| New Zealand | female | 1685.09(1590.13,1781.49) | 1817.99(1718.88,1924.18) | 0.34 | 186.46(177.65,195.60) | 174.90(166.84,183.27) | -0.20(-0.21,-0.19) |
| Nicaragua | female | 232.12(218.67,247.60) | 410.57(385.22,436.20) | 1.96 | 27.80(24.18,31.89) | 23.60(21.36,26.01) | -0.54(-0.57,-0.52) |
| Niger | female | 743.31(698.05,789.79) | 1802.06(1692.00,1918.89) | 2.99 | 45.12(41.82,48.64) | 39.62(37.72,41.61) | -0.49(-0.55,-0.44) |
| Nigeria | female | 9067.39(8590.63,9578.95) | 18077.64(17089.88,19090.68) | 2.31 | 47.89(46.85,48.94) | 35.58(35.04,36.12) | -1.11(-1.19,-1.03) |
| Niue | female | 0.19(0.18,0.20) | 0.13(0.12,0.14) | -1.75 | 38.79(0.00,904.43) | 33.29(0.00,1180.69) | -0.53(-0.54,-0.52) |
| North Macedonia | female | 377.72(355.29,400.54) | 313.81(295.77,332.82) | -0.65 | 73.73(66.48,81.58) | 58.62(52.16,65.75) | -0.82(-0.86,-0.79) |
| Northern Mariana Islands | female | 4.57(4.26,4.86) | 3.04(2.83,3.25) | -1.57 | 34.15(9.75,97.08) | 31.99(5.52,121.49) | -0.12(-0.16,-0.08) |
| Norway | female | 2054.36(1949.50,2157.34) | 2267.99(2152.81,2381.10) | 0.34 | 192.72(184.45,201.29) | 182.57(175.02,190.38) | -0.20(-0.21,-0.19) |
| Oman | female | 251.61(235.90,268.52) | 529.51(497.67,561.52) | 2.61 | 77.70(68.08,88.46) | 55.03(50.32,60.11) | -1.18(-1.27,-1.10) |
| Pakistan | female | 11828.58(11208.95,12511.28) | 23529.30(22252.85,24916.89) | 2.38 | 52.65(51.68,53.63) | 44.35(43.77,44.93) | -0.62(-0.64,-0.61) |
| Palau | female | 1.42(1.32,1.51) | 1.27(1.19,1.36) | -0.70 | 35.17(2.15,176.90) | 31.03(1.26,180.46) | -0.38(-0.40,-0.36) |
| Palestine | female | 364.20(340.68,388.01) | 788.71(739.60,836.75) | 2.85 | 88.51(79.20,98.72) | 65.62(61.03,70.50) | -1.06(-1.08,-1.04) |
| Panama | female | 132.93(124.66,141.36) | 208.42(195.95,221.77) | 1.55 | 22.75(18.98,27.11) | 20.02(17.39,22.93) | -0.42(-0.44,-0.41) |
| Papua New Guinea | female | 426.33(397.54,456.50) | 1013.54(942.73,1085.23) | 3.16 | 46.39(41.98,51.19) | 41.52(38.98,44.19) | -0.33(-0.36,-0.30) |
| Paraguay | female | 891.15(827.57,961.38) | 1687.31(1565.54,1812.54) | 2.24 | 99.37(92.82,106.30) | 93.92(89.47,98.54) | -0.18(-0.20,-0.16) |
| Peru | female | 5054.42(4697.87,5436.01) | 7690.21(7138.76,8222.67) | 1.44 | 98.11(95.36,100.93) | 85.55(83.64,87.48) | -0.48(-0.50,-0.45) |
| Philippines | female | 4146.31(3925.76,4387.63) | 7115.07(6742.05,7507.53) | 2.00 | 27.82(26.95,28.70) | 25.32(24.73,25.92) | -0.20(-0.27,-0.13) |
| Poland | female | 7374.33(6970.49,7766.00) | 6285.48(5999.76,6554.53) | -0.73 | 77.69(75.90,79.51) | 67.55(65.81,69.33) | -0.43(-0.47,-0.39) |
| Portugal | female | 6715.81(6331.23,7090.47) | 6049.81(5652.18,6422.36) | -0.37 | 264.49(258.20,270.90) | 241.85(235.51,248.34) | -0.29(-0.30,-0.28) |
| Puerto Rico | female | 536.45(502.12,571.78) | 424.18(398.34,451.70) | -0.89 | 56.07(51.42,61.02) | 50.35(45.62,55.47) | -0.40(-0.41,-0.39) |
| Qatar | female | 53.89(50.34,57.56) | 259.59(243.45,275.51) | 6.19 | 68.45(50.77,91.60) | 54.11(47.35,61.76) | -0.81(-0.84,-0.78) |
| Republic of Korea | female | 5956.16(5595.19,6339.97) | 5528.06(5180.83,5869.95) | -0.20 | 47.96(46.73,49.21) | 42.42(41.26,43.61) | -0.38(-0.43,-0.34) |
| Republic of Moldova | female | 841.26(791.56,892.77) | 590.67(555.25,626.41) | -1.38 | 74.72(69.72,80.00) | 62.95(57.79,68.51) | -0.62(-0.67,-0.56) |
| Romania | female | 3960.96(3732.53,4194.18) | 2448.31(2306.12,2597.06) | -1.75 | 70.11(67.93,72.34) | 54.90(52.64,57.23) | -0.84(-0.88,-0.79) |
| Russian Federation | female | 25737.87(24350.39,27161.04) | 21813.68(20723.35,22884.17) | -0.79 | 69.39(68.53,70.25) | 60.46(59.62,61.31) | -0.48(-0.50,-0.45) |
| Rwanda | female | 401.68(369.24,438.32) | 745.17(686.93,808.36) | 2.75 | 26.37(23.73,29.26) | 23.36(21.68,25.15) | -0.43(-0.45,-0.41) |
| Saint Kitts and Nevis | female | 5.64(5.23,6.07) | 8.45(7.91,8.97) | 1.49 | 59.88(19.43,157.73) | 52.84(23.31,104.87) | -0.42(-0.44,-0.40) |
| Saint Lucia | female | 20.24(18.89,21.64) | 26.66(24.95,28.38) | 1.04 | 63.16(37.92,100.96) | 55.50(36.33,81.81) | -0.42(-0.46,-0.38) |
| Saint Vincent and the Grenadines | female | 15.95(14.89,17.10) | 16.29(15.28,17.41) | 0.06 | 64.97(35.91,111.75) | 57.08(32.75,92.87) | -0.43(-0.45,-0.41) |
| Samoa | female | 13.73(12.81,14.71) | 18.09(16.93,19.42) | 0.90 | 39.74(20.94,70.20) | 37.19(21.89,59.64) | -0.16(-0.19,-0.13) |
| San Marino | female | 13.90(13.14,14.70) | 18.69(17.62,19.82) | 1.13 | 236.43(128.64,401.65) | 225.72(133.11,363.78) | -0.15(-0.16,-0.14) |
| Sao Tome and Principe | female | 8.58(8.05,9.13) | 14.83(13.99,15.70) | 1.82 | 35.69(15.19,74.20) | 28.92(15.98,48.98) | -0.82(-0.87,-0.77) |
| Saudi Arabia | female | 2568.38(2401.07,2742.63) | 5518.73(5160.02,5855.46) | 2.87 | 82.40(79.11,85.81) | 55.33(53.86,56.83) | -1.33(-1.41,-1.24) |
| Senegal | female | 652.48(613.22,694.87) | 1204.55(1133.15,1283.88) | 2.04 | 40.21(37.06,43.59) | 33.82(31.90,35.85) | -0.64(-0.69,-0.59) |
| Serbia | female | 1701.38(1603.53,1804.37) | 1181.62(1111.23,1248.96) | -1.57 | 74.03(70.54,77.66) | 58.47(55.13,61.99) | -0.84(-0.88,-0.81) |
| Seychelles | female | 4.23(3.97,4.50) | 5.41(5.08,5.75) | 0.98 | 24.52(6.54,69.12) | 21.62(7.30,51.08) | -0.36(-0.40,-0.32) |
| Sierra Leone | female | 332.57(312.01,355.55) | 684.66(644.55,729.23) | 2.84 | 40.00(35.70,44.74) | 33.91(31.32,36.69) | -0.63(-0.69,-0.58) |
| Singapore | female | 365.09(343.56,387.28) | 546.98(514.00,580.88) | 1.52 | 39.09(35.15,43.37) | 35.05(31.97,38.42) | -0.35(-0.38,-0.32) |
| Slovakia | female | 992.12(934.60,1050.03) | 770.52(727.83,816.15) | -0.99 | 74.19(69.62,78.98) | 57.65(53.46,62.13) | -0.85(-0.89,-0.81) |
| Slovenia | female | 349.59(329.49,368.80) | 252.70(237.40,267.24) | -1.17 | 69.93(62.76,77.74) | 55.97(48.94,63.88) | -0.77(-0.81,-0.73) |
| Solomon Islands | female | 33.08(30.80,35.46) | 66.58(62.29,71.25) | 2.48 | 47.42(31.99,68.62) | 41.60(32.15,53.11) | -0.39(-0.43,-0.35) |
| Somalia | female | 505.30(473.09,537.06) | 1431.52(1340.59,1522.28) | 3.51 | 33.91(30.97,37.07) | 32.83(31.10,34.64) | -0.12(-0.15,-0.10) |
| South Africa | female | 3687.33(3490.03,3898.79) | 5266.89(4996.57,5557.70) | 1.12 | 39.20(37.91,40.52) | 34.66(33.73,35.61) | -0.48(-0.51,-0.44) |
| South Sudan | female | 349.30(326.74,373.90) | 564.84(529.89,599.40) | 1.85 | 28.91(25.79,32.36) | 26.74(24.54,29.11) | -0.28(-0.30,-0.25) |
| Spain | female | 23982.05(22491.36,25404.04) | 25070.58(23498.54,26706.59) | 0.42 | 249.30(246.15,252.48) | 232.11(229.06,235.19) | -0.22(-0.23,-0.21) |
| Sri Lanka | female | 1971.08(1834.79,2124.66) | 2040.98(1898.43,2195.18) | 0.09 | 43.72(41.80,45.71) | 35.94(34.39,37.54) | -0.61(-0.65,-0.58) |
| Sudan | female | 3844.32(3592.64,4105.53) | 6679.40(6269.63,7105.73) | 1.82 | 86.34(83.54,89.21) | 65.23(63.64,66.85) | -0.99(-1.00,-0.98) |
| Suriname | female | 55.62(51.95,59.36) | 79.21(74.21,84.34) | 1.48 | 60.01(45.06,78.59) | 53.84(42.62,67.18) | -0.41(-0.42,-0.39) |
| Sweden | female | 4243.46(4004.06,4470.88) | 4419.01(4165.11,4657.80) | 0.20 | 201.51(195.40,207.76) | 197.11(191.23,203.13) | -0.07(-0.08,-0.06) |
| Switzerland | female | 4147.81(3920.38,4381.65) | 4497.86(4233.97,4764.95) | 0.37 | 230.61(223.55,237.87) | 221.42(214.79,228.24) | -0.13(-0.14,-0.13) |
| Syrian Arab Republic | female | 2184.32(2038.01,2332.61) | 2580.44(2421.96,2747.53) | 0.65 | 84.24(80.56,88.07) | 65.90(63.29,68.61) | -0.88(-0.93,-0.84) |
| Taiwan (Province of China) | female | 3370.30(3149.98,3596.41) | 2987.01(2776.70,3165.21) | -0.45 | 61.90(59.80,64.06) | 49.05(47.23,50.93) | -0.75(-0.77,-0.73) |
| Tajikistan | female | 933.92(868.17,1007.34) | 1629.75(1518.88,1745.48) | 2.13 | 81.98(76.37,87.97) | 68.01(64.71,71.44) | -0.62(-0.67,-0.57) |
| Thailand | female | 4197.23(3938.80,4499.26) | 4373.08(4105.69,4667.46) | 0.19 | 26.96(26.15,27.81) | 23.32(22.62,24.05) | -0.45(-0.47,-0.42) |
| Timor-Leste | female | 58.08(54.11,61.89) | 82.47(77.23,88.28) | 0.90 | 32.28(24.36,42.25) | 26.59(20.98,33.36) | -0.82(-0.91,-0.73) |
| Togo | female | 306.62(288.62,325.57) | 637.44(601.31,673.52) | 2.53 | 37.93(33.65,42.68) | 31.93(29.48,34.55) | -0.62(-0.68,-0.56) |
| Tokelau | female | 0.14(0.13,0.15) | 0.11(0.10,0.11) | -1.35 | 40.02(0.00,1547.51) | 33.53(0.00,1820.48) | -0.59(-0.61,-0.58) |
| Tonga | female | 8.93(8.35,9.54) | 9.04(8.44,9.62) | 0.21 | 42.05(18.63,83.18) | 36.82(16.78,70.58) | -0.39(-0.42,-0.36) |
| Trinidad and Tobago | female | 169.39(158.43,180.59) | 178.68(165.93,190.51) | 0.10 | 56.39(48.14,65.75) | 50.44(43.22,58.63) | -0.42(-0.45,-0.39) |
| Tunisia | female | 1568.11(1458.94,1666.35) | 1990.50(1866.16,2121.42) | 0.79 | 79.88(75.87,84.08) | 63.22(60.44,66.10) | -0.83(-0.87,-0.79) |
| Turkey | female | 16259.06(15200.97,17334.29) | 19764.79(18559.59,20919.17) | 0.59 | 114.55(112.77,116.36) | 87.73(86.51,88.97) | -0.92(-0.94,-0.90) |
| Turkmenistan | female | 643.85(599.27,690.76) | 737.52(691.19,786.77) | 0.43 | 76.50(70.34,83.15) | 59.49(55.27,63.96) | -0.89(-0.91,-0.86) |
| Tuvalu | female | 0.99(0.93,1.06) | 0.97(0.90,1.03) | -0.05 | 40.95(0.99,242.89) | 35.11(0.76,213.72) | -0.46(-0.49,-0.43) |
| Uganda | female | 1077.89(1006.11,1152.13) | 2350.29(2214.35,2506.95) | 2.63 | 30.04(28.16,32.03) | 25.48(24.40,26.59) | -0.67(-0.71,-0.64) |
| Ukraine | female | 9242.62(8730.21,9760.71) | 6839.15(6426.71,7246.59) | -1.14 | 72.18(70.71,73.68) | 62.15(60.60,63.74) | -0.54(-0.58,-0.50) |
| United Arab Emirates | female | 222.38(207.88,238.16) | 940.34(877.85,1004.35) | 5.93 | 66.48(57.45,76.93) | 51.91(48.21,55.87) | -0.88(-0.94,-0.81) |
| United Kingdom | female | 31105.74(29547.52,32528.43) | 30875.71(29393.16,32290.36) | 0.05 | 215.58(213.17,218.00) | 197.98(195.74,200.25) | -0.30(-0.30,-0.29) |
| United Republic of Tanzania | female | 1642.29(1499.39,1802.87) | 3459.12(3181.72,3775.20) | 2.55 | 29.20(27.73,30.73) | 25.71(24.84,26.60) | -0.43(-0.46,-0.40) |
| United States of America | female | 70059.99(66601.77,73625.21) | 71178.87(67698.20,74866.11) | -0.11 | 103.45(102.68,104.22) | 92.93(92.24,93.62) | -0.48(-0.56,-0.39) |
| United States Virgin Islands | female | 16.64(15.54,17.75) | 11.74(10.98,12.50) | -1.27 | 56.81(32.73,92.60) | 49.57(25.04,89.74) | -0.50(-0.53,-0.48) |
| Uruguay | female | 873.38(812.72,941.39) | 928.94(867.45,992.18) | 0.18 | 116.14(108.57,124.11) | 109.32(102.37,116.64) | -0.20(-0.22,-0.19) |
| Uzbekistan | female | 3800.18(3532.48,4072.27) | 5730.66(5337.86,6100.49) | 1.49 | 81.62(78.86,84.48) | 64.20(62.54,65.89) | -0.82(-0.85,-0.80) |
| Vanuatu | female | 15.30(14.35,16.33) | 28.81(26.78,30.91) | 2.30 | 45.78(25.24,78.63) | 40.42(26.85,59.01) | -0.40(-0.43,-0.38) |
| Venezuela (Bolivarian Republic of) | female | 1083.14(1017.15,1151.25) | 1591.05(1495.82,1695.58) | 1.55 | 23.42(22.02,24.91) | 21.21(20.18,22.29) | -0.28(-0.33,-0.24) |
| Viet Nam | female | 4951.65(4626.73,5309.26) | 6713.56(6274.93,7151.49) | 1.11 | 30.43(29.54,31.33) | 25.06(24.46,25.68) | -0.64(-0.66,-0.63) |
| Yemen | female | 2361.89(2206.25,2518.52) | 5235.31(4915.42,5576.52) | 2.71 | 90.33(86.58,94.22) | 69.48(67.57,71.44) | -1.05(-1.10,-0.99) |
| Zambia | female | 493.04(462.26,525.96) | 1094.68(1029.40,1169.85) | 2.78 | 28.87(26.22,31.75) | 25.14(23.61,26.76) | -0.51(-0.57,-0.46) |
| Zimbabwe | female | 940.48(880.01,1011.15) | 1472.47(1370.50,1572.19) | 1.36 | 41.85(39.07,44.81) | 38.79(36.79,40.88) | -0.18(-0.24,-0.11) |
| Afghanistan | male | 1850.27(1725.44,1993.53) | 6772.90(6359.12,7236.86) | 3.88 | 91.76(87.29,96.45) | 76.96(75.06,78.89) | -0.65(-0.73,-0.57) |
| Albania | male | 717.36(675.56,757.71) | 446.91(422.70,472.88) | -1.95 | 84.30(78.16,90.83) | 68.64(62.39,75.36) | -0.88(-0.96,-0.79) |
| Algeria | male | 4004.54(3737.07,4278.21) | 6637.69(6195.07,7096.65) | 1.80 | 72.10(69.77,74.50) | 57.68(56.30,59.10) | -0.76(-0.79,-0.73) |
| American Samoa | male | 4.47(4.17,4.76) | 4.56(4.25,4.87) | -0.17 | 39.22(11.41,101.43) | 34.71(10.27,89.51) | -0.41(-0.44,-0.38) |
| Andorra | male | 43.26(40.80,45.64) | 52.56(49.38,55.66) | 0.31 | 240.41(173.32,328.22) | 232.60(170.34,313.97) | -0.13(-0.14,-0.12) |
| Angola | male | 1515.31(1402.40,1623.96) | 3199.26(2976.96,3430.74) | 2.63 | 66.73(63.32,70.29) | 51.86(50.04,53.75) | -0.86(-0.91,-0.80) |
| Antigua and Barbuda | male | 8.23(7.67,8.78) | 11.97(11.20,12.74) | 1.36 | 56.14(24.18,115.09) | 50.56(26.03,89.45) | -0.35(-0.37,-0.34) |
| Argentina | male | 9113.53(8516.18,9737.51) | 12756.05(11890.94,13621.95) | 1.21 | 116.92(114.53,119.35) | 110.31(108.41,112.25) | -0.19(-0.20,-0.17) |
| Armenia | male | 662.43(616.41,706.17) | 490.69(458.27,522.54) | -1.33 | 81.06(74.81,87.76) | 65.78(60.02,72.01) | -0.84(-0.93,-0.76) |
| Australia | male | 7414.98(6937.14,7885.85) | 8875.58(8274.40,9481.77) | 0.62 | 162.27(158.59,166.01) | 149.62(146.50,152.80) | -0.23(-0.28,-0.18) |
| Austria | male | 5412.52(5124.85,5739.80) | 5262.18(4972.22,5571.63) | -0.10 | 259.87(252.95,266.95) | 245.31(238.61,252.18) | -0.21(-0.23,-0.20) |
| Azerbaijan | male | 1325.27(1235.43,1422.31) | 1891.07(1766.75,2019.82) | 1.20 | 78.01(73.64,82.62) | 66.41(63.43,69.52) | -0.68(-0.80,-0.57) |
| Bahamas | male | 36.45(34.04,39.00) | 51.03(47.37,54.44) | 1.12 | 54.39(37.85,76.41) | 50.35(37.45,66.40) | -0.31(-0.34,-0.28) |
| Bahrain | male | 123.71(114.99,132.72) | 349.24(323.25,374.92) | 4.82 | 64.57(52.93,78.55) | 53.16(46.84,60.30) | -0.68(-0.70,-0.66) |
| Bangladesh | male | 11925.85(11216.93,12729.68) | 16574.71(15571.21,17606.14) | 1.14 | 48.93(48.03,49.84) | 40.68(40.06,41.31) | -0.58(-0.61,-0.55) |
| Barbados | male | 34.46(32.22,36.86) | 34.89(32.58,37.25) | 0.04 | 53.16(36.75,75.03) | 49.35(34.21,69.20) | -0.24(-0.25,-0.23) |
| Belarus | male | 1993.52(1875.73,2110.56) | 1509.88(1420.60,1604.26) | -1.17 | 78.83(75.38,82.40) | 65.31(61.95,68.84) | -0.72(-0.78,-0.65) |
| Belgium | male | 6825.41(6443.64,7214.42) | 6516.89(6130.57,6926.57) | -0.19 | 264.79(258.52,271.19) | 248.40(242.31,254.62) | -0.23(-0.24,-0.22) |
| Belize | male | 26.83(25.04,28.75) | 60.89(56.77,65.07) | 2.97 | 66.75(42.95,101.04) | 56.90(43.43,73.40) | -0.51(-0.54,-0.47) |
| Benin | male | 323.95(304.80,344.67) | 847.83(797.42,901.34) | 3.37 | 37.63(33.51,42.19) | 31.99(29.80,34.31) | -0.55(-0.60,-0.51) |
| Bermuda | male | 9.78(9.11,10.43) | 7.63(7.10,8.15) | -0.90 | 57.24(26.92,110.78) | 51.56(20.56,113.38) | -0.39(-0.40,-0.38) |
| Bhutan | male | 77.14(72.41,82.26) | 97.25(91.74,103.24) | 1.33 | 50.84(39.54,64.81) | 42.64(34.52,52.23) | -0.62(-0.63,-0.61) |
| Bolivia (Plurinational State of) | male | 1472.20(1369.52,1581.74) | 2737.32(2546.49,2944.18) | 2.29 | 105.97(100.52,111.66) | 91.05(87.66,94.55) | -0.46(-0.51,-0.42) |
| Bosnia and Herzegovina | male | 1058.92(997.37,1124.04) | 541.47(509.49,575.92) | -2.14 | 85.74(80.62,91.12) | 67.74(62.04,73.87) | -1.00(-1.08,-0.92) |
| Botswana | male | 103.65(96.84,110.97) | 214.04(199.41,229.42) | 2.40 | 39.18(31.69,48.14) | 32.70(28.44,37.46) | -0.65(-0.69,-0.60) |
| Brazil | male | 33405.37(31684.27,35189.89) | 49979.35(47405.18,52557.43) | 1.43 | 91.97(90.97,92.97) | 86.39(85.63,87.15) | -0.20(-0.21,-0.19) |
| Brunei Darussalam | male | 31.12(29.29,33.23) | 55.73(52.58,59.02) | 1.98 | 40.19(26.98,58.81) | 38.28(28.85,50.04) | -0.16(-0.17,-0.14) |
| Bulgaria | male | 1695.78(1597.15,1800.13) | 1100.53(1031.77,1168.68) | -1.54 | 79.39(75.63,83.30) | 65.49(61.48,69.74) | -0.73(-0.81,-0.65) |
| Burkina Faso | male | 657.25(616.64,702.10) | 1597.81(1500.05,1699.29) | 3.17 | 40.31(37.17,43.65) | 34.95(33.21,36.78) | -0.52(-0.56,-0.47) |
| Burundi | male | 305.69(285.53,327.17) | 685.65(645.03,731.86) | 3.18 | 27.59(24.45,31.09) | 26.45(24.46,28.57) | -0.12(-0.16,-0.09) |
| Cabo Verde | male | 22.49(21.05,24.03) | 46.85(44.05,49.80) | 2.62 | 36.43(21.19,61.07) | 29.53(21.62,39.58) | -0.77(-0.83,-0.72) |
| Cambodia | male | 600.38(563.15,640.49) | 1132.93(1059.44,1207.22) | 2.23 | 32.31(29.66,35.16) | 27.11(25.54,28.76) | -0.65(-0.67,-0.63) |
| Cameroon | male | 699.34(660.41,744.02) | 1940.63(1819.87,2073.97) | 3.65 | 34.22(31.66,36.95) | 28.80(27.50,30.16) | -0.58(-0.62,-0.54) |
| Canada | male | 7616.92(7162.55,8052.97) | 7709.55(7273.24,8153.20) | -0.08 | 99.40(97.16,101.67) | 91.93(89.87,94.03) | -0.29(-0.31,-0.28) |
| Central African Republic | male | 383.61(354.86,415.27) | 693.09(646.96,743.97) | 2.03 | 66.34(59.65,73.66) | 58.26(53.92,62.88) | -0.44(-0.46,-0.42) |
| Chad | male | 456.09(428.98,487.98) | 1097.97(1029.93,1168.28) | 3.03 | 41.57(37.70,45.77) | 35.88(33.69,38.18) | -0.52(-0.56,-0.47) |
| Chile | male | 4001.79(3722.67,4285.76) | 5054.20(4693.39,5404.63) | 0.77 | 117.45(113.81,121.19) | 107.45(104.50,110.47) | -0.32(-0.34,-0.29) |
| China | male | 259347.99(245561.99,273035.27) | 253085.52(239956.86,266113.39) | 0.02 | 79.96(79.65,80.27) | 62.52(62.27,62.78) | -0.83(-0.85,-0.82) |
| Colombia | male | 1767.86(1661.17,1871.89) | 2383.57(2231.83,2539.39) | 0.96 | 22.45(21.40,23.55) | 19.52(18.74,20.32) | -0.49(-0.51,-0.48) |
| Comoros | male | 25.24(23.59,26.96) | 45.14(42.32,47.89) | 2.08 | 27.01(17.13,41.08) | 24.39(17.77,32.75) | -0.31(-0.33,-0.29) |
| Congo | male | 281.91(262.45,304.80) | 572.40(533.24,614.68) | 2.49 | 55.26(48.67,62.60) | 43.96(40.42,47.73) | -0.78(-0.82,-0.75) |
| Cook Islands | male | 1.79(1.67,1.90) | 1.33(1.24,1.43) | -0.92 | 39.12(3.69,172.28) | 33.85(1.78,168.62) | -0.49(-0.50,-0.48) |
| Costa Rica | male | 161.61(150.93,172.39) | 234.38(219.87,249.24) | 1.22 | 22.10(18.75,25.95) | 19.36(16.96,22.02) | -0.44(-0.47,-0.42) |
| Croatia | male | 985.84(927.00,1043.17) | 646.24(605.47,682.04) | -1.65 | 77.78(72.97,82.84) | 64.85(59.83,70.24) | -0.75(-0.83,-0.67) |
| Cuba | male | 1767.83(1651.11,1892.04) | 1551.17(1449.75,1651.41) | -0.35 | 59.05(56.30,61.91) | 55.03(52.27,57.92) | -0.24(-0.26,-0.21) |
| Cyprus | male | 537.39(509.09,566.30) | 831.05(785.06,879.36) | 1.60 | 259.29(237.81,282.25) | 232.42(216.49,249.44) | -0.39(-0.43,-0.35) |
| Czechia | male | 2133.90(2011.61,2251.23) | 1724.73(1612.73,1823.39) | -0.80 | 78.87(75.52,82.34) | 64.57(61.37,67.93) | -0.74(-0.81,-0.67) |
| Côte d'Ivoire | male | 973.00(916.42,1032.90) | 1998.68(1876.40,2127.72) | 2.46 | 35.98(33.68,38.41) | 30.64(29.30,32.04) | -0.51(-0.57,-0.46) |
| Democratic People's Republic of Korea | male | 4717.91(4427.72,5007.12) | 5974.32(5600.80,6342.83) | 1.07 | 94.28(91.60,97.02) | 77.58(75.60,79.60) | -0.68(-0.71,-0.66) |
| Democratic Republic of the Congo | male | 4792.50(4451.67,5172.90) | 10645.37(9875.16,11466.29) | 2.88 | 61.77(59.94,63.65) | 52.86(51.83,53.91) | -0.46(-0.58,-0.34) |
| Denmark | male | 3645.53(3442.81,3856.28) | 3313.84(3134.58,3503.44) | -0.41 | 260.31(251.86,268.98) | 247.66(239.20,256.35) | -0.19(-0.20,-0.17) |
| Djibouti | male | 32.78(30.62,35.00) | 79.84(75.22,84.94) | 3.48 | 27.22(18.23,39.88) | 24.06(19.07,30.01) | -0.44(-0.47,-0.42) |
| Dominica | male | 10.00(9.38,10.64) | 8.89(8.31,9.49) | -0.43 | 57.53(27.02,110.78) | 50.95(23.12,97.91) | -0.44(-0.47,-0.40) |
| Dominican Republic | male | 1087.03(1012.35,1164.76) | 1664.12(1557.26,1775.55) | 1.43 | 65.90(61.90,70.10) | 57.49(54.75,60.32) | -0.54(-0.58,-0.50) |
| Ecuador | male | 2041.27(1894.33,2202.33) | 3423.40(3185.19,3689.30) | 1.76 | 88.40(84.52,92.43) | 76.33(73.78,78.94) | -0.49(-0.51,-0.48) |
| Egypt | male | 6213.14(5828.28,6626.48) | 9574.08(9041.65,10158.50) | 1.72 | 47.38(46.19,48.59) | 36.81(36.08,37.56) | -0.68(-0.73,-0.62) |
| El Salvador | male | 283.45(265.51,302.86) | 317.72(296.39,339.05) | 0.32 | 25.41(22.45,28.69) | 21.66(19.32,24.22) | -0.59(-0.63,-0.56) |
| Equatorial Guinea | male | 51.17(47.45,55.01) | 156.60(145.07,169.51) | 3.97 | 66.42(48.86,89.12) | 41.18(34.35,49.29) | -1.83(-1.90,-1.76) |
| Eritrea | male | 190.03(177.72,203.09) | 438.04(410.86,466.96) | 3.09 | 29.65(25.40,34.51) | 25.92(23.49,28.56) | -0.40(-0.45,-0.35) |
| Estonia | male | 292.55(275.13,310.63) | 200.20(187.99,212.49) | -1.27 | 76.32(67.81,85.64) | 63.12(54.39,73.07) | -0.70(-0.75,-0.65) |
| Eswatini | male | 62.46(58.37,67.35) | 100.13(93.63,107.42) | 1.44 | 41.23(31.23,53.69) | 35.28(28.57,43.27) | -0.54(-0.57,-0.51) |
| Ethiopia | male | 2945.27(2788.37,3111.47) | 6317.97(5974.22,6697.02) | 2.56 | 29.02(27.95,30.12) | 25.58(24.92,26.24) | -0.41(-0.44,-0.37) |
| Fiji | male | 80.50(75.30,86.07) | 89.23(83.52,94.77) | 0.39 | 43.03(34.04,53.83) | 37.23(29.90,45.82) | -0.49(-0.52,-0.46) |
| Finland | male | 3849.18(3629.26,4065.55) | 3276.63(3099.10,3461.87) | -0.66 | 280.26(271.31,289.46) | 263.99(254.93,273.32) | -0.22(-0.23,-0.21) |
| France | male | 44363.76(41742.93,47216.67) | 40866.86(38381.93,43272.31) | -0.38 | 298.48(295.70,301.29) | 281.00(278.24,283.78) | -0.24(-0.26,-0.23) |
| Gabon | male | 116.49(107.43,125.19) | 176.62(164.47,189.88) | 1.43 | 53.87(44.17,65.38) | 41.13(35.25,47.77) | -0.89(-0.91,-0.87) |
| Gambia | male | 79.53(74.64,84.34) | 163.33(153.17,173.68) | 2.32 | 38.55(30.31,48.58) | 32.09(27.20,37.71) | -0.65(-0.70,-0.60) |
| Georgia | male | 974.58(907.39,1041.01) | 574.52(537.49,612.36) | -1.99 | 75.27(70.60,80.19) | 66.40(61.02,72.18) | -0.49(-0.58,-0.40) |
| Germany | male | 45261.40(42652.39,47719.63) | 40460.41(38227.30,42686.47) | -0.55 | 214.72(212.72,216.74) | 208.76(206.70,210.84) | -0.12(-0.16,-0.08) |
| Ghana | male | 1268.31(1177.43,1367.40) | 2592.98(2404.93,2792.25) | 2.46 | 40.19(37.94,42.54) | 33.83(32.52,35.18) | -0.61(-0.64,-0.58) |
| Greece | male | 6689.85(6306.85,7064.01) | 5781.79(5436.56,6107.02) | -0.54 | 262.36(256.10,268.73) | 244.62(238.09,251.31) | -0.25(-0.27,-0.22) |
| Greenland | male | 23.41(22.09,24.76) | 15.44(14.56,16.30) | -1.39 | 118.91(75.26,184.12) | 107.73(60.66,180.71) | -0.41(-0.44,-0.39) |
| Grenada | male | 11.43(10.67,12.25) | 14.73(13.73,15.72) | 0.93 | 61.74(30.24,117.04) | 52.69(29.17,88.76) | -0.47(-0.53,-0.40) |
| Guam | male | 14.41(13.45,15.37) | 13.41(12.55,14.30) | -0.40 | 35.95(19.57,62.18) | 31.66(16.97,54.41) | -0.50(-0.54,-0.46) |
| Guatemala | male | 439.73(413.51,471.66) | 998.46(938.10,1067.48) | 2.99 | 27.72(25.12,30.54) | 22.97(21.52,24.50) | -0.64(-0.67,-0.61) |
| Guinea | male | 452.20(425.33,479.52) | 829.00(782.00,884.39) | 1.89 | 38.85(35.29,42.70) | 33.47(31.15,35.93) | -0.52(-0.57,-0.47) |
| Guinea-Bissau | male | 75.02(70.79,80.10) | 140.89(132.54,150.46) | 2.19 | 38.96(30.28,49.67) | 33.09(27.65,39.45) | -0.57(-0.61,-0.52) |
| Guyana | male | 110.62(103.12,118.04) | 105.05(98.54,112.16) | -0.34 | 58.87(48.09,71.65) | 52.43(42.80,63.67) | -0.38(-0.40,-0.36) |
| Haiti | male | 972.84(906.62,1038.66) | 1990.83(1853.31,2138.33) | 2.52 | 72.70(68.12,77.52) | 65.78(62.89,68.78) | -0.35(-0.36,-0.33) |
| Honduras | male | 254.73(239.67,272.56) | 552.91(519.20,589.40) | 2.70 | 27.30(23.94,31.04) | 23.57(21.60,25.67) | -0.52(-0.56,-0.48) |
| Hungary | male | 2089.83(1965.09,2214.19) | 1573.89(1475.58,1667.95) | -1.12 | 79.68(76.26,83.22) | 65.51(62.17,69.00) | -0.76(-0.83,-0.69) |
| Iceland | male | 188.80(178.52,199.34) | 225.29(212.97,237.96) | 0.54 | 279.28(240.74,322.56) | 260.63(227.49,297.68) | -0.26(-0.27,-0.25) |
| India | male | 107064.61(101568.08,113013.03) | 181984.40(172836.26,192314.74) | 1.87 | 50.56(50.25,50.87) | 47.12(46.91,47.34) | -0.25(-0.31,-0.20) |
| Indonesia | male | 6265.60(5919.12,6636.99) | 9611.13(9048.58,10157.91) | 1.47 | 14.57(14.20,14.94) | 12.95(12.69,13.21) | -0.42(-0.46,-0.38) |
| Iran (Islamic Republic of) | male | 9538.41(9055.84,10047.41) | 14544.16(13789.79,15296.89) | 1.68 | 76.09(74.50,77.71) | 57.93(56.97,58.90) | -1.00(-1.04,-0.95) |
| Iraq | male | 3217.64(3000.30,3438.18) | 7639.97(7177.76,8129.20) | 2.74 | 85.57(82.55,88.69) | 66.21(64.71,67.73) | -0.92(-0.95,-0.88) |
| Ireland | male | 2402.55(2275.40,2534.97) | 2965.38(2786.85,3136.82) | 0.97 | 271.37(260.60,282.48) | 248.18(239.12,257.52) | -0.31(-0.33,-0.29) |
| Israel | male | 2917.02(2758.51,3085.66) | 5190.20(4914.74,5471.55) | 1.89 | 247.25(238.28,256.50) | 233.95(227.62,240.42) | -0.19(-0.21,-0.17) |
| Italy | male | 30045.89(28533.71,31552.89) | 26184.05(24673.74,27594.76) | -0.20 | 206.66(204.33,209.02) | 193.56(191.12,196.02) | -0.11(-0.17,-0.05) |
| Jamaica | male | 324.38(302.38,345.87) | 399.20(372.05,428.70) | 0.73 | 60.48(53.87,67.76) | 53.30(48.18,58.83) | -0.43(-0.45,-0.41) |
| Japan | male | 14686.38(13913.55,15432.33) | 12550.07(11870.68,13215.99) | -0.48 | 43.49(42.78,44.21) | 44.30(43.50,45.13) | 0.11(0.10,0.13) |
| Jordan | male | 597.94(559.37,638.06) | 1877.23(1763.92,2000.34) | 4.04 | 68.87(63.07,75.13) | 55.04(52.57,57.60) | -0.80(-0.82,-0.79) |
| Kazakhstan | male | 3246.91(3024.10,3465.60) | 3169.59(2951.67,3395.12) | -0.03 | 81.25(78.42,84.16) | 67.65(65.30,70.07) | -0.69(-0.76,-0.63) |
| Kenya | male | 1134.69(1073.33,1199.83) | 2744.27(2599.20,2902.80) | 3.10 | 24.34(22.87,25.90) | 22.18(21.34,23.05) | -0.26(-0.30,-0.23) |
| Kiribati | male | 7.07(6.64,7.53) | 11.19(10.53,11.86) | 1.86 | 43.07(16.96,93.28) | 40.40(20.12,74.09) | -0.13(-0.16,-0.09) |
| Kuwait | male | 383.30(356.63,410.25) | 819.10(765.96,873.13) | 3.13 | 59.45(53.43,66.06) | 49.23(45.69,53.04) | -0.77(-0.81,-0.73) |
| Kyrgyzstan | male | 829.91(769.68,893.35) | 1204.14(1127.60,1283.36) | 1.27 | 83.43(77.58,89.67) | 72.85(68.77,77.13) | -0.49(-0.55,-0.43) |
| Lao People's Democratic Republic | male | 270.86(253.26,287.42) | 518.65(485.91,550.88) | 2.25 | 33.19(29.27,37.52) | 27.74(25.39,30.27) | -0.65(-0.68,-0.62) |
| Latvia | male | 486.78(460.24,514.99) | 276.15(259.42,291.29) | -1.94 | 75.44(68.87,82.49) | 62.99(55.55,71.30) | -0.65(-0.71,-0.59) |
| Lebanon | male | 509.24(476.44,545.77) | 752.58(702.35,798.66) | 1.47 | 72.06(65.82,78.75) | 55.96(52.00,60.17) | -0.92(-0.95,-0.89) |
| Lesotho | male | 172.02(160.39,184.18) | 217.94(202.75,234.83) | 0.66 | 44.85(38.22,52.39) | 38.35(33.33,44.00) | -0.52(-0.55,-0.49) |
| Liberia | male | 135.85(128.16,144.62) | 377.72(356.38,401.58) | 3.66 | 36.48(30.52,43.31) | 31.62(28.49,35.02) | -0.61(-0.66,-0.56) |
| Libya | male | 685.12(641.33,729.46) | 1168.10(1093.87,1245.38) | 2.31 | 68.74(63.55,74.28) | 53.34(50.30,56.52) | -0.86(-0.95,-0.78) |
| Lithuania | male | 683.59(645.36,722.28) | 391.25(368.82,414.35) | -2.03 | 75.24(69.69,81.11) | 61.77(55.69,68.41) | -0.74(-0.80,-0.68) |
| Luxembourg | male | 263.82(249.26,278.19) | 394.69(372.08,417.35) | 1.33 | 250.29(220.64,283.38) | 240.58(216.87,266.55) | -0.16(-0.17,-0.15) |
| Madagascar | male | 709.09(665.19,758.36) | 1653.36(1554.62,1759.41) | 2.97 | 28.73(26.56,31.05) | 26.34(25.05,27.68) | -0.28(-0.32,-0.24) |
| Malawi | male | 577.44(540.33,617.50) | 1079.32(1011.63,1149.26) | 2.15 | 29.43(26.99,32.06) | 26.24(24.64,27.92) | -0.40(-0.43,-0.36) |
| Malaysia | male | 1019.35(956.86,1082.97) | 1805.49(1690.40,1923.66) | 1.85 | 23.98(22.51,25.53) | 19.99(19.07,20.94) | -0.64(-0.67,-0.60) |
| Maldives | male | 12.26(11.53,13.09) | 49.56(46.29,53.02) | 4.96 | 29.19(14.70,53.30) | 23.22(16.87,31.91) | -0.79(-0.84,-0.74) |
| Mali | male | 585.80(542.16,632.10) | 1292.21(1194.31,1400.50) | 2.80 | 36.01(33.10,39.13) | 29.67(28.00,31.41) | -0.70(-0.73,-0.67) |
| Malta | male | 259.08(243.82,275.09) | 256.15(241.12,271.36) | -0.12 | 261.40(230.22,295.91) | 239.90(210.70,272.75) | -0.30(-0.32,-0.29) |
| Marshall Islands | male | 4.24(3.96,4.51) | 5.95(5.57,6.34) | 1.03 | 45.76(12.23,133.07) | 38.99(14.19,87.12) | -0.53(-0.55,-0.51) |
| Mauritania | male | 154.63(145.90,164.77) | 273.74(257.72,291.60) | 1.92 | 36.79(31.04,43.40) | 31.01(27.35,35.06) | -0.57(-0.62,-0.53) |
| Mauritius | male | 78.44(73.31,83.55) | 78.63(73.16,83.97) | -0.14 | 26.83(21.12,33.79) | 22.90(18.09,28.68) | -0.57(-0.58,-0.55) |
| Mexico | male | 4738.37(4487.43,5015.74) | 6921.19(6557.70,7307.46) | 1.29 | 24.44(23.73,25.18) | 21.44(20.93,21.95) | -0.44(-0.46,-0.41) |
| Micronesia (Federated States of) | male | 9.53(8.91,10.15) | 9.81(9.15,10.46) | -0.06 | 43.24(19.76,85.98) | 37.58(17.77,70.49) | -0.48(-0.49,-0.46) |
| Monaco | male | 17.32(16.37,18.35) | 16.99(15.99,17.93) | 0.01 | 240.87(138.35,406.78) | 231.96(132.48,382.24) | -0.14(-0.15,-0.13) |
| Mongolia | male | 424.68(394.86,456.28) | 665.19(619.53,708.62) | 1.68 | 87.92(79.34,97.30) | 72.03(66.60,77.83) | -0.74(-0.80,-0.68) |
| Montenegro | male | 122.20(115.34,129.67) | 98.91(92.88,104.73) | -1.00 | 75.88(62.96,90.81) | 64.13(52.02,78.38) | -0.73(-0.83,-0.63) |
| Morocco | male | 4688.64(4379.07,5021.57) | 6197.77(5807.23,6602.87) | 0.93 | 80.14(77.80,82.54) | 64.57(62.97,66.20) | -0.75(-0.76,-0.74) |
| Mozambique | male | 781.57(733.10,835.81) | 1638.18(1540.59,1744.48) | 2.48 | 32.03(29.78,34.40) | 27.79(26.41,29.23) | -0.48(-0.52,-0.45) |
| Myanmar | male | 2972.07(2792.04,3163.28) | 3714.33(3478.06,3952.47) | 0.63 | 32.64(31.45,33.88) | 26.94(26.08,27.82) | -0.74(-0.78,-0.71) |
| Namibia | male | 116.96(109.66,125.27) | 198.34(184.64,212.12) | 1.78 | 38.78(31.79,47.02) | 33.32(28.79,38.41) | -0.52(-0.54,-0.50) |
| Nauru | male | 0.92(0.86,0.98) | 0.91(0.85,0.97) | -0.13 | 38.36(0.70,257.93) | 34.23(0.58,233.15) | -0.35(-0.44,-0.27) |
| Nepal | male | 1970.73(1857.70,2093.71) | 2572.48(2419.22,2732.05) | 0.79 | 47.34(45.25,49.52) | 36.44(35.02,37.90) | -1.00(-1.03,-0.96) |
| Netherlands | male | 10881.97(10263.43,11510.47) | 9524.11(8968.62,10058.60) | -0.57 | 257.69(252.85,262.60) | 244.54(239.59,249.57) | -0.19(-0.20,-0.18) |
| New Zealand | male | 1610.82(1528.93,1707.10) | 1638.74(1549.72,1729.62) | 0.06 | 178.92(170.28,187.88) | 167.46(159.39,175.84) | -0.24(-0.25,-0.23) |
| Nicaragua | male | 203.16(189.96,217.85) | 381.79(357.46,407.79) | 2.15 | 26.48(22.80,30.66) | 22.60(20.37,25.02) | -0.53(-0.56,-0.51) |
| Niger | male | 655.86(615.51,696.73) | 1633.83(1536.98,1748.36) | 3.08 | 42.64(39.37,46.13) | 38.21(36.27,40.25) | -0.37(-0.42,-0.32) |
| Nigeria | male | 9168.27(8699.89,9668.76) | 15380.31(14582.85,16280.12) | 1.74 | 45.35(44.42,46.30) | 34.03(33.48,34.59) | -1.05(-1.12,-0.97) |
| Niue | male | 0.21(0.19,0.22) | 0.14(0.13,0.15) | -1.64 | 40.98(0.00,893.23) | 35.22(0.00,1186.68) | -0.56(-0.58,-0.55) |
| North Macedonia | male | 424.03(399.69,447.10) | 386.74(363.90,410.07) | -0.41 | 79.95(72.51,87.98) | 65.97(59.43,73.12) | -0.79(-0.87,-0.71) |
| Northern Mariana Islands | male | 5.65(5.27,6.03) | 3.81(3.54,4.09) | -1.26 | 36.04(12.57,85.85) | 33.83(7.55,110.05) | -0.15(-0.19,-0.11) |
| Norway | male | 2170.21(2057.10,2276.79) | 2412.50(2292.52,2525.74) | 0.32 | 192.20(184.18,200.51) | 181.59(174.33,189.11) | -0.24(-0.26,-0.22) |
| Oman | male | 446.54(415.94,476.92) | 1143.27(1065.25,1228.29) | 3.70 | 71.57(64.90,78.84) | 49.90(46.74,53.30) | -1.23(-1.32,-1.14) |
| Pakistan | male | 12415.22(11740.76,13165.14) | 22967.52(21626.41,24436.89) | 2.10 | 50.66(49.75,51.59) | 42.56(42.00,43.13) | -0.63(-0.65,-0.61) |
| Palau | male | 1.56(1.46,1.67) | 1.97(1.83,2.10) | 0.23 | 37.14(2.77,173.42) | 32.77(3.47,149.12) | -0.42(-0.43,-0.40) |
| Palestine | male | 333.78(310.65,357.52) | 751.27(704.54,802.00) | 2.86 | 81.85(72.60,92.14) | 60.87(56.50,65.52) | -1.04(-1.06,-1.01) |
| Panama | male | 128.29(120.85,136.68) | 202.62(190.43,215.81) | 1.55 | 21.62(17.99,25.83) | 19.13(16.59,21.96) | -0.41(-0.42,-0.39) |
| Papua New Guinea | male | 459.32(429.80,490.17) | 1075.94(1005.96,1146.03) | 3.11 | 49.22(44.72,54.08) | 43.96(41.35,46.70) | -0.37(-0.39,-0.35) |
| Paraguay | male | 861.06(799.37,925.85) | 1659.25(1544.56,1783.71) | 2.31 | 94.50(88.18,101.18) | 89.32(85.06,93.76) | -0.17(-0.19,-0.15) |
| Peru | male | 4647.96(4319.25,5006.37) | 7192.40(6665.95,7701.39) | 1.50 | 93.21(90.49,96.00) | 81.16(79.30,83.06) | -0.46(-0.50,-0.43) |
| Philippines | male | 3890.52(3686.51,4102.05) | 7126.53(6740.33,7498.78) | 2.18 | 27.24(26.37,28.13) | 24.62(24.05,25.21) | -0.26(-0.32,-0.21) |
| Poland | male | 7867.69(7419.37,8300.69) | 7040.65(6732.75,7333.91) | -0.58 | 80.81(79.01,82.65) | 71.79(70.06,73.56) | -0.43(-0.47,-0.39) |
| Portugal | male | 6799.92(6415.26,7176.73) | 6124.38(5759.33,6492.75) | -0.40 | 276.82(270.28,283.48) | 253.64(247.11,260.31) | -0.30(-0.31,-0.28) |
| Puerto Rico | male | 467.21(435.96,499.40) | 377.65(353.09,404.09) | -0.76 | 53.41(48.67,58.50) | 47.85(43.11,52.99) | -0.39(-0.40,-0.39) |
| Qatar | male | 140.23(130.40,149.95) | 885.75(829.06,952.44) | 8.33 | 62.71(52.00,75.67) | 49.00(45.31,53.12) | -0.84(-0.87,-0.82) |
| Republic of Korea | male | 6161.32(5787.69,6570.61) | 6421.34(6035.94,6843.99) | 0.14 | 47.65(46.46,48.88) | 43.82(42.72,44.95) | -0.24(-0.29,-0.20) |
| Republic of Moldova | male | 826.45(775.83,875.13) | 643.31(604.86,681.31) | -1.06 | 76.96(71.76,82.46) | 65.55(60.44,71.05) | -0.61(-0.69,-0.53) |
| Romania | male | 4494.49(4228.49,4760.41) | 3042.02(2858.57,3226.95) | -1.50 | 78.55(76.26,80.90) | 64.26(61.90,66.70) | -0.80(-0.89,-0.71) |
| Russian Federation | male | 26912.95(25438.58,28454.54) | 22925.60(21737.39,24041.91) | -0.79 | 71.62(70.75,72.49) | 63.44(62.59,64.30) | -0.45(-0.48,-0.42) |
| Rwanda | male | 354.13(323.60,388.84) | 664.16(612.29,719.10) | 2.86 | 24.60(21.94,27.56) | 21.99(20.30,23.81) | -0.38(-0.41,-0.35) |
| Saint Kitts and Nevis | male | 5.31(4.93,5.70) | 8.21(7.69,8.75) | 1.64 | 56.94(17.72,154.78) | 50.23(21.75,101.22) | -0.41(-0.43,-0.39) |
| Saint Lucia | male | 18.32(17.12,19.67) | 25.32(23.66,26.97) | 1.24 | 60.15(35.00,98.74) | 52.86(34.21,78.50) | -0.41(-0.45,-0.37) |
| Saint Vincent and the Grenadines | male | 15.58(14.49,16.66) | 15.98(14.90,17.04) | 0.05 | 61.84(33.80,107.80) | 54.38(30.98,89.12) | -0.42(-0.45,-0.39) |
| Samoa | male | 14.85(13.89,15.85) | 19.77(18.58,21.06) | 0.95 | 41.99(22.52,73.14) | 39.35(23.81,61.64) | -0.19(-0.22,-0.16) |
| San Marino | male | 15.11(14.32,15.92) | 17.36(16.37,18.32) | 0.47 | 245.71(137.71,407.17) | 235.94(136.96,382.20) | -0.16(-0.18,-0.14) |
| Sao Tome and Principe | male | 7.60(7.14,8.06) | 14.59(13.73,15.47) | 2.31 | 33.68(13.14,74.84) | 27.86(15.34,47.21) | -0.69(-0.73,-0.65) |
| Saudi Arabia | male | 3546.30(3311.06,3791.63) | 7678.73(7205.54,8162.46) | 2.84 | 75.97(73.45,78.56) | 50.18(49.04,51.36) | -1.37(-1.45,-1.29) |
| Senegal | male | 553.00(518.93,585.63) | 1118.44(1046.87,1192.26) | 2.38 | 37.93(34.72,41.40) | 32.58(30.63,34.63) | -0.52(-0.56,-0.47) |
| Serbia | male | 1901.52(1793.15,2021.33) | 1394.29(1310.00,1476.30) | -1.52 | 80.37(76.79,84.09) | 65.87(62.41,69.48) | -0.81(-0.89,-0.73) |
| Seychelles | male | 4.17(3.92,4.44) | 6.67(6.25,7.11) | 1.81 | 23.91(6.46,65.74) | 20.91(8.04,47.24) | -0.43(-0.47,-0.39) |
| Sierra Leone | male | 282.51(266.62,300.09) | 632.10(593.85,671.26) | 3.37 | 37.75(33.41,42.55) | 32.69(30.12,35.44) | -0.50(-0.54,-0.46) |
| Singapore | male | 373.43(350.72,397.39) | 639.34(601.57,678.64) | 2.08 | 38.73(34.87,42.92) | 36.25(33.28,39.48) | -0.20(-0.23,-0.18) |
| Slovakia | male | 1090.51(1029.21,1154.15) | 930.23(873.33,986.29) | -0.74 | 80.40(75.68,85.35) | 64.86(60.59,69.40) | -0.82(-0.89,-0.75) |
| Slovenia | male | 399.41(375.57,423.70) | 315.78(296.80,334.62) | -0.94 | 75.87(68.57,83.77) | 63.01(55.92,70.91) | -0.73(-0.80,-0.66) |
| Solomon Islands | male | 34.20(31.92,36.57) | 68.73(64.05,73.31) | 2.46 | 50.36(34.39,71.79) | 44.05(34.19,55.96) | -0.42(-0.45,-0.40) |
| Somalia | male | 480.40(450.01,512.67) | 1441.96(1346.82,1538.72) | 3.79 | 31.91(29.05,35.00) | 31.03(29.39,32.75) | -0.05(-0.08,-0.02) |
| South Africa | male | 3275.86(3102.39,3466.44) | 5049.39(4792.80,5332.34) | 1.45 | 36.87(35.59,38.19) | 33.01(32.10,33.94) | -0.40(-0.42,-0.39) |
| South Sudan | male | 369.67(346.68,396.38) | 481.08(451.39,510.77) | 1.10 | 27.15(24.32,30.26) | 25.24(22.94,27.71) | -0.23(-0.26,-0.20) |
| Spain | male | 24171.97(22805.23,25670.66) | 25785.81(24223.68,27421.40) | 0.56 | 248.45(245.33,251.61) | 231.49(228.50,234.50) | -0.23(-0.25,-0.21) |
| Sri Lanka | male | 1924.49(1792.10,2058.37) | 1966.93(1825.88,2113.01) | 0.00 | 43.70(41.75,45.72) | 35.99(34.41,37.63) | -0.64(-0.66,-0.61) |
| Sudan | male | 3325.25(3116.70,3549.42) | 5871.36(5498.29,6268.76) | 1.82 | 79.82(77.05,82.67) | 60.43(58.86,62.04) | -0.96(-0.98,-0.95) |
| Suriname | male | 54.08(50.73,57.88) | 73.58(68.76,78.31) | 1.33 | 57.03(42.43,75.42) | 51.15(40.11,64.36) | -0.40(-0.42,-0.38) |
| Sweden | male | 4413.50(4169.17,4648.36) | 4663.34(4411.07,4923.73) | 0.20 | 198.86(192.96,204.90) | 195.45(189.81,201.24) | -0.08(-0.10,-0.06) |
| Switzerland | male | 4621.38(4364.90,4886.17) | 4955.74(4673.26,5256.13) | 0.30 | 239.34(232.41,246.44) | 231.27(224.72,237.99) | -0.13(-0.14,-0.13) |
| Syrian Arab Republic | male | 2054.09(1916.72,2194.60) | 1997.79(1867.64,2133.63) | 0.08 | 77.72(74.22,81.35) | 61.09(58.22,64.09) | -0.85(-0.89,-0.81) |
| Taiwan (Province of China) | male | 4341.07(4073.16,4614.12) | 3946.40(3690.34,4198.50) | -0.45 | 78.14(75.80,80.55) | 60.98(59.04,62.97) | -0.82(-0.84,-0.80) |
| Tajikistan | male | 975.60(906.73,1045.29) | 1810.00(1688.01,1936.48) | 2.26 | 86.57(80.88,92.62) | 74.15(70.73,77.71) | -0.59(-0.68,-0.50) |
| Thailand | male | 3855.13(3601.15,4097.31) | 4359.52(4078.64,4679.20) | 0.46 | 26.32(25.48,27.17) | 22.65(21.96,23.35) | -0.51(-0.54,-0.49) |
| Timor-Leste | male | 57.20(53.66,60.89) | 76.06(71.29,81.14) | 0.69 | 31.61(23.79,41.40) | 25.89(20.25,32.74) | -0.89(-1.00,-0.79) |
| Togo | male | 252.69(236.82,268.81) | 557.55(524.18,593.62) | 2.74 | 35.81(31.30,40.88) | 30.71(28.17,33.43) | -0.50(-0.55,-0.45) |
| Tokelau | male | 0.13(0.12,0.14) | 0.12(0.11,0.12) | -0.80 | 42.33(0.00,1756.36) | 35.48(0.00,1441.82) | -0.63(-0.65,-0.62) |
| Tonga | male | 8.41(7.89,9.00) | 8.74(8.18,9.28) | 0.34 | 44.50(18.78,91.31) | 38.95(17.38,76.05) | -0.42(-0.44,-0.40) |
| Trinidad and Tobago | male | 162.79(152.75,174.05) | 175.22(163.35,187.69) | 0.17 | 53.62(45.63,62.73) | 47.95(41.02,55.82) | -0.42(-0.45,-0.39) |
| Tunisia | male | 1433.65(1337.20,1537.79) | 1784.31(1671.05,1899.25) | 0.69 | 73.65(69.76,77.71) | 58.40(55.70,61.20) | -0.80(-0.84,-0.77) |
| Turkey | male | 14362.20(13433.11,15379.83) | 18710.61(17457.48,19931.19) | 0.82 | 98.21(96.58,99.86) | 78.29(77.17,79.42) | -0.77(-0.79,-0.76) |
| Turkmenistan | male | 662.82(618.95,708.24) | 905.98(844.82,967.05) | 0.99 | 80.73(74.33,87.63) | 64.93(60.76,69.32) | -0.85(-0.90,-0.79) |
| Tuvalu | male | 0.85(0.80,0.90) | 1.14(1.07,1.22) | 1.10 | 43.30(0.57,304.57) | 37.06(1.35,198.78) | -0.50(-0.52,-0.48) |
| Uganda | male | 948.54(887.39,1018.45) | 2041.40(1910.42,2176.28) | 2.63 | 28.22(26.35,30.20) | 24.04(22.95,25.18) | -0.63(-0.66,-0.59) |
| Ukraine | male | 9234.34(8696.04,9762.32) | 7095.59(6667.91,7511.60) | -1.04 | 74.29(72.78,75.83) | 64.76(63.20,66.34) | -0.52(-0.59,-0.46) |
| United Arab Emirates | male | 537.32(500.37,575.96) | 2811.06(2610.49,3021.82) | 7.15 | 60.91(55.51,66.84) | 46.65(44.11,49.36) | -0.93(-1.00,-0.86) |
| United Kingdom | male | 39011.30(37094.43,40807.39) | 39872.37(37971.76,41630.39) | 0.16 | 269.46(266.78,272.16) | 250.60(248.12,253.11) | -0.25(-0.27,-0.23) |
| United Republic of Tanzania | male | 1369.23(1252.04,1497.96) | 2924.23(2699.56,3179.59) | 2.63 | 27.43(25.93,29.01) | 24.25(23.36,25.17) | -0.38(-0.42,-0.35) |
| United States of America | male | 75029.89(71247.59,78715.53) | 74126.45(70506.63,77701.66) | -0.25 | 108.90(108.12,109.68) | 95.86(95.17,96.56) | -0.57(-0.65,-0.49) |
| United States Virgin Islands | male | 14.30(13.40,15.26) | 10.08(9.42,10.74) | -1.25 | 54.14(29.62,91.83) | 47.14(22.39,88.86) | -0.50(-0.52,-0.47) |
| Uruguay | male | 841.12(787.89,902.09) | 895.13(837.23,955.73) | 0.16 | 115.24(107.58,123.31) | 109.19(102.13,116.61) | -0.19(-0.21,-0.18) |
| Uzbekistan | male | 3958.41(3677.87,4244.62) | 6269.15(5867.43,6677.40) | 1.54 | 86.11(83.26,89.05) | 69.99(68.26,71.75) | -0.79(-0.85,-0.73) |
| Vanuatu | male | 15.71(14.68,16.75) | 28.97(27.11,30.87) | 2.24 | 48.62(27.31,81.03) | 42.78(28.54,61.94) | -0.44(-0.45,-0.43) |
| Venezuela (Bolivarian Republic of) | male | 1009.80(948.22,1071.07) | 1453.24(1364.92,1539.74) | 1.61 | 22.31(20.92,23.78) | 20.29(19.25,21.37) | -0.28(-0.32,-0.23) |
| Viet Nam | male | 4123.80(3864.75,4421.69) | 6779.34(6303.24,7215.86) | 1.73 | 29.81(28.84,30.80) | 24.38(23.80,24.98) | -0.71(-0.73,-0.69) |
| Yemen | male | 2132.44(1996.99,2279.59) | 4881.81(4558.23,5199.58) | 2.80 | 83.65(80.04,87.40) | 64.98(63.13,66.88) | -1.01(-1.07,-0.96) |
| Zambia | male | 428.43(399.32,458.25) | 1007.44(948.11,1070.82) | 3.06 | 27.11(24.46,30.01) | 23.73(22.25,25.29) | -0.46(-0.52,-0.41) |
| Zimbabwe | male | 818.11(762.19,879.57) | 1274.40(1190.86,1361.14) | 1.41 | 39.67(36.85,42.67) | 36.97(34.93,39.11) | -0.12(-0.19,-0.06) |

**Supplementary Table 2. The DALY of global psoriasis burden of young adults in 1990 and 2019, and its temporal trends from 1990 to 2019.**

|  |  | DALY No.(95%UI) |  |  | Age-standardized DALY rate (per 100000) No.95%UI) |  |  |
| --- | --- | --- | --- | --- | --- | --- | --- |
| nation | sex | 1990 | 2019 | 1990-2019 EAPC No.(95%CI) | 1990 | 2019 | 1990-2019 EAPC No.(95%CI) |
| Afghanistan | both | 2798.96(1954.64,3795.20) | 8840.95(6116.15,12007.61) | 3.57 | 64.19(61.69,66.78) | 52.47(51.34,53.63) | -0.75(-0.84,-0.65) |
| Albania | both | 895.48(610.58,1229.44) | 537.61(369.11,735.59) | -2.01 | 54.21(50.65,57.98) | 41.33(37.90,45.00) | -1.07(-1.13,-1.01) |
| Algeria | both | 5084.55(3496.80,6976.23) | 8286.22(5768.55,11182.01) | 1.74 | 46.78(45.43,48.15) | 36.19(35.41,36.98) | -0.87(-0.90,-0.83) |
| American Samoa | both | 4.83(3.30,6.64) | 4.97(3.38,6.85) | -0.12 | 21.19(6.47,54.10) | 18.60(5.89,46.07) | -0.41(-0.44,-0.37) |
| Andorra | both | 59.74(42.13,79.83) | 75.53(53.47,100.43) | 0.51 | 180.56(137.23,234.76) | 167.14(129.24,214.61) | -0.28(-0.29,-0.27) |
| Angola | both | 2269.32(1559.86,3104.72) | 4871.39(3360.52,6605.17) | 2.71 | 50.70(48.58,52.91) | 37.48(36.40,38.57) | -1.02(-1.08,-0.96) |
| Antigua and Barbuda | both | 10.71(7.47,14.52) | 15.40(10.72,21.08) | 1.29 | 35.17(17.15,65.90) | 31.53(17.73,52.43) | -0.37(-0.40,-0.35) |
| Argentina | both | 14619.64(10090.58,19596.52) | 19103.27(13173.87,25509.47) | 0.98 | 92.55(91.06,94.07) | 81.48(80.33,82.65) | -0.40(-0.42,-0.38) |
| Armenia | both | 853.12(596.97,1158.66) | 593.19(412.47,800.37) | -1.45 | 51.48(47.98,55.20) | 39.43(36.26,42.84) | -1.00(-1.05,-0.96) |
| Australia | both | 14102.50(9878.63,18738.21) | 16209.44(11361.51,21750.16) | 0.51 | 155.08(152.53,157.67) | 135.27(133.17,137.40) | -0.37(-0.45,-0.29) |
| Austria | both | 8282.33(5788.62,11210.65) | 7742.94(5554.35,10343.54) | -0.18 | 202.30(197.94,206.74) | 181.33(177.22,185.52) | -0.38(-0.40,-0.36) |
| Azerbaijan | both | 1703.81(1142.84,2302.09) | 2247.69(1545.17,3081.90) | 1.01 | 48.79(46.37,51.31) | 40.05(38.39,41.77) | -0.77(-0.86,-0.67) |
| Bahamas | both | 46.17(32.01,63.14) | 65.04(45.36,89.06) | 1.14 | 33.70(24.52,45.52) | 31.20(24.06,39.89) | -0.33(-0.37,-0.30) |
| Bahrain | both | 118.94(82.52,163.63) | 328.10(221.52,454.28) | 4.49 | 39.82(32.58,48.53) | 32.25(28.52,36.43) | -0.74(-0.77,-0.70) |
| Bangladesh | both | 14786.73(10026.44,20487.72) | 22027.62(14685.06,30433.05) | 1.43 | 31.14(30.62,31.66) | 25.93(25.58,26.27) | -0.56(-0.60,-0.52) |
| Barbados | both | 43.34(29.98,58.90) | 44.40(30.03,60.24) | 0.10 | 32.78(23.68,44.49) | 30.63(22.19,41.40) | -0.21(-0.23,-0.20) |
| Belarus | both | 2576.00(1803.12,3508.16) | 1833.64(1289.68,2480.34) | -1.31 | 51.08(49.11,53.11) | 40.39(38.48,42.38) | -0.85(-0.89,-0.81) |
| Belgium | both | 10558.98(7365.46,14135.30) | 9738.45(6882.50,13170.04) | -0.31 | 208.47(204.49,212.51) | 184.78(181.06,188.56) | -0.41(-0.42,-0.40) |
| Belize | both | 35.42(24.55,47.99) | 80.61(55.71,107.90) | 3.00 | 44.59(30.46,64.08) | 36.84(29.19,45.96) | -0.60(-0.65,-0.56) |
| Benin | both | 435.85(298.43,592.13) | 1079.43(747.76,1487.71) | 3.14 | 22.96(20.78,25.34) | 19.55(18.36,20.80) | -0.57(-0.64,-0.51) |
| Bermuda | both | 12.43(8.49,16.90) | 9.59(6.63,13.20) | -0.96 | 36.04(18.68,64.91) | 32.34(14.56,65.25) | -0.42(-0.44,-0.40) |
| Bhutan | both | 93.32(62.18,127.73) | 118.32(80.99,162.69) | 1.27 | 33.07(26.38,41.17) | 27.64(22.85,33.21) | -0.63(-0.64,-0.62) |
| Bolivia (Plurinational State of) | both | 2548.05(1783.89,3445.92) | 4472.41(3123.74,6018.09) | 2.08 | 89.05(85.55,92.66) | 74.47(72.30,76.70) | -0.55(-0.61,-0.49) |
| Bosnia and Herzegovina | both | 1333.05(929.01,1812.24) | 642.70(447.93,874.97) | -2.36 | 55.53(52.57,58.61) | 40.72(37.56,44.09) | -1.22(-1.30,-1.14) |
| Botswana | both | 136.63(92.05,189.88) | 257.33(174.78,357.32) | 2.07 | 24.12(20.07,28.85) | 19.56(17.23,22.14) | -0.75(-0.82,-0.67) |
| Brazil | both | 52137.93(36980.14,70010.94) | 78703.51(55270.43,104757.46) | 1.45 | 70.47(69.85,71.08) | 67.03(66.56,67.51) | -0.16(-0.17,-0.14) |
| Brunei Darussalam | both | 31.48(21.69,43.44) | 55.63(38.47,76.71) | 1.91 | 22.25(14.95,32.53) | 20.51(15.45,26.81) | -0.28(-0.30,-0.26) |
| Bulgaria | both | 2081.41(1460.73,2832.18) | 1246.00(860.92,1677.66) | -1.78 | 48.89(46.80,51.06) | 38.69(36.45,41.05) | -0.81(-0.84,-0.77) |
| Burkina Faso | both | 924.02(640.81,1274.99) | 2176.80(1439.41,3004.08) | 3.04 | 25.38(23.73,27.14) | 22.32(21.37,23.31) | -0.49(-0.55,-0.43) |
| Burundi | both | 370.84(238.53,513.90) | 837.67(563.06,1161.88) | 3.10 | 16.15(14.48,17.99) | 16.04(14.94,17.21) | -0.05(-0.08,-0.03) |
| Cabo Verde | both | 29.95(20.92,41.53) | 52.77(35.74,72.56) | 1.98 | 22.16(14.30,33.59) | 17.37(12.97,22.88) | -0.90(-0.98,-0.83) |
| Cambodia | both | 796.01(549.81,1107.43) | 1331.74(896.54,1841.73) | 1.81 | 18.62(17.30,20.02) | 15.58(14.75,16.45) | -0.65(-0.67,-0.63) |
| Cameroon | both | 857.44(576.98,1176.50) | 2277.43(1558.60,3123.22) | 3.49 | 20.01(18.65,21.45) | 16.66(15.96,17.39) | -0.62(-0.68,-0.56) |
| Canada | both | 15490.01(10797.46,20926.96) | 15243.00(10744.85,20262.66) | -0.19 | 102.24(100.62,103.87) | 90.11(88.66,91.57) | -0.45(-0.46,-0.44) |
| Central African Republic | both | 596.47(416.19,807.17) | 1081.16(742.58,1447.07) | 2.07 | 49.95(45.90,54.31) | 43.96(41.32,46.74) | -0.41(-0.43,-0.40) |
| Chad | both | 631.04(431.90,878.07) | 1486.16(1016.75,2043.61) | 2.91 | 26.76(24.65,29.03) | 23.20(21.98,24.48) | -0.54(-0.59,-0.49) |
| Chile | both | 6415.79(4462.93,8665.29) | 7373.24(5148.09,9908.73) | 0.49 | 92.90(90.63,95.23) | 78.32(76.54,80.15) | -0.58(-0.60,-0.55) |
| China | both | 322404.46(227210.82,432328.97) | 299176.04(210611.01,397239.54) | -0.13 | 51.09(50.91,51.27) | 38.29(38.15,38.43) | -1.00(-1.01,-0.98) |
| Colombia | both | 2061.50(1365.48,2854.36) | 2753.61(1845.60,3788.79) | 0.93 | 12.69(12.14,13.27) | 11.06(10.65,11.48) | -0.48(-0.50,-0.46) |
| Comoros | both | 30.50(20.60,42.35) | 53.01(35.65,72.79) | 1.95 | 15.86(10.54,23.17) | 14.49(10.84,19.03) | -0.28(-0.30,-0.26) |
| Congo | both | 402.06(276.02,543.58) | 777.39(526.79,1069.53) | 2.32 | 38.51(34.65,42.73) | 29.41(27.38,31.57) | -0.91(-0.95,-0.86) |
| Cook Islands | both | 1.89(1.27,2.62) | 1.52(1.01,2.08) | -0.64 | 21.10(2.21,89.35) | 18.22(1.31,82.33) | -0.49(-0.50,-0.48) |
| Costa Rica | both | 184.85(123.54,263.65) | 274.08(185.61,379.07) | 1.32 | 12.49(10.71,14.51) | 10.92(9.67,12.31) | -0.44(-0.47,-0.42) |
| Croatia | both | 1175.36(823.29,1579.53) | 738.60(505.56,999.76) | -1.74 | 47.37(44.68,50.18) | 38.03(35.26,40.98) | -0.84(-0.87,-0.80) |
| Cuba | both | 2237.75(1507.65,3041.34) | 1956.43(1375.09,2629.98) | -0.34 | 37.41(35.86,39.01) | 35.31(33.72,36.96) | -0.20(-0.24,-0.16) |
| Cyprus | both | 822.06(589.23,1113.62) | 1219.92(860.51,1638.08) | 1.46 | 202.33(188.72,216.70) | 166.79(157.26,176.86) | -0.67(-0.73,-0.62) |
| Czechia | both | 2594.93(1817.57,3479.87) | 1936.54(1352.85,2633.88) | -1.02 | 48.52(46.64,50.45) | 37.76(35.98,39.61) | -0.81(-0.87,-0.76) |
| Côte d'Ivoire | both | 1123.28(756.48,1537.23) | 2307.70(1616.54,3174.08) | 2.42 | 21.33(20.05,22.68) | 18.28(17.53,19.06) | -0.50(-0.58,-0.43) |
| Democratic People's Republic of Korea | both | 6652.98(4634.41,9103.83) | 7472.03(5224.12,10114.27) | 0.61 | 63.45(61.93,65.00) | 51.33(50.16,52.52) | -0.75(-0.78,-0.73) |
| Democratic Republic of the Congo | both | 7146.37(4974.42,9728.11) | 15313.13(10446.87,20988.37) | 2.78 | 44.96(43.88,46.06) | 38.38(37.76,39.02) | -0.43(-0.59,-0.28) |
| Denmark | both | 5604.10(3988.62,7578.18) | 4881.88(3411.37,6570.46) | -0.50 | 203.11(197.78,208.55) | 183.63(178.45,188.93) | -0.34(-0.36,-0.32) |
| Djibouti | both | 35.03(23.83,49.45) | 91.25(61.47,128.72) | 3.70 | 16.03(10.92,23.09) | 14.26(11.48,17.55) | -0.44(-0.47,-0.42) |
| Dominica | both | 12.22(8.35,16.56) | 10.80(7.57,14.71) | -0.40 | 36.30(18.56,65.25) | 31.68(15.67,57.44) | -0.51(-0.55,-0.46) |
| Dominican Republic | both | 1507.50(1050.17,2069.45) | 2140.63(1494.64,2911.03) | 1.09 | 43.86(41.59,46.23) | 37.43(35.86,39.05) | -0.65(-0.72,-0.59) |
| Ecuador | both | 3250.43(2249.57,4453.11) | 5160.72(3609.71,7026.37) | 1.59 | 68.82(66.42,71.30) | 56.96(55.41,58.54) | -0.63(-0.65,-0.60) |
| Egypt | both | 6534.51(4400.37,8935.93) | 10139.74(6904.50,13696.73) | 1.75 | 25.47(24.85,26.10) | 20.09(19.70,20.49) | -0.62(-0.67,-0.56) |
| El Salvador | both | 342.27(233.06,475.69) | 399.40(269.90,547.17) | 0.47 | 14.52(12.98,16.21) | 12.41(11.22,13.70) | -0.58(-0.62,-0.55) |
| Equatorial Guinea | both | 86.15(59.15,117.55) | 189.69(129.04,263.42) | 2.62 | 50.14(39.83,62.63) | 26.93(23.00,31.47) | -2.40(-2.50,-2.30) |
| Eritrea | both | 227.84(153.78,314.78) | 519.35(347.76,709.57) | 3.04 | 17.74(15.42,20.34) | 15.71(14.36,17.17) | -0.36(-0.43,-0.29) |
| Estonia | both | 371.72(260.31,509.48) | 232.74(160.93,314.72) | -1.55 | 48.35(43.54,53.57) | 38.32(33.37,43.90) | -0.79(-0.82,-0.77) |
| Eswatini | both | 86.51(57.85,121.25) | 125.54(88.20,172.40) | 1.09 | 25.90(20.51,32.43) | 21.46(17.79,25.76) | -0.66(-0.72,-0.59) |
| Ethiopia | both | 3670.56(2574.17,4960.78) | 7728.36(5460.39,10357.04) | 2.50 | 17.55(16.97,18.15) | 15.59(15.23,15.95) | -0.39(-0.43,-0.35) |
| Fiji | both | 89.23(62.54,122.11) | 95.52(65.36,131.02) | 0.29 | 23.71(18.99,29.34) | 20.48(16.58,25.03) | -0.49(-0.53,-0.46) |
| Finland | both | 6156.27(4363.38,8211.85) | 4942.99(3515.88,6567.04) | -0.88 | 226.63(220.90,232.49) | 202.81(197.13,208.63) | -0.39(-0.40,-0.38) |
| France | both | 76003.53(53697.08,101846.40) | 67927.52(47466.75,91296.77) | -0.48 | 257.00(255.17,258.84) | 228.64(226.90,230.39) | -0.42(-0.45,-0.40) |
| Gabon | both | 157.01(107.64,215.52) | 242.57(170.01,328.17) | 1.54 | 37.09(31.27,43.85) | 26.86(23.56,30.52) | -1.05(-1.08,-1.02) |
| Gambia | both | 100.07(69.36,136.47) | 205.44(136.34,285.95) | 2.33 | 23.93(19.28,29.53) | 19.49(16.82,22.52) | -0.74(-0.81,-0.67) |
| Georgia | both | 1234.91(870.24,1667.79) | 682.02(474.62,940.68) | -2.14 | 46.47(43.90,49.15) | 39.97(36.97,43.17) | -0.52(-0.59,-0.45) |
| Germany | both | 60896.34(43044.15,81753.66) | 52255.89(36572.09,69422.68) | -0.65 | 148.27(147.08,149.47) | 138.17(136.96,139.38) | -0.29(-0.34,-0.24) |
| Ghana | both | 1662.01(1134.90,2281.28) | 3413.68(2314.28,4725.54) | 2.48 | 25.54(24.29,26.84) | 21.27(20.55,22.00) | -0.69(-0.73,-0.64) |
| Greece | both | 10519.91(7374.42,13990.34) | 8748.87(6176.79,11683.89) | -0.64 | 205.50(201.59,209.48) | 180.52(176.58,184.53) | -0.44(-0.48,-0.40) |
| Greenland | both | 42.28(29.96,56.52) | 29.84(21.27,40.23) | -1.10 | 126.55(90.70,173.92) | 110.73(74.43,159.87) | -0.53(-0.56,-0.51) |
| Grenada | both | 14.84(10.07,20.60) | 17.94(12.75,24.39) | 0.75 | 40.06(21.76,69.71) | 33.17(19.56,53.17) | -0.55(-0.63,-0.47) |
| Guam | both | 14.08(9.48,19.20) | 13.63(9.26,18.89) | -0.27 | 19.08(10.30,33.28) | 16.69(9.01,28.51) | -0.51(-0.56,-0.46) |
| Guatemala | both | 530.51(355.21,739.24) | 1225.53(817.90,1716.88) | 3.03 | 16.12(14.74,17.61) | 13.34(12.58,14.13) | -0.66(-0.70,-0.62) |
| Guinea | both | 610.54(409.37,828.13) | 1130.75(747.92,1552.97) | 2.00 | 24.21(22.29,26.26) | 20.83(19.59,22.13) | -0.52(-0.59,-0.45) |
| Guinea-Bissau | both | 100.97(69.32,137.91) | 183.31(122.00,253.12) | 2.08 | 24.20(19.54,29.78) | 20.41(17.45,23.79) | -0.61(-0.68,-0.55) |
| Guyana | both | 141.20(97.06,190.61) | 132.56(91.70,182.38) | -0.35 | 36.98(30.94,44.01) | 32.57(27.22,38.69) | -0.40(-0.43,-0.38) |
| Haiti | both | 1398.63(989.52,1886.57) | 2861.08(2005.76,3892.91) | 2.52 | 49.80(47.17,52.55) | 44.74(43.10,46.44) | -0.38(-0.40,-0.36) |
| Honduras | both | 307.44(207.30,419.83) | 684.72(459.87,949.60) | 2.79 | 15.93(14.14,17.91) | 13.75(12.72,14.84) | -0.53(-0.57,-0.49) |
| Hungary | both | 2568.56(1791.48,3473.22) | 1820.13(1235.38,2454.35) | -1.29 | 49.19(47.28,51.16) | 38.71(36.86,40.63) | -0.83(-0.87,-0.78) |
| Iceland | both | 298.51(209.37,401.14) | 336.34(237.08,453.96) | 0.34 | 226.57(201.52,254.02) | 200.04(179.10,222.96) | -0.44(-0.46,-0.43) |
| India | both | 132054.33(92813.62,175836.98) | 240046.71(168582.32,320468.08) | 2.12 | 32.46(32.28,32.64) | 31.98(31.85,32.11) | -0.06(-0.14,0.02) |
| Indonesia | both | 7201.71(5004.88,9858.86) | 10551.78(7333.04,14484.62) | 1.33 | 8.07(7.88,8.26) | 7.26(7.12,7.40) | -0.34(-0.37,-0.32) |
| Iran (Islamic Republic of) | both | 12191.74(8628.16,16316.30) | 17797.53(12504.85,24026.47) | 1.47 | 49.76(48.84,50.70) | 36.04(35.51,36.59) | -1.18(-1.25,-1.11) |
| Iraq | both | 4240.07(2963.50,5763.02) | 9428.11(6420.63,13017.10) | 2.57 | 58.26(56.46,60.11) | 42.43(41.56,43.30) | -1.12(-1.18,-1.06) |
| Ireland | both | 3801.31(2687.58,5072.00) | 4541.73(3209.09,6068.82) | 0.85 | 215.79(208.97,222.78) | 184.04(178.59,189.62) | -0.53(-0.56,-0.50) |
| Israel | both | 4476.56(3130.69,5997.05) | 7482.64(5178.56,9931.65) | 1.67 | 188.04(182.52,193.69) | 168.79(164.98,172.67) | -0.34(-0.36,-0.32) |
| Italy | both | 53431.95(37408.96,71573.36) | 47013.62(33214.64,62701.40) | -0.14 | 183.99(182.43,185.56) | 170.91(169.29,172.54) | -0.15(-0.23,-0.07) |
| Jamaica | both | 429.91(290.65,585.05) | 512.63(356.83,697.51) | 0.61 | 39.06(35.33,43.12) | 33.77(30.90,36.84) | -0.50(-0.52,-0.48) |
| Japan | both | 16733.61(11855.83,22498.36) | 13824.29(9925.61,18507.41) | -0.58 | 24.89(24.51,25.28) | 24.78(24.35,25.22) | 0.04(0.03,0.06) |
| Jordan | both | 707.55(494.48,960.70) | 2098.92(1417.17,2867.44) | 3.84 | 43.71(40.34,47.32) | 33.89(32.45,35.38) | -0.92(-0.94,-0.90) |
| Kazakhstan | both | 4127.54(2855.17,5573.20) | 3847.42(2607.38,5182.85) | -0.10 | 51.49(49.90,53.12) | 40.84(39.54,42.16) | -0.79(-0.81,-0.77) |
| Kenya | both | 1351.05(946.89,1844.00) | 3262.68(2292.63,4385.44) | 3.10 | 14.30(13.50,15.14) | 13.05(12.60,13.52) | -0.25(-0.29,-0.21) |
| Kiribati | both | 8.10(5.66,11.21) | 13.18(8.96,18.11) | 2.01 | 23.60(10.00,48.89) | 22.63(12.03,39.47) | -0.01(-0.06,0.03) |
| Kuwait | both | 372.74(261.99,509.22) | 921.69(635.18,1258.40) | 3.61 | 35.67(32.02,39.68) | 29.37(27.39,31.49) | -0.83(-0.88,-0.77) |
| Kyrgyzstan | both | 1074.59(745.04,1465.30) | 1509.58(1031.87,2063.39) | 1.28 | 53.76(50.44,57.27) | 45.51(43.23,47.88) | -0.51(-0.53,-0.48) |
| Lao People's Democratic Republic | both | 339.00(230.56,471.24) | 606.67(399.28,836.88) | 2.05 | 19.41(17.35,21.67) | 16.09(14.83,17.44) | -0.68(-0.71,-0.65) |
| Latvia | both | 617.09(430.16,825.76) | 327.59(227.81,444.02) | -2.10 | 47.34(43.66,51.26) | 38.27(34.07,42.91) | -0.72(-0.75,-0.68) |
| Lebanon | both | 673.40(475.94,909.25) | 947.26(666.29,1275.14) | 1.30 | 46.51(43.03,50.21) | 34.57(32.38,36.88) | -1.08(-1.12,-1.04) |
| Lesotho | both | 228.35(157.38,313.56) | 267.61(184.97,365.95) | 0.38 | 29.09(25.33,33.31) | 24.01(21.17,27.17) | -0.65(-0.70,-0.59) |
| Liberia | both | 170.94(114.09,236.32) | 452.94(313.24,613.11) | 3.41 | 21.63(18.46,25.23) | 18.91(17.19,20.76) | -0.65(-0.70,-0.59) |
| Libya | both | 783.67(540.03,1066.57) | 1360.62(932.20,1883.67) | 2.28 | 43.65(40.53,46.97) | 32.26(30.56,34.04) | -1.01(-1.12,-0.90) |
| Lithuania | both | 866.71(610.84,1175.28) | 465.46(327.78,631.91) | -2.18 | 47.19(44.09,50.45) | 37.08(33.71,40.74) | -0.86(-0.88,-0.83) |
| Luxembourg | both | 393.47(274.64,531.07) | 573.91(405.27,775.49) | 1.26 | 190.91(172.28,211.29) | 175.86(161.40,191.45) | -0.29(-0.30,-0.29) |
| Madagascar | both | 858.01(584.53,1175.75) | 2035.32(1349.85,2816.34) | 3.05 | 17.18(16.00,18.44) | 16.12(15.41,16.85) | -0.20(-0.23,-0.16) |
| Malawi | both | 708.39(472.37,987.14) | 1354.88(887.40,1900.66) | 2.22 | 17.59(16.27,19.01) | 15.95(15.08,16.86) | -0.36(-0.39,-0.33) |
| Malaysia | both | 1121.90(736.33,1553.14) | 1881.77(1253.23,2708.31) | 1.71 | 13.05(12.28,13.86) | 10.90(10.41,11.41) | -0.61(-0.65,-0.57) |
| Maldives | both | 14.29(9.67,19.89) | 41.19(27.98,58.07) | 3.81 | 16.43(8.70,29.03) | 12.76(9.03,17.84) | -0.86(-0.91,-0.82) |
| Mali | both | 740.02(495.62,1013.59) | 1578.98(1073.85,2205.98) | 2.68 | 21.41(19.86,23.05) | 17.47(16.58,18.40) | -0.75(-0.78,-0.72) |
| Malta | both | 407.00(284.63,542.75) | 366.39(259.35,488.03) | -0.46 | 204.94(185.29,226.27) | 174.98(157.07,194.72) | -0.52(-0.54,-0.50) |
| Marshall Islands | both | 4.75(3.24,6.65) | 6.45(4.38,9.02) | 0.94 | 25.88(7.48,72.74) | 21.64(8.28,46.80) | -0.57(-0.59,-0.55) |
| Mauritania | both | 194.15(132.50,262.01) | 346.02(231.62,480.53) | 1.94 | 22.44(19.30,26.01) | 18.75(16.77,20.90) | -0.62(-0.68,-0.55) |
| Mauritius | both | 86.66(58.27,117.96) | 84.97(56.92,117.07) | -0.21 | 14.77(11.78,18.37) | 12.66(10.09,15.72) | -0.56(-0.58,-0.54) |
| Mexico | both | 5734.42(4076.29,7682.93) | 8330.18(5851.53,11130.20) | 1.27 | 14.26(13.88,14.65) | 12.53(12.27,12.81) | -0.44(-0.46,-0.41) |
| Micronesia (Federated States of) | both | 10.45(7.28,14.30) | 10.65(7.16,14.52) | -0.09 | 23.98(11.36,46.56) | 20.61(10.09,37.77) | -0.49(-0.51,-0.48) |
| Monaco | both | 26.70(18.56,35.88) | 25.30(17.58,33.98) | -0.02 | 180.49(116.50,275.57) | 165.59(105.44,250.75) | -0.28(-0.29,-0.28) |
| Mongolia | both | 562.11(385.66,764.98) | 820.87(566.70,1121.65) | 1.52 | 57.93(52.98,63.28) | 44.72(41.67,47.95) | -0.87(-0.89,-0.85) |
| Montenegro | both | 144.45(99.12,195.21) | 114.01(77.78,157.71) | -1.03 | 45.70(38.53,53.86) | 37.43(30.80,45.16) | -0.81(-0.87,-0.74) |
| Morocco | both | 6349.99(4378.38,8566.52) | 8080.72(5692.62,11067.21) | 0.80 | 54.06(52.70,55.44) | 41.97(41.06,42.90) | -0.88(-0.90,-0.87) |
| Mozambique | both | 1062.24(719.73,1453.55) | 2171.95(1451.71,2976.03) | 2.36 | 19.75(18.56,21.00) | 17.23(16.49,18.01) | -0.49(-0.52,-0.46) |
| Myanmar | both | 3589.78(2381.14,4907.94) | 4486.21(3056.62,6219.05) | 0.64 | 18.96(18.33,19.61) | 15.54(15.08,16.00) | -0.77(-0.81,-0.73) |
| Namibia | both | 147.00(101.26,205.06) | 246.40(168.29,337.78) | 1.70 | 23.72(19.88,28.17) | 20.18(17.71,22.92) | -0.56(-0.60,-0.53) |
| Nauru | both | 0.98(0.65,1.34) | 0.99(0.66,1.35) | -0.05 | 20.65(0.45,134.61) | 18.36(0.42,117.59) | -0.33(-0.43,-0.22) |
| Nepal | both | 2509.23(1725.19,3481.17) | 3416.83(2368.38,4684.36) | 0.94 | 29.45(28.29,30.65) | 21.85(21.12,22.61) | -1.16(-1.22,-1.10) |
| Netherlands | both | 16634.16(11731.80,22246.06) | 14148.43(9957.46,19157.10) | -0.61 | 201.28(198.22,204.38) | 180.84(177.83,183.90) | -0.34(-0.36,-0.33) |
| New Zealand | both | 3128.29(2193.09,4235.55) | 3165.89(2203.97,4262.95) | 0.09 | 173.18(167.16,179.36) | 155.14(149.72,160.71) | -0.36(-0.38,-0.35) |
| Nicaragua | both | 245.97(157.20,354.91) | 448.87(307.61,621.02) | 2.05 | 15.29(13.35,17.47) | 13.08(11.89,14.36) | -0.52(-0.56,-0.49) |
| Niger | both | 886.50(606.16,1210.71) | 2259.31(1496.73,3169.06) | 3.19 | 27.88(26.03,29.85) | 25.66(24.55,26.82) | -0.28(-0.35,-0.22) |
| Nigeria | both | 11948.48(8392.49,16097.78) | 20724.45(14615.88,27688.54) | 1.82 | 30.56(30.00,31.13) | 21.60(21.30,21.90) | -1.28(-1.38,-1.18) |
| Niue | both | 0.22(0.15,0.31) | 0.15(0.10,0.20) | -1.72 | 22.27(0.00,453.59) | 19.10(0.00,595.31) | -0.57(-0.59,-0.55) |
| North Macedonia | both | 519.06(360.65,700.27) | 439.22(304.25,600.39) | -0.61 | 49.77(45.58,54.26) | 39.20(35.54,43.17) | -0.89(-0.93,-0.85) |
| Northern Mariana Islands | both | 5.51(3.79,7.54) | 3.85(2.66,5.38) | -1.25 | 19.40(6.61,46.16) | 18.28(4.22,59.33) | -0.12(-0.17,-0.07) |
| Norway | both | 3478.31(2454.29,4657.88) | 3780.28(2666.83,5020.35) | 0.27 | 157.45(152.24,162.80) | 145.13(140.47,149.91) | -0.29(-0.31,-0.28) |
| Oman | both | 428.23(295.73,580.24) | 948.23(645.46,1306.92) | 3.03 | 45.76(41.41,50.49) | 29.47(27.50,31.58) | -1.49(-1.60,-1.38) |
| Pakistan | both | 15404.72(10968.25,20910.11) | 29380.20(20646.12,39832.60) | 2.22 | 32.75(32.22,33.29) | 27.43(27.11,27.75) | -0.65(-0.68,-0.62) |
| Palau | both | 1.62(1.10,2.20) | 1.76(1.18,2.41) | -0.18 | 19.71(1.58,90.11) | 17.31(1.47,80.23) | -0.41(-0.43,-0.39) |
| Palestine | both | 444.43(313.51,602.58) | 925.69(652.23,1273.78) | 2.65 | 55.40(50.04,61.24) | 38.50(36.01,41.13) | -1.27(-1.31,-1.24) |
| Panama | both | 144.07(97.84,199.39) | 226.30(153.63,309.32) | 1.56 | 12.18(10.24,14.41) | 10.77(9.41,12.27) | -0.39(-0.41,-0.38) |
| Papua New Guinea | both | 519.58(364.02,709.02) | 1235.16(832.43,1690.01) | 3.17 | 28.13(25.70,30.75) | 25.27(23.87,26.74) | -0.33(-0.36,-0.31) |
| Paraguay | both | 1349.09(939.93,1820.76) | 2608.74(1831.62,3544.21) | 2.30 | 74.49(70.49,78.67) | 71.46(68.73,74.28) | -0.14(-0.16,-0.11) |
| Peru | both | 7545.97(5303.53,10308.41) | 11186.04(7738.38,15042.27) | 1.36 | 74.05(72.34,75.78) | 62.71(61.56,63.89) | -0.56(-0.61,-0.51) |
| Philippines | both | 4439.36(3153.01,5989.82) | 7964.67(5572.12,10790.73) | 2.15 | 15.17(14.71,15.63) | 13.93(13.63,14.25) | -0.17(-0.24,-0.10) |
| Poland | both | 9929.62(7074.25,13230.36) | 8847.39(6233.07,11930.40) | -0.50 | 51.83(50.80,52.87) | 46.55(45.55,47.58) | -0.28(-0.33,-0.23) |
| Portugal | both | 11098.66(7806.86,14826.35) | 9633.03(6772.39,12988.21) | -0.49 | 221.83(217.72,226.00) | 190.92(186.97,194.94) | -0.50(-0.52,-0.48) |
| Puerto Rico | both | 600.92(408.36,810.63) | 476.15(323.45,642.35) | -0.86 | 32.77(30.20,35.50) | 29.20(26.61,31.98) | -0.43(-0.44,-0.42) |
| Qatar | both | 113.37(78.19,156.46) | 645.47(440.49,894.30) | 7.64 | 37.77(30.78,46.16) | 28.43(26.03,31.11) | -0.97(-1.00,-0.94) |
| Republic of Korea | both | 6998.33(4729.39,9631.10) | 6730.71(4511.51,9338.25) | -0.10 | 27.76(27.11,28.43) | 24.15(23.55,24.76) | -0.43(-0.48,-0.38) |
| Republic of Moldova | both | 1077.49(750.91,1447.99) | 774.25(540.42,1070.79) | -1.26 | 49.16(46.24,52.21) | 40.71(37.80,43.82) | -0.65(-0.72,-0.58) |
| Romania | both | 5352.57(3712.64,7237.50) | 3365.27(2347.71,4513.27) | -1.70 | 47.03(45.77,48.31) | 36.67(35.39,38.00) | -0.89(-0.93,-0.84) |
| Russian Federation | both | 32458.87(23101.21,43454.69) | 27559.90(19363.05,36776.43) | -0.73 | 43.66(43.18,44.14) | 38.67(38.19,39.15) | -0.39(-0.43,-0.35) |
| Rwanda | both | 416.31(277.18,581.60) | 788.44(524.59,1111.97) | 2.84 | 14.08(12.68,15.61) | 12.72(11.82,13.67) | -0.36(-0.39,-0.33) |
| Saint Kitts and Nevis | both | 6.70(4.59,9.15) | 10.05(6.78,13.62) | 1.51 | 35.78(13.04,86.91) | 31.09(14.83,58.47) | -0.47(-0.49,-0.44) |
| Saint Lucia | both | 24.13(16.93,32.85) | 31.93(21.95,43.30) | 1.08 | 38.45(24.19,59.12) | 33.32(22.71,47.44) | -0.46(-0.51,-0.41) |
| Saint Vincent and the Grenadines | both | 20.01(13.73,27.25) | 20.03(13.72,27.42) | 0.00 | 40.08(23.75,65.16) | 34.56(21.08,53.69) | -0.48(-0.51,-0.45) |
| Samoa | both | 16.26(11.12,22.38) | 21.87(15.01,30.02) | 1.01 | 23.13(12.85,39.19) | 22.08(13.72,33.86) | -0.09(-0.12,-0.05) |
| San Marino | both | 22.43(15.65,30.12) | 27.03(19.09,36.12) | 0.73 | 186.66(117.45,282.77) | 169.89(110.84,251.49) | -0.32(-0.34,-0.30) |
| Sao Tome and Principe | both | 9.18(6.27,12.32) | 16.72(11.41,23.23) | 2.05 | 19.76(8.62,40.38) | 16.14(9.28,26.46) | -0.78(-0.82,-0.73) |
| Saudi Arabia | both | 3858.32(2641.06,5196.31) | 7554.22(5320.70,10282.57) | 2.54 | 50.16(48.56,51.82) | 29.75(29.07,30.44) | -1.70(-1.81,-1.58) |
| Senegal | both | 715.33(491.38,986.05) | 1401.57(923.87,1929.18) | 2.26 | 23.28(21.54,25.15) | 20.08(19.02,21.20) | -0.52(-0.59,-0.46) |
| Serbia | both | 2327.33(1628.90,3177.10) | 1601.79(1101.16,2142.78) | -1.66 | 49.98(47.96,52.07) | 38.74(36.84,40.72) | -0.94(-0.98,-0.90) |
| Seychelles | both | 4.52(3.06,6.33) | 6.44(4.30,9.01) | 1.40 | 13.02(3.72,34.71) | 11.38(4.29,25.56) | -0.42(-0.46,-0.38) |
| Sierra Leone | both | 362.41(241.21,495.18) | 789.98(539.11,1121.31) | 3.15 | 23.00(20.65,25.58) | 20.04(18.62,21.55) | -0.51(-0.55,-0.46) |
| Singapore | both | 403.98(268.52,545.56) | 645.87(444.17,880.77) | 1.79 | 21.35(19.31,23.57) | 19.33(17.76,21.03) | -0.30(-0.33,-0.27) |
| Slovakia | both | 1345.29(942.04,1828.66) | 1051.06(739.79,1425.84) | -0.96 | 50.05(47.40,52.82) | 38.09(35.72,40.60) | -0.93(-0.98,-0.88) |
| Slovenia | both | 468.47(322.45,632.89) | 345.99(239.96,468.63) | -1.11 | 45.65(41.59,50.02) | 36.45(32.52,40.80) | -0.80(-0.85,-0.76) |
| Solomon Islands | both | 40.00(27.18,54.62) | 80.55(55.83,106.71) | 2.47 | 29.16(20.49,40.62) | 25.50(20.20,31.82) | -0.41(-0.44,-0.38) |
| Somalia | both | 589.82(390.72,831.87) | 1838.01(1257.07,2530.97) | 3.87 | 19.71(18.12,21.41) | 20.49(19.54,21.49) | 0.14(0.11,0.16) |
| South Africa | both | 4088.84(2867.84,5499.79) | 6045.06(4266.37,8029.97) | 1.26 | 22.38(21.68,23.10) | 19.85(19.35,20.36) | -0.46(-0.49,-0.43) |
| South Sudan | both | 403.16(272.34,559.93) | 600.66(404.14,828.48) | 1.56 | 15.73(14.16,17.46) | 14.97(13.76,16.26) | -0.17(-0.20,-0.15) |
| Spain | both | 37919.67(26379.42,50783.02) | 39015.97(27184.53,52367.57) | 0.44 | 196.25(194.27,198.23) | 172.52(170.71,174.35) | -0.41(-0.43,-0.39) |
| Sri Lanka | both | 2507.22(1704.68,3445.44) | 2503.50(1757.77,3416.52) | -0.07 | 28.13(27.03,29.27) | 22.46(21.59,23.36) | -0.74(-0.77,-0.71) |
| Sudan | both | 4577.15(3160.03,6190.01) | 7577.21(5247.27,10286.19) | 1.63 | 53.77(52.18,55.41) | 38.39(37.51,39.28) | -1.17(-1.19,-1.15) |
| Suriname | both | 67.25(46.70,92.77) | 92.59(63.23,126.31) | 1.36 | 35.79(27.58,45.84) | 31.81(25.65,39.04) | -0.45(-0.47,-0.42) |
| Sweden | both | 5966.08(4209.23,7953.95) | 6084.10(4320.30,8198.89) | 0.11 | 136.30(132.82,139.86) | 130.04(126.74,133.41) | -0.16(-0.17,-0.14) |
| Switzerland | both | 6682.14(4657.26,8911.56) | 7034.38(4984.33,9445.94) | 0.25 | 177.54(173.26,181.91) | 165.42(161.47,169.45) | -0.25(-0.25,-0.24) |
| Syrian Arab Republic | both | 2671.98(1796.73,3619.25) | 2815.60(1963.00,3783.78) | 0.32 | 51.95(49.90,54.08) | 39.05(37.55,40.61) | -1.00(-1.06,-0.95) |
| Taiwan (Province of China) | both | 5069.29(3497.88,6967.72) | 4475.68(3116.68,6138.62) | -0.51 | 46.74(45.44,48.07) | 35.17(34.11,36.25) | -0.93(-0.96,-0.91) |
| Tajikistan | both | 1290.06(900.51,1734.76) | 2261.74(1574.57,3082.79) | 2.17 | 56.96(53.67,60.44) | 46.67(44.75,48.67) | -0.65(-0.71,-0.59) |
| Thailand | both | 4384.32(2960.08,6079.80) | 4761.23(3202.70,6806.45) | 0.32 | 14.48(14.05,14.92) | 12.52(12.16,12.89) | -0.48(-0.51,-0.46) |
| Timor-Leste | both | 65.71(43.85,88.48) | 89.29(59.34,123.82) | 0.72 | 18.17(13.95,23.39) | 14.66(11.68,18.21) | -0.95(-1.06,-0.84) |
| Togo | both | 324.04(217.18,451.23) | 699.14(461.40,960.82) | 2.69 | 21.47(19.09,24.10) | 18.35(17.00,19.79) | -0.51(-0.57,-0.44) |
| Tokelau | both | 0.15(0.10,0.21) | 0.12(0.08,0.17) | -1.12 | 23.12(0.00,822.59) | 19.26(0.00,714.48) | -0.66(-0.68,-0.64) |
| Tonga | both | 9.99(6.98,14.00) | 10.22(6.87,13.98) | 0.28 | 24.75(11.47,47.56) | 21.67(10.41,40.12) | -0.38(-0.41,-0.35) |
| Trinidad and Tobago | both | 199.08(137.59,271.27) | 209.31(145.17,281.10) | 0.07 | 32.99(28.52,38.01) | 29.18(25.31,33.53) | -0.47(-0.51,-0.43) |
| Tunisia | both | 1862.30(1300.91,2533.82) | 2297.01(1579.49,3130.36) | 0.68 | 48.23(46.00,50.55) | 36.70(35.20,38.25) | -0.94(-0.99,-0.89) |
| Turkey | both | 21919.91(15291.77,29762.06) | 26967.84(19031.36,35827.48) | 0.64 | 77.08(76.04,78.13) | 57.81(57.12,58.50) | -0.98(-1.00,-0.95) |
| Turkmenistan | both | 855.82(603.93,1159.97) | 1024.25(709.61,1389.50) | 0.58 | 51.40(47.80,55.25) | 38.89(36.54,41.36) | -1.00(-1.03,-0.98) |
| Tuvalu | both | 1.05(0.71,1.44) | 1.19(0.80,1.65) | 0.49 | 23.91(0.68,139.37) | 20.43(0.84,106.33) | -0.50(-0.53,-0.47) |
| Uganda | both | 1140.31(774.11,1584.14) | 2504.16(1661.39,3462.30) | 2.64 | 16.47(15.47,17.52) | 14.16(13.59,14.76) | -0.65(-0.69,-0.61) |
| Ukraine | both | 11592.26(8177.63,15812.24) | 8601.86(5946.54,11577.31) | -1.10 | 46.07(45.23,46.92) | 39.59(38.72,40.48) | -0.54(-0.58,-0.49) |
| United Arab Emirates | both | 438.61(297.52,600.81) | 2167.86(1490.50,3012.63) | 6.78 | 36.36(32.86,40.19) | 27.11(25.61,28.71) | -1.04(-1.13,-0.94) |
| United Kingdom | both | 52766.54(37191.16,70711.74) | 50683.33(35758.03,67499.76) | -0.03 | 181.32(179.77,182.89) | 158.76(157.36,160.17) | -0.43(-0.44,-0.42) |
| United Republic of Tanzania | both | 1693.08(1143.39,2377.69) | 3682.98(2397.20,5168.10) | 2.67 | 16.03(15.24,16.86) | 14.45(13.97,14.93) | -0.33(-0.37,-0.30) |
| United States of America | both | 150092.84(106221.89,200795.40) | 144570.40(101616.89,191470.09) | -0.41 | 110.04(109.48,110.60) | 93.42(92.94,93.91) | -0.77(-0.87,-0.68) |
| United States Virgin Islands | both | 18.66(12.73,25.69) | 12.93(9.07,17.68) | -1.34 | 33.44(19.94,53.07) | 28.71(15.08,50.33) | -0.57(-0.61,-0.54) |
| Uruguay | both | 1344.96(941.99,1795.63) | 1347.77(922.36,1810.60) | -0.02 | 90.70(85.91,95.68) | 80.28(76.04,84.71) | -0.40(-0.42,-0.39) |
| Uzbekistan | both | 5206.38(3547.54,7002.15) | 7694.69(5398.20,10367.93) | 1.40 | 56.27(54.64,57.94) | 42.98(42.02,43.95) | -0.93(-0.94,-0.92) |
| Vanuatu | both | 18.16(12.33,24.63) | 33.93(23.19,46.63) | 2.28 | 27.79(16.27,45.11) | 24.48(16.87,34.57) | -0.43(-0.44,-0.41) |
| Venezuela (Bolivarian Republic of) | both | 1152.29(768.71,1587.55) | 1687.75(1137.25,2389.34) | 1.60 | 12.55(11.81,13.32) | 11.53(10.98,12.10) | -0.25(-0.30,-0.19) |
| Viet Nam | both | 5138.23(3441.61,7247.11) | 7514.92(5059.79,10416.45) | 1.35 | 17.00(16.51,17.50) | 13.80(13.48,14.12) | -0.74(-0.76,-0.71) |
| Yemen | both | 2910.42(2049.32,3928.73) | 6263.89(4351.02,8482.40) | 2.55 | 57.11(54.99,59.31) | 42.05(40.99,43.13) | -1.23(-1.30,-1.16) |
| Zambia | both | 519.64(348.38,728.08) | 1197.17(811.95,1665.00) | 2.94 | 15.86(14.45,17.40) | 13.91(13.11,14.76) | -0.48(-0.52,-0.43) |
| Zimbabwe | both | 1051.86(717.99,1451.75) | 1682.63(1113.79,2360.47) | 1.51 | 24.45(22.92,26.08) | 23.24(22.12,24.40) | -0.03(-0.13,0.07) |
| Afghanistan | female | 1587.42(1102.81,2189.82) | 4481.31(3077.81,6148.36) | 3.23 | 66.90(63.50,70.46) | 54.25(52.60,55.95) | -0.79(-0.88,-0.70) |
| Albania | female | 424.80(293.74,586.52) | 254.77(172.96,347.18) | -1.96 | 52.15(47.18,57.57) | 39.58(34.84,44.81) | -1.05(-1.10,-1.00) |
| Algeria | female | 2641.57(1804.52,3634.17) | 4291.60(2954.27,5985.36) | 1.72 | 48.63(46.70,50.63) | 37.81(36.68,38.96) | -0.86(-0.90,-0.83) |
| American Samoa | female | 2.46(1.60,3.48) | 2.55(1.67,3.58) | -0.10 | 21.44(3.21,81.24) | 18.98(3.13,65.38) | -0.38(-0.42,-0.34) |
| Andorra | female | 26.77(18.51,36.13) | 35.24(24.73,47.27) | 0.70 | 177.25(116.00,262.43) | 164.45(111.47,238.09) | -0.27(-0.28,-0.26) |
| Angola | female | 1138.19(776.32,1553.06) | 2580.08(1760.70,3617.43) | 2.92 | 51.53(48.48,54.73) | 37.84(36.35,39.37) | -1.04(-1.11,-0.98) |
| Antigua and Barbuda | female | 5.62(3.86,7.70) | 8.06(5.51,11.18) | 1.25 | 35.58(12.30,84.55) | 32.06(13.76,65.13) | -0.36(-0.39,-0.33) |
| Argentina | female | 7646.63(5295.66,10283.42) | 9901.12(6734.48,13398.11) | 0.94 | 95.63(93.50,97.80) | 83.67(82.02,85.33) | -0.42(-0.44,-0.40) |
| Armenia | female | 430.13(294.21,578.58) | 290.90(195.17,401.86) | -1.48 | 50.72(45.92,55.96) | 38.59(34.15,43.53) | -1.01(-1.06,-0.97) |
| Australia | female | 7550.48(5261.44,10081.65) | 8742.43(6079.01,11673.25) | 0.53 | 167.83(164.05,171.67) | 146.81(143.70,149.97) | -0.37(-0.45,-0.29) |
| Austria | female | 4006.38(2786.47,5388.24) | 3702.54(2648.34,4967.30) | -0.19 | 199.51(193.34,205.85) | 178.57(172.71,184.61) | -0.38(-0.40,-0.36) |
| Azerbaijan | female | 871.52(598.72,1184.94) | 1079.17(731.43,1506.35) | 0.82 | 47.86(44.58,51.36) | 39.10(36.77,41.57) | -0.77(-0.86,-0.68) |
| Bahamas | female | 23.89(16.24,33.56) | 33.75(22.36,47.14) | 1.15 | 34.12(21.64,51.86) | 31.50(21.74,44.35) | -0.35(-0.39,-0.31) |
| Bahrain | female | 46.12(31.24,63.11) | 116.71(76.70,160.37) | 3.87 | 41.60(29.81,57.57) | 33.92(27.88,41.01) | -0.72(-0.75,-0.68) |
| Bangladesh | female | 7239.62(4765.83,10000.83) | 11527.34(7824.88,16233.00) | 1.69 | 31.21(30.46,31.98) | 26.04(25.57,26.52) | -0.56(-0.60,-0.51) |
| Barbados | female | 22.42(15.49,31.22) | 23.13(15.81,31.63) | 0.13 | 33.20(20.82,50.74) | 31.15(19.61,47.48) | -0.21(-0.23,-0.19) |
| Belarus | female | 1298.95(876.68,1797.09) | 901.97(622.15,1231.33) | -1.37 | 51.47(48.69,54.37) | 40.74(37.98,43.67) | -0.85(-0.89,-0.82) |
| Belgium | female | 5079.61(3561.80,6799.10) | 4702.57(3327.34,6326.44) | -0.29 | 205.37(199.73,211.14) | 182.01(176.75,187.40) | -0.42(-0.43,-0.40) |
| Belize | female | 17.65(11.81,24.50) | 41.71(27.51,56.87) | 3.12 | 45.05(25.58,76.14) | 37.36(26.83,50.80) | -0.60(-0.65,-0.56) |
| Benin | female | 242.81(160.74,336.54) | 565.17(363.94,789.55) | 2.90 | 23.35(20.39,26.67) | 19.66(18.02,21.43) | -0.64(-0.71,-0.57) |
| Bermuda | female | 6.36(4.25,8.75) | 4.87(3.25,6.75) | -1.00 | 36.43(13.61,83.77) | 32.77(9.74,88.00) | -0.42(-0.44,-0.40) |
| Bhutan | female | 43.24(28.86,61.33) | 56.12(37.25,78.69) | 1.26 | 33.08(23.57,45.66) | 27.92(21.05,36.48) | -0.61(-0.63,-0.60) |
| Bolivia (Plurinational State of) | female | 1319.62(910.15,1790.86) | 2258.15(1558.42,3042.50) | 1.97 | 90.02(85.13,95.13) | 75.27(72.19,78.46) | -0.55(-0.61,-0.49) |
| Bosnia and Herzegovina | female | 628.50(429.49,869.22) | 299.96(205.16,408.45) | -2.41 | 53.70(49.58,58.10) | 39.08(34.68,43.93) | -1.22(-1.30,-1.14) |
| Botswana | female | 74.10(48.67,108.20) | 130.63(86.68,186.35) | 1.82 | 24.45(18.96,31.26) | 19.77(16.50,23.53) | -0.78(-0.85,-0.70) |
| Brazil | female | 26743.23(19101.45,35918.33) | 40368.59(28420.03,53650.83) | 1.43 | 71.09(70.23,71.96) | 67.71(67.05,68.38) | -0.16(-0.17,-0.15) |
| Brunei Darussalam | female | 14.78(9.43,20.65) | 25.95(17.01,36.33) | 1.86 | 22.62(12.27,39.95) | 20.62(13.44,30.55) | -0.35(-0.38,-0.32) |
| Bulgaria | female | 994.54(676.45,1350.84) | 559.92(382.32,764.92) | -2.00 | 47.41(44.48,50.49) | 37.00(33.82,40.44) | -0.83(-0.87,-0.79) |
| Burkina Faso | female | 522.53(348.47,733.22) | 1165.87(739.58,1646.90) | 2.83 | 25.93(23.70,28.33) | 22.47(21.17,23.84) | -0.56(-0.64,-0.49) |
| Burundi | female | 196.85(123.52,280.46) | 433.41(279.41,606.51) | 2.94 | 16.49(14.18,19.12) | 16.45(14.87,18.17) | -0.05(-0.09,-0.01) |
| Cabo Verde | female | 16.67(11.20,23.75) | 25.47(16.21,36.45) | 1.42 | 22.54(12.50,38.74) | 17.51(11.34,26.06) | -0.96(-1.04,-0.87) |
| Cambodia | female | 468.94(308.37,672.94) | 721.42(461.55,1043.02) | 1.50 | 19.63(17.85,21.56) | 16.60(15.40,17.87) | -0.62(-0.64,-0.60) |
| Cameroon | female | 459.37(296.26,644.57) | 1164.21(757.98,1699.49) | 3.30 | 20.43(18.54,22.48) | 16.75(15.77,17.79) | -0.69(-0.77,-0.62) |
| Canada | female | 7972.08(5522.76,10798.37) | 7863.18(5607.91,10442.72) | -0.16 | 106.20(103.86,108.59) | 93.72(91.62,95.86) | -0.44(-0.46,-0.43) |
| Central African Republic | female | 312.51(216.56,426.51) | 564.45(384.14,778.17) | 2.07 | 50.65(45.02,56.85) | 44.54(40.84,48.51) | -0.42(-0.44,-0.40) |
| Chad | female | 344.40(225.66,471.57) | 784.02(501.51,1101.37) | 2.76 | 27.24(24.35,30.41) | 23.37(21.69,25.17) | -0.60(-0.67,-0.54) |
| Chile | female | 3387.11(2362.15,4611.72) | 3772.21(2591.07,5112.26) | 0.40 | 95.51(92.30,98.81) | 80.43(77.86,83.06) | -0.60(-0.62,-0.57) |
| China | female | 157344.14(111213.34,210636.74) | 141155.45(99446.13,187313.52) | -0.22 | 50.56(50.31,50.82) | 38.21(38.00,38.42) | -0.97(-0.99,-0.95) |
| Colombia | female | 1075.01(703.27,1524.79) | 1424.34(929.58,1997.48) | 0.88 | 12.90(12.12,13.72) | 11.24(10.66,11.84) | -0.50(-0.52,-0.47) |
| Comoros | female | 16.11(10.63,23.06) | 26.78(16.83,38.15) | 1.77 | 16.23(9.07,27.39) | 14.81(9.72,21.73) | -0.29(-0.32,-0.26) |
| Congo | female | 207.34(143.38,285.56) | 397.61(264.19,559.08) | 2.27 | 38.81(33.47,44.86) | 29.70(26.83,32.80) | -0.92(-0.97,-0.87) |
| Cook Islands | female | 0.94(0.63,1.34) | 0.81(0.52,1.17) | -0.39 | 21.33(0.39,148.08) | 18.51(0.22,128.22) | -0.47(-0.48,-0.45) |
| Costa Rica | female | 95.12(62.22,134.17) | 144.07(94.99,202.06) | 1.42 | 12.71(10.23,15.69) | 11.09(9.35,13.08) | -0.45(-0.49,-0.42) |
| Croatia | female | 551.74(375.81,744.81) | 339.76(227.52,465.18) | -1.78 | 45.70(41.94,49.72) | 36.42(32.54,40.69) | -0.84(-0.87,-0.81) |
| Cuba | female | 1126.39(747.32,1557.24) | 976.00(656.40,1347.08) | -0.36 | 37.76(35.57,40.05) | 35.78(33.51,38.19) | -0.19(-0.23,-0.15) |
| Cyprus | female | 396.18(281.52,535.24) | 604.66(424.16,816.57) | 1.61 | 199.06(179.91,219.77) | 164.78(151.35,179.33) | -0.66(-0.72,-0.61) |
| Czechia | female | 1228.32(847.60,1692.10) | 871.93(600.40,1216.77) | -1.20 | 46.92(44.30,49.67) | 36.15(33.63,38.84) | -0.83(-0.90,-0.76) |
| Côte d'Ivoire | female | 560.13(361.55,800.15) | 1126.07(731.15,1610.45) | 2.31 | 21.80(19.93,23.81) | 18.43(17.34,19.58) | -0.57(-0.65,-0.48) |
| Democratic People's Republic of Korea | female | 3343.35(2309.77,4581.80) | 3338.82(2298.91,4610.50) | 0.13 | 60.94(58.88,63.07) | 49.17(47.50,50.90) | -0.75(-0.78,-0.72) |
| Democratic Republic of the Congo | female | 3697.41(2592.22,5064.06) | 7659.39(5176.23,10691.63) | 2.64 | 45.38(43.87,46.92) | 38.82(37.93,39.73) | -0.45(-0.60,-0.29) |
| Denmark | female | 2678.69(1902.36,3641.13) | 2346.22(1637.33,3157.70) | -0.47 | 200.17(192.60,207.99) | 180.67(173.33,188.27) | -0.34(-0.36,-0.33) |
| Djibouti | female | 16.28(10.39,23.91) | 44.94(27.72,65.42) | 3.91 | 16.43(9.16,28.10) | 14.57(10.60,19.65) | -0.47(-0.49,-0.44) |
| Dominica | female | 5.98(4.08,8.27) | 5.32(3.63,7.34) | -0.33 | 36.79(13.09,84.51) | 32.04(10.84,74.07) | -0.52(-0.57,-0.48) |
| Dominican Republic | female | 793.25(536.62,1107.67) | 1071.09(727.57,1472.18) | 0.86 | 44.46(41.30,47.83) | 37.91(35.67,40.26) | -0.66(-0.73,-0.59) |
| Ecuador | female | 1670.25(1155.09,2298.50) | 2632.34(1811.48,3565.79) | 1.57 | 69.53(66.14,73.06) | 57.61(55.43,59.86) | -0.62(-0.65,-0.60) |
| Egypt | female | 3210.51(2133.04,4446.24) | 5098.52(3336.02,7156.13) | 1.83 | 25.38(24.50,26.29) | 20.75(20.19,21.34) | -0.51(-0.56,-0.45) |
| El Salvador | female | 183.44(118.76,267.98) | 219.19(141.54,307.80) | 0.58 | 14.84(12.71,17.25) | 12.56(10.94,14.34) | -0.60(-0.64,-0.56) |
| Equatorial Guinea | female | 47.97(31.91,65.01) | 87.33(57.13,122.87) | 1.87 | 50.61(37.02,68.10) | 27.08(21.53,33.83) | -2.42(-2.52,-2.31) |
| Eritrea | female | 118.42(77.37,169.07) | 260.46(167.60,368.84) | 2.89 | 18.26(15.02,22.07) | 16.07(14.13,18.23) | -0.40(-0.46,-0.33) |
| Estonia | female | 187.56(128.66,259.99) | 111.17(74.98,151.51) | -1.76 | 48.92(42.12,56.57) | 38.57(31.46,47.04) | -0.81(-0.83,-0.78) |
| Eswatini | female | 48.23(31.49,67.82) | 65.39(44.03,93.38) | 0.84 | 26.34(19.13,35.73) | 21.72(16.66,28.04) | -0.68(-0.75,-0.61) |
| Ethiopia | female | 1937.20(1350.15,2641.03) | 3958.28(2729.68,5345.17) | 2.39 | 17.91(17.09,18.77) | 15.86(15.35,16.38) | -0.41(-0.45,-0.36) |
| Fiji | female | 45.65(30.96,63.73) | 47.60(31.68,66.71) | 0.20 | 24.11(17.55,32.50) | 20.94(15.42,27.82) | -0.47(-0.50,-0.43) |
| Finland | female | 2929.95(2072.93,3878.29) | 2340.89(1653.46,3155.97) | -0.88 | 223.37(215.20,231.82) | 199.58(191.47,207.97) | -0.40(-0.41,-0.39) |
| France | female | 38129.11(27024.18,51057.76) | 34053.55(23704.36,46252.28) | -0.47 | 260.96(258.33,263.60) | 230.42(227.94,232.92) | -0.44(-0.47,-0.40) |
| Gabon | female | 77.39(53.34,107.15) | 127.94(88.11,179.28) | 1.79 | 37.34(29.10,47.61) | 27.05(22.52,32.26) | -1.06(-1.09,-1.03) |
| Gambia | female | 51.72(35.32,71.17) | 106.96(67.77,152.32) | 2.36 | 24.33(17.78,33.00) | 19.55(15.89,23.95) | -0.80(-0.88,-0.72) |
| Georgia | female | 625.59(437.91,856.95) | 324.68(219.55,465.19) | -2.31 | 45.89(42.35,49.66) | 39.05(34.81,43.73) | -0.53(-0.59,-0.47) |
| Germany | female | 28550.86(19907.16,38808.77) | 24132.80(17018.62,32607.03) | -0.67 | 143.51(141.83,145.21) | 133.81(132.09,135.55) | -0.31(-0.36,-0.26) |
| Ghana | female | 879.25(585.00,1216.56) | 1794.89(1189.20,2495.23) | 2.46 | 26.18(24.42,28.05) | 21.36(20.37,22.39) | -0.79(-0.85,-0.72) |
| Greece | female | 5160.67(3605.32,6902.42) | 4218.42(2999.98,5636.33) | -0.70 | 202.37(196.87,207.98) | 177.36(171.78,183.10) | -0.45(-0.49,-0.41) |
| Greenland | female | 18.99(13.53,25.48) | 14.66(10.46,19.68) | -0.79 | 130.33(77.20,209.96) | 114.28(63.22,192.37) | -0.52(-0.55,-0.50) |
| Grenada | female | 7.53(5.15,10.64) | 8.77(6.08,12.05) | 0.64 | 40.69(16.40,88.19) | 33.58(15.08,65.77) | -0.56(-0.64,-0.48) |
| Guam | female | 6.60(4.23,9.22) | 6.58(4.26,9.51) | -0.18 | 19.42(7.39,44.22) | 16.90(6.52,36.35) | -0.50(-0.55,-0.46) |
| Guatemala | female | 278.39(176.57,415.37) | 651.20(411.50,952.59) | 3.08 | 16.39(14.47,18.53) | 13.50(12.47,14.60) | -0.67(-0.71,-0.63) |
| Guinea | female | 335.37(221.95,466.14) | 617.62(400.55,874.61) | 2.04 | 24.66(22.04,27.53) | 20.88(19.21,22.67) | -0.58(-0.66,-0.50) |
| Guinea-Bissau | female | 55.72(37.35,78.15) | 96.82(62.85,137.62) | 1.94 | 24.74(18.47,32.72) | 20.44(16.44,25.27) | -0.69(-0.77,-0.61) |
| Guyana | female | 72.40(50.35,99.81) | 67.88(46.48,94.32) | -0.36 | 37.42(29.03,47.81) | 32.94(25.53,41.90) | -0.41(-0.43,-0.39) |
| Haiti | female | 739.23(512.50,1017.94) | 1522.26(1043.82,2098.94) | 2.54 | 50.36(46.71,54.25) | 45.21(42.94,47.56) | -0.39(-0.41,-0.37) |
| Honduras | female | 160.73(105.27,227.74) | 365.07(239.55,519.62) | 2.88 | 16.19(13.69,19.05) | 13.92(12.51,15.46) | -0.55(-0.59,-0.50) |
| Hungary | female | 1224.62(835.44,1680.52) | 835.90(569.71,1155.68) | -1.41 | 47.57(44.90,50.37) | 37.04(34.44,39.80) | -0.84(-0.89,-0.78) |
| Iceland | female | 144.07(101.83,193.57) | 159.53(110.66,213.03) | 0.31 | 223.09(188.06,263.02) | 197.30(167.68,231.01) | -0.44(-0.46,-0.43) |
| India | female | 63772.05(45132.00,84924.91) | 117090.08(83025.77,156047.84) | 2.17 | 32.67(32.41,32.93) | 32.12(31.94,32.31) | -0.06(-0.14,0.02) |
| Indonesia | female | 4029.20(2778.95,5431.55) | 5655.74(3941.71,7844.17) | 1.23 | 8.74(8.47,9.02) | 7.93(7.72,8.14) | -0.26(-0.30,-0.23) |
| Iran (Islamic Republic of) | female | 6344.46(4457.89,8505.33) | 9051.77(6359.00,12389.42) | 1.38 | 51.91(50.58,53.27) | 37.66(36.87,38.47) | -1.18(-1.25,-1.11) |
| Iraq | female | 2151.03(1500.40,2915.65) | 4714.18(3208.99,6535.96) | 2.56 | 59.99(57.39,62.69) | 43.41(42.16,44.68) | -1.14(-1.21,-1.07) |
| Ireland | female | 1861.17(1306.25,2484.77) | 2234.44(1588.73,2952.65) | 0.84 | 212.50(202.94,222.41) | 181.35(173.70,189.28) | -0.54(-0.56,-0.51) |
| Israel | female | 2234.51(1577.53,3023.04) | 3642.69(2534.48,4903.77) | 1.57 | 185.20(177.55,193.12) | 166.00(160.64,171.50) | -0.35(-0.37,-0.33) |
| Italy | female | 26810.73(18857.05,35778.57) | 23069.07(16345.86,30911.70) | -0.24 | 185.60(183.38,187.84) | 172.70(170.35,175.07) | -0.15(-0.24,-0.06) |
| Jamaica | female | 222.88(148.25,306.48) | 263.32(176.94,369.42) | 0.56 | 39.56(34.35,45.42) | 34.24(30.22,38.65) | -0.50(-0.52,-0.47) |
| Japan | female | 8388.25(5905.90,11244.35) | 6675.57(4697.10,8912.57) | -0.73 | 25.41(24.86,25.97) | 24.79(24.16,25.42) | -0.04(-0.05,-0.02) |
| Jordan | female | 352.46(240.65,480.52) | 994.28(660.02,1358.19) | 3.66 | 45.69(40.75,51.12) | 35.44(33.26,37.73) | -0.91(-0.93,-0.88) |
| Kazakhstan | female | 2050.04(1386.87,2822.14) | 1886.43(1292.42,2559.58) | -0.12 | 50.56(48.37,52.84) | 39.94(38.13,41.81) | -0.80(-0.82,-0.78) |
| Kenya | female | 708.05(494.86,955.60) | 1675.03(1176.48,2253.82) | 3.01 | 14.75(13.61,15.97) | 13.27(12.62,13.94) | -0.31(-0.35,-0.27) |
| Kiribati | female | 4.31(2.85,6.17) | 7.07(4.60,10.06) | 2.03 | 24.08(6.72,65.57) | 23.20(9.31,49.35) | 0.01(-0.04,0.05) |
| Kuwait | female | 152.55(104.89,209.56) | 446.03(299.73,618.81) | 4.25 | 37.27(31.33,44.25) | 30.80(27.85,34.07) | -0.83(-0.89,-0.78) |
| Kyrgyzstan | female | 536.84(367.86,738.70) | 739.44(505.55,1007.87) | 1.24 | 52.58(48.02,57.52) | 44.31(41.16,47.64) | -0.51(-0.53,-0.49) |
| Lao People's Democratic Republic | female | 190.42(127.26,278.40) | 325.48(208.70,470.17) | 1.91 | 20.57(17.68,23.83) | 17.17(15.34,19.17) | -0.64(-0.67,-0.61) |
| Latvia | female | 312.81(215.48,420.57) | 159.36(108.52,220.44) | -2.23 | 47.86(42.66,53.56) | 38.42(32.41,45.37) | -0.73(-0.77,-0.69) |
| Lebanon | female | 362.57(251.78,497.29) | 504.05(345.56,681.80) | 1.27 | 48.56(43.66,53.88) | 36.17(33.03,39.57) | -1.07(-1.11,-1.04) |
| Lesotho | female | 119.10(79.14,165.89) | 132.64(88.08,182.79) | 0.18 | 29.61(24.36,35.78) | 24.28(20.25,28.96) | -0.67(-0.73,-0.61) |
| Liberia | female | 91.87(59.01,130.72) | 228.39(150.56,319.95) | 3.14 | 21.99(17.62,27.23) | 19.02(16.60,21.72) | -0.71(-0.78,-0.65) |
| Libya | female | 370.70(255.63,507.34) | 679.49(458.82,945.19) | 2.33 | 45.53(40.76,50.77) | 33.78(31.27,36.43) | -0.99(-1.10,-0.89) |
| Lithuania | female | 439.65(304.06,602.94) | 229.69(158.28,318.90) | -2.27 | 47.57(43.21,52.26) | 37.35(32.53,42.77) | -0.86(-0.89,-0.84) |
| Luxembourg | female | 187.23(130.79,253.49) | 272.78(191.20,364.90) | 1.25 | 187.82(161.53,217.79) | 172.77(152.33,195.59) | -0.30(-0.31,-0.30) |
| Madagascar | female | 447.70(285.70,641.32) | 1045.70(671.04,1514.37) | 3.01 | 17.65(15.99,19.47) | 16.39(15.39,17.45) | -0.23(-0.26,-0.20) |
| Malawi | female | 374.50(237.59,538.46) | 714.30(446.74,1046.44) | 2.20 | 18.05(16.19,20.09) | 16.26(15.03,17.57) | -0.39(-0.43,-0.35) |
| Malaysia | female | 596.96(351.90,855.16) | 956.53(609.71,1419.64) | 1.59 | 13.75(12.65,14.93) | 11.61(10.89,12.38) | -0.54(-0.59,-0.50) |
| Maldives | female | 7.73(5.03,11.32) | 15.65(9.92,22.45) | 2.69 | 17.34(6.87,38.33) | 13.70(7.69,23.02) | -0.77(-0.83,-0.71) |
| Mali | female | 396.40(264.30,566.61) | 824.20(533.27,1191.91) | 2.59 | 21.62(19.49,23.93) | 17.56(16.33,18.88) | -0.79(-0.84,-0.74) |
| Malta | female | 196.84(137.52,261.10) | 171.81(120.41,233.24) | -0.55 | 201.73(174.17,232.76) | 171.85(146.46,201.09) | -0.53(-0.55,-0.51) |
| Marshall Islands | female | 2.42(1.58,3.38) | 3.23(2.13,4.54) | 0.90 | 26.25(3.55,115.07) | 22.13(4.91,64.46) | -0.52(-0.55,-0.49) |
| Mauritania | female | 102.36(68.11,144.70) | 182.86(117.65,266.97) | 1.92 | 22.94(18.58,28.14) | 18.95(16.24,22.02) | -0.69(-0.77,-0.60) |
| Mauritius | female | 46.37(30.50,65.95) | 44.29(27.89,63.02) | -0.27 | 15.76(11.50,21.25) | 13.46(9.77,18.17) | -0.54(-0.56,-0.51) |
| Mexico | female | 2996.78(2139.89,3997.13) | 4337.20(3001.32,5832.09) | 1.26 | 14.46(13.93,15.00) | 12.70(12.32,13.08) | -0.44(-0.47,-0.41) |
| Micronesia (Federated States of) | female | 5.32(3.44,7.54) | 5.36(3.56,7.59) | -0.14 | 24.39(7.78,63.09) | 21.06(7.10,49.17) | -0.46(-0.49,-0.44) |
| Monaco | female | 13.11(9.16,17.59) | 12.53(8.86,16.90) | 0.04 | 177.70(91.76,331.26) | 163.27(82.76,297.00) | -0.28(-0.29,-0.27) |
| Mongolia | female | 281.72(193.61,390.65) | 398.69(267.11,545.70) | 1.42 | 56.68(49.89,64.28) | 43.79(39.53,48.43) | -0.85(-0.87,-0.83) |
| Montenegro | female | 68.57(47.75,94.06) | 53.34(36.08,73.36) | -1.03 | 44.16(34.32,56.01) | 35.88(26.77,47.30) | -0.80(-0.86,-0.75) |
| Morocco | female | 3359.05(2282.63,4573.05) | 4185.51(2899.42,5778.81) | 0.74 | 55.98(54.06,57.96) | 43.41(42.10,44.74) | -0.89(-0.91,-0.87) |
| Mozambique | female | 595.35(390.80,836.26) | 1177.09(771.83,1699.44) | 2.22 | 20.23(18.61,21.97) | 17.52(16.50,18.60) | -0.52(-0.55,-0.49) |
| Myanmar | female | 1968.80(1292.90,2809.76) | 2487.44(1581.65,3548.94) | 0.70 | 20.13(19.23,21.07) | 16.56(15.92,17.22) | -0.75(-0.79,-0.71) |
| Namibia | female | 77.00(51.00,109.24) | 127.95(83.71,180.88) | 1.63 | 24.13(18.82,30.65) | 20.42(17.00,24.37) | -0.59(-0.63,-0.55) |
| Nauru | female | 0.49(0.32,0.70) | 0.51(0.34,0.71) | 0.01 | 20.91(0.01,242.77) | 18.75(0.02,203.49) | -0.30(-0.41,-0.19) |
| Nepal | female | 1294.01(878.05,1834.25) | 1890.31(1261.52,2696.31) | 1.21 | 29.69(28.06,31.39) | 22.07(21.08,23.10) | -1.16(-1.22,-1.11) |
| Netherlands | female | 7958.61(5572.26,10730.16) | 6850.42(4840.20,9344.16) | -0.55 | 198.08(193.73,202.50) | 177.98(173.73,182.33) | -0.35(-0.36,-0.33) |
| New Zealand | female | 1692.36(1195.32,2306.21) | 1753.64(1244.38,2360.15) | 0.18 | 187.00(178.18,196.14) | 168.02(160.14,176.21) | -0.35(-0.37,-0.34) |
| Nicaragua | female | 130.49(81.61,192.39) | 231.08(149.01,335.28) | 1.94 | 15.57(12.89,18.71) | 13.28(11.61,15.12) | -0.54(-0.58,-0.51) |
| Niger | female | 466.54(306.57,651.93) | 1168.39(760.60,1657.95) | 3.13 | 28.36(25.75,31.19) | 25.72(24.18,27.33) | -0.35(-0.42,-0.28) |
| Nigeria | female | 5895.75(4145.53,7952.19) | 11072.88(7896.56,14651.85) | 2.10 | 31.17(30.34,32.03) | 21.78(21.37,22.21) | -1.31(-1.42,-1.21) |
| Niue | female | 0.11(0.08,0.16) | 0.07(0.05,0.11) | -1.78 | 22.82(0.00,874.00) | 19.43(0.00,1154.81) | -0.56(-0.58,-0.54) |
| North Macedonia | female | 246.80(167.32,332.71) | 197.40(130.78,269.16) | -0.76 | 48.12(42.30,54.53) | 37.32(32.18,43.15) | -0.91(-0.94,-0.87) |
| Northern Mariana Islands | female | 2.58(1.72,3.64) | 1.78(1.17,2.52) | -1.43 | 19.41(3.03,76.85) | 18.54(1.36,102.28) | -0.06(-0.11,0.00) |
| Norway | female | 1712.50(1203.14,2283.48) | 1846.51(1303.70,2455.61) | 0.26 | 159.96(152.45,167.76) | 147.70(140.94,154.72) | -0.28(-0.30,-0.25) |
| Oman | female | 155.12(105.78,212.28) | 301.32(198.31,423.03) | 2.36 | 48.23(40.70,56.93) | 31.30(27.78,35.20) | -1.45(-1.57,-1.32) |
| Pakistan | female | 7426.24(5147.26,10055.24) | 14685.55(10305.07,19767.24) | 2.36 | 32.94(32.18,33.72) | 27.64(27.19,28.10) | -0.64(-0.68,-0.61) |
| Palau | female | 0.81(0.53,1.13) | 0.73(0.48,1.02) | -0.66 | 20.07(0.22,152.97) | 17.66(0.09,159.31) | -0.37(-0.40,-0.34) |
| Palestine | female | 234.92(165.01,321.49) | 479.80(328.41,664.87) | 2.64 | 57.79(50.28,66.22) | 40.25(36.66,44.13) | -1.27(-1.31,-1.24) |
| Panama | female | 72.42(47.32,102.81) | 113.82(72.69,161.10) | 1.56 | 12.33(9.61,15.65) | 10.93(9.01,13.14) | -0.39(-0.41,-0.38) |
| Papua New Guinea | female | 262.32(175.64,362.18) | 631.09(416.39,882.98) | 3.21 | 28.63(25.18,32.47) | 25.89(23.90,28.02) | -0.29(-0.32,-0.26) |
| Paraguay | female | 674.29(469.52,910.21) | 1294.85(880.29,1767.97) | 2.27 | 75.02(69.34,81.07) | 72.13(68.23,76.19) | -0.14(-0.16,-0.11) |
| Peru | female | 3872.33(2687.00,5253.35) | 5693.66(3948.69,7592.98) | 1.33 | 74.73(72.34,77.19) | 63.46(61.82,65.13) | -0.56(-0.60,-0.51) |
| Philippines | female | 2390.45(1687.66,3193.44) | 4167.72(2924.51,5691.93) | 2.07 | 16.04(15.39,16.72) | 14.82(14.37,15.28) | -0.13(-0.21,-0.06) |
| Poland | female | 4880.22(3486.51,6544.96) | 4282.84(3019.59,5781.74) | -0.54 | 51.85(50.39,53.35) | 46.97(45.51,48.47) | -0.24(-0.30,-0.17) |
| Portugal | female | 5561.30(3880.19,7458.28) | 4775.44(3313.39,6510.82) | -0.53 | 218.90(213.18,224.74) | 188.71(183.15,194.42) | -0.50(-0.52,-0.48) |
| Puerto Rico | female | 317.89(209.64,439.34) | 249.44(166.65,343.40) | -0.93 | 33.21(29.65,37.07) | 29.66(26.05,33.66) | -0.44(-0.46,-0.42) |
| Qatar | female | 31.48(21.67,43.97) | 147.10(100.10,205.58) | 6.10 | 40.08(26.85,58.95) | 30.39(25.41,36.27) | -0.92(-0.95,-0.88) |
| Republic of Korea | female | 3526.03(2279.88,4834.36) | 3140.78(2069.65,4368.72) | -0.32 | 28.44(27.50,29.41) | 24.09(23.22,24.99) | -0.51(-0.56,-0.46) |
| Republic of Moldova | female | 553.95(383.78,771.17) | 374.82(259.53,525.13) | -1.45 | 49.43(45.37,53.77) | 40.87(36.68,45.47) | -0.67(-0.74,-0.60) |
| Romania | female | 2502.95(1721.01,3456.54) | 1478.24(984.13,2044.52) | -1.91 | 44.33(42.60,46.12) | 33.94(32.15,35.81) | -0.91(-0.95,-0.86) |
| Russian Federation | female | 16242.49(11569.16,21804.92) | 13647.42(9718.13,18242.27) | -0.75 | 44.02(43.34,44.71) | 39.00(38.32,39.70) | -0.39(-0.43,-0.36) |
| Rwanda | female | 219.41(135.18,318.33) | 412.13(255.15,601.11) | 2.80 | 14.42(12.48,16.61) | 12.94(11.69,14.29) | -0.38(-0.41,-0.34) |
| Saint Kitts and Nevis | female | 3.42(2.23,4.70) | 5.04(3.37,6.91) | 1.44 | 36.28(7.54,125.71) | 31.55(10.22,76.19) | -0.47(-0.49,-0.44) |
| Saint Lucia | female | 12.53(8.51,17.43) | 16.16(10.87,22.34) | 0.98 | 38.90(19.85,70.90) | 33.71(19.23,55.49) | -0.46(-0.52,-0.41) |
| Saint Vincent and the Grenadines | female | 10.01(6.64,13.85) | 9.95(6.77,14.19) | -0.01 | 40.59(18.61,80.95) | 34.85(16.63,64.79) | -0.49(-0.52,-0.46) |
| Samoa | female | 8.16(5.39,11.45) | 10.95(7.33,15.32) | 0.99 | 23.62(9.87,49.49) | 22.52(11.09,41.27) | -0.09(-0.12,-0.05) |
| San Marino | female | 10.76(7.59,14.36) | 14.00(9.96,18.79) | 1.02 | 183.24(90.42,334.24) | 167.80(90.00,291.64) | -0.30(-0.32,-0.28) |
| Sao Tome and Principe | female | 4.82(3.23,6.75) | 8.34(5.35,11.80) | 1.81 | 20.06(5.88,53.15) | 16.24(7.06,32.80) | -0.83(-0.89,-0.77) |
| Saudi Arabia | female | 1627.76(1093.01,2207.68) | 3148.95(2147.93,4297.12) | 2.54 | 52.69(50.06,55.45) | 31.44(30.33,32.58) | -1.68(-1.79,-1.57) |
| Senegal | female | 383.45(259.24,531.87) | 716.66(460.43,1007.40) | 2.09 | 23.69(21.28,26.32) | 20.17(18.68,21.74) | -0.58(-0.66,-0.50) |
| Serbia | female | 1096.10(756.71,1497.92) | 738.90(503.60,1012.62) | -1.69 | 48.09(45.27,51.04) | 37.06(34.39,39.90) | -0.94(-0.98,-0.90) |
| Seychelles | female | 2.37(1.43,3.43) | 3.04(1.97,4.41) | 1.00 | 13.75(1.91,53.72) | 12.16(2.48,37.66) | -0.35(-0.40,-0.31) |
| Sierra Leone | female | 195.10(128.28,276.30) | 405.21(258.96,588.88) | 2.89 | 23.52(20.25,27.24) | 20.12(18.14,22.30) | -0.58(-0.64,-0.52) |
| Singapore | female | 203.91(128.24,281.96) | 302.11(193.23,426.36) | 1.48 | 21.85(18.93,25.12) | 19.36(17.09,21.94) | -0.38(-0.41,-0.35) |
| Slovakia | female | 643.81(444.84,865.39) | 476.94(325.40,653.53) | -1.12 | 48.42(44.74,52.34) | 36.42(33.07,40.06) | -0.95(-1.01,-0.89) |
| Slovenia | female | 220.20(152.41,299.54) | 153.35(104.01,214.85) | -1.27 | 44.19(38.52,50.50) | 34.74(29.20,41.19) | -0.81(-0.86,-0.76) |
| Solomon Islands | female | 20.59(13.80,29.37) | 41.57(28.09,56.91) | 2.49 | 29.63(17.74,47.45) | 26.02(18.67,35.46) | -0.38(-0.41,-0.34) |
| Somalia | female | 298.02(192.88,423.07) | 911.36(598.20,1299.48) | 3.75 | 19.99(17.75,22.46) | 20.97(19.59,22.44) | 0.13(0.10,0.15) |
| South Africa | female | 2147.13(1531.35,2913.54) | 3037.47(2160.88,4059.11) | 1.09 | 22.84(21.85,23.85) | 20.02(19.31,20.75) | -0.50(-0.54,-0.47) |
| South Sudan | female | 194.31(127.07,278.31) | 322.54(208.25,462.89) | 1.94 | 16.11(13.80,18.75) | 15.26(13.60,17.07) | -0.20(-0.23,-0.17) |
| Spain | female | 19306.84(13101.04,26099.54) | 19244.15(13329.35,25805.25) | 0.27 | 200.78(197.96,203.64) | 175.90(173.27,178.57) | -0.43(-0.45,-0.41) |
| Sri Lanka | female | 1339.25(877.55,1865.49) | 1357.53(935.67,1886.60) | -0.03 | 29.77(28.18,31.43) | 23.91(22.65,25.22) | -0.74(-0.77,-0.71) |
| Sudan | female | 2470.73(1687.03,3361.00) | 4069.24(2774.29,5560.75) | 1.64 | 55.96(53.70,58.29) | 40.00(38.76,41.28) | -1.17(-1.19,-1.15) |
| Suriname | female | 33.58(22.49,46.87) | 47.51(32.57,66.30) | 1.43 | 36.13(24.79,51.15) | 32.31(23.76,42.99) | -0.45(-0.47,-0.42) |
| Sweden | female | 2929.15(2029.26,3879.52) | 2989.13(2105.88,4018.77) | 0.12 | 138.33(133.30,143.52) | 132.50(127.71,137.44) | -0.14(-0.16,-0.12) |
| Switzerland | female | 3168.72(2225.62,4250.18) | 3336.36(2297.24,4413.50) | 0.26 | 174.90(168.79,181.21) | 162.59(156.96,168.41) | -0.26(-0.26,-0.25) |
| Syrian Arab Republic | female | 1384.40(941.46,1884.97) | 1581.98(1082.08,2161.84) | 0.56 | 54.04(51.08,57.14) | 40.54(38.50,42.68) | -1.01(-1.06,-0.95) |
| Taiwan (Province of China) | female | 2309.31(1553.57,3199.68) | 1999.23(1347.93,2758.19) | -0.55 | 42.83(41.08,44.65) | 32.50(31.04,34.03) | -0.91(-0.94,-0.89) |
| Tajikistan | female | 648.90(440.74,878.99) | 1102.71(754.69,1518.81) | 2.08 | 55.63(51.09,60.55) | 45.50(42.82,48.31) | -0.64(-0.70,-0.59) |
| Thailand | female | 2381.86(1559.04,3367.68) | 2500.26(1575.56,3690.64) | 0.20 | 15.33(14.71,15.97) | 13.29(12.76,13.84) | -0.45(-0.48,-0.42) |
| Timor-Leste | female | 34.88(22.61,48.83) | 48.65(30.74,69.12) | 0.79 | 19.40(13.38,27.51) | 15.62(11.41,21.01) | -0.95(-1.06,-0.83) |
| Togo | female | 176.29(113.37,251.79) | 367.97(238.85,517.11) | 2.57 | 21.85(18.63,25.54) | 18.45(16.59,20.47) | -0.57(-0.65,-0.49) |
| Tokelau | female | 0.08(0.05,0.11) | 0.06(0.04,0.09) | -1.41 | 23.58(0.00,1519.66) | 19.57(0.00,1797.79) | -0.66(-0.68,-0.64) |
| Tonga | female | 5.35(3.64,7.60) | 5.43(3.62,7.67) | 0.23 | 25.21(8.25,60.49) | 22.12(7.55,50.80) | -0.37(-0.41,-0.34) |
| Trinidad and Tobago | female | 99.99(69.09,138.12) | 104.37(70.58,141.65) | 0.04 | 33.33(27.06,40.73) | 29.58(24.10,36.03) | -0.47(-0.50,-0.43) |
| Tunisia | female | 977.41(658.42,1333.06) | 1210.92(820.44,1634.82) | 0.72 | 50.18(47.00,53.55) | 38.22(36.07,40.47) | -0.94(-0.99,-0.89) |
| Turkey | female | 11888.24(8377.27,16212.31) | 14213.53(9813.78,19025.26) | 0.55 | 84.59(83.04,86.15) | 62.82(61.79,63.87) | -1.01(-1.04,-0.99) |
| Turkmenistan | female | 433.08(303.92,592.77) | 469.36(317.02,651.64) | 0.25 | 50.39(45.47,55.79) | 37.85(34.50,41.44) | -1.01(-1.04,-0.98) |
| Tuvalu | female | 0.59(0.40,0.82) | 0.57(0.38,0.81) | -0.05 | 24.42(0.06,214.51) | 20.83(0.04,189.11) | -0.48(-0.52,-0.45) |
| Uganda | female | 602.93(388.83,864.89) | 1322.44(822.48,1928.80) | 2.64 | 16.85(15.44,18.36) | 14.36(13.56,15.20) | -0.67(-0.71,-0.63) |
| Ukraine | female | 5895.70(4086.32,8038.40) | 4269.79(2910.66,5792.62) | -1.16 | 46.43(45.25,47.64) | 39.92(38.66,41.21) | -0.53(-0.57,-0.49) |
| United Arab Emirates | female | 127.30(85.72,174.38) | 531.62(361.52,754.13) | 5.86 | 38.31(31.51,46.57) | 28.90(26.17,31.88) | -1.00(-1.09,-0.92) |
| United Kingdom | female | 22309.49(15862.97,29805.15) | 21101.21(14915.14,28179.94) | -0.10 | 154.02(152.00,156.07) | 134.56(132.72,136.42) | -0.45(-0.47,-0.43) |
| United Republic of Tanzania | female | 914.44(590.84,1312.43) | 1975.92(1183.35,2908.13) | 2.63 | 16.33(15.23,17.49) | 14.68(14.02,15.36) | -0.35(-0.39,-0.32) |
| United States of America | female | 77148.90(54447.82,102975.43) | 74266.28(52031.53,97860.32) | -0.39 | 113.77(112.97,114.59) | 96.96(96.26,97.66) | -0.76(-0.85,-0.66) |
| United States Virgin Islands | female | 9.93(6.61,13.64) | 6.88(4.68,9.56) | -1.33 | 33.86(16.09,63.66) | 29.10(11.34,63.05) | -0.56(-0.60,-0.52) |
| Uruguay | female | 703.12(488.87,970.73) | 700.72(488.14,940.23) | -0.03 | 93.51(86.73,100.69) | 82.37(76.35,88.75) | -0.42(-0.44,-0.41) |
| Uzbekistan | female | 2604.47(1777.72,3590.01) | 3757.19(2551.02,5182.01) | 1.35 | 54.80(52.57,57.12) | 41.90(40.57,43.27) | -0.92(-0.94,-0.90) |
| Vanuatu | female | 9.41(6.19,13.09) | 17.69(11.78,25.03) | 2.31 | 28.28(12.79,56.55) | 24.89(14.54,40.39) | -0.41(-0.43,-0.39) |
| Venezuela (Bolivarian Republic of) | female | 592.66(389.21,841.29) | 873.86(568.74,1264.16) | 1.56 | 12.77(11.73,13.88) | 11.68(10.92,12.49) | -0.26(-0.32,-0.20) |
| Viet Nam | female | 2923.62(1901.03,4252.95) | 3925.63(2506.56,5683.13) | 1.08 | 18.01(17.33,18.71) | 14.68(14.22,15.15) | -0.70(-0.72,-0.67) |
| Yemen | female | 1531.94(1067.53,2096.92) | 3249.65(2182.32,4436.25) | 2.52 | 59.14(56.10,62.32) | 43.45(41.93,45.01) | -1.24(-1.31,-1.17) |
| Zambia | female | 275.25(176.96,398.65) | 619.43(410.18,907.82) | 2.79 | 16.15(14.18,18.35) | 14.22(13.08,15.45) | -0.50(-0.56,-0.45) |
| Zimbabwe | female | 556.54(373.70,790.00) | 894.27(572.38,1297.78) | 1.48 | 24.76(22.63,27.07) | 23.54(21.99,25.19) | -0.06(-0.15,0.04) |
| Afghanistan | male | 1211.55(815.82,1672.31) | 4359.64(2957.06,6104.16) | 3.96 | 61.17(57.50,65.04) | 50.78(49.22,52.37) | -0.69(-0.79,-0.59) |
| Albania | male | 470.68(314.19,650.45) | 282.84(188.61,388.13) | -2.05 | 55.92(50.92,61.32) | 43.33(38.41,48.74) | -1.03(-1.09,-0.97) |
| Algeria | male | 2442.98(1675.08,3364.22) | 3994.62(2720.43,5506.36) | 1.76 | 44.95(43.10,46.88) | 34.62(33.55,35.72) | -0.87(-0.90,-0.83) |
| American Samoa | male | 2.37(1.58,3.31) | 2.42(1.62,3.41) | -0.13 | 20.80(3.08,74.74) | 18.23(2.78,65.62) | -0.42(-0.45,-0.38) |
| Andorra | male | 32.97(23.04,43.72) | 40.30(28.04,54.44) | 0.36 | 183.01(125.29,261.68) | 169.81(118.45,240.21) | -0.26(-0.27,-0.25) |
| Angola | male | 1131.13(753.79,1574.76) | 2291.31(1518.04,3193.43) | 2.49 | 49.90(46.95,53.00) | 37.08(35.54,38.68) | -1.00(-1.06,-0.93) |
| Antigua and Barbuda | male | 5.09(3.42,7.19) | 7.34(5.03,10.05) | 1.34 | 34.72(11.15,86.32) | 30.99(12.73,63.99) | -0.39(-0.40,-0.37) |
| Argentina | male | 6973.00(4719.34,9404.02) | 9202.15(6393.20,12433.04) | 1.02 | 89.45(87.36,91.58) | 79.39(77.77,81.03) | -0.37(-0.39,-0.35) |
| Armenia | male | 422.99(287.06,593.34) | 302.29(200.31,410.96) | -1.42 | 52.47(47.43,57.97) | 40.60(36.09,45.57) | -0.98(-1.03,-0.93) |
| Australia | male | 6552.03(4585.52,8761.65) | 7467.01(5205.19,10129.31) | 0.49 | 142.54(139.11,146.05) | 123.95(121.12,126.83) | -0.37(-0.45,-0.29) |
| Austria | male | 4275.95(3023.91,5834.85) | 4040.40(2860.90,5423.34) | -0.17 | 205.17(199.03,211.47) | 184.41(178.66,190.32) | -0.37(-0.39,-0.35) |
| Azerbaijan | male | 832.29(550.37,1130.89) | 1168.52(792.46,1615.66) | 1.19 | 49.94(46.41,53.70) | 41.15(38.80,43.61) | -0.77(-0.87,-0.66) |
| Bahamas | male | 22.28(14.99,30.52) | 31.29(21.01,43.37) | 1.13 | 33.26(20.67,51.48) | 30.89(21.00,43.98) | -0.31(-0.34,-0.28) |
| Bahrain | male | 72.82(48.78,101.30) | 211.39(140.11,298.46) | 4.87 | 38.43(29.53,49.73) | 30.90(26.23,36.38) | -0.75(-0.78,-0.72) |
| Bangladesh | male | 7547.12(5048.49,10618.60) | 10500.28(6683.37,14826.61) | 1.16 | 31.05(30.34,31.78) | 25.80(25.31,26.30) | -0.57(-0.61,-0.52) |
| Barbados | male | 20.92(13.98,28.90) | 21.27(13.93,29.73) | 0.07 | 32.35(19.89,50.41) | 30.08(18.58,46.39) | -0.22(-0.23,-0.20) |
| Belarus | male | 1277.04(883.54,1753.68) | 931.67(638.33,1279.22) | -1.25 | 50.77(48.00,53.66) | 40.22(37.59,43.02) | -0.83(-0.87,-0.79) |
| Belgium | male | 5479.37(3830.82,7310.84) | 5035.88(3528.09,6852.46) | -0.32 | 211.53(205.94,217.25) | 187.63(182.39,192.98) | -0.41(-0.42,-0.39) |
| Belize | male | 17.76(12.07,24.66) | 38.91(26.86,53.78) | 2.87 | 44.15(25.30,73.78) | 36.31(25.74,49.94) | -0.61(-0.65,-0.56) |
| Benin | male | 193.04(130.46,274.76) | 514.26(346.05,740.04) | 3.43 | 22.50(19.33,26.09) | 19.42(17.73,21.26) | -0.50(-0.56,-0.43) |
| Bermuda | male | 6.06(4.12,8.50) | 4.72(3.19,6.62) | -0.91 | 35.63(12.98,82.45) | 31.91(9.17,86.88) | -0.42(-0.44,-0.40) |
| Bhutan | male | 50.08(33.17,70.46) | 62.20(41.75,87.69) | 1.29 | 33.07(24.09,44.79) | 27.38(20.95,35.31) | -0.64(-0.65,-0.63) |
| Bolivia (Plurinational State of) | male | 1228.43(862.06,1686.48) | 2214.26(1534.78,3005.06) | 2.19 | 88.04(83.08,93.23) | 73.68(70.63,76.83) | -0.55(-0.61,-0.48) |
| Bosnia and Herzegovina | male | 704.55(488.00,964.99) | 342.74(234.09,467.40) | -2.32 | 57.40(53.21,61.85) | 42.40(37.94,47.29) | -1.22(-1.29,-1.14) |
| Botswana | male | 62.52(41.42,91.05) | 126.70(83.35,181.07) | 2.34 | 23.74(17.98,30.98) | 19.36(16.12,23.11) | -0.71(-0.79,-0.63) |
| Brazil | male | 25394.70(17891.24,34345.58) | 38334.92(26913.04,51449.59) | 1.46 | 69.82(68.95,70.70) | 66.34(65.68,67.01) | -0.15(-0.17,-0.14) |
| Brunei Darussalam | male | 16.70(11.08,24.14) | 29.68(19.93,41.83) | 1.96 | 21.85(12.41,36.94) | 20.41(13.71,29.50) | -0.20(-0.22,-0.17) |
| Bulgaria | male | 1086.87(740.76,1486.64) | 686.08(467.17,938.14) | -1.59 | 50.49(47.51,53.61) | 40.28(37.16,43.63) | -0.80(-0.85,-0.76) |
| Burkina Faso | male | 401.50(266.66,568.18) | 1010.93(650.26,1428.40) | 3.29 | 24.71(22.27,27.36) | 22.16(20.77,23.62) | -0.40(-0.46,-0.34) |
| Burundi | male | 173.98(107.79,249.47) | 404.25(257.05,584.56) | 3.27 | 15.77(13.41,18.50) | 15.63(14.11,17.29) | -0.04(-0.07,-0.02) |
| Cabo Verde | male | 13.28(8.67,19.14) | 27.30(17.95,39.39) | 2.56 | 21.68(10.38,42.83) | 17.24(11.34,25.32) | -0.84(-0.92,-0.76) |
| Cambodia | male | 327.07(214.31,475.02) | 610.32(394.18,858.72) | 2.22 | 17.53(15.60,19.67) | 14.61(13.46,15.84) | -0.67(-0.70,-0.64) |
| Cameroon | male | 398.08(260.67,568.24) | 1113.22(722.73,1577.86) | 3.70 | 19.54(17.62,21.64) | 16.56(15.58,17.60) | -0.54(-0.59,-0.49) |
| Canada | male | 7517.93(5198.78,10303.04) | 7379.82(5079.93,9966.36) | -0.22 | 98.39(96.16,100.65) | 86.74(84.76,88.77) | -0.45(-0.46,-0.44) |
| Central African Republic | male | 283.96(194.27,392.73) | 516.71(356.33,712.52) | 2.07 | 49.21(43.46,55.58) | 43.36(39.62,47.37) | -0.41(-0.43,-0.39) |
| Chad | male | 286.65(194.25,408.02) | 702.14(464.72,996.03) | 3.08 | 26.22(23.16,29.61) | 23.02(21.27,24.88) | -0.46(-0.51,-0.41) |
| Chile | male | 3028.69(2094.36,4128.88) | 3601.03(2481.04,4940.14) | 0.59 | 90.34(87.13,93.66) | 76.41(73.93,78.96) | -0.56(-0.58,-0.53) |
| China | male | 165060.32(116531.48,222614.35) | 158020.59(111151.91,211579.70) | -0.05 | 51.44(51.19,51.70) | 38.48(38.28,38.67) | -1.00(-1.02,-0.99) |
| Colombia | male | 986.49(626.84,1416.88) | 1329.27(862.00,1876.44) | 0.99 | 12.48(11.70,13.30) | 10.88(10.30,11.48) | -0.46(-0.49,-0.43) |
| Comoros | male | 14.39(8.93,20.80) | 26.23(17.47,37.11) | 2.15 | 15.46(8.26,26.98) | 14.18(9.26,20.88) | -0.25(-0.28,-0.23) |
| Congo | male | 194.72(130.67,269.09) | 379.79(252.12,523.84) | 2.38 | 38.20(32.75,44.40) | 29.13(26.27,32.22) | -0.90(-0.94,-0.86) |
| Cook Islands | male | 0.95(0.62,1.35) | 0.71(0.45,1.00) | -0.90 | 20.84(0.40,143.81) | 17.88(0.11,141.98) | -0.52(-0.53,-0.50) |
| Costa Rica | male | 89.73(56.97,131.99) | 130.00(84.90,187.29) | 1.22 | 12.26(9.80,15.22) | 10.75(8.98,12.78) | -0.44(-0.46,-0.41) |
| Croatia | male | 623.62(429.12,859.98) | 398.84(271.68,549.38) | -1.71 | 49.00(45.21,53.04) | 39.61(35.73,43.86) | -0.83(-0.87,-0.79) |
| Cuba | male | 1111.36(753.61,1516.86) | 980.42(672.60,1352.97) | -0.31 | 37.06(34.88,39.33) | 34.85(32.65,37.17) | -0.21(-0.25,-0.17) |
| Cyprus | male | 425.87(294.87,571.56) | 615.26(431.46,831.28) | 1.32 | 205.93(186.82,226.52) | 169.87(156.40,184.42) | -0.66(-0.71,-0.60) |
| Czechia | male | 1366.60(942.41,1900.14) | 1064.61(721.98,1445.28) | -0.86 | 50.16(47.50,52.93) | 39.28(36.80,41.90) | -0.81(-0.86,-0.76) |
| Côte d'Ivoire | male | 563.15(364.60,796.13) | 1181.62(791.11,1686.81) | 2.53 | 20.90(19.16,22.78) | 18.13(17.10,19.22) | -0.45(-0.52,-0.37) |
| Democratic People's Republic of Korea | male | 3309.63(2240.55,4554.82) | 4133.22(2823.82,5702.77) | 1.05 | 65.83(63.60,68.12) | 53.22(51.59,54.89) | -0.76(-0.79,-0.72) |
| Democratic Republic of the Congo | male | 3448.96(2331.17,4810.53) | 7653.74(5153.21,10632.58) | 2.93 | 44.52(42.96,46.12) | 37.96(37.09,38.85) | -0.42(-0.57,-0.26) |
| Denmark | male | 2925.41(2071.69,3946.08) | 2535.66(1763.13,3426.20) | -0.53 | 206.01(198.55,213.69) | 186.69(179.41,194.21) | -0.33(-0.35,-0.31) |
| Djibouti | male | 18.75(12.18,27.64) | 46.31(29.17,66.17) | 3.52 | 15.69(9.08,26.13) | 13.96(10.23,18.67) | -0.43(-0.46,-0.39) |
| Dominica | male | 6.25(4.17,8.51) | 5.47(3.76,7.63) | -0.48 | 35.84(13.09,82.17) | 31.35(10.81,71.53) | -0.49(-0.54,-0.44) |
| Dominican Republic | male | 714.25(482.13,984.67) | 1069.54(733.27,1465.37) | 1.33 | 43.21(39.99,46.65) | 36.95(34.77,39.24) | -0.64(-0.70,-0.58) |
| Ecuador | male | 1580.18(1078.29,2154.39) | 2528.38(1753.58,3513.67) | 1.61 | 68.10(64.70,71.65) | 56.30(54.12,58.55) | -0.63(-0.65,-0.60) |
| Egypt | male | 3324.00(2137.14,4634.92) | 5041.23(3359.53,7142.62) | 1.66 | 25.57(24.70,26.47) | 19.46(18.92,20.00) | -0.73(-0.79,-0.66) |
| El Salvador | male | 158.84(100.69,235.86) | 180.21(117.69,257.65) | 0.35 | 14.17(11.98,16.67) | 12.24(10.50,14.21) | -0.57(-0.61,-0.53) |
| Equatorial Guinea | male | 38.18(26.29,52.16) | 102.36(65.78,143.39) | 3.42 | 49.57(34.58,69.77) | 26.78(21.35,33.53) | -2.37(-2.47,-2.27) |
| Eritrea | male | 109.42(72.36,161.16) | 258.88(161.96,368.71) | 3.19 | 17.19(13.98,21.01) | 15.37(13.51,17.44) | -0.32(-0.39,-0.25) |
| Estonia | male | 184.16(127.29,252.46) | 121.57(83.07,166.00) | -1.35 | 48.04(41.33,55.55) | 38.11(31.42,46.04) | -0.79(-0.81,-0.77) |
| Eswatini | male | 38.27(25.53,55.10) | 60.15(40.07,84.52) | 1.38 | 25.38(17.67,35.60) | 21.19(16.08,27.62) | -0.62(-0.68,-0.56) |
| Ethiopia | male | 1733.36(1192.49,2370.05) | 3770.07(2598.37,5098.59) | 2.61 | 17.17(16.34,18.02) | 15.31(14.81,15.83) | -0.37(-0.40,-0.33) |
| Fiji | male | 43.58(28.91,60.70) | 47.92(31.63,68.51) | 0.37 | 23.34(16.85,31.67) | 20.02(14.75,26.57) | -0.52(-0.55,-0.48) |
| Finland | male | 3226.32(2251.74,4327.18) | 2602.10(1833.64,3461.67) | -0.87 | 229.76(221.76,238.02) | 205.89(197.97,214.08) | -0.38(-0.40,-0.37) |
| France | male | 37874.42(26436.80,51231.74) | 33873.97(23670.14,45869.20) | -0.49 | 253.16(250.60,255.73) | 227.28(224.83,229.75) | -0.41(-0.43,-0.39) |
| Gabon | male | 79.61(52.62,112.23) | 114.63(77.25,158.09) | 1.28 | 36.84(28.90,46.61) | 26.66(21.97,32.11) | -1.04(-1.07,-1.01) |
| Gambia | male | 48.35(31.73,68.63) | 98.48(64.67,140.48) | 2.29 | 23.53(17.19,31.70) | 19.43(15.66,23.94) | -0.68(-0.74,-0.61) |
| Georgia | male | 609.32(411.60,852.55) | 357.34(238.37,487.82) | -1.97 | 47.31(43.60,51.25) | 41.10(36.90,45.69) | -0.51(-0.59,-0.42) |
| Germany | male | 32345.48(22729.26,43596.71) | 28123.09(19644.30,37456.18) | -0.63 | 152.87(151.19,154.56) | 142.59(140.90,144.29) | -0.26(-0.32,-0.21) |
| Ghana | male | 782.76(500.36,1109.86) | 1618.78(1041.70,2273.77) | 2.51 | 24.86(23.11,26.73) | 21.16(20.12,22.24) | -0.57(-0.62,-0.53) |
| Greece | male | 5359.24(3732.55,7167.61) | 4530.46(3142.84,6035.74) | -0.59 | 208.88(203.32,214.56) | 183.96(178.41,189.66) | -0.43(-0.47,-0.40) |
| Greenland | male | 23.29(16.26,31.54) | 15.18(10.74,20.42) | -1.38 | 122.50(77.30,189.57) | 107.07(59.90,180.30) | -0.54(-0.57,-0.52) |
| Grenada | male | 7.31(4.83,10.35) | 9.17(6.24,12.73) | 0.86 | 39.44(15.47,88.16) | 32.78(15.02,63.43) | -0.53(-0.61,-0.46) |
| Guam | male | 7.48(4.89,10.78) | 7.06(4.64,9.93) | -0.36 | 18.81(7.66,40.33) | 16.54(6.63,34.78) | -0.52(-0.57,-0.46) |
| Guatemala | male | 252.12(162.08,362.13) | 574.33(369.56,833.58) | 2.98 | 15.83(13.88,18.00) | 13.16(12.07,14.33) | -0.65(-0.69,-0.62) |
| Guinea | male | 275.17(180.07,391.00) | 513.12(322.37,725.21) | 1.95 | 23.67(20.91,26.73) | 20.77(18.95,22.73) | -0.45(-0.52,-0.38) |
| Guinea-Bissau | male | 45.24(29.48,64.21) | 86.49(54.55,124.71) | 2.23 | 23.57(16.93,32.28) | 20.37(16.15,25.53) | -0.52(-0.59,-0.46) |
| Guyana | male | 68.80(45.73,94.32) | 64.68(43.47,89.43) | -0.34 | 36.53(28.16,46.94) | 32.19(24.76,41.24) | -0.40(-0.43,-0.38) |
| Haiti | male | 659.40(461.66,896.98) | 1338.82(892.52,1870.17) | 2.48 | 49.19(45.45,53.19) | 44.24(41.88,46.72) | -0.37(-0.39,-0.35) |
| Honduras | male | 146.71(94.84,212.24) | 319.65(203.59,461.87) | 2.69 | 15.65(13.14,18.55) | 13.56(12.09,15.18) | -0.52(-0.56,-0.47) |
| Hungary | male | 1343.94(926.10,1823.00) | 984.22(670.49,1342.01) | -1.18 | 50.93(48.21,53.77) | 40.39(37.80,43.14) | -0.83(-0.87,-0.79) |
| Iceland | male | 154.44(107.54,208.44) | 176.81(123.37,241.16) | 0.38 | 229.83(194.89,269.55) | 202.57(173.64,235.42) | -0.44(-0.45,-0.43) |
| India | male | 68282.28(47643.57,91203.55) | 122956.63(85140.50,164609.51) | 2.08 | 32.26(32.02,32.51) | 31.85(31.67,32.03) | -0.05(-0.13,0.02) |
| Indonesia | male | 3172.51(2202.32,4321.52) | 4896.04(3363.18,6683.36) | 1.47 | 7.37(7.11,7.63) | 6.60(6.41,6.79) | -0.41(-0.46,-0.37) |
| Iran (Islamic Republic of) | male | 5847.28(4055.85,7865.58) | 8745.76(6143.95,11701.62) | 1.57 | 47.68(46.41,48.98) | 34.49(33.76,35.24) | -1.18(-1.24,-1.11) |
| Iraq | male | 2089.04(1433.92,2918.68) | 4713.92(3131.66,6677.53) | 2.58 | 56.60(54.12,59.17) | 41.50(40.31,42.72) | -1.11(-1.17,-1.05) |
| Ireland | male | 1940.14(1350.21,2620.02) | 2307.29(1617.30,3143.12) | 0.86 | 219.11(209.44,229.11) | 187.20(179.47,195.21) | -0.52(-0.55,-0.49) |
| Israel | male | 2242.05(1549.17,3010.62) | 3839.95(2671.22,5146.29) | 1.77 | 191.40(183.48,199.59) | 171.78(166.38,177.31) | -0.34(-0.36,-0.32) |
| Italy | male | 26621.22(18608.66,35791.26) | 23944.55(16891.14,32049.38) | -0.05 | 182.69(180.49,184.90) | 169.62(167.39,171.89) | -0.14(-0.21,-0.07) |
| Jamaica | male | 207.03(135.00,283.34) | 249.30(165.73,348.90) | 0.67 | 38.54(33.30,44.45) | 33.30(29.28,37.73) | -0.50(-0.52,-0.48) |
| Japan | male | 8345.36(5915.47,11244.84) | 7148.72(5106.76,9632.56) | -0.44 | 24.41(23.88,24.95) | 24.79(24.19,25.40) | 0.12(0.10,0.13) |
| Jordan | male | 355.09(238.81,494.11) | 1104.64(736.12,1530.09) | 4.01 | 42.00(37.44,47.02) | 32.59(30.69,34.57) | -0.92(-0.94,-0.90) |
| Kazakhstan | male | 2077.50(1434.86,2849.22) | 1960.99(1335.71,2650.96) | -0.08 | 52.62(50.33,54.99) | 41.87(40.02,43.79) | -0.79(-0.82,-0.76) |
| Kenya | male | 642.99(453.36,874.79) | 1587.65(1097.34,2139.43) | 3.19 | 13.84(12.73,15.03) | 12.84(12.20,13.51) | -0.18(-0.22,-0.14) |
| Kiribati | male | 3.78(2.53,5.35) | 6.10(4.02,8.55) | 1.99 | 23.10(5.74,65.83) | 22.11(8.08,50.13) | -0.02(-0.06,0.02) |
| Kuwait | male | 220.19(147.35,309.94) | 475.66(316.68,656.96) | 3.13 | 34.38(29.83,39.54) | 28.03(25.41,30.93) | -0.85(-0.90,-0.80) |
| Kyrgyzstan | male | 537.75(363.01,731.71) | 770.14(516.81,1055.24) | 1.32 | 55.02(50.24,60.20) | 46.84(43.57,50.31) | -0.49(-0.53,-0.46) |
| Lao People's Democratic Republic | male | 148.58(95.84,210.06) | 281.18(177.74,395.83) | 2.21 | 18.14(15.28,21.43) | 15.03(13.31,16.92) | -0.70(-0.73,-0.66) |
| Latvia | male | 304.28(209.05,414.83) | 168.23(114.74,229.69) | -1.97 | 47.06(41.91,52.70) | 38.23(32.49,44.84) | -0.71(-0.74,-0.67) |
| Lebanon | male | 310.83(212.61,423.85) | 443.21(302.23,622.34) | 1.33 | 44.49(39.59,49.84) | 33.06(30.02,36.34) | -1.08(-1.12,-1.03) |
| Lesotho | male | 109.25(72.10,153.55) | 134.97(89.21,190.30) | 0.58 | 28.56(23.32,34.72) | 23.75(19.84,28.31) | -0.62(-0.67,-0.56) |
| Liberia | male | 79.07(52.89,111.27) | 224.55(148.75,314.87) | 3.69 | 21.22(16.74,26.57) | 18.80(16.40,21.46) | -0.58(-0.64,-0.52) |
| Libya | male | 412.97(283.83,575.07) | 681.13(462.35,938.51) | 2.24 | 42.02(37.96,46.44) | 30.84(28.55,33.27) | -1.02(-1.14,-0.91) |
| Lithuania | male | 427.06(290.03,583.58) | 235.77(158.71,324.11) | -2.10 | 47.04(42.67,51.73) | 36.96(32.31,42.17) | -0.85(-0.88,-0.83) |
| Luxembourg | male | 206.24(143.52,280.83) | 301.13(210.76,407.08) | 1.27 | 193.64(167.83,222.89) | 178.92(158.82,201.26) | -0.28(-0.29,-0.27) |
| Madagascar | male | 410.31(266.22,591.34) | 989.61(633.29,1396.93) | 3.09 | 16.70(15.05,18.50) | 15.84(14.84,16.89) | -0.16(-0.21,-0.12) |
| Malawi | male | 333.89(217.86,477.57) | 640.58(404.30,925.07) | 2.23 | 17.10(15.25,19.15) | 15.62(14.39,16.94) | -0.32(-0.35,-0.29) |
| Malaysia | male | 524.94(350.60,756.80) | 925.23(612.77,1348.58) | 1.83 | 12.34(11.29,13.47) | 10.25(9.60,10.94) | -0.66(-0.70,-0.61) |
| Maldives | male | 6.56(4.37,9.22) | 25.53(16.41,37.70) | 4.86 | 15.47(5.68,35.31) | 12.17(7.68,19.14) | -0.83(-0.89,-0.77) |
| Mali | male | 343.63(218.96,485.91) | 754.78(478.54,1085.68) | 2.79 | 21.18(18.96,23.60) | 17.37(16.10,18.72) | -0.71(-0.75,-0.67) |
| Malta | male | 210.16(148.54,283.15) | 194.58(136.34,258.98) | -0.37 | 208.51(181.00,239.34) | 177.88(153.19,206.21) | -0.52(-0.54,-0.50) |
| Marshall Islands | male | 2.33(1.50,3.36) | 3.22(2.08,4.55) | 0.97 | 25.37(3.42,104.62) | 21.19(4.69,62.05) | -0.59(-0.61,-0.57) |
| Mauritania | male | 91.79(58.53,131.04) | 163.15(102.22,232.77) | 1.96 | 21.92(17.54,27.19) | 18.53(15.72,21.73) | -0.54(-0.59,-0.48) |
| Mauritius | male | 40.30(25.96,57.37) | 40.68(26.74,58.13) | -0.16 | 13.82(9.81,19.11) | 11.87(8.48,16.24) | -0.59(-0.62,-0.56) |
| Mexico | male | 2737.64(1900.16,3685.67) | 3992.98(2800.43,5333.63) | 1.28 | 14.05(13.51,14.61) | 12.36(11.98,12.75) | -0.43(-0.46,-0.40) |
| Micronesia (Federated States of) | male | 5.14(3.28,7.10) | 5.29(3.44,7.43) | -0.05 | 23.45(7.43,59.81) | 20.20(6.76,47.19) | -0.49(-0.51,-0.47) |
| Monaco | male | 13.59(9.41,18.39) | 12.77(8.76,17.14) | -0.08 | 183.75(97.13,334.62) | 168.81(87.07,301.61) | -0.28(-0.29,-0.27) |
| Mongolia | male | 280.39(190.18,392.40) | 422.18(292.15,584.90) | 1.62 | 59.14(52.08,67.01) | 45.77(41.47,50.45) | -0.88(-0.90,-0.86) |
| Montenegro | male | 75.89(51.01,103.56) | 60.68(41.40,84.95) | -1.04 | 47.32(37.23,59.47) | 39.09(29.81,50.54) | -0.80(-0.87,-0.72) |
| Morocco | male | 2990.94(2001.68,4136.73) | 3895.20(2697.55,5345.34) | 0.86 | 52.09(50.20,54.05) | 40.57(39.30,41.86) | -0.87(-0.88,-0.85) |
| Mozambique | male | 466.89(309.71,656.62) | 994.86(644.38,1434.82) | 2.53 | 19.17(17.44,21.03) | 16.91(15.83,18.04) | -0.45(-0.48,-0.41) |
| Myanmar | male | 1620.98(1043.84,2301.47) | 1998.78(1309.49,2891.24) | 0.56 | 17.78(16.90,18.70) | 14.48(13.85,15.13) | -0.81(-0.86,-0.76) |
| Namibia | male | 70.00(45.97,99.50) | 118.46(77.39,167.68) | 1.77 | 23.29(17.94,29.90) | 19.93(16.46,23.97) | -0.53(-0.57,-0.49) |
| Nauru | male | 0.49(0.32,0.69) | 0.48(0.32,0.68) | -0.11 | 20.29(0.01,228.00) | 18.02(0.01,206.18) | -0.33(-0.44,-0.23) |
| Nepal | male | 1215.22(807.82,1692.58) | 1526.52(1021.64,2173.99) | 0.62 | 29.21(27.56,30.93) | 21.59(20.50,22.72) | -1.17(-1.23,-1.10) |
| Netherlands | male | 8675.54(6103.32,11612.60) | 7298.01(5093.60,9876.93) | -0.65 | 204.30(200.01,208.67) | 183.91(179.65,188.24) | -0.33(-0.35,-0.32) |
| New Zealand | male | 1435.93(993.07,1938.19) | 1412.25(981.71,1905.13) | -0.02 | 159.40(151.26,167.88) | 142.28(134.89,149.97) | -0.38(-0.39,-0.36) |
| Nicaragua | male | 115.48(73.11,171.84) | 217.78(142.67,306.26) | 2.17 | 14.99(12.26,18.22) | 12.88(11.21,14.74) | -0.50(-0.53,-0.47) |
| Niger | male | 419.97(280.64,596.48) | 1090.92(708.92,1522.39) | 3.24 | 27.37(24.76,30.21) | 25.61(24.01,27.29) | -0.21(-0.28,-0.14) |
| Nigeria | male | 6052.73(4237.61,8152.65) | 9651.57(6717.28,13068.41) | 1.53 | 30.00(29.24,30.77) | 21.39(20.95,21.83) | -1.25(-1.35,-1.14) |
| Niue | male | 0.11(0.07,0.16) | 0.08(0.05,0.10) | -1.66 | 21.91(0.00,857.05) | 18.84(0.00,1156.87) | -0.60(-0.62,-0.57) |
| North Macedonia | male | 272.26(185.05,372.51) | 241.83(164.96,336.71) | -0.47 | 51.46(45.52,57.99) | 40.95(35.86,46.66) | -0.89(-0.94,-0.84) |
| Northern Mariana Islands | male | 2.93(1.92,4.19) | 2.06(1.39,2.96) | -1.07 | 18.87(3.71,62.00) | 17.85(1.72,87.17) | -0.10(-0.15,-0.06) |
| Norway | male | 1765.81(1255.00,2366.12) | 1933.77(1350.76,2585.99) | 0.29 | 155.04(147.87,162.49) | 142.71(136.33,149.33) | -0.31(-0.33,-0.30) |
| Oman | male | 273.11(185.13,378.58) | 646.91(433.19,906.79) | 3.38 | 44.22(38.98,50.06) | 28.43(26.05,31.05) | -1.50(-1.62,-1.38) |
| Pakistan | male | 7978.48(5546.08,11010.68) | 14694.65(10126.92,20196.74) | 2.09 | 32.59(31.85,33.33) | 27.23(26.78,27.68) | -0.66(-0.69,-0.62) |
| Palau | male | 0.81(0.55,1.15) | 1.03(0.65,1.44) | 0.25 | 19.41(0.22,145.23) | 17.06(0.37,125.97) | -0.43(-0.45,-0.41) |
| Palestine | male | 209.51(143.71,288.87) | 445.88(309.27,609.81) | 2.66 | 53.07(45.56,61.65) | 36.82(33.41,40.51) | -1.27(-1.31,-1.24) |
| Panama | male | 71.65(45.84,105.14) | 112.48(73.94,159.81) | 1.56 | 12.03(9.37,15.28) | 10.61(8.74,12.77) | -0.39(-0.41,-0.37) |
| Papua New Guinea | male | 257.26(171.00,361.40) | 604.07(396.87,841.11) | 3.13 | 27.62(24.27,31.34) | 24.70(22.76,26.78) | -0.37(-0.39,-0.34) |
| Paraguay | male | 674.80(467.66,918.45) | 1313.89(894.29,1798.47) | 2.34 | 73.96(68.38,79.91) | 70.82(67.02,74.79) | -0.14(-0.17,-0.11) |
| Peru | male | 3673.63(2529.99,5085.30) | 5492.38(3711.75,7552.95) | 1.39 | 73.35(70.94,75.83) | 61.97(60.34,63.63) | -0.57(-0.61,-0.52) |
| Philippines | male | 2048.91(1451.42,2749.23) | 3796.95(2660.06,5145.09) | 2.24 | 14.29(13.66,14.94) | 13.09(12.67,13.51) | -0.20(-0.26,-0.14) |
| Poland | male | 5049.40(3592.15,6749.02) | 4564.55(3197.94,6139.50) | -0.47 | 51.91(50.47,53.39) | 46.21(44.83,47.63) | -0.32(-0.36,-0.28) |
| Portugal | male | 5537.36(3807.18,7384.19) | 4857.60(3364.63,6563.04) | -0.45 | 225.48(219.58,231.50) | 193.87(188.26,199.62) | -0.49(-0.51,-0.47) |
| Puerto Rico | male | 283.02(187.27,384.96) | 226.71(150.40,310.04) | -0.78 | 32.28(28.62,36.29) | 28.71(25.07,32.75) | -0.42(-0.43,-0.40) |
| Qatar | male | 81.89(55.51,113.99) | 498.36(332.23,700.54) | 8.17 | 36.68(28.61,47.07) | 27.77(25.02,30.96) | -0.94(-0.98,-0.91) |
| Republic of Korea | male | 3472.30(2317.29,4968.13) | 3589.92(2365.91,4961.31) | 0.11 | 27.11(26.21,28.04) | 24.22(23.40,25.06) | -0.35(-0.40,-0.29) |
| Republic of Moldova | male | 523.54(359.09,718.42) | 399.43(271.21,552.87) | -1.08 | 49.08(44.93,53.53) | 40.64(36.64,45.02) | -0.64(-0.72,-0.56) |
| Romania | male | 2849.62(1944.90,3937.00) | 1887.03(1299.92,2589.02) | -1.54 | 49.74(47.92,51.61) | 39.29(37.46,41.20) | -0.88(-0.94,-0.82) |
| Russian Federation | male | 16216.38(11521.31,21528.40) | 13912.48(9768.74,18504.41) | -0.71 | 43.43(42.75,44.11) | 38.49(37.83,39.16) | -0.38(-0.43,-0.34) |
| Rwanda | male | 196.91(125.49,287.34) | 376.31(241.83,539.07) | 2.88 | 13.71(11.74,15.98) | 12.48(11.21,13.87) | -0.34(-0.37,-0.31) |
| Saint Kitts and Nevis | male | 3.28(2.21,4.64) | 5.00(3.32,7.00) | 1.59 | 35.28(7.04,125.40) | 30.63(9.80,74.77) | -0.47(-0.49,-0.44) |
| Saint Lucia | male | 11.60(7.90,16.03) | 15.77(10.67,21.89) | 1.19 | 37.98(18.76,71.09) | 32.94(18.66,54.36) | -0.45(-0.51,-0.40) |
| Saint Vincent and the Grenadines | male | 9.99(6.67,13.82) | 10.08(6.73,14.07) | 0.00 | 39.57(18.05,79.67) | 34.27(16.43,63.71) | -0.47(-0.50,-0.44) |
| Samoa | male | 8.10(5.35,11.28) | 10.92(7.09,15.38) | 1.03 | 22.84(9.32,48.53) | 21.62(10.66,39.49) | -0.13(-0.16,-0.09) |
| San Marino | male | 11.67(8.03,15.74) | 13.02(8.94,17.59) | 0.44 | 189.15(96.53,335.78) | 173.10(91.04,302.71) | -0.29(-0.31,-0.27) |
| Sao Tome and Principe | male | 4.36(2.80,6.19) | 8.39(5.10,12.08) | 2.31 | 19.43(5.07,55.50) | 16.04(7.02,32.12) | -0.72(-0.76,-0.67) |
| Saudi Arabia | male | 2230.56(1523.62,3077.78) | 4405.27(3000.99,6193.66) | 2.54 | 48.32(46.31,50.41) | 28.51(27.65,29.40) | -1.71(-1.83,-1.59) |
| Senegal | male | 331.87(218.65,468.58) | 684.92(445.63,979.97) | 2.45 | 22.84(20.35,25.57) | 20.00(18.48,21.62) | -0.46(-0.52,-0.40) |
| Serbia | male | 1231.23(849.17,1683.09) | 862.90(574.20,1205.52) | -1.63 | 51.87(49.01,54.87) | 40.44(37.75,43.28) | -0.93(-0.98,-0.88) |
| Seychelles | male | 2.15(1.43,3.04) | 3.40(2.20,4.85) | 1.80 | 12.27(1.53,48.87) | 10.73(2.45,33.37) | -0.43(-0.48,-0.37) |
| Sierra Leone | male | 167.31(106.42,233.10) | 384.77(246.43,556.02) | 3.44 | 22.43(19.11,26.21) | 19.95(17.95,22.13) | -0.43(-0.48,-0.37) |
| Singapore | male | 200.07(127.81,282.63) | 343.75(230.65,482.69) | 2.08 | 20.92(18.10,24.07) | 19.24(17.10,21.63) | -0.25(-0.29,-0.22) |
| Slovakia | male | 701.48(483.91,968.79) | 574.12(389.58,798.27) | -0.82 | 51.78(48.00,55.78) | 39.69(36.38,43.28) | -0.92(-0.97,-0.87) |
| Slovenia | male | 248.26(167.37,339.11) | 192.64(132.31,264.50) | -0.98 | 46.99(41.30,53.29) | 37.99(32.56,44.23) | -0.80(-0.84,-0.75) |
| Solomon Islands | male | 19.41(12.96,26.98) | 38.99(26.21,53.87) | 2.44 | 28.63(16.96,45.90) | 24.97(17.70,34.32) | -0.44(-0.46,-0.41) |
| Somalia | male | 291.81(184.55,419.56) | 926.65(611.65,1324.06) | 3.99 | 19.44(17.22,21.89) | 20.04(18.72,21.43) | 0.15(0.11,0.19) |
| South Africa | male | 1941.71(1343.89,2636.46) | 3007.59(2105.70,3975.60) | 1.44 | 21.90(20.91,22.93) | 19.68(18.98,20.40) | -0.41(-0.43,-0.38) |
| South Sudan | male | 208.86(131.23,303.52) | 278.12(175.32,398.43) | 1.17 | 15.40(13.29,17.80) | 14.65(12.91,16.57) | -0.16(-0.19,-0.13) |
| Spain | male | 18612.84(12993.34,25270.00) | 19771.81(13797.02,26745.37) | 0.61 | 192.02(189.27,194.81) | 169.35(166.86,171.88) | -0.39(-0.41,-0.36) |
| Sri Lanka | male | 1167.97(794.30,1610.51) | 1145.97(746.59,1619.28) | -0.12 | 26.50(24.99,28.08) | 20.97(19.77,22.23) | -0.75(-0.78,-0.72) |
| Sudan | male | 2106.43(1445.49,2912.21) | 3507.98(2345.62,4816.81) | 1.63 | 51.47(49.23,53.79) | 36.82(35.58,38.09) | -1.16(-1.18,-1.14) |
| Suriname | male | 33.67(23.21,47.20) | 45.08(29.69,63.24) | 1.28 | 35.47(24.19,50.60) | 31.31(22.83,41.99) | -0.45(-0.47,-0.43) |
| Sweden | male | 3036.93(2159.59,4038.45) | 3094.98(2210.86,4152.64) | 0.10 | 134.39(129.59,139.34) | 127.85(123.33,132.51) | -0.17(-0.19,-0.14) |
| Switzerland | male | 3513.42(2408.03,4711.22) | 3698.01(2638.75,4987.39) | 0.25 | 180.14(174.17,186.28) | 168.33(162.82,174.01) | -0.23(-0.24,-0.23) |
| Syrian Arab Republic | male | 1287.58(863.35,1769.00) | 1233.63(841.89,1681.40) | 0.05 | 49.94(47.11,52.91) | 37.28(35.07,39.62) | -1.04(-1.08,-0.99) |
| Taiwan (Province of China) | male | 2759.98(1872.51,3796.33) | 2476.45(1690.98,3423.08) | -0.47 | 50.51(48.61,52.47) | 37.99(36.47,39.56) | -0.93(-0.96,-0.90) |
| Tajikistan | male | 641.15(442.86,885.32) | 1159.03(780.00,1619.24) | 2.26 | 58.14(53.44,63.21) | 47.92(45.16,50.81) | -0.65(-0.72,-0.58) |
| Thailand | male | 2002.46(1316.13,2848.52) | 2260.97(1451.11,3331.97) | 0.46 | 13.64(13.04,14.26) | 11.76(11.27,12.27) | -0.51(-0.54,-0.48) |
| Timor-Leste | male | 30.83(20.25,44.53) | 40.65(27.03,58.95) | 0.63 | 17.01(11.43,24.61) | 13.65(9.67,18.84) | -1.00(-1.10,-0.89) |
| Togo | male | 147.74(97.33,212.04) | 331.17(210.53,479.29) | 2.83 | 21.02(17.59,25.01) | 18.25(16.31,20.37) | -0.43(-0.50,-0.36) |
| Tokelau | male | 0.07(0.05,0.10) | 0.06(0.04,0.09) | -0.82 | 23.01(0.00,1723.54) | 18.99(0.00,1411.79) | -0.68(-0.70,-0.65) |
| Tonga | male | 4.64(3.09,6.56) | 4.79(3.16,6.71) | 0.35 | 24.50(6.98,64.02) | 21.24(6.57,51.99) | -0.42(-0.45,-0.39) |
| Trinidad and Tobago | male | 99.09(66.99,139.95) | 104.94(71.85,145.23) | 0.10 | 32.65(26.48,39.95) | 28.80(23.48,35.07) | -0.47(-0.51,-0.43) |
| Tunisia | male | 884.89(606.83,1223.42) | 1086.09(724.19,1519.03) | 0.63 | 46.29(43.19,49.57) | 35.18(33.10,37.36) | -0.94(-0.99,-0.89) |
| Turkey | male | 10031.67(6786.66,13868.58) | 12754.31(8832.64,17242.43) | 0.74 | 69.82(68.44,71.23) | 53.07(52.15,54.01) | -0.93(-0.96,-0.90) |
| Turkmenistan | male | 422.74(289.94,578.21) | 554.89(384.76,772.98) | 0.89 | 52.53(47.33,58.23) | 39.87(36.61,43.34) | -1.01(-1.04,-0.99) |
| Tuvalu | male | 0.46(0.30,0.66) | 0.62(0.40,0.87) | 1.08 | 23.60(0.01,271.28) | 20.10(0.07,169.79) | -0.53(-0.55,-0.50) |
| Uganda | male | 537.39(336.33,775.29) | 1181.72(762.30,1690.42) | 2.65 | 16.07(14.66,17.59) | 13.95(13.12,14.82) | -0.62(-0.66,-0.58) |
| Ukraine | male | 5696.55(3905.03,7781.27) | 4332.07(2924.03,5899.75) | -1.04 | 45.90(44.72,47.12) | 39.45(38.25,40.70) | -0.54(-0.58,-0.49) |
| United Arab Emirates | male | 311.31(209.04,435.96) | 1636.24(1096.13,2284.86) | 7.10 | 35.28(31.20,39.89) | 26.00(24.17,28.00) | -1.05(-1.13,-0.96) |
| United Kingdom | male | 30457.05(21382.85,40816.05) | 29582.12(20839.97,39517.71) | 0.02 | 208.78(206.43,211.15) | 182.85(180.75,184.98) | -0.41(-0.43,-0.40) |
| United Republic of Tanzania | male | 778.65(495.84,1130.60) | 1707.06(1077.83,2457.08) | 2.72 | 15.69(14.56,16.90) | 14.18(13.50,14.89) | -0.31(-0.35,-0.27) |
| United States of America | male | 72943.94(51470.72,97352.03) | 70304.12(49569.21,93462.62) | -0.44 | 106.58(105.81,107.36) | 90.11(89.44,90.78) | -0.79(-0.88,-0.70) |
| United States Virgin Islands | male | 8.74(5.82,12.27) | 6.05(4.08,8.50) | -1.34 | 32.97(14.73,64.71) | 28.29(10.24,63.86) | -0.59(-0.63,-0.55) |
| Uruguay | male | 641.84(444.55,862.46) | 647.05(442.64,883.35) | -0.01 | 87.99(81.31,95.07) | 78.34(72.41,84.65) | -0.39(-0.41,-0.37) |
| Uzbekistan | male | 2601.90(1773.65,3599.33) | 3937.51(2712.39,5292.56) | 1.46 | 57.74(55.38,60.18) | 44.19(42.81,45.60) | -0.93(-0.94,-0.91) |
| Vanuatu | male | 8.76(5.81,12.00) | 16.24(10.69,22.87) | 2.25 | 27.13(12.03,53.71) | 23.97(13.69,39.30) | -0.43(-0.45,-0.42) |
| Venezuela (Bolivarian Republic of) | male | 559.62(353.42,804.00) | 813.89(528.58,1172.84) | 1.64 | 12.32(11.29,13.42) | 11.37(10.60,12.19) | -0.23(-0.28,-0.18) |
| Viet Nam | male | 2214.61(1453.74,3187.30) | 3589.29(2327.61,5158.34) | 1.67 | 15.97(15.27,16.70) | 12.96(12.53,13.39) | -0.77(-0.80,-0.73) |
| Yemen | male | 1378.49(929.76,1903.71) | 3014.25(2050.82,4185.48) | 2.58 | 55.08(52.12,58.17) | 40.68(39.21,42.20) | -1.23(-1.29,-1.16) |
| Zambia | male | 244.40(153.78,357.04) | 577.74(370.64,831.26) | 3.10 | 15.55(13.55,17.80) | 13.61(12.49,14.80) | -0.45(-0.49,-0.40) |
| Zimbabwe | male | 495.31(322.32,720.86) | 788.36(512.33,1090.94) | 1.54 | 24.11(21.92,26.49) | 22.91(21.30,24.61) | 0.01(-0.10,0.11) |

**Supplementary Table 3. Age distribution of DALY rate for global psoriasis burden of young adults in different countries in 2019.**

| 2019 DALY rate | 15-19 years | 20-24 years | 25-29 years | 30-34 years | 35-39 years | 40-44 years | 45-49 years |
| --- | --- | --- | --- | --- | --- | --- | --- |
| Afghanistan | 31.53 | 39.11 | 48.46 | 57.14 | 63.26 | 67.34 | 72.61 |
| Albania | 33.26 | 34.93 | 35.41 | 40.27 | 45.36 | 50.78 | 56.78 |
| Algeria | 21.95 | 26.82 | 33.60 | 39.52 | 43.38 | 46.37 | 50.01 |
| American Samoa | 11.95 | 13.58 | 15.18 | 17.75 | 21.79 | 26.37 | 29.07 |
| Andorra | 103.36 | 121.39 | 149.73 | 177.68 | 200.14 | 219.66 | 239.51 |
| Angola | 28.85 | 30.83 | 34.57 | 38.70 | 41.76 | 45.25 | 48.43 |
| Antigua and Barbuda | 23.53 | 24.72 | 27.76 | 32.06 | 35.87 | 39.63 | 43.50 |
| Argentina | 52.96 | 60.29 | 73.27 | 86.65 | 96.86 | 105.41 | 113.64 |
| Armenia | 31.25 | 33.37 | 34.21 | 38.25 | 43.33 | 48.50 | 54.30 |
| Australia | 88.04 | 102.31 | 124.94 | 146.29 | 160.13 | 170.94 | 182.84 |
| Austria | 111.96 | 131.60 | 161.97 | 192.33 | 217.83 | 238.47 | 260.44 |
| Azerbaijan | 31.71 | 33.85 | 34.54 | 38.23 | 44.39 | 49.81 | 55.32 |
| Bahamas | 23.05 | 24.39 | 27.73 | 31.60 | 35.62 | 38.98 | 43.41 |
| Bahrain | 19.71 | 24.09 | 29.96 | 35.13 | 38.30 | 41.28 | 44.62 |
| Bangladesh | 18.38 | 20.55 | 23.33 | 26.75 | 29.88 | 32.64 | 35.14 |
| Barbados | 22.57 | 24.06 | 27.16 | 31.19 | 34.73 | 38.59 | 42.26 |
| Belarus | 33.47 | 34.42 | 34.20 | 38.99 | 44.15 | 49.28 | 55.20 |
| Belgium | 114.43 | 134.59 | 165.93 | 196.13 | 220.88 | 242.33 | 264.94 |
| Belize | 27.17 | 29.15 | 32.59 | 37.43 | 41.99 | 46.21 | 50.74 |
| Benin | 14.02 | 15.94 | 17.86 | 20.25 | 22.32 | 23.94 | 26.11 |
| Bermuda | 23.96 | 25.43 | 28.55 | 32.81 | 36.68 | 40.76 | 44.75 |
| Bhutan | 19.78 | 21.63 | 24.68 | 28.69 | 31.58 | 34.77 | 37.98 |
| Bolivia (Plurinational State of) | 56.09 | 58.69 | 64.94 | 75.21 | 84.21 | 93.53 | 103.68 |
| Bosnia and Herzegovina | 32.90 | 34.53 | 34.79 | 39.32 | 44.88 | 49.97 | 55.97 |
| Botswana | 14.52 | 16.03 | 18.12 | 20.31 | 21.96 | 23.76 | 25.58 |
| Brazil | 49.98 | 52.88 | 58.77 | 67.36 | 76.01 | 84.50 | 93.46 |
| Brunei Darussalam | 13.46 | 15.14 | 18.69 | 21.58 | 24.29 | 26.64 | 28.40 |
| Bulgaria | 30.98 | 32.74 | 32.77 | 37.77 | 42.50 | 47.59 | 53.62 |
| Burkina Faso | 16.23 | 17.93 | 20.31 | 23.01 | 25.40 | 27.71 | 29.88 |
| Burundi | 11.49 | 13.17 | 14.95 | 16.70 | 18.36 | 19.43 | 21.01 |
| Cabo Verde | 12.49 | 13.86 | 15.92 | 18.11 | 19.90 | 21.34 | 23.23 |
| Cambodia | 10.57 | 11.66 | 13.15 | 15.04 | 17.99 | 21.26 | 23.51 |
| Cameroon | 11.82 | 13.40 | 15.17 | 17.16 | 19.20 | 20.62 | 22.49 |
| Canada | 56.96 | 62.98 | 81.45 | 97.69 | 109.51 | 117.61 | 126.16 |
| Central African Republic | 33.39 | 36.17 | 40.43 | 45.27 | 49.41 | 53.04 | 57.36 |
| Chad | 16.68 | 18.80 | 20.97 | 24.25 | 26.44 | 28.53 | 31.09 |
| Chile | 50.89 | 57.76 | 69.83 | 83.85 | 93.20 | 101.48 | 109.35 |
| China | 23.40 | 26.79 | 29.81 | 35.10 | 44.58 | 56.84 | 64.76 |
| Colombia | 8.09 | 8.81 | 10.01 | 11.20 | 12.35 | 13.97 | 15.20 |
| Comoros | 10.45 | 11.71 | 13.29 | 15.24 | 16.54 | 17.70 | 19.13 |
| Congo | 22.67 | 24.56 | 26.98 | 29.94 | 32.80 | 35.50 | 38.24 |
| Cook Islands | 11.83 | 13.24 | 14.70 | 17.72 | 21.42 | 25.53 | 28.43 |
| Costa Rica | 8.03 | 8.65 | 9.94 | 11.06 | 12.37 | 13.67 | 14.89 |
| Croatia | 30.77 | 32.16 | 32.48 | 36.61 | 42.24 | 46.45 | 52.34 |
| Cuba | 26.15 | 28.01 | 31.08 | 35.79 | 40.21 | 44.50 | 48.48 |
| Cyprus | 103.07 | 122.37 | 149.68 | 178.20 | 200.29 | 217.81 | 236.69 |
| Czechia | 30.45 | 31.63 | 32.14 | 36.87 | 41.64 | 46.30 | 52.21 |
| Côte d'Ivoire | 13.21 | 14.65 | 16.60 | 19.05 | 20.83 | 22.63 | 24.42 |
| Democratic People's Republic of Korea | 31.08 | 35.68 | 40.02 | 47.76 | 60.09 | 76.11 | 86.21 |
| Democratic Republic of the Congo | 29.40 | 31.37 | 35.34 | 39.69 | 42.80 | 46.30 | 50.18 |
| Denmark | 113.15 | 134.79 | 164.03 | 195.85 | 219.51 | 240.69 | 262.76 |
| Djibouti | 10.29 | 11.34 | 13.43 | 14.73 | 16.32 | 17.32 | 18.97 |
| Dominica | 23.61 | 24.95 | 28.10 | 32.04 | 36.27 | 39.57 | 43.52 |
| Dominican Republic | 27.55 | 29.96 | 33.04 | 38.30 | 42.19 | 46.95 | 51.43 |
| Ecuador | 43.04 | 44.38 | 49.58 | 57.24 | 64.81 | 71.91 | 79.41 |
| Egypt | 12.22 | 14.99 | 18.64 | 22.18 | 24.05 | 25.52 | 27.52 |
| El Salvador | 9.13 | 9.99 | 10.90 | 12.57 | 14.07 | 15.54 | 17.16 |
| Equatorial Guinea | 20.67 | 22.24 | 24.77 | 27.81 | 29.87 | 32.54 | 35.03 |
| Eritrea | 11.28 | 12.91 | 14.60 | 16.53 | 17.77 | 18.95 | 20.70 |
| Estonia | 31.65 | 32.20 | 32.41 | 36.96 | 42.01 | 46.72 | 53.22 |
| Eswatini | 16.04 | 17.69 | 19.85 | 22.21 | 24.19 | 25.80 | 28.06 |
| Ethiopia | 11.24 | 12.65 | 14.37 | 16.18 | 17.75 | 19.17 | 20.57 |
| Fiji | 13.02 | 14.80 | 16.52 | 19.84 | 23.99 | 28.89 | 32.45 |
| Finland | 125.46 | 147.93 | 180.29 | 215.86 | 243.25 | 266.99 | 290.36 |
| France | 141.30 | 167.34 | 204.38 | 242.12 | 274.40 | 299.26 | 328.49 |
| Gabon | 20.72 | 22.26 | 24.66 | 27.62 | 29.89 | 32.52 | 34.71 |
| Gambia | 13.98 | 15.68 | 17.62 | 20.50 | 22.33 | 23.88 | 26.11 |
| Georgia | 31.60 | 33.98 | 34.82 | 38.62 | 43.76 | 48.88 | 55.43 |
| Germany | 84.02 | 100.21 | 124.33 | 147.54 | 166.30 | 181.54 | 197.73 |
| Ghana | 15.22 | 17.11 | 19.32 | 22.11 | 24.28 | 26.04 | 28.86 |
| Greece | 111.88 | 131.64 | 161.78 | 191.98 | 216.76 | 236.50 | 257.49 |
| Greenland | 71.31 | 76.74 | 99.72 | 120.25 | 133.97 | 144.64 | 154.89 |
| Grenada | 24.56 | 26.11 | 29.46 | 33.53 | 37.70 | 41.60 | 45.91 |
| Guam | 10.53 | 12.02 | 13.41 | 15.85 | 19.62 | 23.90 | 26.65 |
| Guatemala | 9.86 | 10.62 | 11.87 | 13.47 | 15.14 | 16.81 | 18.21 |
| Guinea | 14.93 | 16.91 | 19.19 | 21.60 | 23.67 | 25.46 | 27.87 |
| Guinea-Bissau | 14.46 | 16.50 | 18.70 | 21.24 | 23.27 | 25.02 | 27.52 |
| Guyana | 24.27 | 26.09 | 28.80 | 32.61 | 36.95 | 40.69 | 45.02 |
| Haiti | 33.03 | 35.06 | 39.54 | 46.12 | 51.13 | 55.51 | 61.74 |
| Honduras | 10.18 | 10.86 | 12.25 | 13.80 | 15.64 | 17.44 | 18.83 |
| Hungary | 31.28 | 32.44 | 33.09 | 37.53 | 42.78 | 47.60 | 53.29 |
| Iceland | 123.83 | 145.46 | 178.61 | 212.55 | 239.68 | 263.00 | 286.99 |
| India | 23.32 | 25.22 | 28.75 | 32.94 | 36.68 | 40.11 | 43.07 |
| Indonesia | 4.61 | 5.27 | 5.99 | 7.09 | 8.69 | 10.11 | 11.10 |
| Iran (Islamic Republic of) | 21.94 | 26.94 | 33.32 | 39.05 | 43.09 | 46.46 | 49.79 |
| Iraq | 25.55 | 31.47 | 38.96 | 46.02 | 50.71 | 54.89 | 59.43 |
| Ireland | 113.86 | 133.93 | 165.06 | 195.22 | 220.63 | 241.39 | 263.82 |
| Israel | 103.77 | 123.11 | 151.30 | 179.87 | 202.20 | 221.20 | 242.01 |
| Italy | 107.48 | 124.42 | 151.86 | 182.30 | 204.93 | 223.19 | 244.02 |
| Jamaica | 24.86 | 26.59 | 29.88 | 34.25 | 38.23 | 42.54 | 46.96 |
| Japan | 16.31 | 18.43 | 22.31 | 26.34 | 29.31 | 31.94 | 34.40 |
| Jordan | 20.69 | 25.34 | 31.09 | 36.97 | 40.79 | 43.45 | 46.65 |
| Kazakhstan | 32.39 | 34.75 | 35.41 | 39.74 | 44.58 | 50.07 | 56.32 |
| Kenya | 9.77 | 10.85 | 12.06 | 13.46 | 14.58 | 15.76 | 17.09 |
| Kiribati | 14.30 | 16.28 | 18.72 | 21.65 | 26.62 | 32.13 | 35.45 |
| Kuwait | 17.84 | 21.97 | 27.28 | 32.02 | 35.04 | 37.57 | 40.55 |
| Kyrgyzstan | 35.98 | 38.56 | 39.61 | 44.09 | 49.88 | 55.76 | 63.04 |
| Lao People's Democratic Republic | 10.94 | 12.29 | 13.26 | 15.62 | 18.43 | 21.85 | 24.54 |
| Latvia | 31.76 | 32.47 | 32.32 | 36.94 | 41.97 | 46.68 | 52.42 |
| Lebanon | 20.89 | 25.82 | 31.93 | 37.62 | 41.52 | 44.28 | 47.88 |
| Lesotho | 17.76 | 19.82 | 22.25 | 25.02 | 27.16 | 28.83 | 31.29 |
| Liberia | 13.57 | 15.18 | 17.25 | 19.64 | 21.64 | 23.45 | 25.18 |
| Libya | 19.60 | 24.12 | 30.11 | 35.04 | 38.50 | 41.11 | 44.77 |
| Lithuania | 30.48 | 31.25 | 31.72 | 35.94 | 40.56 | 45.27 | 50.86 |
| Luxembourg | 108.92 | 128.76 | 157.33 | 186.71 | 209.89 | 230.78 | 252.15 |
| Madagascar | 11.45 | 13.15 | 14.86 | 16.98 | 18.28 | 19.69 | 21.35 |
| Malawi | 11.66 | 12.96 | 14.77 | 16.65 | 17.99 | 19.43 | 20.99 |
| Malaysia | 7.43 | 8.31 | 9.16 | 10.52 | 12.58 | 14.85 | 16.30 |
| Maldives | 8.60 | 9.39 | 10.41 | 11.89 | 14.67 | 17.83 | 20.24 |
| Mali | 12.55 | 13.81 | 16.04 | 18.12 | 20.01 | 21.49 | 23.60 |
| Malta | 107.88 | 127.42 | 156.85 | 185.22 | 209.85 | 230.56 | 250.65 |
| Marshall Islands | 13.78 | 15.65 | 17.57 | 20.72 | 25.29 | 30.55 | 34.48 |
| Mauritania | 13.46 | 15.20 | 17.14 | 19.34 | 21.42 | 23.23 | 24.91 |
| Mauritius | 8.70 | 9.51 | 10.61 | 12.25 | 14.42 | 17.23 | 19.25 |
| Mexico | 9.28 | 10.00 | 11.07 | 12.69 | 14.23 | 15.77 | 17.19 |
| Micronesia (Federated States of) | 12.94 | 14.88 | 16.72 | 20.08 | 24.14 | 29.13 | 32.58 |
| Monaco | 102.66 | 121.52 | 148.40 | 176.26 | 197.41 | 217.14 | 236.35 |
| Mongolia | 35.88 | 37.98 | 38.51 | 43.04 | 49.34 | 54.89 | 61.50 |
| Montenegro | 30.17 | 31.45 | 31.88 | 36.54 | 41.28 | 45.91 | 51.63 |
| Morocco | 25.32 | 31.07 | 38.55 | 45.42 | 50.53 | 54.29 | 58.56 |
| Mozambique | 12.60 | 14.10 | 16.02 | 17.95 | 19.50 | 20.97 | 22.49 |
| Myanmar | 10.62 | 11.64 | 13.04 | 15.05 | 17.82 | 21.09 | 23.60 |
| Namibia | 14.75 | 16.39 | 18.76 | 20.91 | 22.89 | 24.53 | 26.59 |
| Nauru | 11.84 | 13.10 | 14.83 | 17.62 | 21.58 | 25.82 | 29.29 |
| Nepal | 15.70 | 17.07 | 19.67 | 22.64 | 25.32 | 27.25 | 29.63 |
| Netherlands | 111.66 | 132.32 | 161.85 | 192.46 | 216.79 | 236.84 | 258.59 |
| New Zealand | 101.91 | 117.18 | 142.17 | 167.73 | 183.51 | 196.28 | 210.03 |
| Nicaragua | 9.65 | 10.43 | 11.51 | 13.14 | 14.92 | 16.52 | 18.01 |
| Niger | 18.37 | 20.85 | 23.34 | 26.71 | 29.17 | 31.71 | 34.30 |
| Nigeria | 15.83 | 17.58 | 19.70 | 22.34 | 24.46 | 26.46 | 28.74 |
| Niue | 11.98 | 13.64 | 15.50 | 18.43 | 22.56 | 27.18 | 30.26 |
| North Macedonia | 31.56 | 32.76 | 33.46 | 38.22 | 43.05 | 48.46 | 54.06 |
| Northern Mariana Islands | 11.79 | 12.83 | 14.52 | 17.34 | 21.54 | 25.48 | 30.24 |
| Norway | 90.44 | 106.30 | 129.06 | 153.29 | 173.24 | 190.42 | 209.31 |
| Oman | 18.43 | 21.48 | 26.75 | 32.08 | 35.67 | 37.84 | 40.86 |
| Pakistan | 19.72 | 21.50 | 24.73 | 28.39 | 31.53 | 34.49 | 37.07 |
| Palau | 10.99 | 12.36 | 13.71 | 16.39 | 20.24 | 24.88 | 28.05 |
| Palestine | 23.21 | 28.73 | 35.65 | 41.99 | 46.14 | 49.47 | 53.17 |
| Panama | 7.94 | 8.61 | 9.66 | 10.95 | 12.30 | 13.37 | 14.64 |
| Papua New Guinea | 15.90 | 18.10 | 20.51 | 24.38 | 29.76 | 35.92 | 40.02 |
| Paraguay | 53.01 | 56.07 | 62.52 | 72.03 | 81.06 | 90.77 | 99.56 |
| Peru | 46.99 | 49.63 | 54.52 | 62.86 | 71.68 | 78.63 | 87.47 |
| Philippines | 9.50 | 10.51 | 11.66 | 13.31 | 16.00 | 19.07 | 21.21 |
| Poland | 38.35 | 39.51 | 39.47 | 44.89 | 50.98 | 56.75 | 64.20 |
| Portugal | 118.20 | 140.14 | 170.84 | 203.26 | 228.61 | 249.80 | 272.48 |
| Puerto Rico | 21.64 | 23.00 | 25.74 | 29.49 | 33.33 | 36.70 | 40.39 |
| Qatar | 17.35 | 20.37 | 25.77 | 31.12 | 34.88 | 36.92 | 39.32 |
| Republic of Korea | 15.85 | 18.00 | 21.63 | 25.52 | 28.84 | 31.26 | 33.43 |
| Republic of Moldova | 33.51 | 34.65 | 34.63 | 39.49 | 44.40 | 49.64 | 55.79 |
| Romania | 29.31 | 30.84 | 31.21 | 35.78 | 40.29 | 45.04 | 51.07 |
| Russian Federation | 32.45 | 33.06 | 32.83 | 37.25 | 41.92 | 46.79 | 52.95 |
| Rwanda | 9.19 | 10.36 | 11.79 | 13.13 | 14.34 | 15.39 | 17.14 |
| Saint Kitts and Nevis | 22.66 | 24.43 | 27.51 | 31.71 | 35.48 | 39.35 | 42.82 |
| Saint Lucia | 24.88 | 26.29 | 29.55 | 33.68 | 37.90 | 41.73 | 45.84 |
| Saint Vincent and the Grenadines | 25.79 | 27.21 | 30.33 | 35.04 | 39.49 | 43.43 | 47.54 |
| Samoa | 14.02 | 15.81 | 17.82 | 21.37 | 25.92 | 31.13 | 35.21 |
| San Marino | 105.58 | 125.45 | 152.32 | 181.14 | 203.03 | 221.02 | 241.88 |
| Sao Tome and Principe | 11.55 | 13.02 | 14.84 | 16.54 | 18.58 | 20.00 | 21.46 |
| Saudi Arabia | 18.22 | 22.00 | 27.60 | 32.59 | 35.62 | 38.04 | 40.90 |
| Senegal | 14.31 | 16.27 | 18.19 | 20.85 | 22.84 | 24.93 | 27.05 |
| Serbia | 31.23 | 32.53 | 33.11 | 37.61 | 42.54 | 47.80 | 53.47 |
| Seychelles | 7.88 | 8.66 | 9.36 | 11.01 | 13.04 | 15.53 | 17.18 |
| Sierra Leone | 14.28 | 16.21 | 18.20 | 20.83 | 23.24 | 24.48 | 26.76 |
| Singapore | 12.61 | 14.34 | 17.38 | 20.17 | 23.00 | 25.16 | 27.12 |
| Slovakia | 31.10 | 32.37 | 32.36 | 36.69 | 42.00 | 46.65 | 52.25 |
| Slovenia | 29.49 | 30.76 | 30.75 | 35.37 | 40.18 | 45.11 | 50.19 |
| Solomon Islands | 15.94 | 18.31 | 20.92 | 24.46 | 30.16 | 36.10 | 40.33 |
| Somalia | 14.69 | 16.54 | 18.90 | 21.45 | 23.04 | 25.39 | 27.25 |
| South Africa | 14.67 | 16.37 | 18.25 | 20.50 | 22.41 | 24.09 | 26.09 |
| South Sudan | 10.70 | 12.15 | 13.88 | 15.61 | 17.02 | 18.31 | 19.85 |
| Spain | 106.88 | 126.81 | 155.70 | 182.22 | 206.31 | 225.51 | 246.50 |
| Sri Lanka | 15.19 | 16.97 | 18.54 | 21.69 | 25.76 | 30.86 | 34.32 |
| Sudan | 23.25 | 28.50 | 35.75 | 41.47 | 46.11 | 49.50 | 53.01 |
| Suriname | 23.73 | 24.99 | 28.09 | 32.20 | 36.22 | 39.89 | 43.97 |
| Sweden | 81.51 | 96.32 | 116.82 | 137.52 | 154.99 | 170.15 | 184.31 |
| Switzerland | 101.92 | 121.00 | 148.36 | 175.50 | 198.83 | 216.76 | 236.46 |
| Syrian Arab Republic | 23.56 | 29.36 | 36.07 | 42.45 | 46.62 | 50.22 | 54.08 |
| Taiwan (Province of China) | 20.96 | 24.96 | 28.00 | 33.35 | 41.68 | 51.31 | 57.52 |
| Tajikistan | 36.79 | 39.41 | 40.57 | 45.29 | 51.34 | 57.32 | 64.67 |
| Thailand | 8.40 | 9.51 | 10.62 | 11.92 | 14.36 | 17.17 | 19.02 |
| Timor-Leste | 10.12 | 11.10 | 11.94 | 14.10 | 16.84 | 19.95 | 22.48 |
| Togo | 13.22 | 14.68 | 16.94 | 19.06 | 20.90 | 22.54 | 24.57 |
| Tokelau | 12.21 | 13.94 | 15.34 | 18.68 | 22.56 | 27.08 | 30.91 |
| Tonga | 13.62 | 15.56 | 17.77 | 20.78 | 25.46 | 30.66 | 34.42 |
| Trinidad and Tobago | 21.50 | 22.87 | 25.76 | 29.54 | 33.46 | 36.69 | 40.39 |
| Tunisia | 21.85 | 27.50 | 33.97 | 39.42 | 44.40 | 47.43 | 50.94 |
| Turkey | 34.45 | 42.92 | 52.55 | 62.19 | 69.69 | 75.18 | 81.73 |
| Turkmenistan | 30.67 | 32.55 | 33.66 | 37.63 | 43.41 | 48.31 | 53.21 |
| Tuvalu | 13.02 | 14.60 | 16.68 | 19.39 | 23.95 | 29.19 | 32.43 |
| Uganda | 10.17 | 11.76 | 13.01 | 14.84 | 16.03 | 17.32 | 18.52 |
| Ukraine | 32.38 | 33.68 | 34.09 | 38.32 | 43.05 | 48.28 | 54.27 |
| United Arab Emirates | 16.95 | 20.50 | 24.60 | 29.49 | 32.26 | 34.65 | 37.47 |
| United Kingdom | 98.08 | 116.52 | 141.80 | 167.35 | 189.21 | 208.34 | 229.87 |
| United Republic of Tanzania | 10.46 | 11.95 | 13.26 | 15.08 | 16.48 | 17.49 | 18.92 |
| United States of America | 59.33 | 65.01 | 83.90 | 101.98 | 113.51 | 122.37 | 130.21 |
| United States Virgin Islands | 21.25 | 22.65 | 25.49 | 29.08 | 32.58 | 36.02 | 39.67 |
| Uruguay | 52.51 | 59.14 | 71.53 | 85.68 | 95.67 | 103.98 | 111.92 |
| Uzbekistan | 34.04 | 36.28 | 36.83 | 41.90 | 47.22 | 52.96 | 59.64 |
| Vanuatu | 15.32 | 17.72 | 20.18 | 23.52 | 28.68 | 34.36 | 39.01 |
| Venezuela (Bolivarian Republic of) | 8.50 | 9.18 | 10.20 | 11.67 | 13.20 | 14.38 | 15.88 |
| Viet Nam | 9.36 | 10.41 | 11.47 | 13.13 | 15.89 | 19.06 | 21.00 |
| Yemen | 25.37 | 31.41 | 38.52 | 45.75 | 50.54 | 54.29 | 58.26 |
| Zambia | 10.19 | 11.48 | 12.90 | 14.51 | 15.79 | 16.84 | 18.02 |
| Zimbabwe | 16.87 | 19.04 | 21.70 | 24.20 | 26.37 | 28.18 | 30.32 |

**Supplementary Table 4. Age distribution of incidence rate for global psoriasis burden of young adults in different countries in 2019.**

| 2019 incidence rate | 15-19 years | 20-24 years | 25-29 years | 30-34 years | 35-39 years | 40-44 years | 45-49 years |
| --- | --- | --- | --- | --- | --- | --- | --- |
| Afghanistan | 50.87 | 64.68 | 78.47 | 85.81 | 88.92 | 94.36 | 101.44 |
| Albania | 56.05 | 46.48 | 54.51 | 67.07 | 72.72 | 79.54 | 88.78 |
| Algeria | 38.97 | 48.96 | 59.73 | 65.70 | 67.77 | 71.91 | 78.35 |
| American Samoa | 21.21 | 22.79 | 27.19 | 33.96 | 42.85 | 48.55 | 49.63 |
| Andorra | 157.91 | 187.10 | 224.48 | 244.41 | 254.83 | 268.71 | 295.03 |
| Angola | 38.64 | 43.50 | 51.29 | 55.90 | 58.86 | 63.15 | 69.39 |
| Antigua and Barbuda | 35.32 | 39.72 | 48.05 | 54.68 | 59.79 | 65.51 | 71.37 |
| Argentina | 72.54 | 88.86 | 110.42 | 119.03 | 124.84 | 131.75 | 143.67 |
| Armenia | 52.37 | 46.51 | 53.00 | 64.18 | 71.54 | 78.16 | 86.48 |
| Australia | 107.57 | 129.95 | 160.81 | 166.47 | 165.44 | 171.36 | 189.05 |
| Austria | 165.96 | 196.72 | 237.06 | 257.54 | 268.32 | 281.82 | 310.22 |
| Azerbaijan | 52.88 | 47.04 | 53.63 | 65.20 | 72.68 | 79.24 | 87.19 |
| Bahamas | 35.14 | 39.57 | 47.76 | 54.50 | 59.52 | 65.17 | 70.82 |
| Bahrain | 36.08 | 45.01 | 55.32 | 60.82 | 61.92 | 65.61 | 71.79 |
| Bangladesh | 27.99 | 32.96 | 39.56 | 44.37 | 47.64 | 51.37 | 55.17 |
| Barbados | 34.43 | 38.78 | 46.79 | 53.39 | 58.56 | 64.04 | 69.53 |
| Belarus | 54.39 | 44.54 | 53.41 | 65.95 | 71.41 | 80.08 | 90.57 |
| Belgium | 168.74 | 199.56 | 239.52 | 260.41 | 271.16 | 285.58 | 313.98 |
| Belize | 39.73 | 44.85 | 54.30 | 61.94 | 66.85 | 72.98 | 79.92 |
| Benin | 23.14 | 26.32 | 30.90 | 34.41 | 36.55 | 39.67 | 43.21 |
| Bermuda | 35.93 | 40.55 | 48.99 | 55.78 | 60.96 | 66.56 | 72.36 |
| Bhutan | 29.22 | 34.41 | 41.51 | 46.33 | 49.77 | 53.69 | 57.57 |
| Bolivia (Plurinational State of) | 62.32 | 70.84 | 88.25 | 100.86 | 107.90 | 116.06 | 128.41 |
| Bosnia and Herzegovina | 55.30 | 45.72 | 53.65 | 65.93 | 72.00 | 79.17 | 87.75 |
| Botswana | 24.03 | 27.49 | 31.96 | 35.06 | 37.65 | 40.28 | 43.94 |
| Brazil | 59.67 | 67.53 | 82.68 | 94.51 | 102.35 | 110.94 | 121.52 |
| Brunei Darussalam | 23.56 | 29.30 | 36.48 | 40.68 | 44.20 | 46.92 | 50.42 |
| Bulgaria | 53.39 | 44.52 | 51.87 | 63.87 | 69.92 | 76.71 | 85.01 |
| Burkina Faso | 25.37 | 28.88 | 33.90 | 37.71 | 39.84 | 43.20 | 47.17 |
| Burundi | 19.60 | 22.63 | 26.13 | 28.49 | 30.25 | 32.48 | 35.68 |
| Cabo Verde | 21.37 | 24.18 | 28.42 | 31.70 | 33.67 | 36.59 | 39.89 |
| Cambodia | 18.11 | 19.09 | 22.51 | 27.58 | 34.11 | 38.21 | 39.43 |
| Cameroon | 20.75 | 23.63 | 27.72 | 31.00 | 32.86 | 35.82 | 39.03 |
| Canada | 46.82 | 76.17 | 103.74 | 101.16 | 101.09 | 103.11 | 116.22 |
| Central African Republic | 43.32 | 48.99 | 58.08 | 63.39 | 65.78 | 70.27 | 77.86 |
| Chad | 26.09 | 29.63 | 34.77 | 38.68 | 40.85 | 44.40 | 48.13 |
| Chile | 70.43 | 86.28 | 107.57 | 116.33 | 121.65 | 128.82 | 140.32 |
| China | 36.69 | 38.64 | 44.74 | 58.37 | 77.39 | 86.41 | 83.46 |
| Colombia | 13.44 | 15.20 | 18.23 | 20.84 | 23.19 | 25.58 | 27.76 |
| Comoros | 18.05 | 20.74 | 24.02 | 26.28 | 27.81 | 30.12 | 32.98 |
| Congo | 32.75 | 36.64 | 42.93 | 46.88 | 50.26 | 54.04 | 58.83 |
| Cook Islands | 20.82 | 22.44 | 26.54 | 33.05 | 41.71 | 46.97 | 48.12 |
| Costa Rica | 13.34 | 15.10 | 18.05 | 20.69 | 23.01 | 25.37 | 27.56 |
| Croatia | 52.83 | 44.05 | 51.50 | 63.10 | 69.10 | 75.95 | 84.09 |
| Cuba | 38.47 | 43.30 | 52.56 | 59.67 | 64.69 | 70.86 | 77.31 |
| Cyprus | 157.99 | 186.82 | 224.92 | 244.40 | 253.95 | 267.70 | 293.80 |
| Czechia | 52.52 | 43.89 | 51.14 | 62.96 | 68.75 | 75.53 | 83.94 |
| Côte d'Ivoire | 22.15 | 25.15 | 29.57 | 32.99 | 34.93 | 37.89 | 41.29 |
| Democratic People's Republic of Korea | 44.37 | 46.77 | 54.86 | 71.07 | 94.30 | 105.49 | 101.72 |
| Democratic Republic of the Congo | 39.20 | 44.33 | 52.42 | 57.04 | 59.91 | 64.10 | 70.61 |
| Denmark | 168.14 | 198.79 | 238.68 | 259.96 | 270.18 | 284.39 | 313.41 |
| Djibouti | 17.73 | 20.45 | 23.66 | 25.97 | 27.43 | 29.67 | 32.63 |
| Dominica | 35.52 | 40.07 | 48.25 | 55.21 | 60.18 | 65.60 | 71.59 |
| Dominican Republic | 40.17 | 45.43 | 54.89 | 62.34 | 67.42 | 73.48 | 80.63 |
| Ecuador | 52.10 | 59.29 | 73.24 | 83.70 | 90.67 | 98.29 | 106.81 |
| Egypt | 24.73 | 31.07 | 38.21 | 41.96 | 42.85 | 45.97 | 50.62 |
| El Salvador | 14.96 | 16.91 | 20.42 | 23.28 | 25.59 | 28.28 | 30.76 |
| Equatorial Guinea | 30.54 | 34.33 | 39.95 | 43.68 | 47.33 | 50.80 | 55.19 |
| Eritrea | 19.19 | 22.13 | 25.46 | 27.92 | 29.75 | 31.88 | 34.94 |
| Estonia | 52.38 | 43.29 | 51.84 | 63.91 | 69.21 | 77.66 | 87.71 |
| Eswatini | 25.95 | 29.72 | 34.60 | 37.83 | 40.30 | 43.32 | 47.27 |
| Ethiopia | 19.00 | 21.73 | 25.12 | 27.46 | 29.28 | 31.57 | 34.36 |
| Fiji | 22.75 | 24.44 | 29.26 | 36.53 | 45.87 | 51.97 | 53.44 |
| Finland | 178.85 | 212.71 | 254.89 | 276.78 | 287.88 | 302.11 | 333.45 |
| France | 193.39 | 228.08 | 274.43 | 297.63 | 308.43 | 324.26 | 359.90 |
| Gabon | 30.59 | 34.42 | 40.11 | 43.78 | 47.28 | 50.66 | 54.98 |
| Gambia | 23.23 | 26.46 | 31.10 | 34.60 | 36.69 | 39.75 | 43.31 |
| Georgia | 52.70 | 47.08 | 53.76 | 64.87 | 72.54 | 79.01 | 87.23 |
| Germany | 139.82 | 166.03 | 200.11 | 218.51 | 228.44 | 241.38 | 263.63 |
| Ghana | 24.50 | 27.85 | 32.85 | 36.52 | 38.72 | 41.92 | 45.75 |
| Greece | 166.28 | 196.73 | 235.79 | 256.38 | 267.65 | 280.71 | 308.83 |
| Greenland | 52.90 | 88.70 | 124.30 | 119.68 | 117.08 | 119.47 | 134.49 |
| Grenada | 36.69 | 41.42 | 50.07 | 57.12 | 62.09 | 67.75 | 73.90 |
| Guam | 19.15 | 20.51 | 24.52 | 30.72 | 39.39 | 44.72 | 45.65 |
| Guatemala | 15.84 | 18.03 | 21.69 | 24.70 | 27.05 | 29.86 | 32.51 |
| Guinea | 24.29 | 27.56 | 32.39 | 36.21 | 38.23 | 41.47 | 45.12 |
| Guinea-Bissau | 23.90 | 27.28 | 32.06 | 35.70 | 37.73 | 41.04 | 44.61 |
| Guyana | 36.47 | 41.31 | 49.92 | 56.70 | 61.88 | 67.65 | 73.66 |
| Haiti | 45.69 | 52.05 | 63.46 | 71.94 | 76.68 | 83.39 | 92.29 |
| Honduras | 16.28 | 18.52 | 22.32 | 25.39 | 27.67 | 30.46 | 33.44 |
| Hungary | 53.45 | 44.39 | 51.82 | 63.80 | 69.56 | 76.44 | 85.00 |
| Iceland | 177.55 | 209.67 | 252.41 | 273.26 | 284.62 | 299.17 | 328.61 |
| India | 32.52 | 38.22 | 46.13 | 51.14 | 55.00 | 59.10 | 62.77 |
| Indonesia | 8.40 | 9.07 | 10.73 | 13.52 | 16.96 | 18.95 | 19.71 |
| Iran (Islamic Republic of) | 39.08 | 49.20 | 59.91 | 65.73 | 68.27 | 72.46 | 78.54 |
| Iraq | 43.65 | 55.02 | 67.07 | 73.74 | 76.50 | 81.03 | 87.68 |
| Ireland | 168.76 | 199.52 | 239.60 | 259.63 | 270.13 | 284.33 | 314.14 |
| Israel | 158.72 | 188.14 | 225.47 | 245.42 | 256.18 | 270.45 | 296.52 |
| Italy | 130.64 | 150.51 | 189.84 | 204.01 | 204.27 | 213.71 | 242.19 |
| Jamaica | 37.10 | 41.95 | 50.73 | 57.82 | 62.73 | 68.65 | 75.03 |
| Japan | 27.63 | 33.98 | 42.51 | 47.17 | 50.31 | 53.85 | 58.05 |
| Jordan | 37.13 | 46.52 | 56.96 | 62.78 | 64.70 | 68.76 | 74.80 |
| Kazakhstan | 54.18 | 47.81 | 54.72 | 66.18 | 73.74 | 80.38 | 88.92 |
| Kenya | 16.71 | 19.07 | 21.86 | 23.67 | 25.11 | 27.28 | 29.92 |
| Kiribati | 24.88 | 26.66 | 32.00 | 39.90 | 49.36 | 55.35 | 56.92 |
| Kuwait | 33.49 | 42.00 | 51.33 | 56.61 | 58.12 | 61.71 | 67.70 |
| Kyrgyzstan | 58.28 | 51.81 | 59.49 | 71.44 | 79.11 | 86.41 | 95.41 |
| Lao People's Democratic Republic | 18.61 | 19.54 | 22.93 | 28.22 | 34.97 | 39.20 | 40.45 |
| Latvia | 52.39 | 43.29 | 51.73 | 63.70 | 69.09 | 77.32 | 87.30 |
| Lebanon | 37.67 | 47.40 | 57.97 | 63.74 | 66.11 | 70.26 | 76.54 |
| Lesotho | 28.29 | 32.48 | 37.86 | 41.05 | 43.44 | 46.95 | 51.50 |
| Liberia | 22.82 | 26.06 | 30.58 | 34.07 | 36.10 | 39.06 | 42.57 |
| Libya | 36.10 | 45.26 | 55.35 | 60.98 | 62.82 | 66.77 | 72.93 |
| Lithuania | 51.29 | 42.55 | 50.84 | 62.31 | 67.77 | 75.89 | 85.64 |
| Luxembourg | 163.70 | 193.53 | 231.66 | 251.42 | 263.41 | 277.22 | 305.02 |
| Madagascar | 19.50 | 22.48 | 25.94 | 28.38 | 30.18 | 32.47 | 35.56 |
| Malawi | 19.44 | 22.40 | 25.88 | 28.33 | 30.08 | 32.32 | 35.34 |
| Malaysia | 13.33 | 14.19 | 16.52 | 20.37 | 25.37 | 28.49 | 29.48 |
| Maldives | 15.40 | 16.01 | 18.71 | 23.39 | 29.56 | 33.65 | 35.11 |
| Mali | 21.44 | 24.47 | 28.73 | 32.07 | 34.11 | 36.95 | 40.17 |
| Malta | 162.96 | 192.87 | 231.07 | 252.14 | 262.90 | 276.75 | 304.07 |
| Marshall Islands | 23.93 | 25.67 | 30.70 | 38.41 | 48.08 | 54.12 | 55.74 |
| Mauritania | 22.46 | 25.53 | 29.92 | 33.48 | 35.38 | 38.39 | 41.89 |
| Mauritius | 15.34 | 16.29 | 19.07 | 23.32 | 28.96 | 32.44 | 33.61 |
| Mexico | 14.85 | 16.76 | 20.20 | 22.95 | 25.31 | 27.93 | 30.26 |
| Micronesia (Federated States of) | 22.85 | 24.70 | 29.57 | 36.94 | 46.37 | 52.33 | 53.76 |
| Monaco | 157.78 | 186.55 | 223.99 | 243.19 | 252.63 | 267.23 | 294.45 |
| Mongolia | 57.62 | 51.26 | 58.62 | 70.47 | 78.25 | 85.59 | 94.44 |
| Montenegro | 52.24 | 43.70 | 50.89 | 62.43 | 68.09 | 74.81 | 83.32 |
| Morocco | 43.39 | 54.63 | 66.37 | 72.91 | 75.21 | 80.12 | 86.81 |
| Mozambique | 20.62 | 23.75 | 27.38 | 29.95 | 31.94 | 34.29 | 37.41 |
| Myanmar | 18.08 | 19.14 | 22.52 | 27.43 | 33.89 | 37.92 | 39.06 |
| Namibia | 24.49 | 28.04 | 32.51 | 35.68 | 38.28 | 41.10 | 44.84 |
| Nauru | 20.87 | 22.44 | 26.79 | 33.47 | 42.24 | 47.88 | 48.91 |
| Nepal | 25.08 | 29.38 | 35.29 | 39.77 | 42.72 | 46.15 | 49.58 |
| Netherlands | 166.43 | 196.89 | 235.37 | 256.71 | 266.77 | 281.24 | 310.13 |
| New Zealand | 121.67 | 145.52 | 178.65 | 185.89 | 183.09 | 191.41 | 213.65 |
| Nicaragua | 15.59 | 17.72 | 21.30 | 24.26 | 26.58 | 29.30 | 31.91 |
| Niger | 27.71 | 31.59 | 37.01 | 41.18 | 43.49 | 47.11 | 51.38 |
| Nigeria | 25.05 | 28.35 | 33.15 | 36.69 | 38.94 | 42.12 | 45.66 |
| Niue | 21.22 | 23.10 | 27.52 | 34.46 | 43.51 | 49.34 | 50.14 |
| North Macedonia | 53.69 | 44.76 | 52.47 | 64.45 | 70.36 | 77.24 | 85.81 |
| Northern Mariana Islands | 20.56 | 22.15 | 26.37 | 33.04 | 42.38 | 46.17 | 51.28 |
| Norway | 127.35 | 148.60 | 179.22 | 194.08 | 203.30 | 215.29 | 237.06 |
| Oman | 34.06 | 41.45 | 51.85 | 56.90 | 58.14 | 61.91 | 67.68 |
| Pakistan | 29.44 | 34.27 | 41.48 | 46.32 | 49.90 | 53.76 | 57.15 |
| Palau | 19.87 | 21.11 | 25.21 | 31.94 | 41.17 | 46.97 | 47.85 |
| Palestine | 40.97 | 51.51 | 63.01 | 69.01 | 71.20 | 75.63 | 82.25 |
| Panama | 13.19 | 14.87 | 17.79 | 20.42 | 22.75 | 25.12 | 27.14 |
| Papua New Guinea | 26.77 | 29.02 | 34.93 | 43.60 | 53.71 | 60.22 | 62.85 |
| Paraguay | 61.50 | 69.53 | 85.86 | 97.89 | 105.06 | 114.32 | 126.05 |
| Peru | 55.54 | 63.19 | 78.16 | 89.37 | 96.36 | 104.46 | 113.92 |
| Philippines | 16.48 | 17.45 | 20.46 | 25.01 | 30.99 | 34.94 | 36.16 |
| Poland | 61.11 | 48.77 | 58.17 | 71.58 | 78.23 | 86.66 | 96.53 |
| Portugal | 172.51 | 203.89 | 244.07 | 265.42 | 276.43 | 290.15 | 320.33 |
| Puerto Rico | 33.29 | 37.56 | 45.35 | 51.73 | 56.78 | 62.07 | 67.56 |
| Qatar | 32.43 | 39.53 | 50.64 | 55.86 | 57.03 | 60.46 | 66.09 |
| Republic of Korea | 27.25 | 33.68 | 42.20 | 46.82 | 49.96 | 53.25 | 57.56 |
| Republic of Moldova | 54.66 | 44.87 | 53.63 | 66.34 | 71.95 | 80.63 | 90.70 |
| Romania | 51.12 | 43.04 | 50.28 | 61.47 | 67.21 | 73.98 | 81.80 |
| Russian Federation | 52.79 | 43.05 | 51.92 | 63.71 | 68.98 | 77.67 | 87.73 |
| Rwanda | 16.21 | 18.72 | 21.60 | 23.77 | 25.13 | 27.29 | 30.18 |
| Saint Kitts and Nevis | 35.02 | 39.53 | 47.67 | 54.40 | 59.35 | 64.80 | 70.63 |
| Saint Lucia | 36.83 | 41.66 | 50.25 | 57.05 | 62.39 | 68.06 | 74.26 |
| Saint Vincent and the Grenadines | 37.87 | 42.82 | 51.85 | 58.80 | 64.00 | 69.89 | 76.24 |
| Samoa | 24.04 | 25.88 | 31.02 | 38.78 | 48.37 | 54.70 | 56.67 |
| San Marino | 161.18 | 188.96 | 225.75 | 247.09 | 257.33 | 270.22 | 299.44 |
| Sao Tome and Principe | 20.10 | 22.80 | 26.85 | 29.90 | 31.84 | 34.59 | 37.73 |
| Saudi Arabia | 33.60 | 42.35 | 52.22 | 57.49 | 58.98 | 62.59 | 68.41 |
| Senegal | 23.53 | 26.79 | 31.49 | 35.22 | 37.12 | 40.34 | 44.01 |
| Serbia | 53.66 | 44.67 | 52.25 | 63.95 | 70.02 | 76.94 | 85.55 |
| Seychelles | 13.98 | 14.74 | 17.15 | 21.23 | 26.55 | 29.95 | 31.08 |
| Sierra Leone | 23.67 | 26.88 | 31.61 | 35.26 | 37.29 | 40.41 | 44.01 |
| Singapore | 22.21 | 27.77 | 34.27 | 38.38 | 41.97 | 44.34 | 48.03 |
| Slovakia | 52.90 | 43.98 | 51.57 | 63.27 | 69.10 | 75.85 | 84.12 |
| Slovenia | 51.11 | 42.89 | 50.01 | 61.52 | 67.32 | 74.03 | 81.89 |
| Solomon Islands | 27.00 | 29.12 | 35.07 | 43.69 | 53.70 | 60.47 | 62.72 |
| Somalia | 23.00 | 26.60 | 30.52 | 33.38 | 35.81 | 37.93 | 41.34 |
| South Africa | 24.42 | 27.79 | 32.23 | 35.26 | 37.86 | 40.71 | 44.35 |
| South Sudan | 18.67 | 21.55 | 24.91 | 27.32 | 28.96 | 31.16 | 34.15 |
| Spain | 162.10 | 190.45 | 227.35 | 247.65 | 259.21 | 273.00 | 300.45 |
| Sri Lanka | 23.86 | 24.73 | 29.39 | 36.33 | 44.76 | 50.00 | 52.09 |
| Sudan | 40.77 | 51.16 | 62.37 | 68.58 | 70.81 | 75.52 | 81.66 |
| Suriname | 35.65 | 40.21 | 48.58 | 55.32 | 60.40 | 66.33 | 72.10 |
| Sweden | 136.21 | 160.00 | 191.45 | 209.82 | 221.14 | 234.08 | 254.02 |
| Switzerland | 156.82 | 185.51 | 222.73 | 243.12 | 253.80 | 267.06 | 293.27 |
| Syrian Arab Republic | 41.33 | 52.21 | 62.93 | 69.38 | 71.69 | 76.13 | 82.69 |
| Taiwan (Province of China) | 34.77 | 37.43 | 43.38 | 55.80 | 72.02 | 79.37 | 76.68 |
| Tajikistan | 59.16 | 52.78 | 60.60 | 72.90 | 80.43 | 87.84 | 97.12 |
| Thailand | 15.12 | 16.09 | 18.83 | 23.01 | 28.60 | 32.09 | 33.09 |
| Timor-Leste | 17.24 | 18.36 | 21.60 | 26.37 | 32.65 | 36.73 | 38.06 |
| Togo | 22.18 | 25.25 | 29.71 | 33.17 | 35.14 | 38.12 | 41.55 |
| Tokelau | 21.42 | 23.34 | 27.55 | 34.61 | 44.58 | 48.42 | 51.79 |
| Tonga | 23.85 | 25.77 | 30.69 | 38.25 | 47.58 | 53.63 | 55.38 |
| Trinidad and Tobago | 33.42 | 37.62 | 45.35 | 51.69 | 56.81 | 62.31 | 67.37 |
| Tunisia | 39.31 | 49.67 | 60.47 | 66.29 | 68.77 | 72.72 | 79.21 |
| Turkey | 54.14 | 68.14 | 82.21 | 90.06 | 94.04 | 98.89 | 107.13 |
| Turkmenistan | 51.64 | 45.91 | 52.68 | 64.03 | 71.53 | 77.77 | 85.16 |
| Tuvalu | 22.46 | 24.26 | 29.07 | 36.40 | 45.95 | 52.12 | 53.29 |
| Uganda | 17.82 | 20.51 | 23.72 | 25.93 | 27.42 | 29.75 | 32.64 |
| Ukraine | 53.38 | 44.91 | 52.86 | 64.91 | 71.22 | 79.52 | 90.02 |
| United Arab Emirates | 31.86 | 39.71 | 48.60 | 53.16 | 54.51 | 57.48 | 62.91 |
| United Kingdom | 153.30 | 185.49 | 221.64 | 240.11 | 251.01 | 264.91 | 292.21 |
| United Republic of Tanzania | 17.96 | 20.68 | 23.90 | 26.22 | 27.78 | 29.97 | 32.89 |
| United States of America | 48.82 | 78.23 | 108.81 | 107.23 | 106.26 | 107.45 | 119.70 |
| United States Virgin Islands | 32.82 | 36.98 | 44.60 | 51.03 | 55.95 | 61.23 | 66.51 |
| Uruguay | 71.72 | 87.74 | 109.20 | 118.14 | 123.64 | 130.83 | 142.15 |
| Uzbekistan | 55.94 | 49.69 | 56.81 | 68.45 | 76.29 | 83.28 | 91.52 |
| Vanuatu | 26.30 | 28.41 | 34.08 | 42.39 | 52.18 | 58.90 | 61.17 |
| Venezuela (Bolivarian Republic of) | 13.96 | 15.84 | 18.95 | 21.73 | 24.05 | 26.53 | 28.80 |
| Viet Nam | 16.24 | 17.26 | 20.26 | 24.76 | 30.76 | 34.53 | 35.71 |
| Yemen | 43.59 | 54.91 | 66.77 | 73.38 | 76.03 | 80.23 | 87.30 |
| Zambia | 17.53 | 20.24 | 23.38 | 25.56 | 27.07 | 29.28 | 32.19 |
| Zimbabwe | 27.31 | 31.27 | 36.43 | 39.69 | 42.13 | 45.24 | 49.84 |

**Supplementary Table 5. The age-standerized rate of incidence and DALY for global psoriasis burden of young adults in 1990 and 2019, by countries and regions and gender.**

| Country | Region | Sex | ASIR 1990 | DALYR 1990 | ASIR 2019 | DALYR 2019 |
| --- | --- | --- | --- | --- | --- | --- |
| Afghanistan | North Africa and Middle East | both | 95.53 | 64.19 | 78.69 | 52.47 |
| Albania | Central Europe | both | 81.07 | 54.21 | 64.75 | 41.33 |
| Algeria | North Africa and Middle East | both | 75.19 | 46.78 | 60.10 | 36.19 |
| American Samoa | Oceania | both | 38.33 | 21.19 | 33.78 | 18.60 |
| Andorra | Western Europe | both | 236.50 | 180.56 | 227.81 | 167.14 |
| Angola | Central sub-Saharan Africa | both | 68.36 | 50.70 | 53.13 | 37.48 |
| Antigua and Barbuda | Caribbean | both | 57.67 | 35.17 | 51.93 | 31.53 |
| Argentina | Southern Latin America | both | 117.37 | 92.55 | 110.23 | 81.48 |
| Armenia | Central Asia | both | 78.83 | 51.48 | 62.89 | 39.43 |
| Australia | Australasia | both | 165.02 | 155.08 | 152.90 | 135.27 |
| Austria | Western Europe | both | 254.45 | 202.30 | 239.72 | 181.33 |
| Azerbaijan | Central Asia | both | 75.74 | 48.79 | 63.68 | 40.05 |
| Bahrain | North Africa and Middle East | both | 67.02 | 39.82 | 55.28 | 32.25 |
| Bangladesh | South Asia | both | 49.88 | 31.14 | 41.59 | 25.93 |
| Barbados | Caribbean | both | 54.50 | 32.78 | 50.69 | 30.63 |
| Belarus | Eastern Europe | both | 77.75 | 51.08 | 63.92 | 40.39 |
| Belgium | Western Europe | both | 259.25 | 208.47 | 242.71 | 184.78 |
| Belize | Caribbean | both | 68.42 | 44.59 | 58.37 | 36.84 |
| Benin | Western sub-Saharan Africa | both | 38.84 | 22.96 | 32.62 | 19.55 |
| Bermuda | Caribbean | both | 58.67 | 36.04 | 52.88 | 32.34 |
| Bhutan | South Asia | both | 51.76 | 33.07 | 43.46 | 27.64 |
| Bolivia (Plurinational State of) | Andean Latin America | both | 108.88 | 89.05 | 93.60 | 74.47 |
| Bosnia and Herzegovina | Central Europe | both | 82.44 | 55.53 | 63.95 | 40.72 |
| Botswana | Southern sub-Saharan Africa | both | 40.37 | 24.12 | 33.52 | 19.56 |
| Brazil | Tropical Latin America | both | 94.43 | 70.47 | 88.68 | 67.03 |
| Brunei Darussalam | High-income Asia Pacific | both | 40.43 | 22.25 | 37.69 | 20.51 |
| Bulgaria | Central Europe | both | 76.27 | 48.89 | 61.97 | 38.69 |
| Burkina Faso | Western sub-Saharan Africa | both | 41.64 | 25.38 | 35.68 | 22.32 |
| Burundi | Eastern Sub-Saharan Africa | both | 28.52 | 16.15 | 27.24 | 16.04 |
| Cambodia | Southeast Asia | both | 32.51 | 18.62 | 27.39 | 15.58 |
| Cameroon | Western sub-Saharan Africa | both | 35.26 | 20.01 | 29.35 | 16.66 |
| Canada | High-income North America | both | 97.47 | 102.24 | 90.38 | 90.11 |
| Cabo Verde | Western sub-Saharan Africa | both | 37.69 | 22.16 | 30.05 | 17.37 |
| Central African Republic | Central sub-Saharan Africa | both | 68.04 | 49.95 | 59.71 | 43.96 |
| Chad | Western sub-Saharan Africa | both | 42.87 | 26.76 | 36.59 | 23.20 |
| Chile | Southern Latin America | both | 117.86 | 92.90 | 107.46 | 78.32 |
| China | East Asia | both | 74.88 | 51.09 | 58.37 | 38.29 |
| Colombia | Central Latin America | both | 23.03 | 12.69 | 19.98 | 11.06 |
| Comoros | Eastern Sub-Saharan Africa | both | 27.95 | 15.86 | 25.10 | 14.49 |
| Congo | Central sub-Saharan Africa | both | 56.68 | 38.51 | 44.96 | 29.41 |
| Costa Rica | Central Latin America | both | 22.65 | 12.49 | 19.82 | 10.92 |
| Côte d'Ivoire | Western sub-Saharan Africa | both | 37.02 | 21.33 | 31.20 | 18.28 |
| Croatia | Central Europe | both | 74.81 | 47.37 | 61.32 | 38.03 |
| Cuba | Caribbean | both | 60.57 | 37.41 | 56.46 | 35.31 |
| Cyprus | Western Europe | both | 253.86 | 202.33 | 227.46 | 166.79 |
| Czechia | Central Europe | both | 75.78 | 48.52 | 61.05 | 37.76 |
| Democratic Republic of the Congo | Central sub-Saharan Africa | both | 63.38 | 44.96 | 54.09 | 38.38 |
| Denmark | Western Europe | both | 255.16 | 203.11 | 241.95 | 183.63 |
| Djibouti | Eastern Sub-Saharan Africa | both | 28.05 | 16.03 | 24.76 | 14.26 |
| Dominica | Caribbean | both | 58.93 | 36.30 | 52.22 | 31.68 |
| Dominican Republic | Caribbean | both | 67.66 | 43.86 | 58.90 | 37.43 |
| Ecuador | Andean Latin America | both | 90.77 | 68.82 | 78.25 | 56.96 |
| Egypt | North Africa and Middle East | both | 47.77 | 25.47 | 38.35 | 20.09 |
| El Salvador | Central Latin America | both | 26.07 | 14.52 | 22.20 | 12.41 |
| Equatorial Guinea | Central sub-Saharan Africa | both | 68.27 | 50.14 | 42.08 | 26.93 |
| Eritrea | Eastern Sub-Saharan Africa | both | 30.62 | 17.74 | 26.68 | 15.71 |
| Estonia | Eastern Europe | both | 75.19 | 48.35 | 61.92 | 38.32 |
| Ethiopia | Eastern Sub-Saharan Africa | both | 29.93 | 17.55 | 26.30 | 15.59 |
| Micronesia (Federated States of) | Oceania | both | 42.26 | 23.98 | 36.57 | 20.61 |
| Fiji | Oceania | both | 41.85 | 23.71 | 36.26 | 20.48 |
| Finland | Western Europe | both | 274.04 | 226.63 | 257.78 | 202.81 |
| France | Western Europe | both | 296.45 | 257.00 | 277.24 | 228.64 |
| Gabon | Central sub-Saharan Africa | both | 55.12 | 37.09 | 42.09 | 26.86 |
| Georgia | Central Asia | both | 73.13 | 46.47 | 63.59 | 39.97 |
| Germany | Western Europe | both | 209.72 | 148.27 | 203.36 | 138.17 |
| Ghana | Western sub-Saharan Africa | both | 41.55 | 25.54 | 34.56 | 21.27 |
| Greece | Western Europe | both | 256.74 | 205.50 | 239.02 | 180.52 |
| Greenland | High-income North America | both | 116.53 | 126.55 | 105.54 | 110.73 |
| Grenada | Caribbean | both | 63.37 | 40.06 | 53.98 | 33.17 |
| Guam | Oceania | both | 35.10 | 19.08 | 30.79 | 16.69 |
| Guatemala | Central Latin America | both | 28.42 | 16.12 | 23.52 | 13.34 |
| Guinea | Western sub-Saharan Africa | both | 40.12 | 24.21 | 34.17 | 20.83 |
| Guinea-Bissau | Western sub-Saharan Africa | both | 40.20 | 24.20 | 33.76 | 20.41 |
| Guyana | Caribbean | both | 60.40 | 36.98 | 53.77 | 32.57 |
| Haiti | Caribbean | both | 74.70 | 49.80 | 67.41 | 44.74 |
| Honduras | Central Latin America | both | 27.96 | 15.93 | 24.13 | 13.75 |
| Hungary | Central Europe | both | 76.57 | 49.19 | 61.85 | 38.71 |
| Iceland | Western Europe | both | 272.92 | 226.57 | 254.83 | 200.04 |
| India | South Asia | both | 51.52 | 32.46 | 48.00 | 31.98 |
| Indonesia | Southeast Asia | both | 14.98 | 8.07 | 13.36 | 7.26 |
| Iran (Islamic Republic of) | North Africa and Middle East | both | 79.30 | 49.76 | 60.35 | 36.04 |
| Iraq | North Africa and Middle East | both | 88.02 | 58.26 | 67.53 | 42.43 |
| Ireland | Western Europe | both | 265.09 | 215.79 | 242.33 | 184.04 |
| Israel | Western Europe | both | 242.23 | 188.04 | 228.98 | 168.79 |
| Italy | Western Europe | both | 199.97 | 183.99 | 186.52 | 170.91 |
| Jamaica | Caribbean | both | 62.02 | 39.06 | 54.66 | 33.77 |
| Japan | High-income Asia Pacific | both | 43.66 | 24.89 | 43.55 | 24.78 |
| Jordan | North Africa and Middle East | both | 71.73 | 43.71 | 57.34 | 33.89 |
| Kazakhstan | Central Asia | both | 79.04 | 51.49 | 64.81 | 40.84 |
| Kenya | Eastern Sub-Saharan Africa | both | 25.32 | 14.30 | 22.83 | 13.05 |
| Kiribati | Oceania | both | 41.85 | 23.60 | 39.17 | 22.63 |
| Kuwait | North Africa and Middle East | both | 61.79 | 35.67 | 51.67 | 29.37 |
| Kyrgyzstan | Central Asia | both | 81.19 | 53.76 | 69.85 | 45.51 |
| Lao People's Democratic Republic | Southeast Asia | both | 33.51 | 19.41 | 28.06 | 16.09 |
| Latvia | Eastern Europe | both | 74.32 | 47.34 | 61.77 | 38.27 |
| Lebanon | North Africa and Middle East | both | 75.26 | 46.51 | 58.44 | 34.57 |
| Lesotho | Southern sub-Saharan Africa | both | 46.21 | 29.09 | 39.28 | 24.01 |
| Liberia | Western sub-Saharan Africa | both | 37.63 | 21.63 | 32.22 | 18.91 |
| Libya | North Africa and Middle East | both | 71.52 | 43.65 | 55.75 | 32.26 |
| Lithuania | Eastern Europe | both | 74.12 | 47.19 | 60.58 | 37.08 |
| Luxembourg | Western Europe | both | 245.48 | 190.91 | 235.28 | 175.86 |
| North Macedonia | Central Europe | both | 76.89 | 49.77 | 62.44 | 39.20 |
| Madagascar | Eastern Sub-Saharan Africa | both | 29.69 | 17.18 | 27.13 | 16.12 |
| Malawi | Eastern Sub-Saharan Africa | both | 30.41 | 17.59 | 27.03 | 15.95 |
| Malaysia | Southeast Asia | both | 24.29 | 13.05 | 20.32 | 10.90 |
| Maldives | Southeast Asia | both | 29.58 | 16.43 | 23.58 | 12.76 |
| Mali | Western sub-Saharan Africa | both | 36.95 | 21.41 | 30.35 | 17.47 |
| Malta | Western Europe | both | 256.13 | 204.94 | 234.83 | 174.98 |
| Marshall Islands | Oceania | both | 44.70 | 25.88 | 37.97 | 21.64 |
| Mauritania | Western sub-Saharan Africa | both | 37.92 | 22.44 | 31.63 | 18.75 |
| Mauritius | Southeast Asia | both | 27.13 | 14.77 | 23.26 | 12.66 |
| Mexico | Central Latin America | both | 25.07 | 14.26 | 21.94 | 12.53 |
| Republic of Moldova | Eastern Europe | both | 75.75 | 49.16 | 64.26 | 40.71 |
| Mongolia | Central Asia | both | 85.53 | 57.93 | 69.04 | 44.72 |
| Montenegro | Central Europe | both | 73.01 | 45.70 | 60.60 | 37.43 |
| Morocco | North Africa and Middle East | both | 83.41 | 54.06 | 66.81 | 41.97 |
| Mozambique | Eastern Sub-Saharan Africa | both | 33.14 | 19.75 | 28.64 | 17.23 |
| Myanmar | Southeast Asia | both | 32.95 | 18.96 | 27.27 | 15.54 |
| Namibia | Southern sub-Saharan Africa | both | 39.91 | 23.72 | 34.15 | 20.18 |
| Nepal | South Asia | both | 48.32 | 29.45 | 37.26 | 21.85 |
| Netherlands | Western Europe | both | 252.67 | 201.28 | 239.15 | 180.84 |
| New Zealand | Australasia | both | 182.52 | 173.18 | 170.99 | 155.14 |
| Nicaragua | Central Latin America | both | 27.17 | 15.29 | 23.11 | 13.08 |
| Niger | Western sub-Saharan Africa | both | 43.91 | 27.88 | 38.94 | 25.66 |
| Nigeria | Western sub-Saharan Africa | both | 46.54 | 30.56 | 34.85 | 21.60 |
| Democratic People's Republic of Korea | East Asia | both | 85.75 | 63.45 | 71.10 | 51.33 |
| Northern Mariana Islands | Oceania | both | 35.71 | 19.40 | 33.13 | 18.28 |
| Norway | Western Europe | both | 192.48 | 157.45 | 182.05 | 145.13 |
| Oman | North Africa and Middle East | both | 73.88 | 45.76 | 51.83 | 29.47 |
| Pakistan | South Asia | both | 51.62 | 32.75 | 43.45 | 27.43 |
| Palestine | North Africa and Middle East | both | 85.22 | 55.40 | 63.20 | 38.50 |
| Panama | Central Latin America | both | 22.18 | 12.18 | 19.57 | 10.77 |
| Papua New Guinea | Oceania | both | 47.89 | 28.13 | 42.71 | 25.27 |
| Paraguay | Tropical Latin America | both | 96.91 | 74.49 | 91.58 | 71.46 |
| Peru | Andean Latin America | both | 95.70 | 74.05 | 83.37 | 62.71 |
| Philippines | Southeast Asia | both | 27.54 | 15.17 | 24.97 | 13.93 |
| Poland | Central Europe | both | 79.25 | 51.83 | 69.69 | 46.55 |
| Portugal | Western Europe | both | 270.41 | 221.83 | 247.46 | 190.92 |
| Puerto Rico | Caribbean | both | 54.80 | 32.77 | 49.14 | 29.20 |
| Qatar | North Africa and Middle East | both | 64.53 | 37.77 | 50.40 | 28.43 |
| Romania | Central Europe | both | 74.35 | 47.03 | 59.68 | 36.67 |
| Russian Federation | Eastern Europe | both | 70.50 | 43.66 | 61.91 | 38.67 |
| Rwanda | Eastern Sub-Saharan Africa | both | 25.52 | 14.08 | 22.70 | 12.72 |
| Saint Lucia | Caribbean | both | 61.69 | 38.45 | 54.18 | 33.32 |
| Saint Vincent and the Grenadines | Caribbean | both | 63.39 | 40.08 | 55.71 | 34.56 |
| Samoa | Oceania | both | 40.75 | 23.13 | 38.34 | 22.08 |
| Sao Tome and Principe | Central sub-Saharan Africa | both | 34.73 | 19.76 | 28.38 | 16.14 |
| Saudi Arabia | North Africa and Middle East | both | 78.62 | 50.16 | 52.32 | 29.75 |
| Senegal | Western sub-Saharan Africa | both | 39.13 | 23.28 | 33.22 | 20.08 |
| Serbia | Central Europe | both | 77.25 | 49.98 | 62.21 | 38.74 |
| Seychelles | Southeast Asia | both | 24.27 | 13.02 | 21.26 | 11.38 |
| Sierra Leone | Western sub-Saharan Africa | both | 38.93 | 23.00 | 33.31 | 20.04 |
| Singapore | High-income Asia Pacific | both | 38.87 | 21.35 | 35.66 | 19.33 |
| Slovakia | Central Europe | both | 77.32 | 50.05 | 61.34 | 38.09 |
| Slovenia | Central Europe | both | 72.97 | 45.65 | 59.66 | 36.45 |
| Solomon Islands | Oceania | both | 49.00 | 29.16 | 42.82 | 25.50 |
| Somalia | Eastern Sub-Saharan Africa | both | 32.91 | 19.71 | 31.91 | 20.49 |
| South Africa | Southern sub-Saharan Africa | both | 38.06 | 22.38 | 33.83 | 19.85 |
| Republic of Korea | High-income Asia Pacific | both | 47.80 | 27.76 | 43.16 | 24.15 |
| South Sudan | Eastern Sub-Saharan Africa | both | 27.98 | 15.73 | 26.04 | 14.97 |
| Spain | Western Europe | both | 248.79 | 196.25 | 231.70 | 172.52 |
| Sri Lanka | Southeast Asia | both | 43.71 | 28.13 | 35.93 | 22.46 |
| Sudan | North Africa and Middle East | both | 83.13 | 53.77 | 62.81 | 38.39 |
| Suriname | Caribbean | both | 58.52 | 35.79 | 52.51 | 31.81 |
| Eswatini | Southern sub-Saharan Africa | both | 42.54 | 25.90 | 36.12 | 21.46 |
| Sweden | Western Europe | both | 200.15 | 136.30 | 196.21 | 130.04 |
| Switzerland | Western Europe | both | 235.12 | 177.54 | 226.37 | 165.42 |
| Syrian Arab Republic | North Africa and Middle East | both | 80.92 | 51.95 | 63.59 | 39.05 |
| Taiwan (Province of China) | East Asia | both | 70.18 | 46.74 | 54.91 | 35.17 |
| Tajikistan | Central Asia | both | 84.25 | 56.96 | 71.09 | 46.67 |
| United Republic of Tanzania | Eastern Sub-Saharan Africa | both | 28.37 | 16.03 | 25.02 | 14.45 |
| Thailand | Southeast Asia | both | 26.62 | 14.48 | 22.96 | 12.52 |
| Bahamas | Caribbean | both | 55.82 | 33.70 | 51.67 | 31.20 |
| Gambia | Western sub-Saharan Africa | both | 39.70 | 23.93 | 32.76 | 19.49 |
| Timor-Leste | Southeast Asia | both | 31.92 | 18.17 | 26.28 | 14.66 |
| Togo | Western sub-Saharan Africa | both | 36.95 | 21.47 | 31.35 | 18.35 |
| Tonga | Oceania | both | 43.02 | 24.75 | 37.78 | 21.67 |
| Trinidad and Tobago | Caribbean | both | 55.00 | 32.99 | 49.18 | 29.18 |
| Tunisia | North Africa and Middle East | both | 76.76 | 48.23 | 60.81 | 36.70 |
| Turkey | North Africa and Middle East | both | 106.24 | 77.08 | 82.89 | 57.81 |
| Turkmenistan | Central Asia | both | 78.53 | 51.40 | 62.41 | 38.89 |
| Uganda | Eastern Sub-Saharan Africa | both | 29.15 | 16.47 | 24.79 | 14.16 |
| Ukraine | Eastern Europe | both | 73.18 | 46.07 | 63.41 | 39.59 |
| United Arab Emirates | North Africa and Middle East | both | 62.75 | 36.36 | 48.56 | 27.11 |
| United Kingdom | Western Europe | both | 242.35 | 181.32 | 224.40 | 158.76 |
| United States of America | High-income North America | both | 106.09 | 110.04 | 94.36 | 93.42 |
| Uruguay | Southern Latin America | both | 115.62 | 90.70 | 109.15 | 80.28 |
| Uzbekistan | Central Asia | both | 83.86 | 56.27 | 67.06 | 42.98 |
| Vanuatu | Oceania | both | 47.38 | 27.79 | 41.67 | 24.48 |
| Venezuela (Bolivarian Republic of) | Central Latin America | both | 22.87 | 12.55 | 20.76 | 11.53 |
| Viet nam | Southeast Asia | both | 30.05 | 17.00 | 24.70 | 13.80 |
| United States Virgin Islands | Caribbean | both | 55.55 | 33.44 | 48.41 | 28.71 |
| Yemen | North Africa and Middle East | both | 86.93 | 57.11 | 67.19 | 42.05 |
| Zambia | Eastern Sub-Saharan Africa | both | 28.03 | 15.86 | 24.44 | 13.91 |
| Zimbabwe | Southern sub-Saharan Africa | both | 40.81 | 24.45 | 37.93 | 23.24 |
| Monaco | Western Europe | both | 236.27 | 180.49 | 226.89 | 165.59 |
| San Marino | Western Europe | both | 241.34 | 186.66 | 230.26 | 169.89 |
| Saint Kitts and Nevis | Caribbean | both | 58.42 | 35.78 | 51.52 | 31.09 |
| Cook Islands | Oceania | both | 38.14 | 21.10 | 32.90 | 18.22 |
| Nauru | Oceania | both | 37.46 | 20.65 | 33.28 | 18.36 |
| Niue | Oceania | both | 39.76 | 22.27 | 34.20 | 19.10 |
| Palau | Oceania | both | 36.16 | 19.71 | 32.06 | 17.31 |
| Tokelau | Oceania | both | 40.80 | 23.12 | 34.51 | 19.26 |
| Tuvalu | Oceania | both | 41.77 | 23.91 | 36.14 | 20.43 |
| Afghanistan | North Africa and Middle East | male | 91.76 | 61.17 | 76.96 | 50.78 |
| Albania | Central Europe | male | 84.30 | 55.92 | 68.64 | 43.33 |
| Algeria | North Africa and Middle East | male | 72.10 | 44.95 | 57.68 | 34.62 |
| American Samoa | Oceania | male | 39.22 | 20.80 | 34.71 | 18.23 |
| Andorra | Western Europe | male | 240.41 | 183.01 | 232.60 | 169.81 |
| Angola | Central sub-Saharan Africa | male | 66.73 | 49.90 | 51.86 | 37.08 |
| Antigua and Barbuda | Caribbean | male | 56.14 | 34.72 | 50.56 | 30.99 |
| Argentina | Southern Latin America | male | 116.92 | 89.45 | 110.31 | 79.39 |
| Armenia | Central Asia | male | 81.06 | 52.47 | 65.78 | 40.60 |
| Australia | Australasia | male | 162.27 | 142.54 | 149.62 | 123.95 |
| Austria | Western Europe | male | 259.87 | 205.17 | 245.31 | 184.41 |
| Azerbaijan | Central Asia | male | 78.01 | 49.94 | 66.41 | 41.15 |
| Bahrain | North Africa and Middle East | male | 64.57 | 38.43 | 53.16 | 30.90 |
| Bangladesh | South Asia | male | 48.93 | 31.05 | 40.68 | 25.80 |
| Barbados | Caribbean | male | 53.16 | 32.35 | 49.35 | 30.08 |
| Belarus | Eastern Europe | male | 78.83 | 50.77 | 65.31 | 40.22 |
| Belgium | Western Europe | male | 264.79 | 211.53 | 248.40 | 187.63 |
| Belize | Caribbean | male | 66.75 | 44.15 | 56.90 | 36.31 |
| Benin | Western sub-Saharan Africa | male | 37.63 | 22.50 | 31.99 | 19.42 |
| Bermuda | Caribbean | male | 57.24 | 35.63 | 51.56 | 31.91 |
| Bhutan | South Asia | male | 50.84 | 33.07 | 42.64 | 27.38 |
| Bolivia (Plurinational State of) | Andean Latin America | male | 105.97 | 88.04 | 91.05 | 73.68 |
| Bosnia and Herzegovina | Central Europe | male | 85.74 | 57.40 | 67.74 | 42.40 |
| Botswana | Southern sub-Saharan Africa | male | 39.18 | 23.74 | 32.70 | 19.36 |
| Brazil | Tropical Latin America | male | 91.97 | 69.82 | 86.39 | 66.34 |
| Brunei Darussalam | High-income Asia Pacific | male | 40.19 | 21.85 | 38.28 | 20.41 |
| Bulgaria | Central Europe | male | 79.39 | 50.49 | 65.49 | 40.28 |
| Burkina Faso | Western sub-Saharan Africa | male | 40.31 | 24.71 | 34.95 | 22.16 |
| Burundi | Eastern Sub-Saharan Africa | male | 27.59 | 15.77 | 26.45 | 15.63 |
| Cambodia | Southeast Asia | male | 32.31 | 17.53 | 27.11 | 14.61 |
| Cameroon | Western sub-Saharan Africa | male | 34.22 | 19.54 | 28.80 | 16.56 |
| Canada | High-income North America | male | 99.40 | 98.39 | 91.93 | 86.74 |
| Cabo Verde | Western sub-Saharan Africa | male | 36.43 | 21.68 | 29.53 | 17.24 |
| Central African Republic | Central sub-Saharan Africa | male | 66.34 | 49.21 | 58.26 | 43.36 |
| Chad | Western sub-Saharan Africa | male | 41.57 | 26.22 | 35.88 | 23.02 |
| Chile | Southern Latin America | male | 117.45 | 90.34 | 107.45 | 76.41 |
| China | East Asia | male | 79.96 | 51.44 | 62.52 | 38.48 |
| Colombia | Central Latin America | male | 22.45 | 12.48 | 19.52 | 10.88 |
| Comoros | Eastern Sub-Saharan Africa | male | 27.01 | 15.46 | 24.39 | 14.18 |
| Congo | Central sub-Saharan Africa | male | 55.26 | 38.20 | 43.96 | 29.13 |
| Costa Rica | Central Latin America | male | 22.10 | 12.26 | 19.36 | 10.75 |
| Côte d'Ivoire | Western sub-Saharan Africa | male | 35.98 | 20.90 | 30.64 | 18.13 |
| Croatia | Central Europe | male | 77.78 | 49.00 | 64.85 | 39.61 |
| Cuba | Caribbean | male | 59.05 | 37.06 | 55.03 | 34.85 |
| Cyprus | Western Europe | male | 259.29 | 205.93 | 232.42 | 169.87 |
| Czechia | Central Europe | male | 78.87 | 50.16 | 64.57 | 39.28 |
| Democratic Republic of the Congo | Central sub-Saharan Africa | male | 61.77 | 44.52 | 52.86 | 37.96 |
| Denmark | Western Europe | male | 260.31 | 206.01 | 247.66 | 186.69 |
| Djibouti | Eastern Sub-Saharan Africa | male | 27.22 | 15.69 | 24.06 | 13.96 |
| Dominica | Caribbean | male | 57.53 | 35.84 | 50.95 | 31.35 |
| Dominican Republic | Caribbean | male | 65.90 | 43.21 | 57.49 | 36.95 |
| Ecuador | Andean Latin America | male | 88.40 | 68.10 | 76.33 | 56.30 |
| Egypt | North Africa and Middle East | male | 47.38 | 25.57 | 36.81 | 19.46 |
| El Salvador | Central Latin America | male | 25.41 | 14.17 | 21.66 | 12.24 |
| Equatorial Guinea | Central sub-Saharan Africa | male | 66.42 | 49.57 | 41.18 | 26.78 |
| Eritrea | Eastern Sub-Saharan Africa | male | 29.65 | 17.19 | 25.92 | 15.37 |
| Estonia | Eastern Europe | male | 76.32 | 48.04 | 63.12 | 38.11 |
| Ethiopia | Eastern Sub-Saharan Africa | male | 29.02 | 17.17 | 25.58 | 15.31 |
| Micronesia (Federated States of) | Oceania | male | 43.24 | 23.45 | 37.58 | 20.20 |
| Fiji | Oceania | male | 43.03 | 23.34 | 37.23 | 20.02 |
| Finland | Western Europe | male | 280.26 | 229.76 | 263.99 | 205.89 |
| France | Western Europe | male | 298.48 | 253.16 | 281.00 | 227.28 |
| Gabon | Central sub-Saharan Africa | male | 53.87 | 36.84 | 41.13 | 26.66 |
| Georgia | Central Asia | male | 75.27 | 47.31 | 66.40 | 41.10 |
| Germany | Western Europe | male | 214.72 | 152.87 | 208.76 | 142.59 |
| Ghana | Western sub-Saharan Africa | male | 40.19 | 24.86 | 33.83 | 21.16 |
| Greece | Western Europe | male | 262.36 | 208.88 | 244.62 | 183.96 |
| Greenland | High-income North America | male | 118.91 | 122.50 | 107.73 | 107.07 |
| Grenada | Caribbean | male | 61.74 | 39.44 | 52.69 | 32.78 |
| Guam | Oceania | male | 35.95 | 18.81 | 31.66 | 16.54 |
| Guatemala | Central Latin America | male | 27.72 | 15.83 | 22.97 | 13.16 |
| Guinea | Western sub-Saharan Africa | male | 38.85 | 23.67 | 33.47 | 20.77 |
| Guinea-Bissau | Western sub-Saharan Africa | male | 38.96 | 23.57 | 33.09 | 20.37 |
| Guyana | Caribbean | male | 58.87 | 36.53 | 52.43 | 32.19 |
| Haiti | Caribbean | male | 72.70 | 49.19 | 65.78 | 44.24 |
| Honduras | Central Latin America | male | 27.30 | 15.65 | 23.57 | 13.56 |
| Hungary | Central Europe | male | 79.68 | 50.93 | 65.51 | 40.39 |
| Iceland | Western Europe | male | 279.28 | 229.83 | 260.63 | 202.57 |
| India | South Asia | male | 50.56 | 32.26 | 47.12 | 31.85 |
| Indonesia | Southeast Asia | male | 14.57 | 7.37 | 12.95 | 6.60 |
| Iran (Islamic Republic of) | North Africa and Middle East | male | 76.09 | 47.68 | 57.93 | 34.49 |
| Iraq | North Africa and Middle East | male | 85.57 | 56.60 | 66.21 | 41.50 |
| Ireland | Western Europe | male | 271.37 | 219.11 | 248.18 | 187.20 |
| Israel | Western Europe | male | 247.25 | 191.40 | 233.95 | 171.78 |
| Italy | Western Europe | male | 206.66 | 182.69 | 193.56 | 169.62 |
| Jamaica | Caribbean | male | 60.48 | 38.54 | 53.30 | 33.30 |
| Japan | High-income Asia Pacific | male | 43.49 | 24.41 | 44.30 | 24.79 |
| Jordan | North Africa and Middle East | male | 68.87 | 42.00 | 55.04 | 32.59 |
| Kazakhstan | Central Asia | male | 81.25 | 52.62 | 67.65 | 41.87 |
| Kenya | Eastern Sub-Saharan Africa | male | 24.34 | 13.84 | 22.18 | 12.84 |
| Kiribati | Oceania | male | 43.07 | 23.10 | 40.40 | 22.11 |
| Kuwait | North Africa and Middle East | male | 59.45 | 34.38 | 49.23 | 28.03 |
| Kyrgyzstan | Central Asia | male | 83.43 | 55.02 | 72.85 | 46.84 |
| Lao People's Democratic Republic | Southeast Asia | male | 33.19 | 18.14 | 27.74 | 15.03 |
| Latvia | Eastern Europe | male | 75.44 | 47.06 | 62.99 | 38.23 |
| Lebanon | North Africa and Middle East | male | 72.06 | 44.49 | 55.96 | 33.06 |
| Lesotho | Southern sub-Saharan Africa | male | 44.85 | 28.56 | 38.35 | 23.75 |
| Liberia | Western sub-Saharan Africa | male | 36.48 | 21.22 | 31.62 | 18.80 |
| Libya | North Africa and Middle East | male | 68.74 | 42.02 | 53.34 | 30.84 |
| Lithuania | Eastern Europe | male | 75.24 | 47.04 | 61.77 | 36.96 |
| Luxembourg | Western Europe | male | 250.29 | 193.64 | 240.58 | 178.92 |
| North Macedonia | Central Europe | male | 79.95 | 51.46 | 65.97 | 40.95 |
| Madagascar | Eastern Sub-Saharan Africa | male | 28.73 | 16.70 | 26.34 | 15.84 |
| Malawi | Eastern Sub-Saharan Africa | male | 29.43 | 17.10 | 26.24 | 15.62 |
| Malaysia | Southeast Asia | male | 23.98 | 12.34 | 19.99 | 10.25 |
| Maldives | Southeast Asia | male | 29.19 | 15.47 | 23.22 | 12.17 |
| Mali | Western sub-Saharan Africa | male | 36.01 | 21.18 | 29.67 | 17.37 |
| Malta | Western Europe | male | 261.40 | 208.51 | 239.90 | 177.88 |
| Marshall Islands | Oceania | male | 45.76 | 25.37 | 38.99 | 21.19 |
| Mauritania | Western sub-Saharan Africa | male | 36.79 | 21.92 | 31.01 | 18.53 |
| Mauritius | Southeast Asia | male | 26.83 | 13.82 | 22.90 | 11.87 |
| Mexico | Central Latin America | male | 24.44 | 14.05 | 21.44 | 12.36 |
| Republic of Moldova | Eastern Europe | male | 76.96 | 49.08 | 65.55 | 40.64 |
| Mongolia | Central Asia | male | 87.92 | 59.14 | 72.03 | 45.77 |
| Montenegro | Central Europe | male | 75.88 | 47.32 | 64.13 | 39.09 |
| Morocco | North Africa and Middle East | male | 80.14 | 52.09 | 64.57 | 40.57 |
| Mozambique | Eastern Sub-Saharan Africa | male | 32.03 | 19.17 | 27.79 | 16.91 |
| Myanmar | Southeast Asia | male | 32.64 | 17.78 | 26.94 | 14.48 |
| Namibia | Southern sub-Saharan Africa | male | 38.78 | 23.29 | 33.32 | 19.93 |
| Nepal | South Asia | male | 47.34 | 29.21 | 36.44 | 21.59 |
| Netherlands | Western Europe | male | 257.69 | 204.30 | 244.54 | 183.91 |
| New Zealand | Australasia | male | 178.92 | 159.40 | 167.46 | 142.28 |
| Nicaragua | Central Latin America | male | 26.48 | 14.99 | 22.60 | 12.88 |
| Niger | Western sub-Saharan Africa | male | 42.64 | 27.37 | 38.21 | 25.61 |
| Nigeria | Western sub-Saharan Africa | male | 45.35 | 30.00 | 34.03 | 21.39 |
| Democratic People's Republic of Korea | East Asia | male | 94.28 | 65.83 | 77.58 | 53.22 |
| Northern Mariana Islands | Oceania | male | 36.04 | 18.87 | 33.83 | 17.85 |
| Norway | Western Europe | male | 192.20 | 155.04 | 181.59 | 142.71 |
| Oman | North Africa and Middle East | male | 71.57 | 44.22 | 49.90 | 28.43 |
| Pakistan | South Asia | male | 50.66 | 32.59 | 42.56 | 27.23 |
| Palestine | North Africa and Middle East | male | 81.85 | 53.07 | 60.87 | 36.82 |
| Panama | Central Latin America | male | 21.62 | 12.03 | 19.13 | 10.61 |
| Papua New Guinea | Oceania | male | 49.22 | 27.62 | 43.96 | 24.70 |
| Paraguay | Tropical Latin America | male | 94.50 | 73.96 | 89.32 | 70.82 |
| Peru | Andean Latin America | male | 93.21 | 73.35 | 81.16 | 61.97 |
| Philippines | Southeast Asia | male | 27.24 | 14.29 | 24.62 | 13.09 |
| Poland | Central Europe | male | 80.81 | 51.91 | 71.79 | 46.21 |
| Portugal | Western Europe | male | 276.82 | 225.48 | 253.64 | 193.87 |
| Puerto Rico | Caribbean | male | 53.41 | 32.28 | 47.85 | 28.71 |
| Qatar | North Africa and Middle East | male | 62.71 | 36.68 | 49.00 | 27.77 |
| Romania | Central Europe | male | 78.55 | 49.74 | 64.26 | 39.29 |
| Russian Federation | Eastern Europe | male | 71.62 | 43.43 | 63.44 | 38.49 |
| Rwanda | Eastern Sub-Saharan Africa | male | 24.60 | 13.71 | 21.99 | 12.48 |
| Saint Lucia | Caribbean | male | 60.15 | 37.98 | 52.86 | 32.94 |
| Saint Vincent and the Grenadines | Caribbean | male | 61.84 | 39.57 | 54.38 | 34.27 |
| Samoa | Oceania | male | 41.99 | 22.84 | 39.35 | 21.62 |
| Sao Tome and Principe | Central sub-Saharan Africa | male | 33.68 | 19.43 | 27.86 | 16.04 |
| Saudi Arabia | North Africa and Middle East | male | 75.97 | 48.32 | 50.18 | 28.51 |
| Senegal | Western sub-Saharan Africa | male | 37.93 | 22.84 | 32.58 | 20.00 |
| Serbia | Central Europe | male | 80.37 | 51.87 | 65.87 | 40.44 |
| Seychelles | Southeast Asia | male | 23.91 | 12.27 | 20.91 | 10.73 |
| Sierra Leone | Western sub-Saharan Africa | male | 37.75 | 22.43 | 32.69 | 19.95 |
| Singapore | High-income Asia Pacific | male | 38.73 | 20.92 | 36.25 | 19.24 |
| Slovakia | Central Europe | male | 80.40 | 51.78 | 64.86 | 39.69 |
| Slovenia | Central Europe | male | 75.87 | 46.99 | 63.01 | 37.99 |
| Solomon Islands | Oceania | male | 50.36 | 28.63 | 44.05 | 24.97 |
| Somalia | Eastern Sub-Saharan Africa | male | 31.91 | 19.44 | 31.03 | 20.04 |
| South Africa | Southern sub-Saharan Africa | male | 36.87 | 21.90 | 33.01 | 19.68 |
| Republic of Korea | High-income Asia Pacific | male | 47.65 | 27.11 | 43.82 | 24.22 |
| South Sudan | Eastern Sub-Saharan Africa | male | 27.15 | 15.40 | 25.24 | 14.65 |
| Spain | Western Europe | male | 248.45 | 192.02 | 231.49 | 169.35 |
| Sri Lanka | Southeast Asia | male | 43.70 | 26.50 | 35.99 | 20.97 |
| Sudan | North Africa and Middle East | male | 79.82 | 51.47 | 60.43 | 36.82 |
| Suriname | Caribbean | male | 57.03 | 35.47 | 51.15 | 31.31 |
| Eswatini | Southern sub-Saharan Africa | male | 41.23 | 25.38 | 35.28 | 21.19 |
| Sweden | Western Europe | male | 198.86 | 134.39 | 195.45 | 127.85 |
| Switzerland | Western Europe | male | 239.34 | 180.14 | 231.27 | 168.33 |
| Syrian Arab Republic | North Africa and Middle East | male | 77.72 | 49.94 | 61.09 | 37.28 |
| Taiwan (Province of China) | East Asia | male | 78.14 | 50.51 | 60.98 | 37.99 |
| Tajikistan | Central Asia | male | 86.57 | 58.14 | 74.15 | 47.92 |
| United Republic of Tanzania | Eastern Sub-Saharan Africa | male | 27.43 | 15.69 | 24.25 | 14.18 |
| Thailand | Southeast Asia | male | 26.32 | 13.64 | 22.65 | 11.76 |
| Bahamas | Caribbean | male | 54.39 | 33.26 | 50.35 | 30.89 |
| Gambia | Western sub-Saharan Africa | male | 38.55 | 23.53 | 32.09 | 19.43 |
| Timor-Leste | Southeast Asia | male | 31.61 | 17.01 | 25.89 | 13.65 |
| Togo | Western sub-Saharan Africa | male | 35.81 | 21.02 | 30.71 | 18.25 |
| Tonga | Oceania | male | 44.50 | 24.50 | 38.95 | 21.24 |
| Trinidad and Tobago | Caribbean | male | 53.62 | 32.65 | 47.95 | 28.80 |
| Tunisia | North Africa and Middle East | male | 73.65 | 46.29 | 58.40 | 35.18 |
| Turkey | North Africa and Middle East | male | 98.21 | 69.82 | 78.29 | 53.07 |
| Turkmenistan | Central Asia | male | 80.73 | 52.53 | 64.93 | 39.87 |
| Uganda | Eastern Sub-Saharan Africa | male | 28.22 | 16.07 | 24.04 | 13.95 |
| Ukraine | Eastern Europe | male | 74.29 | 45.90 | 64.76 | 39.45 |
| United Arab Emirates | North Africa and Middle East | male | 60.91 | 35.28 | 46.65 | 26.00 |
| United Kingdom | Western Europe | male | 269.46 | 208.78 | 250.60 | 182.85 |
| United States of America | High-income North America | male | 108.90 | 106.58 | 95.86 | 90.11 |
| Uruguay | Southern Latin America | male | 115.24 | 87.99 | 109.19 | 78.34 |
| Uzbekistan | Central Asia | male | 86.11 | 57.74 | 69.99 | 44.19 |
| Vanuatu | Oceania | male | 48.62 | 27.13 | 42.78 | 23.97 |
| Venezuela (Bolivarian Republic of) | Central Latin America | male | 22.31 | 12.32 | 20.29 | 11.37 |
| Viet nam | Southeast Asia | male | 29.81 | 15.97 | 24.38 | 12.96 |
| United States Virgin Islands | Caribbean | male | 54.14 | 32.97 | 47.14 | 28.29 |
| Yemen | North Africa and Middle East | male | 83.65 | 55.08 | 64.98 | 40.68 |
| Zambia | Eastern Sub-Saharan Africa | male | 27.11 | 15.55 | 23.73 | 13.61 |
| Zimbabwe | Southern sub-Saharan Africa | male | 39.67 | 24.11 | 36.97 | 22.91 |
| Monaco | Western Europe | male | 240.87 | 183.75 | 231.96 | 168.81 |
| San Marino | Western Europe | male | 245.71 | 189.15 | 235.94 | 173.10 |
| Saint Kitts and Nevis | Caribbean | male | 56.94 | 35.28 | 50.23 | 30.63 |
| Cook Islands | Oceania | male | 39.12 | 20.84 | 33.85 | 17.88 |
| Nauru | Oceania | male | 38.36 | 20.29 | 34.23 | 18.02 |
| Niue | Oceania | male | 40.98 | 21.91 | 35.22 | 18.84 |
| Palau | Oceania | male | 37.14 | 19.41 | 32.77 | 17.06 |
| Tokelau | Oceania | male | 42.33 | 23.01 | 35.48 | 18.99 |
| Tuvalu | Oceania | male | 43.30 | 23.60 | 37.06 | 20.10 |
| Afghanistan | North Africa and Middle East | female | 98.88 | 66.90 | 80.54 | 54.25 |
| Albania | Central Europe | female | 77.65 | 52.15 | 60.87 | 39.58 |
| Algeria | North Africa and Middle East | female | 78.33 | 48.63 | 62.58 | 37.81 |
| American Samoa | Oceania | female | 37.16 | 21.44 | 32.86 | 18.98 |
| Andorra | Western Europe | female | 231.53 | 177.25 | 222.83 | 164.45 |
| Angola | Central sub-Saharan Africa | female | 70.04 | 51.53 | 54.27 | 37.84 |
| Antigua and Barbuda | Caribbean | female | 59.08 | 35.58 | 53.23 | 32.06 |
| Argentina | Southern Latin America | female | 117.86 | 95.63 | 110.29 | 83.67 |
| Armenia | Central Asia | female | 76.89 | 50.72 | 60.34 | 38.59 |
| Australia | Australasia | female | 168.05 | 167.83 | 156.49 | 146.81 |
| Austria | Western Europe | female | 248.86 | 199.51 | 234.17 | 178.57 |
| Azerbaijan | Central Asia | female | 73.82 | 47.86 | 61.03 | 39.10 |
| Bahrain | North Africa and Middle East | female | 70.48 | 41.60 | 58.27 | 33.92 |
| Bangladesh | South Asia | female | 50.89 | 31.21 | 42.42 | 26.04 |
| Barbados | Caribbean | female | 55.79 | 33.20 | 51.98 | 31.15 |
| Belarus | Eastern Europe | female | 76.67 | 51.47 | 62.57 | 40.74 |
| Belgium | Western Europe | female | 253.54 | 205.37 | 237.15 | 182.01 |
| Belize | Caribbean | female | 70.15 | 45.05 | 59.77 | 37.36 |
| Benin | Western sub-Saharan Africa | female | 39.84 | 23.35 | 33.20 | 19.66 |
| Bermuda | Caribbean | female | 60.08 | 36.43 | 54.18 | 32.77 |
| Bhutan | South Asia | female | 52.82 | 33.08 | 44.38 | 27.92 |
| Bolivia (Plurinational State of) | Andean Latin America | female | 111.64 | 90.02 | 96.15 | 75.27 |
| Bosnia and Herzegovina | Central Europe | female | 78.91 | 53.70 | 60.15 | 39.08 |
| Botswana | Southern sub-Saharan Africa | female | 41.41 | 24.45 | 34.33 | 19.77 |
| Brazil | Tropical Latin America | female | 96.81 | 71.09 | 90.89 | 67.71 |
| Brunei Darussalam | High-income Asia Pacific | female | 40.54 | 22.62 | 37.01 | 20.62 |
| Bulgaria | Central Europe | female | 73.19 | 47.41 | 58.20 | 37.00 |
| Burkina Faso | Western sub-Saharan Africa | female | 42.71 | 25.93 | 36.31 | 22.47 |
| Burundi | Eastern Sub-Saharan Africa | female | 29.37 | 16.49 | 28.03 | 16.45 |
| Cambodia | Southeast Asia | female | 32.93 | 19.63 | 27.81 | 16.60 |
| Cameroon | Western sub-Saharan Africa | female | 36.21 | 20.43 | 29.88 | 16.75 |
| Canada | High-income North America | female | 95.71 | 106.20 | 88.99 | 93.72 |
| Cabo Verde | Western sub-Saharan Africa | female | 38.65 | 22.54 | 30.63 | 17.51 |
| Central African Republic | Central sub-Saharan Africa | female | 69.62 | 50.65 | 61.08 | 44.54 |
| Chad | Western sub-Saharan Africa | female | 43.99 | 27.24 | 37.23 | 23.37 |
| Chile | Southern Latin America | female | 118.38 | 95.51 | 107.57 | 80.43 |
| China | East Asia | female | 69.20 | 50.56 | 54.19 | 38.21 |
| Colombia | Central Latin America | female | 23.58 | 12.90 | 20.42 | 11.24 |
| Comoros | Eastern Sub-Saharan Africa | female | 28.82 | 16.23 | 25.83 | 14.81 |
| Congo | Central sub-Saharan Africa | female | 58.03 | 38.81 | 45.93 | 29.70 |
| Costa Rica | Central Latin America | female | 23.19 | 12.71 | 20.25 | 11.09 |
| Côte d'Ivoire | Western sub-Saharan Africa | female | 38.14 | 21.80 | 31.80 | 18.43 |
| Croatia | Central Europe | female | 71.73 | 45.70 | 57.65 | 36.42 |
| Cuba | Caribbean | female | 62.10 | 37.76 | 57.93 | 35.78 |
| Cyprus | Western Europe | female | 248.37 | 199.06 | 222.80 | 164.78 |
| Czechia | Central Europe | female | 72.64 | 46.92 | 57.32 | 36.15 |
| Democratic Republic of the Congo | Central sub-Saharan Africa | female | 64.87 | 45.38 | 55.36 | 38.82 |
| Denmark | Western Europe | female | 249.74 | 200.17 | 236.11 | 180.67 |
| Djibouti | Eastern Sub-Saharan Africa | female | 29.03 | 16.43 | 25.51 | 14.57 |
| Dominica | Caribbean | female | 60.43 | 36.79 | 53.57 | 32.04 |
| Dominican Republic | Caribbean | female | 69.29 | 44.46 | 60.36 | 37.91 |
| Ecuador | Andean Latin America | female | 93.07 | 69.53 | 80.13 | 57.61 |
| Egypt | North Africa and Middle East | female | 48.20 | 25.38 | 39.98 | 20.75 |
| El Salvador | Central Latin America | female | 26.67 | 14.84 | 22.65 | 12.56 |
| Equatorial Guinea | Central sub-Saharan Africa | female | 69.72 | 50.61 | 43.07 | 27.08 |
| Eritrea | Eastern Sub-Saharan Africa | female | 31.55 | 18.26 | 27.47 | 16.07 |
| Estonia | Eastern Europe | female | 74.19 | 48.92 | 60.61 | 38.57 |
| Ethiopia | Eastern Sub-Saharan Africa | female | 30.79 | 17.91 | 27.00 | 15.86 |
| Micronesia (Federated States of) | Oceania | female | 40.94 | 24.39 | 35.55 | 21.06 |
| Fiji | Oceania | female | 40.67 | 24.11 | 35.20 | 20.94 |
| Finland | Western Europe | female | 267.55 | 223.37 | 251.19 | 199.58 |
| France | Western Europe | female | 294.68 | 260.96 | 273.97 | 230.42 |
| Gabon | Central sub-Saharan Africa | female | 56.46 | 37.34 | 42.97 | 27.05 |
| Georgia | Central Asia | female | 71.30 | 45.89 | 60.88 | 39.05 |
| Germany | Western Europe | female | 204.46 | 143.51 | 197.63 | 133.81 |
| Ghana | Western sub-Saharan Africa | female | 42.84 | 26.18 | 35.22 | 21.36 |
| Greece | Western Europe | female | 251.37 | 202.37 | 233.60 | 177.36 |
| Greenland | High-income North America | female | 113.00 | 130.33 | 102.95 | 114.28 |
| Grenada | Caribbean | female | 64.99 | 40.69 | 55.36 | 33.58 |
| Guam | Oceania | female | 34.08 | 19.42 | 29.94 | 16.90 |
| Guatemala | Central Latin America | female | 29.08 | 16.39 | 24.01 | 13.50 |
| Guinea | Western sub-Saharan Africa | female | 41.20 | 24.66 | 34.75 | 20.88 |
| Guinea-Bissau | Western sub-Saharan Africa | female | 41.25 | 24.74 | 34.35 | 20.44 |
| Guyana | Caribbean | female | 61.90 | 37.42 | 55.08 | 32.94 |
| Haiti | Caribbean | female | 76.55 | 50.36 | 68.87 | 45.21 |
| Honduras | Central Latin America | female | 28.59 | 16.19 | 24.64 | 13.92 |
| Hungary | Central Europe | female | 73.49 | 47.57 | 58.13 | 37.04 |
| Iceland | Western Europe | female | 266.32 | 223.09 | 248.27 | 197.30 |
| India | South Asia | female | 52.57 | 32.67 | 48.94 | 32.12 |
| Indonesia | Southeast Asia | female | 15.36 | 8.74 | 13.77 | 7.93 |
| Iran (Islamic Republic of) | North Africa and Middle East | female | 82.62 | 51.91 | 62.87 | 37.66 |
| Iraq | North Africa and Middle East | female | 90.60 | 59.99 | 68.92 | 43.41 |
| Ireland | Western Europe | female | 259.09 | 212.50 | 236.99 | 181.35 |
| Israel | Western Europe | female | 237.77 | 185.20 | 224.08 | 166.00 |
| Italy | Western Europe | female | 193.36 | 185.60 | 179.44 | 172.70 |
| Jamaica | Caribbean | female | 63.49 | 39.56 | 55.99 | 34.24 |
| Japan | High-income Asia Pacific | female | 43.86 | 25.41 | 42.79 | 24.79 |
| Jordan | North Africa and Middle East | female | 74.93 | 45.69 | 60.04 | 35.44 |
| Kazakhstan | Central Asia | female | 76.95 | 50.56 | 62.11 | 39.94 |
| Kenya | Eastern Sub-Saharan Africa | female | 26.29 | 14.75 | 23.47 | 13.27 |
| Kiribati | Oceania | female | 40.72 | 24.08 | 38.18 | 23.20 |
| Kuwait | North Africa and Middle East | female | 64.94 | 37.27 | 54.41 | 30.80 |
| Kyrgyzstan | Central Asia | female | 79.05 | 52.58 | 66.90 | 44.31 |
| Lao People's Democratic Republic | Southeast Asia | female | 33.79 | 20.57 | 28.40 | 17.17 |
| Latvia | Eastern Europe | female | 73.33 | 47.86 | 60.52 | 38.42 |
| Lebanon | North Africa and Middle East | female | 78.42 | 48.56 | 60.97 | 36.17 |
| Lesotho | Southern sub-Saharan Africa | female | 47.52 | 29.61 | 40.25 | 24.28 |
| Liberia | Western sub-Saharan Africa | female | 38.68 | 21.99 | 32.81 | 19.02 |
| Libya | North Africa and Middle East | female | 74.86 | 45.53 | 58.32 | 33.78 |
| Lithuania | Eastern Europe | female | 73.10 | 47.57 | 59.40 | 37.35 |
| Luxembourg | Western Europe | female | 240.52 | 187.82 | 229.92 | 172.77 |
| North Macedonia | Central Europe | female | 73.73 | 48.12 | 58.62 | 37.32 |
| Madagascar | Eastern Sub-Saharan Africa | female | 30.62 | 17.65 | 27.90 | 16.39 |
| Malawi | Eastern Sub-Saharan Africa | female | 31.34 | 18.05 | 27.78 | 16.26 |
| Malaysia | Southeast Asia | female | 24.55 | 13.75 | 20.67 | 11.61 |
| Maldives | Southeast Asia | female | 29.84 | 17.34 | 23.95 | 13.70 |
| Mali | Western sub-Saharan Africa | female | 37.78 | 21.62 | 30.98 | 17.56 |
| Malta | Western Europe | female | 250.92 | 201.73 | 229.25 | 171.85 |
| Marshall Islands | Oceania | female | 43.25 | 26.25 | 36.91 | 22.13 |
| Mauritania | Western sub-Saharan Africa | female | 38.98 | 22.94 | 32.19 | 18.95 |
| Mauritius | Southeast Asia | female | 27.49 | 15.76 | 23.62 | 13.46 |
| Mexico | Central Latin America | female | 25.66 | 14.46 | 22.41 | 12.70 |
| Republic of Moldova | Eastern Europe | female | 74.72 | 49.43 | 62.95 | 40.87 |
| Mongolia | Central Asia | female | 83.19 | 56.68 | 66.15 | 43.79 |
| Montenegro | Central Europe | female | 70.01 | 44.16 | 57.06 | 35.88 |
| Morocco | North Africa and Middle East | female | 86.59 | 55.98 | 69.07 | 43.41 |
| Mozambique | Eastern Sub-Saharan Africa | female | 34.06 | 20.23 | 29.40 | 17.52 |
| Myanmar | Southeast Asia | female | 33.28 | 20.13 | 27.68 | 16.56 |
| Namibia | Southern sub-Saharan Africa | female | 40.97 | 24.13 | 34.93 | 20.42 |
| Nepal | South Asia | female | 49.27 | 29.69 | 37.93 | 22.07 |
| Netherlands | Western Europe | female | 247.40 | 198.08 | 233.82 | 177.98 |
| New Zealand | Australasia | female | 186.46 | 187.00 | 174.90 | 168.02 |
| Nicaragua | Central Latin America | female | 27.80 | 15.57 | 23.60 | 13.28 |
| Niger | Western sub-Saharan Africa | female | 45.12 | 28.36 | 39.62 | 25.72 |
| Nigeria | Western sub-Saharan Africa | female | 47.89 | 31.17 | 35.58 | 21.78 |
| Democratic People's Republic of Korea | East Asia | female | 77.31 | 60.94 | 63.73 | 49.17 |
| Northern Mariana Islands | Oceania | female | 34.15 | 19.41 | 31.99 | 18.54 |
| Norway | Western Europe | female | 192.72 | 159.96 | 182.57 | 147.70 |
| Oman | North Africa and Middle East | female | 77.70 | 48.23 | 55.03 | 31.30 |
| Pakistan | South Asia | female | 52.65 | 32.94 | 44.35 | 27.64 |
| Palestine | North Africa and Middle East | female | 88.51 | 57.79 | 65.62 | 40.25 |
| Panama | Central Latin America | female | 22.75 | 12.33 | 20.02 | 10.93 |
| Papua New Guinea | Oceania | female | 46.39 | 28.63 | 41.52 | 25.89 |
| Paraguay | Tropical Latin America | female | 99.37 | 75.02 | 93.92 | 72.13 |
| Peru | Andean Latin America | female | 98.11 | 74.73 | 85.55 | 63.46 |
| Philippines | Southeast Asia | female | 27.82 | 16.04 | 25.32 | 14.82 |
| Poland | Central Europe | female | 77.69 | 51.85 | 67.55 | 46.97 |
| Portugal | Western Europe | female | 264.49 | 218.90 | 241.85 | 188.71 |
| Puerto Rico | Caribbean | female | 56.07 | 33.21 | 50.35 | 29.66 |
| Qatar | North Africa and Middle East | female | 68.45 | 40.08 | 54.11 | 30.39 |
| Romania | Central Europe | female | 70.11 | 44.33 | 54.90 | 33.94 |
| Russian Federation | Eastern Europe | female | 69.39 | 44.02 | 60.46 | 39.00 |
| Rwanda | Eastern Sub-Saharan Africa | female | 26.37 | 14.42 | 23.36 | 12.94 |
| Saint Lucia | Caribbean | female | 63.16 | 38.90 | 55.50 | 33.71 |
| Saint Vincent and the Grenadines | Caribbean | female | 64.97 | 40.59 | 57.08 | 34.85 |
| Samoa | Oceania | female | 39.74 | 23.62 | 37.19 | 22.52 |
| Sao Tome and Principe | Central sub-Saharan Africa | female | 35.69 | 20.06 | 28.92 | 16.24 |
| Saudi Arabia | North Africa and Middle East | female | 82.40 | 52.69 | 55.33 | 31.44 |
| Senegal | Western sub-Saharan Africa | female | 40.21 | 23.69 | 33.82 | 20.17 |
| Serbia | Central Europe | female | 74.03 | 48.09 | 58.47 | 37.06 |
| Seychelles | Southeast Asia | female | 24.52 | 13.75 | 21.62 | 12.16 |
| Sierra Leone | Western sub-Saharan Africa | female | 40.00 | 23.52 | 33.91 | 20.12 |
| Singapore | High-income Asia Pacific | female | 39.09 | 21.85 | 35.05 | 19.36 |
| Slovakia | Central Europe | female | 74.19 | 48.42 | 57.65 | 36.42 |
| Slovenia | Central Europe | female | 69.93 | 44.19 | 55.97 | 34.74 |
| Solomon Islands | Oceania | female | 47.42 | 29.63 | 41.60 | 26.02 |
| Somalia | Eastern Sub-Saharan Africa | female | 33.91 | 19.99 | 32.83 | 20.97 |
| South Africa | Southern sub-Saharan Africa | female | 39.20 | 22.84 | 34.66 | 20.02 |
| Republic of Korea | High-income Asia Pacific | female | 47.96 | 28.44 | 42.42 | 24.09 |
| South Sudan | Eastern Sub-Saharan Africa | female | 28.91 | 16.11 | 26.74 | 15.26 |
| Spain | Western Europe | female | 249.30 | 200.78 | 232.11 | 175.90 |
| Sri Lanka | Southeast Asia | female | 43.72 | 29.77 | 35.94 | 23.91 |
| Sudan | North Africa and Middle East | female | 86.34 | 55.96 | 65.23 | 40.00 |
| Suriname | Caribbean | female | 60.01 | 36.13 | 53.84 | 32.31 |
| Eswatini | Southern sub-Saharan Africa | female | 43.61 | 26.34 | 36.92 | 21.72 |
| Sweden | Western Europe | female | 201.51 | 138.33 | 197.11 | 132.50 |
| Switzerland | Western Europe | female | 230.61 | 174.90 | 221.42 | 162.59 |
| Syrian Arab Republic | North Africa and Middle East | female | 84.24 | 54.04 | 65.90 | 40.54 |
| Taiwan (Province of China) | East Asia | female | 61.90 | 42.83 | 49.05 | 32.50 |
| Tajikistan | Central Asia | female | 81.98 | 55.63 | 68.01 | 45.50 |
| United Republic of Tanzania | Eastern Sub-Saharan Africa | female | 29.20 | 16.33 | 25.71 | 14.68 |
| Thailand | Southeast Asia | female | 26.96 | 15.33 | 23.32 | 13.29 |
| Bahamas | Caribbean | female | 57.19 | 34.12 | 52.92 | 31.50 |
| Gambia | Western sub-Saharan Africa | female | 40.86 | 24.33 | 33.38 | 19.55 |
| Timor-Leste | Southeast Asia | female | 32.28 | 19.40 | 26.59 | 15.62 |
| Togo | Western sub-Saharan Africa | female | 37.93 | 21.85 | 31.93 | 18.45 |
| Tonga | Oceania | female | 42.05 | 25.21 | 36.82 | 22.12 |
| Trinidad and Tobago | Caribbean | female | 56.39 | 33.33 | 50.44 | 29.58 |
| Tunisia | North Africa and Middle East | female | 79.88 | 50.18 | 63.22 | 38.22 |
| Turkey | North Africa and Middle East | female | 114.55 | 84.59 | 87.73 | 62.82 |
| Turkmenistan | Central Asia | female | 76.50 | 50.39 | 59.49 | 37.85 |
| Uganda | Eastern Sub-Saharan Africa | female | 30.04 | 16.85 | 25.48 | 14.36 |
| Ukraine | Eastern Europe | female | 72.18 | 46.43 | 62.15 | 39.92 |
| United Arab Emirates | North Africa and Middle East | female | 66.48 | 38.31 | 51.91 | 28.90 |
| United Kingdom | Western Europe | female | 215.58 | 154.02 | 197.98 | 134.56 |
| United States of America | High-income North America | female | 103.45 | 113.77 | 92.93 | 96.96 |
| Uruguay | Southern Latin America | female | 116.14 | 93.51 | 109.32 | 82.37 |
| Uzbekistan | Central Asia | female | 81.62 | 54.80 | 64.20 | 41.90 |
| Vanuatu | Oceania | female | 45.78 | 28.28 | 40.42 | 24.89 |
| Venezuela (Bolivarian Republic of) | Central Latin America | female | 23.42 | 12.77 | 21.21 | 11.68 |
| Viet nam | Southeast Asia | female | 30.43 | 18.01 | 25.06 | 14.68 |
| United States Virgin Islands | Caribbean | female | 56.81 | 33.86 | 49.57 | 29.10 |
| Yemen | North Africa and Middle East | female | 90.33 | 59.14 | 69.48 | 43.45 |
| Zambia | Eastern Sub-Saharan Africa | female | 28.87 | 16.15 | 25.14 | 14.22 |
| Zimbabwe | Southern sub-Saharan Africa | female | 41.85 | 24.76 | 38.79 | 23.54 |
| Monaco | Western Europe | female | 231.99 | 177.70 | 222.30 | 163.27 |
| San Marino | Western Europe | female | 236.43 | 183.24 | 225.72 | 167.80 |
| Saint Kitts and Nevis | Caribbean | female | 59.88 | 36.28 | 52.84 | 31.55 |
| Cook Islands | Oceania | female | 37.01 | 21.33 | 32.05 | 18.51 |
| Nauru | Oceania | female | 36.32 | 20.91 | 32.41 | 18.75 |
| Niue | Oceania | female | 38.79 | 22.82 | 33.29 | 19.43 |
| Palau | Oceania | female | 35.17 | 20.07 | 31.03 | 17.66 |
| Tokelau | Oceania | female | 40.02 | 23.58 | 33.53 | 19.57 |
| Tuvalu | Oceania | female | 40.95 | 24.42 | 35.11 | 20.83 |

**Supplementary Table 6. The number and its trend of Incidence and DALY for global psoriasis burden of young adults in 1990 and 2019, by countries and regions and gender.**

| Country | Region | Sex | incidence 1990 | incidence 2019 | nDALY 1990 | nDALY 2019 | incidence EAPC | DALY EAPC |
| --- | --- | --- | --- | --- | --- | --- | --- | --- |
| Afghanistan | North Africa and Middle East | both | 4216.33 | 13509.85 | 2798.96 | 8840.95 | 3.20 | 3.16 |
| Albania | Central Europe | both | 1340.69 | 839.70 | 895.48 | 537.61 | 0.63 | 0.60 |
| Algeria | North Africa and Middle East | both | 8303.54 | 13717.96 | 5084.55 | 8286.22 | 1.65 | 1.63 |
| American Samoa | Oceania | both | 8.73 | 8.96 | 4.83 | 4.97 | 1.03 | 1.03 |
| Andorra | Western Europe | both | 78.05 | 99.65 | 59.74 | 75.53 | 1.28 | 1.26 |
| Angola | Central sub-Saharan Africa | both | 3059.57 | 6889.92 | 2269.32 | 4871.39 | 2.25 | 2.15 |
| Antigua and Barbuda | Caribbean | both | 17.57 | 25.39 | 10.71 | 15.40 | 1.45 | 1.44 |
| Argentina | Southern Latin America | both | 18541.89 | 25794.01 | 14619.64 | 19103.27 | 1.39 | 1.31 |
| Armenia | Central Asia | both | 1307.21 | 949.32 | 853.12 | 593.19 | 0.73 | 0.70 |
| Australia | Australasia | both | 14942.22 | 18120.68 | 14102.50 | 16209.44 | 1.21 | 1.15 |
| Austria | Western Europe | both | 10389.03 | 10069.95 | 8282.33 | 7742.94 | 0.97 | 0.93 |
| Azerbaijan | Central Asia | both | 2642.61 | 3580.34 | 1703.81 | 2247.69 | 1.35 | 1.32 |
| Bahrain | North Africa and Middle East | both | 202.52 | 548.29 | 118.94 | 328.10 | 2.71 | 2.76 |
| Bangladesh | South Asia | both | 23728.60 | 35357.84 | 14786.73 | 22027.62 | 1.49 | 1.49 |
| Barbados | Caribbean | both | 72.23 | 73.58 | 43.34 | 44.40 | 1.02 | 1.02 |
| Belarus | Eastern Europe | both | 3934.57 | 2936.66 | 2576.00 | 1833.64 | 0.75 | 0.71 |
| Belgium | Western Europe | both | 13061.51 | 12593.96 | 10558.98 | 9738.45 | 0.96 | 0.92 |
| Belize | Caribbean | both | 54.24 | 127.41 | 35.42 | 80.61 | 2.35 | 2.28 |
| Benin | Western sub-Saharan Africa | both | 739.07 | 1802.48 | 435.85 | 1079.43 | 2.44 | 2.48 |
| Bermuda | Caribbean | both | 20.34 | 15.72 | 12.43 | 9.59 | 0.77 | 0.77 |
| Bhutan | South Asia | both | 146.16 | 186.72 | 93.32 | 118.32 | 1.28 | 1.27 |
| Bolivia (Plurinational State of) | Andean Latin America | both | 3098.77 | 5621.23 | 2548.05 | 4472.41 | 1.81 | 1.76 |
| Bosnia and Herzegovina | Central Europe | both | 1978.71 | 1009.79 | 1333.05 | 642.70 | 0.51 | 0.48 |
| Botswana | Southern sub-Saharan Africa | both | 229.25 | 441.19 | 136.63 | 257.33 | 1.92 | 1.88 |
| Brazil | Tropical Latin America | both | 69771.56 | 104318.71 | 52137.93 | 78703.51 | 1.50 | 1.51 |
| Brunei Darussalam | High-income Asia Pacific | both | 57.63 | 102.31 | 31.48 | 55.63 | 1.78 | 1.77 |
| Bulgaria | Central Europe | both | 3246.97 | 2004.10 | 2081.41 | 1246.00 | 0.62 | 0.60 |
| Burkina Faso | Western sub-Saharan Africa | both | 1519.01 | 3483.18 | 924.02 | 2176.80 | 2.29 | 2.36 |
| Burundi | Eastern Sub-Saharan Africa | both | 657.70 | 1425.36 | 370.84 | 837.67 | 2.17 | 2.26 |
| Cambodia | Southeast Asia | both | 1387.57 | 2341.90 | 796.01 | 1331.74 | 1.69 | 1.67 |
| Cameroon | Western sub-Saharan Africa | both | 1514.98 | 4022.09 | 857.44 | 2277.43 | 2.65 | 2.66 |
| Canada | High-income North America | both | 14796.52 | 15166.18 | 15490.01 | 15243.00 | 1.02 | 0.98 |
| Cabo Verde | Western sub-Saharan Africa | both | 51.15 | 91.42 | 29.95 | 52.77 | 1.79 | 1.76 |
| Central African Republic | Central sub-Saharan Africa | both | 812.78 | 1466.47 | 596.47 | 1081.16 | 1.80 | 1.81 |
| Chad | Western sub-Saharan Africa | both | 1012.54 | 2348.16 | 631.04 | 1486.16 | 2.32 | 2.36 |
| Chile | Southern Latin America | both | 8205.60 | 10094.90 | 6415.79 | 7373.24 | 1.23 | 1.15 |
| China | East Asia | both | 476366.43 | 451290.33 | 322404.46 | 299176.04 | 0.95 | 0.93 |
| Colombia | Central Latin America | both | 3723.28 | 4974.01 | 2061.50 | 2753.61 | 1.34 | 1.34 |
| Comoros | Eastern Sub-Saharan Africa | both | 53.91 | 91.95 | 30.50 | 53.01 | 1.71 | 1.74 |
| Congo | Central sub-Saharan Africa | both | 590.85 | 1186.60 | 402.06 | 777.39 | 2.01 | 1.93 |
| Costa Rica | Central Latin America | both | 334.39 | 498.26 | 184.85 | 274.08 | 1.49 | 1.48 |
| Côte d'Ivoire | Western sub-Saharan Africa | both | 1955.55 | 3944.14 | 1123.28 | 2307.70 | 2.02 | 2.05 |
| Croatia | Central Europe | both | 1857.42 | 1192.52 | 1175.36 | 738.60 | 0.64 | 0.63 |
| Cuba | Caribbean | both | 3617.61 | 3134.79 | 2237.75 | 1956.43 | 0.87 | 0.87 |
| Cyprus | Western Europe | both | 1030.34 | 1637.88 | 822.06 | 1219.92 | 1.59 | 1.48 |
| Czechia | Central Europe | both | 4059.05 | 3143.70 | 2594.93 | 1936.54 | 0.77 | 0.75 |
| Democratic Republic of the Congo | Central sub-Saharan Africa | both | 10067.07 | 21554.43 | 7146.37 | 15313.13 | 2.14 | 2.14 |
| Denmark | Western Europe | both | 6967.86 | 6362.32 | 5604.10 | 4881.88 | 0.91 | 0.87 |
| Djibouti | Eastern Sub-Saharan Africa | both | 61.60 | 158.48 | 35.03 | 91.25 | 2.57 | 2.60 |
| Dominica | Caribbean | both | 19.77 | 17.79 | 12.22 | 10.80 | 0.90 | 0.88 |
| Dominican Republic | Caribbean | both | 2319.71 | 3369.04 | 1507.50 | 2140.63 | 1.45 | 1.42 |
| Ecuador | Andean Latin America | both | 4264.39 | 7079.97 | 3250.43 | 5160.72 | 1.66 | 1.59 |
| Egypt | North Africa and Middle East | both | 12320.65 | 19422.48 | 6534.51 | 10139.74 | 1.58 | 1.55 |
| El Salvador | Central Latin America | both | 612.00 | 712.68 | 342.27 | 399.40 | 1.16 | 1.17 |
| Equatorial Guinea | Central sub-Saharan Africa | both | 117.14 | 295.08 | 86.15 | 189.69 | 2.52 | 2.20 |
| Eritrea | Eastern Sub-Saharan Africa | both | 395.13 | 883.43 | 227.84 | 519.35 | 2.24 | 2.28 |
| Estonia | Eastern Europe | both | 580.01 | 379.62 | 371.72 | 232.74 | 0.65 | 0.63 |
| Ethiopia | Eastern Sub-Saharan Africa | both | 6284.94 | 13082.87 | 3670.56 | 7728.36 | 2.08 | 2.11 |
| Micronesia (Federated States of) | Oceania | both | 18.49 | 18.84 | 10.45 | 10.65 | 1.02 | 1.02 |
| Fiji | Oceania | both | 157.68 | 169.31 | 89.23 | 95.52 | 1.07 | 1.07 |
| Finland | Western Europe | both | 7321.55 | 6199.18 | 6156.27 | 4942.99 | 0.85 | 0.80 |
| France | Western Europe | both | 87228.77 | 81102.62 | 76003.53 | 67927.52 | 0.93 | 0.89 |
| Gabon | Central sub-Saharan Africa | both | 233.17 | 379.63 | 157.01 | 242.57 | 1.63 | 1.54 |
| Georgia | Central Asia | both | 1943.91 | 1088.20 | 1234.91 | 682.02 | 0.56 | 0.55 |
| Germany | Western Europe | both | 85659.35 | 75796.99 | 60896.34 | 52255.89 | 0.88 | 0.86 |
| Ghana | Western sub-Saharan Africa | both | 2710.77 | 5559.05 | 1662.01 | 3413.68 | 2.05 | 2.05 |
| Greece | Western Europe | both | 13083.19 | 11271.73 | 10519.91 | 8748.87 | 0.86 | 0.83 |
| Greenland | High-income North America | both | 40.22 | 28.73 | 42.28 | 29.84 | 0.71 | 0.71 |
| Grenada | Caribbean | both | 23.41 | 29.19 | 14.84 | 17.94 | 1.25 | 1.21 |
| Guam | Oceania | both | 26.03 | 25.01 | 14.08 | 13.63 | 0.96 | 0.97 |
| Guatemala | Central Latin America | both | 931.66 | 2153.06 | 530.51 | 1225.53 | 2.31 | 2.31 |
| Guinea | Western sub-Saharan Africa | both | 1013.64 | 1857.54 | 610.54 | 1130.75 | 1.83 | 1.85 |
| Guinea-Bissau | Western sub-Saharan Africa | both | 168.03 | 304.11 | 100.97 | 183.31 | 1.81 | 1.82 |
| Guyana | Caribbean | both | 229.99 | 218.29 | 141.20 | 132.56 | 0.95 | 0.94 |
| Haiti | Caribbean | both | 2095.60 | 4310.28 | 1398.63 | 2861.08 | 2.06 | 2.05 |
| Honduras | Central Latin America | both | 537.20 | 1196.77 | 307.44 | 684.72 | 2.23 | 2.23 |
| Hungary | Central Europe | both | 4007.11 | 2913.38 | 2568.56 | 1820.13 | 0.73 | 0.71 |
| Iceland | Western Europe | both | 360.68 | 424.97 | 298.51 | 336.34 | 1.18 | 1.13 |
| India | South Asia | both | 209681.04 | 360448.47 | 132054.33 | 240046.71 | 1.72 | 1.82 |
| Indonesia | Southeast Asia | both | 13328.17 | 19436.59 | 7201.71 | 10551.78 | 1.46 | 1.47 |
| Iran (Islamic Republic of) | North Africa and Middle East | both | 19728.57 | 29565.33 | 12191.74 | 17797.53 | 1.50 | 1.46 |
| Iraq | North Africa and Middle East | both | 6496.43 | 15170.65 | 4240.07 | 9428.11 | 2.34 | 2.22 |
| Ireland | Western Europe | both | 4677.86 | 5857.50 | 3801.31 | 4541.73 | 1.25 | 1.19 |
| Israel | Western Europe | both | 5791.09 | 10097.15 | 4476.56 | 7482.64 | 1.74 | 1.67 |
| Italy | Western Europe | both | 57909.44 | 49866.52 | 53431.95 | 47013.62 | 0.86 | 0.88 |
| Jamaica | Caribbean | both | 681.18 | 830.31 | 429.91 | 512.63 | 1.22 | 1.19 |
| Japan | High-income Asia Pacific | both | 29143.30 | 24061.44 | 16733.61 | 13824.29 | 0.83 | 0.83 |
| Jordan | North Africa and Middle East | both | 1180.52 | 3567.98 | 707.55 | 2098.92 | 3.02 | 2.97 |
| Kazakhstan | Central Asia | both | 6343.24 | 6119.91 | 4127.54 | 3847.42 | 0.96 | 0.93 |
| Kenya | Eastern Sub-Saharan Africa | both | 2398.24 | 5706.86 | 1351.05 | 3262.68 | 2.38 | 2.41 |
| Kiribati | Oceania | both | 14.38 | 22.85 | 8.10 | 13.18 | 1.59 | 1.63 |
| Kuwait | North Africa and Middle East | both | 650.55 | 1599.06 | 372.74 | 921.69 | 2.46 | 2.47 |
| Kyrgyzstan | Central Asia | both | 1623.68 | 2312.91 | 1074.59 | 1509.58 | 1.42 | 1.40 |
| Lao People's Democratic Republic | Southeast Asia | both | 584.11 | 1056.52 | 339.00 | 606.67 | 1.81 | 1.79 |
| Latvia | Eastern Europe | both | 971.17 | 534.71 | 617.09 | 327.59 | 0.55 | 0.53 |
| Lebanon | North Africa and Middle East | both | 1096.30 | 1597.90 | 673.40 | 947.26 | 1.46 | 1.41 |
| Lesotho | Southern sub-Saharan Africa | both | 363.33 | 437.86 | 228.35 | 267.61 | 1.21 | 1.17 |
| Liberia | Western sub-Saharan Africa | both | 297.43 | 772.06 | 170.94 | 452.94 | 2.60 | 2.65 |
| Libya | North Africa and Middle East | both | 1300.21 | 2337.67 | 783.67 | 1360.62 | 1.80 | 1.74 |
| Lithuania | Eastern Europe | both | 1362.47 | 764.07 | 866.71 | 465.46 | 0.56 | 0.54 |
| Luxembourg | Western Europe | both | 501.69 | 753.97 | 393.47 | 573.91 | 1.50 | 1.46 |
| North Macedonia | Central Europe | both | 801.76 | 700.55 | 519.06 | 439.22 | 0.87 | 0.85 |
| Madagascar | Eastern Sub-Saharan Africa | both | 1486.97 | 3436.13 | 858.01 | 2035.32 | 2.31 | 2.37 |
| Malawi | Eastern Sub-Saharan Africa | both | 1229.41 | 2299.21 | 708.39 | 1354.88 | 1.87 | 1.91 |
| Malaysia | Southeast Asia | both | 2086.40 | 3508.07 | 1121.90 | 1881.77 | 1.68 | 1.68 |
| Maldives | Southeast Asia | both | 25.54 | 77.05 | 14.29 | 41.19 | 3.02 | 2.88 |
| Mali | Western sub-Saharan Africa | both | 1279.27 | 2748.97 | 740.02 | 1578.98 | 2.15 | 2.13 |
| Malta | Western Europe | both | 502.25 | 482.97 | 407.00 | 366.39 | 0.96 | 0.90 |
| Marshall Islands | Oceania | both | 8.24 | 11.35 | 4.75 | 6.45 | 1.38 | 1.36 |
| Mauritania | Western sub-Saharan Africa | both | 328.81 | 584.21 | 194.15 | 346.02 | 1.78 | 1.78 |
| Mauritius | Southeast Asia | both | 159.54 | 156.30 | 86.66 | 84.97 | 0.98 | 0.98 |
| Mexico | Central Latin America | both | 10023.73 | 14579.59 | 5734.42 | 8330.18 | 1.45 | 1.45 |
| Republic of Moldova | Eastern Europe | both | 1667.72 | 1233.98 | 1077.49 | 774.25 | 0.74 | 0.72 |
| Mongolia | Central Asia | both | 829.63 | 1271.22 | 562.11 | 820.87 | 1.53 | 1.46 |
| Montenegro | Central Europe | both | 230.77 | 184.80 | 144.45 | 114.01 | 0.80 | 0.79 |
| Morocco | North Africa and Middle East | both | 9926.42 | 12850.82 | 6349.99 | 8080.72 | 1.29 | 1.27 |
| Mozambique | Eastern Sub-Saharan Africa | both | 1784.42 | 3613.44 | 1062.24 | 2171.95 | 2.02 | 2.04 |
| Myanmar | Southeast Asia | both | 6237.16 | 7870.31 | 3589.78 | 4486.21 | 1.26 | 1.25 |
| Namibia | Southern sub-Saharan Africa | both | 247.84 | 417.31 | 147.00 | 246.40 | 1.68 | 1.68 |
| Nepal | South Asia | both | 4120.73 | 5822.19 | 2509.23 | 3416.83 | 1.41 | 1.36 |
| Netherlands | Western Europe | both | 20771.80 | 18475.52 | 16634.16 | 14148.43 | 0.89 | 0.85 |
| New Zealand | Australasia | both | 3295.91 | 3456.73 | 3128.29 | 3165.89 | 1.05 | 1.01 |
| Nicaragua | Central Latin America | both | 435.28 | 792.36 | 245.97 | 448.87 | 1.82 | 1.82 |
| Niger | Western sub-Saharan Africa | both | 1399.17 | 3435.89 | 886.50 | 2259.31 | 2.46 | 2.55 |
| Nigeria | Western sub-Saharan Africa | both | 18235.66 | 33457.95 | 11948.48 | 20724.45 | 1.83 | 1.73 |
| Democratic People's Republic of Korea | East Asia | both | 8940.17 | 10256.60 | 6652.98 | 7472.03 | 1.15 | 1.12 |
| Northern Mariana Islands | Oceania | both | 10.22 | 6.86 | 5.51 | 3.85 | 0.67 | 0.70 |
| Norway | Western Europe | both | 4224.57 | 4680.49 | 3478.31 | 3780.28 | 1.11 | 1.09 |
| Oman | North Africa and Middle East | both | 698.15 | 1672.78 | 428.23 | 948.23 | 2.40 | 2.21 |
| Pakistan | South Asia | both | 24243.80 | 46496.83 | 15404.72 | 29380.20 | 1.92 | 1.91 |
| Palestine | North Africa and Middle East | both | 697.99 | 1539.98 | 444.43 | 925.69 | 2.21 | 2.08 |
| Panama | Central Latin America | both | 261.22 | 411.04 | 144.07 | 226.30 | 1.57 | 1.57 |
| Papua New Guinea | Oceania | both | 885.65 | 2089.48 | 519.58 | 1235.16 | 2.36 | 2.38 |
| Paraguay | Tropical Latin America | both | 1752.21 | 3346.55 | 1349.09 | 2608.74 | 1.91 | 1.93 |
| Peru | Andean Latin America | both | 9702.38 | 14882.61 | 7545.97 | 11186.04 | 1.53 | 1.48 |
| Philippines | Southeast Asia | both | 8036.83 | 14241.60 | 4439.36 | 7964.67 | 1.77 | 1.79 |
| Poland | Central Europe | both | 15242.02 | 13326.13 | 9929.62 | 8847.39 | 0.87 | 0.89 |
| Portugal | Western Europe | both | 13515.72 | 12174.19 | 11098.66 | 9633.03 | 0.90 | 0.87 |
| Puerto Rico | Caribbean | both | 1003.67 | 801.84 | 600.92 | 476.15 | 0.80 | 0.79 |
| Qatar | North Africa and Middle East | both | 194.12 | 1145.34 | 113.37 | 645.47 | 5.90 | 5.69 |
| Romania | Central Europe | both | 8455.45 | 5490.33 | 5352.57 | 3365.27 | 0.65 | 0.63 |
| Russian Federation | Eastern Europe | both | 52650.82 | 44739.28 | 32458.87 | 27559.90 | 0.85 | 0.85 |
| Rwanda | Eastern Sub-Saharan Africa | both | 755.81 | 1409.33 | 416.31 | 788.44 | 1.86 | 1.89 |
| Saint Lucia | Caribbean | both | 38.56 | 51.97 | 24.13 | 31.93 | 1.35 | 1.32 |
| Saint Vincent and the Grenadines | Caribbean | both | 31.54 | 32.27 | 20.01 | 20.03 | 1.02 | 1.00 |
| Samoa | Oceania | both | 28.58 | 37.87 | 16.26 | 21.87 | 1.32 | 1.35 |
| Sao Tome and Principe | Central sub-Saharan Africa | both | 16.17 | 29.41 | 9.18 | 16.72 | 1.82 | 1.82 |
| Saudi Arabia | North Africa and Middle East | both | 6114.69 | 13197.46 | 3858.32 | 7554.22 | 2.16 | 1.96 |
| Senegal | Western sub-Saharan Africa | both | 1205.47 | 2322.99 | 715.33 | 1401.57 | 1.93 | 1.96 |
| Serbia | Central Europe | both | 3602.90 | 2575.91 | 2327.33 | 1601.79 | 0.71 | 0.69 |
| Seychelles | Southeast Asia | both | 8.40 | 12.08 | 4.52 | 6.44 | 1.44 | 1.43 |
| Sierra Leone | Western sub-Saharan Africa | both | 615.08 | 1316.76 | 362.41 | 789.98 | 2.14 | 2.18 |
| Singapore | High-income Asia Pacific | both | 738.52 | 1186.32 | 403.98 | 645.87 | 1.61 | 1.60 |
| Slovakia | Central Europe | both | 2082.63 | 1700.74 | 1345.29 | 1051.06 | 0.82 | 0.78 |
| Slovenia | Central Europe | both | 749.00 | 568.49 | 468.47 | 345.99 | 0.76 | 0.74 |
| Solomon Islands | Oceania | both | 67.28 | 135.31 | 40.00 | 80.55 | 2.01 | 2.01 |
| Somalia | Eastern Sub-Saharan Africa | both | 985.70 | 2873.48 | 589.82 | 1838.01 | 2.92 | 3.12 |
| South Africa | Southern sub-Saharan Africa | both | 6963.19 | 10316.28 | 4088.84 | 6045.06 | 1.48 | 1.48 |
| Republic of Korea | High-income Asia Pacific | both | 12117.48 | 11949.40 | 6998.33 | 6730.71 | 0.99 | 0.96 |
| South Sudan | Eastern Sub-Saharan Africa | both | 718.96 | 1045.92 | 403.16 | 600.66 | 1.45 | 1.49 |
| Spain | Western Europe | both | 48154.02 | 50856.40 | 37919.67 | 39015.97 | 1.06 | 1.03 |
| Sri Lanka | Southeast Asia | both | 3895.57 | 4007.91 | 2507.22 | 2503.50 | 1.03 | 1.00 |
| Sudan | North Africa and Middle East | both | 7169.57 | 12550.76 | 4577.15 | 7577.21 | 1.75 | 1.66 |
| Suriname | Caribbean | both | 109.70 | 152.79 | 67.25 | 92.59 | 1.39 | 1.38 |
| Eswatini | Southern sub-Saharan Africa | both | 142.52 | 211.23 | 86.51 | 125.54 | 1.48 | 1.45 |
| Sweden | Western Europe | both | 8656.96 | 9082.35 | 5966.08 | 6084.10 | 1.05 | 1.02 |
| Switzerland | Western Europe | both | 8769.19 | 9453.60 | 6682.14 | 7034.38 | 1.08 | 1.05 |
| Syrian Arab Republic | North Africa and Middle East | both | 4238.41 | 4578.23 | 2671.98 | 2815.60 | 1.08 | 1.05 |
| Taiwan (Province of China) | East Asia | both | 7711.37 | 6933.40 | 5069.29 | 4475.68 | 0.90 | 0.88 |
| Tajikistan | Central Asia | both | 1909.53 | 3439.75 | 1290.06 | 2261.74 | 1.80 | 1.75 |
| United Republic of Tanzania | Eastern Sub-Saharan Africa | both | 3011.52 | 6383.35 | 1693.08 | 3682.98 | 2.12 | 2.18 |
| Thailand | Southeast Asia | both | 8052.36 | 8732.60 | 4384.32 | 4761.23 | 1.08 | 1.09 |
| Bahamas | Caribbean | both | 76.48 | 107.76 | 46.17 | 65.04 | 1.41 | 1.41 |
| Gambia | Western sub-Saharan Africa | both | 166.66 | 345.94 | 100.07 | 205.44 | 2.08 | 2.05 |
| Timor-Leste | Southeast Asia | both | 115.28 | 158.52 | 65.71 | 89.29 | 1.38 | 1.36 |
| Togo | Western sub-Saharan Africa | both | 559.31 | 1195.00 | 324.04 | 699.14 | 2.14 | 2.16 |
| Tonga | Oceania | both | 17.34 | 17.78 | 9.99 | 10.22 | 1.03 | 1.02 |
| Trinidad and Tobago | Caribbean | both | 332.18 | 353.90 | 199.08 | 209.31 | 1.07 | 1.05 |
| Tunisia | North Africa and Middle East | both | 3001.76 | 3774.81 | 1862.30 | 2297.01 | 1.26 | 1.23 |
| Turkey | North Africa and Middle East | both | 30621.27 | 38475.40 | 21919.91 | 26967.84 | 1.26 | 1.23 |
| Turkmenistan | Central Asia | both | 1306.68 | 1643.51 | 855.82 | 1024.25 | 1.26 | 1.20 |
| Uganda | Eastern Sub-Saharan Africa | both | 2026.43 | 4391.69 | 1140.31 | 2504.16 | 2.17 | 2.20 |
| Ukraine | Eastern Europe | both | 18476.97 | 13934.74 | 11592.26 | 8601.86 | 0.75 | 0.74 |
| United Arab Emirates | North Africa and Middle East | both | 759.71 | 3751.40 | 438.61 | 2167.86 | 4.94 | 4.94 |
| United Kingdom | Western Europe | both | 70117.04 | 70748.08 | 52766.54 | 50683.33 | 1.01 | 0.96 |
| United States of America | High-income North America | both | 145089.88 | 145305.33 | 150092.84 | 144570.40 | 1.00 | 0.96 |
| Uruguay | Southern Latin America | both | 1714.50 | 1824.07 | 1344.96 | 1347.77 | 1.06 | 1.00 |
| Uzbekistan | Central Asia | both | 7758.59 | 11999.81 | 5206.38 | 7694.69 | 1.55 | 1.48 |
| Vanuatu | Oceania | both | 31.01 | 57.78 | 18.16 | 33.93 | 1.86 | 1.87 |
| Venezuela (Bolivarian Republic of) | Central Latin America | both | 2092.94 | 3044.29 | 1152.29 | 1687.75 | 1.45 | 1.46 |
| Viet nam | Southeast Asia | both | 9075.45 | 13492.90 | 5138.23 | 7514.92 | 1.49 | 1.46 |
| United States Virgin Islands | Caribbean | both | 30.94 | 21.82 | 18.66 | 12.93 | 0.71 | 0.69 |
| Yemen | North Africa and Middle East | both | 4494.33 | 10117.12 | 2910.42 | 6263.89 | 2.25 | 2.15 |
| Zambia | Eastern Sub-Saharan Africa | both | 921.48 | 2102.11 | 519.64 | 1197.17 | 2.28 | 2.30 |
| Zimbabwe | Southern sub-Saharan Africa | both | 1758.58 | 2746.87 | 1051.86 | 1682.63 | 1.56 | 1.60 |
| Monaco | Western Europe | both | 34.22 | 33.88 | 26.70 | 25.30 | 0.99 | 0.95 |
| San Marino | Western Europe | both | 29.01 | 36.05 | 22.43 | 27.03 | 1.24 | 1.20 |
| Saint Kitts and Nevis | Caribbean | both | 10.95 | 16.67 | 6.70 | 10.05 | 1.52 | 1.50 |
| Cook Islands | Oceania | both | 3.42 | 2.74 | 1.89 | 1.52 | 0.80 | 0.80 |
| Nauru | Oceania | both | 1.78 | 1.79 | 0.98 | 0.99 | 1.01 | 1.01 |
| Niue | Oceania | both | 0.40 | 0.27 | 0.22 | 0.15 | 0.68 | 0.67 |
| Palau | Oceania | both | 2.98 | 3.24 | 1.62 | 1.76 | 1.09 | 1.08 |
| Tokelau | Oceania | both | 0.27 | 0.22 | 0.15 | 0.12 | 0.83 | 0.82 |
| Tuvalu | Oceania | both | 1.84 | 2.11 | 1.05 | 1.19 | 1.15 | 1.14 |
| Afghanistan | North Africa and Middle East | male | 1850.27 | 6772.90 | 1211.55 | 4359.64 | 3.66 | 3.60 |
| Albania | Central Europe | male | 717.36 | 446.91 | 470.68 | 282.84 | 0.62 | 0.60 |
| Algeria | North Africa and Middle East | male | 4004.54 | 6637.69 | 2442.98 | 3994.62 | 1.66 | 1.64 |
| American Samoa | Oceania | male | 4.47 | 4.56 | 2.37 | 2.42 | 1.02 | 1.02 |
| Andorra | Western Europe | male | 43.26 | 52.56 | 32.97 | 40.30 | 1.21 | 1.22 |
| Angola | Central sub-Saharan Africa | male | 1515.31 | 3199.26 | 1131.13 | 2291.31 | 2.11 | 2.03 |
| Antigua and Barbuda | Caribbean | male | 8.23 | 11.97 | 5.09 | 7.34 | 1.45 | 1.44 |
| Argentina | Southern Latin America | male | 9113.53 | 12756.05 | 6973.00 | 9202.15 | 1.40 | 1.32 |
| Armenia | Central Asia | male | 662.43 | 490.69 | 422.99 | 302.29 | 0.74 | 0.71 |
| Australia | Australasia | male | 7414.98 | 8875.58 | 6552.03 | 7467.01 | 1.20 | 1.14 |
| Austria | Western Europe | male | 5412.52 | 5262.18 | 4275.95 | 4040.40 | 0.97 | 0.94 |
| Azerbaijan | Central Asia | male | 1325.27 | 1891.07 | 832.29 | 1168.52 | 1.43 | 1.40 |
| Bahrain | North Africa and Middle East | male | 123.71 | 349.24 | 72.82 | 211.39 | 2.82 | 2.90 |
| Bangladesh | South Asia | male | 11925.85 | 16574.71 | 7547.12 | 10500.28 | 1.39 | 1.39 |
| Barbados | Caribbean | male | 34.46 | 34.89 | 20.92 | 21.27 | 1.01 | 1.02 |
| Belarus | Eastern Europe | male | 1993.52 | 1509.88 | 1277.04 | 931.67 | 0.76 | 0.73 |
| Belgium | Western Europe | male | 6825.41 | 6516.89 | 5479.37 | 5035.88 | 0.95 | 0.92 |
| Belize | Caribbean | male | 26.83 | 60.89 | 17.76 | 38.91 | 2.27 | 2.19 |
| Benin | Western sub-Saharan Africa | male | 323.95 | 847.83 | 193.04 | 514.26 | 2.62 | 2.66 |
| Bermuda | Caribbean | male | 9.78 | 7.63 | 6.06 | 4.72 | 0.78 | 0.78 |
| Bhutan | South Asia | male | 77.14 | 97.25 | 50.08 | 62.20 | 1.26 | 1.24 |
| Bolivia (Plurinational State of) | Andean Latin America | male | 1472.20 | 2737.32 | 1228.43 | 2214.26 | 1.86 | 1.80 |
| Bosnia and Herzegovina | Central Europe | male | 1058.92 | 541.47 | 704.55 | 342.74 | 0.51 | 0.49 |
| Botswana | Southern sub-Saharan Africa | male | 103.65 | 214.04 | 62.52 | 126.70 | 2.07 | 2.03 |
| Brazil | Tropical Latin America | male | 33405.37 | 49979.35 | 25394.70 | 38334.92 | 1.50 | 1.51 |
| Brunei Darussalam | High-income Asia Pacific | male | 31.12 | 55.73 | 16.70 | 29.68 | 1.79 | 1.78 |
| Bulgaria | Central Europe | male | 1695.78 | 1100.53 | 1086.87 | 686.08 | 0.65 | 0.63 |
| Burkina Faso | Western sub-Saharan Africa | male | 657.25 | 1597.81 | 401.50 | 1010.93 | 2.43 | 2.52 |
| Burundi | Eastern Sub-Saharan Africa | male | 305.69 | 685.65 | 173.98 | 404.25 | 2.24 | 2.32 |
| Cambodia | Southeast Asia | male | 600.38 | 1132.93 | 327.07 | 610.32 | 1.89 | 1.87 |
| Cameroon | Western sub-Saharan Africa | male | 699.34 | 1940.63 | 398.08 | 1113.22 | 2.77 | 2.80 |
| Canada | High-income North America | male | 7616.92 | 7709.55 | 7517.93 | 7379.82 | 1.01 | 0.98 |
| Cabo Verde | Western sub-Saharan Africa | male | 22.49 | 46.85 | 13.28 | 27.30 | 2.08 | 2.05 |
| Central African Republic | Central sub-Saharan Africa | male | 383.61 | 693.09 | 283.96 | 516.71 | 1.81 | 1.82 |
| Chad | Western sub-Saharan Africa | male | 456.09 | 1097.97 | 286.65 | 702.14 | 2.41 | 2.45 |
| Chile | Southern Latin America | male | 4001.79 | 5054.20 | 3028.69 | 3601.03 | 1.26 | 1.19 |
| China | East Asia | male | 259347.99 | 253085.52 | 165060.32 | 158020.59 | 0.98 | 0.96 |
| Colombia | Central Latin America | male | 1767.86 | 2383.57 | 986.49 | 1329.27 | 1.35 | 1.35 |
| Comoros | Eastern Sub-Saharan Africa | male | 25.24 | 45.14 | 14.39 | 26.23 | 1.79 | 1.82 |
| Congo | Central sub-Saharan Africa | male | 281.91 | 572.40 | 194.72 | 379.79 | 2.03 | 1.95 |
| Costa Rica | Central Latin America | male | 161.61 | 234.38 | 89.73 | 130.00 | 1.45 | 1.45 |
| Côte d'Ivoire | Western sub-Saharan Africa | male | 973.00 | 1998.68 | 563.15 | 1181.62 | 2.05 | 2.10 |
| Croatia | Central Europe | male | 985.84 | 646.24 | 623.62 | 398.84 | 0.66 | 0.64 |
| Cuba | Caribbean | male | 1767.83 | 1551.17 | 1111.36 | 980.42 | 0.88 | 0.88 |
| Cyprus | Western Europe | male | 537.39 | 831.05 | 425.87 | 615.26 | 1.55 | 1.44 |
| Czechia | Central Europe | male | 2133.90 | 1724.73 | 1366.60 | 1064.61 | 0.81 | 0.78 |
| Democratic Republic of the Congo | Central sub-Saharan Africa | male | 4792.50 | 10645.37 | 3448.96 | 7653.74 | 2.22 | 2.22 |
| Denmark | Western Europe | male | 3645.53 | 3313.84 | 2925.41 | 2535.66 | 0.91 | 0.87 |
| Djibouti | Eastern Sub-Saharan Africa | male | 32.78 | 79.84 | 18.75 | 46.31 | 2.44 | 2.47 |
| Dominica | Caribbean | male | 10.00 | 8.89 | 6.25 | 5.47 | 0.89 | 0.88 |
| Dominican Republic | Caribbean | male | 1087.03 | 1664.12 | 714.25 | 1069.54 | 1.53 | 1.50 |
| Ecuador | Andean Latin America | male | 2041.27 | 3423.40 | 1580.18 | 2528.38 | 1.68 | 1.60 |
| Egypt | North Africa and Middle East | male | 6213.14 | 9574.08 | 3324.00 | 5041.23 | 1.54 | 1.52 |
| El Salvador | Central Latin America | male | 283.45 | 317.72 | 158.84 | 180.21 | 1.12 | 1.13 |
| Equatorial Guinea | Central sub-Saharan Africa | male | 51.17 | 156.60 | 38.18 | 102.36 | 3.06 | 2.68 |
| Eritrea | Eastern Sub-Saharan Africa | male | 190.03 | 438.04 | 109.42 | 258.88 | 2.31 | 2.37 |
| Estonia | Eastern Europe | male | 292.55 | 200.20 | 184.16 | 121.57 | 0.68 | 0.66 |
| Ethiopia | Eastern Sub-Saharan Africa | male | 2945.27 | 6317.97 | 1733.36 | 3770.07 | 2.15 | 2.18 |
| Micronesia (Federated States of) | Oceania | male | 9.53 | 9.81 | 5.14 | 5.29 | 1.03 | 1.03 |
| Fiji | Oceania | male | 80.50 | 89.23 | 43.58 | 47.92 | 1.11 | 1.10 |
| Finland | Western Europe | male | 3849.18 | 3276.63 | 3226.32 | 2602.10 | 0.85 | 0.81 |
| France | Western Europe | male | 44363.76 | 40866.86 | 37874.42 | 33873.97 | 0.92 | 0.89 |
| Gabon | Central sub-Saharan Africa | male | 116.49 | 176.62 | 79.61 | 114.63 | 1.52 | 1.44 |
| Georgia | Central Asia | male | 974.58 | 574.52 | 609.32 | 357.34 | 0.59 | 0.59 |
| Germany | Western Europe | male | 45261.40 | 40460.41 | 32345.48 | 28123.09 | 0.89 | 0.87 |
| Ghana | Western sub-Saharan Africa | male | 1268.31 | 2592.98 | 782.76 | 1618.78 | 2.04 | 2.07 |
| Greece | Western Europe | male | 6689.85 | 5781.79 | 5359.24 | 4530.46 | 0.86 | 0.85 |
| Greenland | High-income North America | male | 23.41 | 15.44 | 23.29 | 15.18 | 0.66 | 0.65 |
| Grenada | Caribbean | male | 11.43 | 14.73 | 7.31 | 9.17 | 1.29 | 1.25 |
| Guam | Oceania | male | 14.41 | 13.41 | 7.48 | 7.06 | 0.93 | 0.94 |
| Guatemala | Central Latin America | male | 439.73 | 998.46 | 252.12 | 574.33 | 2.27 | 2.28 |
| Guinea | Western sub-Saharan Africa | male | 452.20 | 829.00 | 275.17 | 513.12 | 1.83 | 1.86 |
| Guinea-Bissau | Western sub-Saharan Africa | male | 75.02 | 140.89 | 45.24 | 86.49 | 1.88 | 1.91 |
| Guyana | Caribbean | male | 110.62 | 105.05 | 68.80 | 64.68 | 0.95 | 0.94 |
| Haiti | Caribbean | male | 972.84 | 1990.83 | 659.40 | 1338.82 | 2.05 | 2.03 |
| Honduras | Central Latin America | male | 254.73 | 552.91 | 146.71 | 319.65 | 2.17 | 2.18 |
| Hungary | Central Europe | male | 2089.83 | 1573.89 | 1343.94 | 984.22 | 0.75 | 0.73 |
| Iceland | Western Europe | male | 188.80 | 225.29 | 154.44 | 176.81 | 1.19 | 1.14 |
| India | South Asia | male | 107064.61 | 181984.40 | 68282.28 | 122956.63 | 1.70 | 1.80 |
| Indonesia | Southeast Asia | male | 6265.60 | 9611.13 | 3172.51 | 4896.04 | 1.53 | 1.54 |
| Iran (Islamic Republic of) | North Africa and Middle East | male | 9538.41 | 14544.16 | 5847.28 | 8745.76 | 1.52 | 1.50 |
| Iraq | North Africa and Middle East | male | 3217.64 | 7639.97 | 2089.04 | 4713.92 | 2.37 | 2.26 |
| Ireland | Western Europe | male | 2402.55 | 2965.38 | 1940.14 | 2307.29 | 1.23 | 1.19 |
| Israel | Western Europe | male | 2917.02 | 5190.20 | 2242.05 | 3839.95 | 1.78 | 1.71 |
| Italy | Western Europe | male | 30045.89 | 26184.05 | 26621.22 | 23944.55 | 0.87 | 0.90 |
| Jamaica | Caribbean | male | 324.38 | 399.20 | 207.03 | 249.30 | 1.23 | 1.20 |
| Japan | High-income Asia Pacific | male | 14686.38 | 12550.07 | 8345.36 | 7148.72 | 0.85 | 0.86 |
| Jordan | North Africa and Middle East | male | 597.94 | 1877.23 | 355.09 | 1104.64 | 3.14 | 3.11 |
| Kazakhstan | Central Asia | male | 3246.91 | 3169.59 | 2077.50 | 1960.99 | 0.98 | 0.94 |
| Kenya | Eastern Sub-Saharan Africa | male | 1134.69 | 2744.27 | 642.99 | 1587.65 | 2.42 | 2.47 |
| Kiribati | Oceania | male | 7.07 | 11.19 | 3.78 | 6.10 | 1.58 | 1.61 |
| Kuwait | North Africa and Middle East | male | 383.30 | 819.10 | 220.19 | 475.66 | 2.14 | 2.16 |
| Kyrgyzstan | Central Asia | male | 829.91 | 1204.14 | 537.75 | 770.14 | 1.45 | 1.43 |
| Lao People's Democratic Republic | Southeast Asia | male | 270.86 | 518.65 | 148.58 | 281.18 | 1.91 | 1.89 |
| Latvia | Eastern Europe | male | 486.78 | 276.15 | 304.28 | 168.23 | 0.57 | 0.55 |
| Lebanon | North Africa and Middle East | male | 509.24 | 752.58 | 310.83 | 443.21 | 1.48 | 1.43 |
| Lesotho | Southern sub-Saharan Africa | male | 172.02 | 217.94 | 109.25 | 134.97 | 1.27 | 1.24 |
| Liberia | Western sub-Saharan Africa | male | 135.85 | 377.72 | 79.07 | 224.55 | 2.78 | 2.84 |
| Libya | North Africa and Middle East | male | 685.12 | 1168.10 | 412.97 | 681.13 | 1.70 | 1.65 |
| Lithuania | Eastern Europe | male | 683.59 | 391.25 | 427.06 | 235.77 | 0.57 | 0.55 |
| Luxembourg | Western Europe | male | 263.82 | 394.69 | 206.24 | 301.13 | 1.50 | 1.46 |
| North Macedonia | Central Europe | male | 424.03 | 386.74 | 272.26 | 241.83 | 0.91 | 0.89 |
| Madagascar | Eastern Sub-Saharan Africa | male | 709.09 | 1653.36 | 410.31 | 989.61 | 2.33 | 2.41 |
| Malawi | Eastern Sub-Saharan Africa | male | 577.44 | 1079.32 | 333.89 | 640.58 | 1.87 | 1.92 |
| Malaysia | Southeast Asia | male | 1019.35 | 1805.49 | 524.94 | 925.23 | 1.77 | 1.76 |
| Maldives | Southeast Asia | male | 12.26 | 49.56 | 6.56 | 25.53 | 4.04 | 3.89 |
| Mali | Western sub-Saharan Africa | male | 585.80 | 1292.21 | 343.63 | 754.78 | 2.21 | 2.20 |
| Malta | Western Europe | male | 259.08 | 256.15 | 210.16 | 194.58 | 0.99 | 0.93 |
| Marshall Islands | Oceania | male | 4.24 | 5.95 | 2.33 | 3.22 | 1.40 | 1.38 |
| Mauritania | Western sub-Saharan Africa | male | 154.63 | 273.74 | 91.79 | 163.15 | 1.77 | 1.78 |
| Mauritius | Southeast Asia | male | 78.44 | 78.63 | 40.30 | 40.68 | 1.00 | 1.01 |
| Mexico | Central Latin America | male | 4738.37 | 6921.19 | 2737.64 | 3992.98 | 1.46 | 1.46 |
| Republic of Moldova | Eastern Europe | male | 826.45 | 643.31 | 523.54 | 399.43 | 0.78 | 0.76 |
| Mongolia | Central Asia | male | 424.68 | 665.19 | 280.39 | 422.18 | 1.57 | 1.51 |
| Montenegro | Central Europe | male | 122.20 | 98.91 | 75.89 | 60.68 | 0.81 | 0.80 |
| Morocco | North Africa and Middle East | male | 4688.64 | 6197.77 | 2990.94 | 3895.20 | 1.32 | 1.30 |
| Mozambique | Eastern Sub-Saharan Africa | male | 781.57 | 1638.18 | 466.89 | 994.86 | 2.10 | 2.13 |
| Myanmar | Southeast Asia | male | 2972.07 | 3714.33 | 1620.98 | 1998.78 | 1.25 | 1.23 |
| Namibia | Southern sub-Saharan Africa | male | 116.96 | 198.34 | 70.00 | 118.46 | 1.70 | 1.69 |
| Nepal | South Asia | male | 1970.73 | 2572.48 | 1215.22 | 1526.52 | 1.31 | 1.26 |
| Netherlands | Western Europe | male | 10881.97 | 9524.11 | 8675.54 | 7298.01 | 0.88 | 0.84 |
| New Zealand | Australasia | male | 1610.82 | 1638.74 | 1435.93 | 1412.25 | 1.02 | 0.98 |
| Nicaragua | Central Latin America | male | 203.16 | 381.79 | 115.48 | 217.78 | 1.88 | 1.89 |
| Niger | Western sub-Saharan Africa | male | 655.86 | 1633.83 | 419.97 | 1090.92 | 2.49 | 2.60 |
| Nigeria | Western sub-Saharan Africa | male | 9168.27 | 15380.31 | 6052.73 | 9651.57 | 1.68 | 1.59 |
| Democratic People's Republic of Korea | East Asia | male | 4717.91 | 5974.32 | 3309.63 | 4133.22 | 1.27 | 1.25 |
| Northern Mariana Islands | Oceania | male | 5.65 | 3.81 | 2.93 | 2.06 | 0.68 | 0.70 |
| Norway | Western Europe | male | 2170.21 | 2412.50 | 1765.81 | 1933.77 | 1.11 | 1.10 |
| Oman | North Africa and Middle East | male | 446.54 | 1143.27 | 273.11 | 646.91 | 2.56 | 2.37 |
| Pakistan | South Asia | male | 12415.22 | 22967.52 | 7978.48 | 14694.65 | 1.85 | 1.84 |
| Palestine | North Africa and Middle East | male | 333.78 | 751.27 | 209.51 | 445.88 | 2.25 | 2.13 |
| Panama | Central Latin America | male | 128.29 | 202.62 | 71.65 | 112.48 | 1.58 | 1.57 |
| Papua New Guinea | Oceania | male | 459.32 | 1075.94 | 257.26 | 604.07 | 2.34 | 2.35 |
| Paraguay | Tropical Latin America | male | 861.06 | 1659.25 | 674.80 | 1313.89 | 1.93 | 1.95 |
| Peru | Andean Latin America | male | 4647.96 | 7192.40 | 3673.63 | 5492.38 | 1.55 | 1.50 |
| Philippines | Southeast Asia | male | 3890.52 | 7126.53 | 2048.91 | 3796.95 | 1.83 | 1.85 |
| Poland | Central Europe | male | 7867.69 | 7040.65 | 5049.40 | 4564.55 | 0.89 | 0.90 |
| Portugal | Western Europe | male | 6799.92 | 6124.38 | 5537.36 | 4857.60 | 0.90 | 0.88 |
| Puerto Rico | Caribbean | male | 467.21 | 377.65 | 283.02 | 226.71 | 0.81 | 0.80 |
| Qatar | North Africa and Middle East | male | 140.23 | 885.75 | 81.89 | 498.36 | 6.32 | 6.09 |
| Romania | Central Europe | male | 4494.49 | 3042.02 | 2849.62 | 1887.03 | 0.68 | 0.66 |
| Russian Federation | Eastern Europe | male | 26912.95 | 22925.60 | 16216.38 | 13912.48 | 0.85 | 0.86 |
| Rwanda | Eastern Sub-Saharan Africa | male | 354.13 | 664.16 | 196.91 | 376.31 | 1.88 | 1.91 |
| Saint Lucia | Caribbean | male | 18.32 | 25.32 | 11.60 | 15.77 | 1.38 | 1.36 |
| Saint Vincent and the Grenadines | Caribbean | male | 15.58 | 15.98 | 9.99 | 10.08 | 1.03 | 1.01 |
| Samoa | Oceania | male | 14.85 | 19.77 | 8.10 | 10.92 | 1.33 | 1.35 |
| Sao Tome and Principe | Central sub-Saharan Africa | male | 7.60 | 14.59 | 4.36 | 8.39 | 1.92 | 1.92 |
| Saudi Arabia | North Africa and Middle East | male | 3546.30 | 7678.73 | 2230.56 | 4405.27 | 2.17 | 1.97 |
| Senegal | Western sub-Saharan Africa | male | 553.00 | 1118.44 | 331.87 | 684.92 | 2.02 | 2.06 |
| Serbia | Central Europe | male | 1901.52 | 1394.29 | 1231.23 | 862.90 | 0.73 | 0.70 |
| Seychelles | Southeast Asia | male | 4.17 | 6.67 | 2.15 | 3.40 | 1.60 | 1.59 |
| Sierra Leone | Western sub-Saharan Africa | male | 282.51 | 632.10 | 167.31 | 384.77 | 2.24 | 2.30 |
| Singapore | High-income Asia Pacific | male | 373.43 | 639.34 | 200.07 | 343.75 | 1.71 | 1.72 |
| Slovakia | Central Europe | male | 1090.51 | 930.23 | 701.48 | 574.12 | 0.85 | 0.82 |
| Slovenia | Central Europe | male | 399.41 | 315.78 | 248.26 | 192.64 | 0.79 | 0.78 |
| Solomon Islands | Oceania | male | 34.20 | 68.73 | 19.41 | 38.99 | 2.01 | 2.01 |
| Somalia | Eastern Sub-Saharan Africa | male | 480.40 | 1441.96 | 291.81 | 926.65 | 3.00 | 3.18 |
| South Africa | Southern sub-Saharan Africa | male | 3275.86 | 5049.39 | 1941.71 | 3007.59 | 1.54 | 1.55 |
| Republic of Korea | High-income Asia Pacific | male | 6161.32 | 6421.34 | 3472.30 | 3589.92 | 1.04 | 1.03 |
| South Sudan | Eastern Sub-Saharan Africa | male | 369.67 | 481.08 | 208.86 | 278.12 | 1.30 | 1.33 |
| Spain | Western Europe | male | 24171.97 | 25785.81 | 18612.84 | 19771.81 | 1.07 | 1.06 |
| Sri Lanka | Southeast Asia | male | 1924.49 | 1966.93 | 1167.97 | 1145.97 | 1.02 | 0.98 |
| Sudan | North Africa and Middle East | male | 3325.25 | 5871.36 | 2106.43 | 3507.98 | 1.77 | 1.67 |
| Suriname | Caribbean | male | 54.08 | 73.58 | 33.67 | 45.08 | 1.36 | 1.34 |
| Eswatini | Southern sub-Saharan Africa | male | 62.46 | 100.13 | 38.27 | 60.15 | 1.60 | 1.57 |
| Sweden | Western Europe | male | 4413.50 | 4663.34 | 3036.93 | 3094.98 | 1.06 | 1.02 |
| Switzerland | Western Europe | male | 4621.38 | 4955.74 | 3513.42 | 3698.01 | 1.07 | 1.05 |
| Syrian Arab Republic | North Africa and Middle East | male | 2054.09 | 1997.79 | 1287.58 | 1233.63 | 0.97 | 0.96 |
| Taiwan (Province of China) | East Asia | male | 4341.07 | 3946.40 | 2759.98 | 2476.45 | 0.91 | 0.90 |
| Tajikistan | Central Asia | male | 975.60 | 1810.00 | 641.15 | 1159.03 | 1.86 | 1.81 |
| United Republic of Tanzania | Eastern Sub-Saharan Africa | male | 1369.23 | 2924.23 | 778.65 | 1707.06 | 2.14 | 2.19 |
| Thailand | Southeast Asia | male | 3855.13 | 4359.52 | 2002.46 | 2260.97 | 1.13 | 1.13 |
| Bahamas | Caribbean | male | 36.45 | 51.03 | 22.28 | 31.29 | 1.40 | 1.40 |
| Gambia | Western sub-Saharan Africa | male | 79.53 | 163.33 | 48.35 | 98.48 | 2.05 | 2.04 |
| Timor-Leste | Southeast Asia | male | 57.20 | 76.06 | 30.83 | 40.65 | 1.33 | 1.32 |
| Togo | Western sub-Saharan Africa | male | 252.69 | 557.55 | 147.74 | 331.17 | 2.21 | 2.24 |
| Tonga | Oceania | male | 8.41 | 8.74 | 4.64 | 4.79 | 1.04 | 1.03 |
| Trinidad and Tobago | Caribbean | male | 162.79 | 175.22 | 99.09 | 104.94 | 1.08 | 1.06 |
| Tunisia | North Africa and Middle East | male | 1433.65 | 1784.31 | 884.89 | 1086.09 | 1.24 | 1.23 |
| Turkey | North Africa and Middle East | male | 14362.20 | 18710.61 | 10031.67 | 12754.31 | 1.30 | 1.27 |
| Turkmenistan | Central Asia | male | 662.82 | 905.98 | 422.74 | 554.89 | 1.37 | 1.31 |
| Uganda | Eastern Sub-Saharan Africa | male | 948.54 | 2041.40 | 537.39 | 1181.72 | 2.15 | 2.20 |
| Ukraine | Eastern Europe | male | 9234.34 | 7095.59 | 5696.55 | 4332.07 | 0.77 | 0.76 |
| United Arab Emirates | North Africa and Middle East | male | 537.32 | 2811.06 | 311.31 | 1636.24 | 5.23 | 5.26 |
| United Kingdom | Western Europe | male | 39011.30 | 39872.37 | 30457.05 | 29582.12 | 1.02 | 0.97 |
| United States of America | High-income North America | male | 75029.89 | 74126.45 | 72943.94 | 70304.12 | 0.99 | 0.96 |
| Uruguay | Southern Latin America | male | 841.12 | 895.13 | 641.84 | 647.05 | 1.06 | 1.01 |
| Uzbekistan | Central Asia | male | 3958.41 | 6269.15 | 2601.90 | 3937.51 | 1.58 | 1.51 |
| Vanuatu | Oceania | male | 15.71 | 28.97 | 8.76 | 16.24 | 1.84 | 1.85 |
| Venezuela (Bolivarian Republic of) | Central Latin America | male | 1009.80 | 1453.24 | 559.62 | 813.89 | 1.44 | 1.45 |
| Viet nam | Southeast Asia | male | 4123.80 | 6779.34 | 2214.61 | 3589.29 | 1.64 | 1.62 |
| United States Virgin Islands | Caribbean | male | 14.30 | 10.08 | 8.74 | 6.05 | 0.70 | 0.69 |
| Yemen | North Africa and Middle East | male | 2132.44 | 4881.81 | 1378.49 | 3014.25 | 2.29 | 2.19 |
| Zambia | Eastern Sub-Saharan Africa | male | 428.43 | 1007.44 | 244.40 | 577.74 | 2.35 | 2.36 |
| Zimbabwe | Southern sub-Saharan Africa | male | 818.11 | 1274.40 | 495.31 | 788.36 | 1.56 | 1.59 |
| Monaco | Western Europe | male | 17.32 | 16.99 | 13.59 | 12.77 | 0.98 | 0.94 |
| San Marino | Western Europe | male | 15.11 | 17.36 | 11.67 | 13.02 | 1.15 | 1.12 |
| Saint Kitts and Nevis | Caribbean | male | 5.31 | 8.21 | 3.28 | 5.00 | 1.55 | 1.52 |
| Cook Islands | Oceania | male | 1.79 | 1.33 | 0.95 | 0.71 | 0.75 | 0.74 |
| Nauru | Oceania | male | 0.92 | 0.91 | 0.49 | 0.48 | 0.99 | 0.98 |
| Niue | Oceania | male | 0.21 | 0.14 | 0.11 | 0.08 | 0.68 | 0.68 |
| Palau | Oceania | male | 1.56 | 1.97 | 0.81 | 1.03 | 1.26 | 1.26 |
| Tokelau | Oceania | male | 0.13 | 0.12 | 0.07 | 0.06 | 0.89 | 0.88 |
| Tuvalu | Oceania | male | 0.85 | 1.14 | 0.46 | 0.62 | 1.35 | 1.35 |
| Afghanistan | North Africa and Middle East | female | 2366.06 | 6736.95 | 1587.42 | 4481.31 | 2.85 | 2.82 |
| Albania | Central Europe | female | 623.33 | 392.79 | 424.80 | 254.77 | 0.63 | 0.60 |
| Algeria | North Africa and Middle East | female | 4299.00 | 7080.27 | 2641.57 | 4291.60 | 1.65 | 1.62 |
| American Samoa | Oceania | female | 4.26 | 4.40 | 2.46 | 2.55 | 1.03 | 1.04 |
| Andorra | Western Europe | female | 34.79 | 47.09 | 26.77 | 35.24 | 1.35 | 1.32 |
| Angola | Central sub-Saharan Africa | female | 1544.26 | 3690.66 | 1138.19 | 2580.08 | 2.39 | 2.27 |
| Antigua and Barbuda | Caribbean | female | 9.34 | 13.42 | 5.62 | 8.06 | 1.44 | 1.44 |
| Argentina | Southern Latin America | female | 9428.36 | 13037.95 | 7646.63 | 9901.12 | 1.38 | 1.29 |
| Armenia | Central Asia | female | 644.79 | 458.63 | 430.13 | 290.90 | 0.71 | 0.68 |
| Australia | Australasia | female | 7527.24 | 9245.10 | 7550.48 | 8742.43 | 1.23 | 1.16 |
| Austria | Western Europe | female | 4976.51 | 4807.77 | 4006.38 | 3702.54 | 0.97 | 0.92 |
| Azerbaijan | Central Asia | female | 1317.34 | 1689.26 | 871.52 | 1079.17 | 1.28 | 1.24 |
| Bahrain | North Africa and Middle East | female | 78.81 | 199.06 | 46.12 | 116.71 | 2.53 | 2.53 |
| Bangladesh | South Asia | female | 11802.75 | 18783.13 | 7239.62 | 11527.34 | 1.59 | 1.59 |
| Barbados | Caribbean | female | 37.77 | 38.69 | 22.42 | 23.13 | 1.02 | 1.03 |
| Belarus | Eastern Europe | female | 1941.05 | 1426.79 | 1298.95 | 901.97 | 0.74 | 0.69 |
| Belgium | Western Europe | female | 6236.09 | 6077.07 | 5079.61 | 4702.57 | 0.97 | 0.93 |
| Belize | Caribbean | female | 27.41 | 66.52 | 17.65 | 41.71 | 2.43 | 2.36 |
| Benin | Western sub-Saharan Africa | female | 415.12 | 954.65 | 242.81 | 565.17 | 2.30 | 2.33 |
| Bermuda | Caribbean | female | 10.56 | 8.09 | 6.36 | 4.87 | 0.77 | 0.77 |
| Bhutan | South Asia | female | 69.02 | 89.47 | 43.24 | 56.12 | 1.30 | 1.30 |
| Bolivia (Plurinational State of) | Andean Latin America | female | 1626.57 | 2883.91 | 1319.62 | 2258.15 | 1.77 | 1.71 |
| Bosnia and Herzegovina | Central Europe | female | 919.79 | 468.31 | 628.50 | 299.96 | 0.51 | 0.48 |
| Botswana | Southern sub-Saharan Africa | female | 125.60 | 227.14 | 74.10 | 130.63 | 1.81 | 1.76 |
| Brazil | Tropical Latin America | female | 36366.18 | 54339.36 | 26743.23 | 40368.59 | 1.49 | 1.51 |
| Brunei Darussalam | High-income Asia Pacific | female | 26.52 | 46.58 | 14.78 | 25.95 | 1.76 | 1.76 |
| Bulgaria | Central Europe | female | 1551.19 | 903.58 | 994.54 | 559.92 | 0.58 | 0.56 |
| Burkina Faso | Western sub-Saharan Africa | female | 861.76 | 1885.37 | 522.53 | 1165.87 | 2.19 | 2.23 |
| Burundi | Eastern Sub-Saharan Africa | female | 352.01 | 739.71 | 196.85 | 433.41 | 2.10 | 2.20 |
| Cambodia | Southeast Asia | female | 787.18 | 1208.97 | 468.94 | 721.42 | 1.54 | 1.54 |
| Cameroon | Western sub-Saharan Africa | female | 815.64 | 2081.46 | 459.37 | 1164.21 | 2.55 | 2.53 |
| Canada | High-income North America | female | 7179.60 | 7456.63 | 7972.08 | 7863.18 | 1.04 | 0.99 |
| Cabo Verde | Western sub-Saharan Africa | female | 28.65 | 44.57 | 16.67 | 25.47 | 1.56 | 1.53 |
| Central African Republic | Central sub-Saharan Africa | female | 429.17 | 773.38 | 312.51 | 564.45 | 1.80 | 1.81 |
| Chad | Western sub-Saharan Africa | female | 556.44 | 1250.19 | 344.40 | 784.02 | 2.25 | 2.28 |
| Chile | Southern Latin America | female | 4203.81 | 5040.70 | 3387.11 | 3772.21 | 1.20 | 1.11 |
| China | East Asia | female | 217018.43 | 198204.80 | 157344.14 | 141155.45 | 0.91 | 0.90 |
| Colombia | Central Latin America | female | 1955.43 | 2590.45 | 1075.01 | 1424.34 | 1.32 | 1.32 |
| Comoros | Eastern Sub-Saharan Africa | female | 28.67 | 46.81 | 16.11 | 26.78 | 1.63 | 1.66 |
| Congo | Central sub-Saharan Africa | female | 308.94 | 614.20 | 207.34 | 397.61 | 1.99 | 1.92 |
| Costa Rica | Central Latin America | female | 172.78 | 263.88 | 95.12 | 144.07 | 1.53 | 1.51 |
| Côte d'Ivoire | Western sub-Saharan Africa | female | 982.55 | 1945.46 | 560.13 | 1126.07 | 1.98 | 2.01 |
| Croatia | Central Europe | female | 871.58 | 546.28 | 551.74 | 339.76 | 0.63 | 0.62 |
| Cuba | Caribbean | female | 1849.77 | 1583.63 | 1126.39 | 976.00 | 0.86 | 0.87 |
| Cyprus | Western Europe | female | 492.96 | 806.83 | 396.18 | 604.66 | 1.64 | 1.53 |
| Czechia | Central Europe | female | 1925.15 | 1418.97 | 1228.32 | 871.93 | 0.74 | 0.71 |
| Democratic Republic of the Congo | Central sub-Saharan Africa | female | 5274.57 | 10909.05 | 3697.41 | 7659.39 | 2.07 | 2.07 |
| Denmark | Western Europe | female | 3322.32 | 3048.48 | 2678.69 | 2346.22 | 0.92 | 0.88 |
| Djibouti | Eastern Sub-Saharan Africa | female | 28.82 | 78.64 | 16.28 | 44.94 | 2.73 | 2.76 |
| Dominica | Caribbean | female | 9.77 | 8.90 | 5.98 | 5.32 | 0.91 | 0.89 |
| Dominican Republic | Caribbean | female | 1232.68 | 1704.92 | 793.25 | 1071.09 | 1.38 | 1.35 |
| Ecuador | Andean Latin America | female | 2223.12 | 3656.57 | 1670.25 | 2632.34 | 1.64 | 1.58 |
| Egypt | North Africa and Middle East | female | 6107.51 | 9848.40 | 3210.51 | 5098.52 | 1.61 | 1.59 |
| El Salvador | Central Latin America | female | 328.54 | 394.96 | 183.44 | 219.19 | 1.20 | 1.19 |
| Equatorial Guinea | Central sub-Saharan Africa | female | 65.97 | 138.48 | 47.97 | 87.33 | 2.10 | 1.82 |
| Eritrea | Eastern Sub-Saharan Africa | female | 205.10 | 445.39 | 118.42 | 260.46 | 2.17 | 2.20 |
| Estonia | Eastern Europe | female | 287.47 | 179.42 | 187.56 | 111.17 | 0.62 | 0.59 |
| Ethiopia | Eastern Sub-Saharan Africa | female | 3339.67 | 6764.90 | 1937.20 | 3958.28 | 2.03 | 2.04 |
| Micronesia (Federated States of) | Oceania | female | 8.96 | 9.03 | 5.32 | 5.36 | 1.01 | 1.01 |
| Fiji | Oceania | female | 77.19 | 80.08 | 45.65 | 47.60 | 1.04 | 1.04 |
| Finland | Western Europe | female | 3472.37 | 2922.55 | 2929.95 | 2340.89 | 0.84 | 0.80 |
| France | Western Europe | female | 42865.01 | 40235.76 | 38129.11 | 34053.55 | 0.94 | 0.89 |
| Gabon | Central sub-Saharan Africa | female | 116.68 | 203.01 | 77.39 | 127.94 | 1.74 | 1.65 |
| Georgia | Central Asia | female | 969.33 | 513.68 | 625.59 | 324.68 | 0.53 | 0.52 |
| Germany | Western Europe | female | 40397.95 | 35336.58 | 28550.86 | 24132.80 | 0.87 | 0.85 |
| Ghana | Western sub-Saharan Africa | female | 1442.46 | 2966.06 | 879.25 | 1794.89 | 2.06 | 2.04 |
| Greece | Western Europe | female | 6393.34 | 5489.94 | 5160.67 | 4218.42 | 0.86 | 0.82 |
| Greenland | High-income North America | female | 16.81 | 13.30 | 18.99 | 14.66 | 0.79 | 0.77 |
| Grenada | Caribbean | female | 11.98 | 14.46 | 7.53 | 8.77 | 1.21 | 1.17 |
| Guam | Oceania | female | 11.63 | 11.60 | 6.60 | 6.58 | 1.00 | 1.00 |
| Guatemala | Central Latin America | female | 491.92 | 1154.60 | 278.39 | 651.20 | 2.35 | 2.34 |
| Guinea | Western sub-Saharan Africa | female | 561.44 | 1028.54 | 335.37 | 617.62 | 1.83 | 1.84 |
| Guinea-Bissau | Western sub-Saharan Africa | female | 93.01 | 163.22 | 55.72 | 96.82 | 1.75 | 1.74 |
| Guyana | Caribbean | female | 119.37 | 113.24 | 72.40 | 67.88 | 0.95 | 0.94 |
| Haiti | Caribbean | female | 1122.76 | 2319.45 | 739.23 | 1522.26 | 2.07 | 2.06 |
| Honduras | Central Latin America | female | 282.47 | 643.86 | 160.73 | 365.07 | 2.28 | 2.27 |
| Hungary | Central Europe | female | 1917.28 | 1339.49 | 1224.62 | 835.90 | 0.70 | 0.68 |
| Iceland | Western Europe | female | 171.88 | 199.68 | 144.07 | 159.53 | 1.16 | 1.11 |
| India | South Asia | female | 102616.43 | 178464.08 | 63772.05 | 117090.08 | 1.74 | 1.84 |
| Indonesia | Southeast Asia | female | 7062.57 | 9825.46 | 4029.20 | 5655.74 | 1.39 | 1.40 |
| Iran (Islamic Republic of) | North Africa and Middle East | female | 10190.15 | 15021.18 | 6344.46 | 9051.77 | 1.47 | 1.43 |
| Iraq | North Africa and Middle East | female | 3278.79 | 7530.68 | 2151.03 | 4714.18 | 2.30 | 2.19 |
| Ireland | Western Europe | female | 2275.31 | 2892.12 | 1861.17 | 2234.44 | 1.27 | 1.20 |
| Israel | Western Europe | female | 2874.07 | 4906.95 | 2234.51 | 3642.69 | 1.71 | 1.63 |
| Italy | Western Europe | female | 27863.56 | 23682.47 | 26810.73 | 23069.07 | 0.85 | 0.86 |
| Jamaica | Caribbean | female | 356.80 | 431.11 | 222.88 | 263.32 | 1.21 | 1.18 |
| Japan | High-income Asia Pacific | female | 14456.91 | 11511.37 | 8388.25 | 6675.57 | 0.80 | 0.80 |
| Jordan | North Africa and Middle East | female | 582.58 | 1690.75 | 352.46 | 994.28 | 2.90 | 2.82 |
| Kazakhstan | Central Asia | female | 3096.33 | 2950.32 | 2050.04 | 1886.43 | 0.95 | 0.92 |
| Kenya | Eastern Sub-Saharan Africa | female | 1263.55 | 2962.59 | 708.05 | 1675.03 | 2.34 | 2.37 |
| Kiribati | Oceania | female | 7.31 | 11.66 | 4.31 | 7.07 | 1.59 | 1.64 |
| Kuwait | North Africa and Middle East | female | 267.25 | 779.96 | 152.55 | 446.03 | 2.92 | 2.92 |
| Kyrgyzstan | Central Asia | female | 793.77 | 1108.77 | 536.84 | 739.44 | 1.40 | 1.38 |
| Lao People's Democratic Republic | Southeast Asia | female | 313.25 | 537.87 | 190.42 | 325.48 | 1.72 | 1.71 |
| Latvia | Eastern Europe | female | 484.40 | 258.56 | 312.81 | 159.36 | 0.53 | 0.51 |
| Lebanon | North Africa and Middle East | female | 587.06 | 845.32 | 362.57 | 504.05 | 1.44 | 1.39 |
| Lesotho | Southern sub-Saharan Africa | female | 191.31 | 219.91 | 119.10 | 132.64 | 1.15 | 1.11 |
| Liberia | Western sub-Saharan Africa | female | 161.58 | 394.34 | 91.87 | 228.39 | 2.44 | 2.49 |
| Libya | North Africa and Middle East | female | 615.09 | 1169.57 | 370.70 | 679.49 | 1.90 | 1.83 |
| Lithuania | Eastern Europe | female | 678.88 | 372.82 | 439.65 | 229.69 | 0.55 | 0.52 |
| Luxembourg | Western Europe | female | 237.87 | 359.28 | 187.23 | 272.78 | 1.51 | 1.46 |
| North Macedonia | Central Europe | female | 377.72 | 313.81 | 246.80 | 197.40 | 0.83 | 0.80 |
| Madagascar | Eastern Sub-Saharan Africa | female | 777.88 | 1782.78 | 447.70 | 1045.70 | 2.29 | 2.34 |
| Malawi | Eastern Sub-Saharan Africa | female | 651.97 | 1219.90 | 374.50 | 714.30 | 1.87 | 1.91 |
| Malaysia | Southeast Asia | female | 1067.05 | 1702.58 | 596.96 | 956.53 | 1.60 | 1.60 |
| Maldives | Southeast Asia | female | 13.29 | 27.48 | 7.73 | 15.65 | 2.07 | 2.03 |
| Mali | Western sub-Saharan Africa | female | 693.46 | 1456.76 | 396.40 | 824.20 | 2.10 | 2.08 |
| Malta | Western Europe | female | 243.17 | 226.82 | 196.84 | 171.81 | 0.93 | 0.87 |
| Marshall Islands | Oceania | female | 4.00 | 5.40 | 2.42 | 3.23 | 1.35 | 1.34 |
| Mauritania | Western sub-Saharan Africa | female | 174.18 | 310.47 | 102.36 | 182.86 | 1.78 | 1.79 |
| Mauritius | Southeast Asia | female | 81.11 | 77.67 | 46.37 | 44.29 | 0.96 | 0.96 |
| Mexico | Central Latin America | female | 5285.36 | 7658.40 | 2996.78 | 4337.20 | 1.45 | 1.45 |
| Republic of Moldova | Eastern Europe | female | 841.26 | 590.67 | 553.95 | 374.82 | 0.70 | 0.68 |
| Mongolia | Central Asia | female | 404.94 | 606.03 | 281.72 | 398.69 | 1.50 | 1.42 |
| Montenegro | Central Europe | female | 108.56 | 85.89 | 68.57 | 53.34 | 0.79 | 0.78 |
| Morocco | North Africa and Middle East | female | 5237.78 | 6653.05 | 3359.05 | 4185.51 | 1.27 | 1.25 |
| Mozambique | Eastern Sub-Saharan Africa | female | 1002.85 | 1975.27 | 595.35 | 1177.09 | 1.97 | 1.98 |
| Myanmar | Southeast Asia | female | 3265.09 | 4155.98 | 1968.80 | 2487.44 | 1.27 | 1.26 |
| Namibia | Southern sub-Saharan Africa | female | 130.88 | 218.97 | 77.00 | 127.95 | 1.67 | 1.66 |
| Nepal | South Asia | female | 2150.00 | 3249.71 | 1294.01 | 1890.31 | 1.51 | 1.46 |
| Netherlands | Western Europe | female | 9889.83 | 8951.40 | 7958.61 | 6850.42 | 0.91 | 0.86 |
| New Zealand | Australasia | female | 1685.09 | 1817.99 | 1692.36 | 1753.64 | 1.08 | 1.04 |
| Nicaragua | Central Latin America | female | 232.12 | 410.57 | 130.49 | 231.08 | 1.77 | 1.77 |
| Niger | Western sub-Saharan Africa | female | 743.31 | 1802.06 | 466.54 | 1168.39 | 2.42 | 2.50 |
| Nigeria | Western sub-Saharan Africa | female | 9067.39 | 18077.64 | 5895.75 | 11072.88 | 1.99 | 1.88 |
| Democratic People's Republic of Korea | East Asia | female | 4222.26 | 4282.27 | 3343.35 | 3338.82 | 1.01 | 1.00 |
| Northern Mariana Islands | Oceania | female | 4.57 | 3.04 | 2.58 | 1.78 | 0.67 | 0.69 |
| Norway | Western Europe | female | 2054.36 | 2267.99 | 1712.50 | 1846.51 | 1.10 | 1.08 |
| Oman | North Africa and Middle East | female | 251.61 | 529.51 | 155.12 | 301.32 | 2.10 | 1.94 |
| Pakistan | South Asia | female | 11828.58 | 23529.30 | 7426.24 | 14685.55 | 1.99 | 1.98 |
| Palestine | North Africa and Middle East | female | 364.20 | 788.71 | 234.92 | 479.80 | 2.17 | 2.04 |
| Panama | Central Latin America | female | 132.93 | 208.42 | 72.42 | 113.82 | 1.57 | 1.57 |
| Papua New Guinea | Oceania | female | 426.33 | 1013.54 | 262.32 | 631.09 | 2.38 | 2.41 |
| Paraguay | Tropical Latin America | female | 891.15 | 1687.31 | 674.29 | 1294.85 | 1.89 | 1.92 |
| Peru | Andean Latin America | female | 5054.42 | 7690.21 | 3872.33 | 5693.66 | 1.52 | 1.47 |
| Philippines | Southeast Asia | female | 4146.31 | 7115.07 | 2390.45 | 4167.72 | 1.72 | 1.74 |
| Poland | Central Europe | female | 7374.33 | 6285.48 | 4880.22 | 4282.84 | 0.85 | 0.88 |
| Portugal | Western Europe | female | 6715.81 | 6049.81 | 5561.30 | 4775.44 | 0.90 | 0.86 |
| Puerto Rico | Caribbean | female | 536.45 | 424.18 | 317.89 | 249.44 | 0.79 | 0.78 |
| Qatar | North Africa and Middle East | female | 53.89 | 259.59 | 31.48 | 147.10 | 4.82 | 4.67 |
| Romania | Central Europe | female | 3960.96 | 2448.31 | 2502.95 | 1478.24 | 0.62 | 0.59 |
| Russian Federation | Eastern Europe | female | 25737.87 | 21813.68 | 16242.49 | 13647.42 | 0.85 | 0.84 |
| Rwanda | Eastern Sub-Saharan Africa | female | 401.68 | 745.17 | 219.41 | 412.13 | 1.86 | 1.88 |
| Saint Lucia | Caribbean | female | 20.24 | 26.66 | 12.53 | 16.16 | 1.32 | 1.29 |
| Saint Vincent and the Grenadines | Caribbean | female | 15.95 | 16.29 | 10.01 | 9.95 | 1.02 | 0.99 |
| Samoa | Oceania | female | 13.73 | 18.09 | 8.16 | 10.95 | 1.32 | 1.34 |
| Sao Tome and Principe | Central sub-Saharan Africa | female | 8.58 | 14.83 | 4.82 | 8.34 | 1.73 | 1.73 |
| Saudi Arabia | North Africa and Middle East | female | 2568.38 | 5518.73 | 1627.76 | 3148.95 | 2.15 | 1.93 |
| Senegal | Western sub-Saharan Africa | female | 652.48 | 1204.55 | 383.45 | 716.66 | 1.85 | 1.87 |
| Serbia | Central Europe | female | 1701.38 | 1181.62 | 1096.10 | 738.90 | 0.69 | 0.67 |
| Seychelles | Southeast Asia | female | 4.23 | 5.41 | 2.37 | 3.04 | 1.28 | 1.28 |
| Sierra Leone | Western sub-Saharan Africa | female | 332.57 | 684.66 | 195.10 | 405.21 | 2.06 | 2.08 |
| Singapore | High-income Asia Pacific | female | 365.09 | 546.98 | 203.91 | 302.11 | 1.50 | 1.48 |
| Slovakia | Central Europe | female | 992.12 | 770.52 | 643.81 | 476.94 | 0.78 | 0.74 |
| Slovenia | Central Europe | female | 349.59 | 252.70 | 220.20 | 153.35 | 0.72 | 0.70 |
| Solomon Islands | Oceania | female | 33.08 | 66.58 | 20.59 | 41.57 | 2.01 | 2.02 |
| Somalia | Eastern Sub-Saharan Africa | female | 505.30 | 1431.52 | 298.02 | 911.36 | 2.83 | 3.06 |
| South Africa | Southern sub-Saharan Africa | female | 3687.33 | 5266.89 | 2147.13 | 3037.47 | 1.43 | 1.41 |
| Republic of Korea | High-income Asia Pacific | female | 5956.16 | 5528.06 | 3526.03 | 3140.78 | 0.93 | 0.89 |
| South Sudan | Eastern Sub-Saharan Africa | female | 349.30 | 564.84 | 194.31 | 322.54 | 1.62 | 1.66 |
| Spain | Western Europe | female | 23982.05 | 25070.58 | 19306.84 | 19244.15 | 1.05 | 1.00 |
| Sri Lanka | Southeast Asia | female | 1971.08 | 2040.98 | 1339.25 | 1357.53 | 1.04 | 1.01 |
| Sudan | North Africa and Middle East | female | 3844.32 | 6679.40 | 2470.73 | 4069.24 | 1.74 | 1.65 |
| Suriname | Caribbean | female | 55.62 | 79.21 | 33.58 | 47.51 | 1.42 | 1.41 |
| Eswatini | Southern sub-Saharan Africa | female | 80.06 | 111.09 | 48.23 | 65.39 | 1.39 | 1.36 |
| Sweden | Western Europe | female | 4243.46 | 4419.01 | 2929.15 | 2989.13 | 1.04 | 1.02 |
| Switzerland | Western Europe | female | 4147.81 | 4497.86 | 3168.72 | 3336.36 | 1.08 | 1.05 |
| Syrian Arab Republic | North Africa and Middle East | female | 2184.32 | 2580.44 | 1384.40 | 1581.98 | 1.18 | 1.14 |
| Taiwan (Province of China) | East Asia | female | 3370.30 | 2987.01 | 2309.31 | 1999.23 | 0.89 | 0.87 |
| Tajikistan | Central Asia | female | 933.92 | 1629.75 | 648.90 | 1102.71 | 1.75 | 1.70 |
| United Republic of Tanzania | Eastern Sub-Saharan Africa | female | 1642.29 | 3459.12 | 914.44 | 1975.92 | 2.11 | 2.16 |
| Thailand | Southeast Asia | female | 4197.23 | 4373.08 | 2381.86 | 2500.26 | 1.04 | 1.05 |
| Bahamas | Caribbean | female | 40.04 | 56.73 | 23.89 | 33.75 | 1.42 | 1.41 |
| Gambia | Western sub-Saharan Africa | female | 87.13 | 182.62 | 51.72 | 106.96 | 2.10 | 2.07 |
| Timor-Leste | Southeast Asia | female | 58.08 | 82.47 | 34.88 | 48.65 | 1.42 | 1.39 |
| Togo | Western sub-Saharan Africa | female | 306.62 | 637.44 | 176.29 | 367.97 | 2.08 | 2.09 |
| Tonga | Oceania | female | 8.93 | 9.04 | 5.35 | 5.43 | 1.01 | 1.01 |
| Trinidad and Tobago | Caribbean | female | 169.39 | 178.68 | 99.99 | 104.37 | 1.05 | 1.04 |
| Tunisia | North Africa and Middle East | female | 1568.11 | 1990.50 | 977.41 | 1210.92 | 1.27 | 1.24 |
| Turkey | North Africa and Middle East | female | 16259.06 | 19764.79 | 11888.24 | 14213.53 | 1.22 | 1.20 |
| Turkmenistan | Central Asia | female | 643.85 | 737.52 | 433.08 | 469.36 | 1.15 | 1.08 |
| Uganda | Eastern Sub-Saharan Africa | female | 1077.89 | 2350.29 | 602.93 | 1322.44 | 2.18 | 2.19 |
| Ukraine | Eastern Europe | female | 9242.62 | 6839.15 | 5895.70 | 4269.79 | 0.74 | 0.72 |
| United Arab Emirates | North Africa and Middle East | female | 222.38 | 940.34 | 127.30 | 531.62 | 4.23 | 4.18 |
| United Kingdom | Western Europe | female | 31105.74 | 30875.71 | 22309.49 | 21101.21 | 0.99 | 0.95 |
| United States of America | High-income North America | female | 70059.99 | 71178.87 | 77148.90 | 74266.28 | 1.02 | 0.96 |
| Uruguay | Southern Latin America | female | 873.38 | 928.94 | 703.12 | 700.72 | 1.06 | 1.00 |
| Uzbekistan | Central Asia | female | 3800.18 | 5730.66 | 2604.47 | 3757.19 | 1.51 | 1.44 |
| Vanuatu | Oceania | female | 15.30 | 28.81 | 9.41 | 17.69 | 1.88 | 1.88 |
| Venezuela (Bolivarian Republic of) | Central Latin America | female | 1083.14 | 1591.05 | 592.66 | 873.86 | 1.47 | 1.47 |
| Viet nam | Southeast Asia | female | 4951.65 | 6713.56 | 2923.62 | 3925.63 | 1.36 | 1.34 |
| United States Virgin Islands | Caribbean | female | 16.64 | 11.74 | 9.93 | 6.88 | 0.71 | 0.69 |
| Yemen | North Africa and Middle East | female | 2361.89 | 5235.31 | 1531.94 | 3249.65 | 2.22 | 2.12 |
| Zambia | Eastern Sub-Saharan Africa | female | 493.04 | 1094.68 | 275.25 | 619.43 | 2.22 | 2.25 |
| Zimbabwe | Southern sub-Saharan Africa | female | 940.48 | 1472.47 | 556.54 | 894.27 | 1.57 | 1.61 |
| Monaco | Western Europe | female | 16.89 | 16.89 | 13.11 | 12.53 | 1.00 | 0.96 |
| San Marino | Western Europe | female | 13.90 | 18.69 | 10.76 | 14.00 | 1.35 | 1.30 |
| Saint Kitts and Nevis | Caribbean | female | 5.64 | 8.45 | 3.42 | 5.04 | 1.50 | 1.47 |
| Cook Islands | Oceania | female | 1.63 | 1.40 | 0.94 | 0.81 | 0.86 | 0.86 |
| Nauru | Oceania | female | 0.86 | 0.88 | 0.49 | 0.51 | 1.03 | 1.03 |
| Niue | Oceania | female | 0.19 | 0.13 | 0.11 | 0.07 | 0.67 | 0.67 |
| Palau | Oceania | female | 1.42 | 1.27 | 0.81 | 0.73 | 0.90 | 0.90 |
| Tokelau | Oceania | female | 0.14 | 0.11 | 0.08 | 0.06 | 0.78 | 0.77 |
| Tuvalu | Oceania | female | 0.99 | 0.97 | 0.59 | 0.57 | 0.98 | 0.97 |

**Supplementary Table 7. The top three and the bottom three countries of global psoriasis burden of young adults.**

| Measure | | sex | Top three countries |  |  | Bottom three countries |  |  |
| --- | --- | --- | --- | --- | --- | --- | --- | --- |
| 2019 ASR (per 100,000 people) | |  |  |  |  |  |  |  |
| ASIR | |  |  |  |  |  |  |  |
|  | | both | Iceland(254.83) | Finland(257.78) | France(277.24) | Indonesia(13.36) | Panama(19.57) | Costa Rica(19.82) |
|  | female | Iceland(248.27) | Finland(251.19) | France(273.97) | Indonesia(13.77) | Panama(20.02) | Costa Rica(20.25) |  |
|  | | male | Iceland(260.63) | Finland(263.99) | France(281.00) | Indonesia(12.95) | Panama(19.13) | Costa Rica(19.36) |
| Age-standardized DALY rate | |  |  |  |  |  |  |  |
|  | | both | Iceland(200.04) | Finland(202.81) | France(228.64) | Indonesia(7.26) | Panama(10.77) | Malaysia(10.90) |
|  | | female | Iceland(197.30) | Finland(199.58) | France(230.42) | Indonesia(7.93) | Panama(10.93) | Costa Rica(11.09) |
|  | | male | Iceland(202.57) | Finland(205.89) | France(227.28) | Indonesia(6.60) | Malaysia(10.25) | Panama(10.61) |
| 1990-2019 increase times | |  |  |  |  |  |  |  |
| Incidence (cases) | |  |  |  |  |  |  |  |
|  | | both | Afghanistan(3.20) | United Arab Emirates(4.94) | Qatar(5.90) | Bosnia and Herzegovina(0.51) | Latvia(0.55) | Georgia(0.56) |
|  | | female | Kuwait(2.92) | United Arab Emirates(4.23) | Qatar(4.82) | Bosnia and Herzegovina(0.51) | Georgia(0.53) | Latvia(0.53) |
|  | | male | Maldives(4.04) | United Arab Emirates(5.23) | Qatar(6.32) | Bosnia and Herzegovina(0.51) | Latvia(0.57) | Lithuania(0.57) |
| DALY (cases) | |  |  |  |  |  |  |  |
|  | | both | Afghanistan(3.16) | United Arab Emirates(4.94) | Qatar(5.69) | Bosnia and Herzegovina(0.48) | Latvia(0.53) | Lithuania(0.54) |
|  | | female | Somalia(3.06) | United Arab Emirates(4.18) | Qatar(4.67) | Bosnia and Herzegovina(0.48) | Latvia(0.51) | Georgia(0.52) |
|  | | male | Maldives(3.89) | United Arab Emirates(5.26) | Qatar(6.09) | Bosnia and Herzegovina(0.49) | Lithuania(0.55) | Latvia(0.55) |
| EAPC | |  |  |  |  |  |  |  |
| Incidence | |  |  |  |  |  |  |  |
|  | | both | Somalia(-0.09) | Sweden(-0.08) | Japan(0.04) | Equatorial Guinea(-1.86) | Saudi Arabia(-1.35) | Oman(-1.22) |
|  | | female | Kiribati(-0.11) | Sweden(-0.07) | Japan(-0.04) | Equatorial Guinea(-1.86) | Saudi Arabia(-1.33) | Oman(-1.18) |
|  | | male | Sweden(-0.08) | Somalia(-0.05) | Japan(0.11) | Equatorial Guinea(-1.83) | Saudi Arabia(-1.37) | Oman(-1.23) |
| DALY | |  |  |  |  |  |  |  |
|  | | both | Kiribati(-0.01) | Japan(0.04) | Somalia(0.14) | Equatorial Guinea(-2.40) | Saudi Arabia(-1.70) | Oman(-1.49) |
|  | | female | Japan(-0.04) | Kiribati(0.01) | Somalia(0.13) | Equatorial Guinea(-2.42) | Saudi Arabia(-1.68) | Oman(-1.45) |
|  | | male | Zimbabwe(0.01) | Japan(0.12) | Somalia(0.15) | Equatorial Guinea(-2.37) | Saudi Arabia(-1.71) | Oman(-1.50) |

**Supplementary Table 8. The top three and the bottom three regions of global psoriasis burden of young adults.**

| Measure | sex | Top three regions |  |  | Bottom three regions |  |  |
| --- | --- | --- | --- | --- | --- | --- | --- |
| 2019 ASR (per 100,000 people) |  |  |  |  |  |  |  |
| ASIR |  |  |  |  |  |  |  |
|  | both | Southern Latin America(109.43) | Australasia(155.56) | Western Europe(226.20) | Southeast Asia(20.50) | Central Latin America(21.59) | Eastern Sub-Saharan Africa(25.85) |
|  | female | Southern Latin America(109.51) | Australasia(159.26) | Western Europe(218.64) | Southeast Asia(20.93) | Central Latin America(22.07) | Eastern Sub-Saharan Africa(26.55) |
|  | male | Southern Latin America(109.47) | Australasia(152.17) | Western Europe(233.73) | Southeast Asia(20.09) | Central Latin America(21.08) | Eastern Sub-Saharan Africa(25.11) |
| Age-standardized DALY rate |  |  |  |  |  |  |  |
|  | both | High-income North America(93.10) | Australasia(138.18) | Western Europe(172.50) | Southeast Asia(11.42) | Central Latin America(12.20) | Eastern Sub-Saharan Africa(15.18) |
|  | female | High-income North America(96.64) | Australasia(149.98) | Western Europe(168.48) | Southeast Asia(12.28) | Central Latin America(12.37) | Eastern Sub-Saharan Africa(15.44) |
|  | male | High-income North America(89.78) | Australasia(126.56) | Western Europe(176.73) | Southeast Asia(10.59) | Central Latin America(12.02) | Eastern Sub-Saharan Africa(14.91) |
| 1990-2019 increase times |  |  |  |  |  |  |  |
| Incidence (cases) |  |  |  |  |  |  |  |
|  | both | Oceania(2.02) | Central sub-Saharan Africa(2.14) | Eastern Sub-Saharan Africa(2.15) | Central Europe(0.75) | Eastern Europe(0.81) | High-income Asia Pacific(0.89) |
|  | female | Western sub-Saharan Africa(2.06) | Central sub-Saharan Africa(2.11) | Eastern Sub-Saharan Africa(2.12) | Central Europe(0.72) | Eastern Europe(0.80) | High-income Asia Pacific(0.85) |
|  | male | Oceania(2.01) | Central sub-Saharan Africa(2.16) | Eastern Sub-Saharan Africa(2.18) | Central Europe(0.77) | Eastern Europe(0.82) | High-income Asia Pacific(0.93) |
| DALY (cases) |  |  |  |  |  |  |  |
|  | both | Oceania(2.04) | Central sub-Saharan Africa(2.11) | Eastern Sub-Saharan Africa(2.19) | Central Europe(0.74) | Eastern Europe(0.80) | High-income Asia Pacific(0.88) |
|  | female | Oceania(2.05) | Central sub-Saharan Africa(2.08) | Eastern Sub-Saharan Africa(2.15) | Central Europe(0.72) | Eastern Europe(0.79) | High-income Asia Pacific(0.84) |
|  | male | Oceania(2.02) | Central sub-Saharan Africa(2.14) | Eastern Sub-Saharan Africa(2.23) | Central Europe(0.76) | Eastern Europe(0.82) | High-income Asia Pacific(0.92) |
| EAPC |  |  |  |  |  |  |  |
| Incidence |  |  |  |  |  |  |  |
|  | both | Tropical Latin America(-0.20) | Western Europe(-0.20) | High-income Asia Pacific(-0.08) | North Africa and Middle East(-0.93) | Western sub-Saharan Africa(-0.85) | East Asia(-0.84) |
|  | female | Tropical Latin America(-0.20) | Australasia(-0.19) | High-income Asia Pacific(-0.16) | North Africa and Middle East(-0.94) | Western sub-Saharan Africa(-0.89) | East Asia(-0.82) |
|  | male | Tropical Latin America(-0.20) | Western Europe(-0.19) | High-income Asia Pacific(-0.01) | North Africa and Middle East(-0.93) | East Asia(-0.83) | Western sub-Saharan Africa(-0.83) |
| DALY |  |  |  |  |  |  |  |
|  | both | South Asia(-0.17) | Tropical Latin America(-0.15) | High-income Asia Pacific(-0.12) | North Africa and Middle East(-1.11) | East Asia(-0.99) | Western sub-Saharan Africa(-0.95) |
|  | female | High-income Asia Pacific(-0.20) | South Asia(-0.18) | Tropical Latin America(-0.16) | North Africa and Middle East(-1.10) | Western sub-Saharan Africa(-0.97) | East Asia(-0.96) |
|  | male | South Asia(-0.16) | Tropical Latin America(-0.15) | High-income Asia Pacific(-0.05) | North Africa and Middle East(-1.11) | East Asia(-0.99) | Western sub-Saharan Africa(-0.92) |
